# Crystal Correlation Of Heterocyclic Imidazo[1,2-*a*]pyridine Analogues and Their Anticholinesterase Potential Evaluation

**DOI:** 10.1038/s41598-018-37486-7

**Published:** 2019-01-30

**Authors:** Huey Chong Kwong, C. S. Chidan Kumar, Siau Hui Mah, Yew Leng Mah, Tze Shyang Chia, Ching Kheng Quah, Gin Keat Lim, Siddegowda Chandraju

**Affiliations:** 1 School of Chemical Sciences, Universiti Sains Malaysia, Penang, 11800 USM Malaysia; 2 Department of Engineering Chemistry, Vidya Vikas Institute of Engineering & Technology, Visvesvaraya Technological University, Alanahalli, Mysuru, 570028 Karnataka India; 3 School of Biosciences, Taylor’s University, Lakeside Campus, 47500 Subang Jaya, Selangor Malaysia; 4 Hospital Pulau Pinang, Jalan Residensi, 10990 George Town, Pulau Pinang, Malaysia; 5 X-ray Crystallography Unit, School of Physics, Universiti Sains Malaysia, Penang, 11800 USM Malaysia; 6 Mandavya Pre University College, Mandya, 571403 India

## Abstract

Imidazo[1,2-*a*]pyridine-based compounds are clinically important to the treatments of heart and circulatory failures, while many are under development for pharmaceutical uses. In this study, a series of imidazo[1,2-*a*]pyridine-based derivatives **2**(**a**–**o**) were synthesized by reacting *a*-haloketones with 2-aminopyridines in a basic media at ambient temperature. Single crystal X-ray diffraction studies suggest that with low degree-of-freedom, the introduction of bulky adamantyl or electron-rich biphenyl moiety into the imidazopyridine derivatives will not affect its structural occupancy. Imidazo[1,2-*a*]pyridine-based derivatives with biphenyl side chain are potential AChE inhibitors. Compound **2h** which bears a biphenyl side chain and methyl substituent at the position **R**_**4**_ of the imidazo[1,2-*a*]pyridine ring showed the strongest AChE inhibition with an IC_50_ value of 79 µM. However, imidazo[1,2-*a*]pyridine derivatives with phenyl side chain exhibit better BChE inhibition effect among the series. Compound **2j** with 3,4-dichlorophenyl side chain and unsubstituted imidazo[1,2-*a*]pyridine ring appears to be the strongest BChE inhibitor with an IC_50_ value of 65 µM and good selectivity. The inhibitory effects of active compounds were further confirmed by computational molecular docking studies. The results unveiled that peripheral anionic sites of AChE and acyl pocket of BChE were the predominated binding sites for the subjected inhibitors.

## Introduction

Imidazo[1,2-*a*]pyridines are probably the most common nitrogen-bridgehead fused heterocycles used in pharmacology research, attributed to their activities spanning a diverse range of targets. These molecules exhibit antiviral^1^, antibacterial^2^, antiparasitic^3^, anti-inflammatory^4^ and antipyretic^5^ properties. Besides that, they also served as *β*-amyloid formation inhibitor, GABA_A_ and benzodiazepine receptor agonists^6^ and cardiotonic agent^7^. Currently, four imidazo[1,2-*a*]pyridine-based derivatives are widely used in clinic, including hypnotic drug Zolpidem^8^, non-sedative anxiolytic drug Alpidem, antiulcer agent Zolmidine^9^ and phosphodiesterase III inhibitor Olprinone for the treatment of heart and circulatory failures^10^.

Aside from the progressive illness mentioned above, Alzheimer’s disease (AD) is a fatal brain degenerative disease that starts with dementia and loss of language, problem-solving and cognitive skills of a person. AD is lethal after a prolonged period of struggle and suffering^11^. Although the key nature of the pathophysiology of AD is still unclear, “cholinergic hypothesis” had been developed from the studies based on basal forebrain and rostral forebrain’s cholinergic pathway. Based on the cholinergic hypothesis, cholinesterase inhibitor is used to enhance cognitive function and slowdown AD progression by inhibiting the hydrolysis of cholinergic neurotransmitters^12^. There are two types of cholinesterases, which are acetylcholinesterase (AChE) and butyrylcholinesterase (BChE) where these enzymes are responsible for the hydrolysis of the neurotransmitter acetylcholine into choline and acetic acid. As early as at the year of 1990s, a series of imidazo[1,2-*a*]pyridines derivatives (2-arylimidazo[1,2,-*a*]pyridinium salts) were subjected for the evaluation of electric eel acetylcholinesterase (AChE) inhibition. These compounds showed positive results with IC_50_ values ranging from 0.2 to 50.0 μM, meanwhile several of them showed protective effects against the organophosphorus AChE inhibitor soman in mice^13^. Besides, *N*-(benzylidene)imidazo[1,2-*a*]pyridine derivatives with halogen as substituent on the attached phenyl ring, showed potential AChE inhibition^14^. Recently, the cholinesterase inhibition effects of imidazole analogues designed to be multi-targeted, were confirmed through computational studies, pharmacological evaluation using animal model and mechanistic *in-vitro* cholinesterase inhibition study^15^.

In view of the potential cholinesterase inhibition activities associated with the imidazo[1,2-*a*]pyridine ring system, it led to our present study on the synthesis of fifteen new imidazo[1,2-*a*]pyridine-based derivatives, **2**(**a**–**o**) and evaluation of their anti-cholinesterase activities. These new derivatives were characterized by spectroscopy methods and single crystal X-diffraction study. By using group comparison of single-crystal X-ray diffraction data, the structural conformation, structural occupancy and crystal packing similarity were studied. The AChE and BChE inhibitory activities of these derivatives were evaluated by Ellman’s colorimetric test^16^. In addition, we also investigated *in-silico* binding mode of the biologically active ligands into AChE and BChE enzymes in comparison with tacrine as reference and protocol validation. The molecular docking procedure was validated by using tacrine as the native ligand.

## Results and Discussion

### Spectroscopy analysis

Cyclization of **2**(**a**-**o**) was confirmed by FTIR, as the N-H stretching bonds were in the range of 3500–3300 cm^−1^, which corresponded to the disappearance of primary amide. Furthermore, some peaks were observed at ~1370 and ~1200 cm^−1^, which corresponded to the C-N stretching of imidazole. The presence of unsaturated C-H (pyridine) stretching was observed near 3100 cm^−1^, and their *v*(C-C) and *ω*(C-H) were showed at ~1600 & 1450 cm^−1^ and ~750 cm^−1^, respectively. Imidazo[1,2-*a*]pyridines-based derivatives contain adamantyl, biphenyl or phenyl moiety as their side chains. The signature absorption bands of these moieties can be identified from their FTIR spectra. Adamantyl’s methylene and methine *v*(C-H) were observed as strong bands around 2900 and 2850 cm^−1^. Meanwhile, the *v*(C-H) of biphenyl and phenyl moieties were revealed at ~3000 cm^−1^. In the FTIR spectra of **2**(**a**–**o**), the unexpected observations are the occurrences of O-H stretching in hydrated compounds **2b**, **2c** and **2e**, as these compounds were co-crystallized with water molecules^17,18^.

In the ^1^H-NMR spectra of **2**(**a**-**o**), the singlet corresponding to the methylene (-CH_2_-) group in **1** was replaced by methine (-CH-) protons at a higher chemical shift (*δ* ≈ 7.1–8.2 ppm) which associated with the other required aromatic peaks. For compounds **2**(**a**-**e**), adamantyl moiety was presented as broad peaks in the lower region, centering around *δ* ≈ 2.1, 2.0 and 1.8 ppm with the integration values of 3:6:6. For compounds **2**(**f**-**i**), the first phenyl ring of the biphenyl moiety was shown as doublet and two sets of triplet near *δ* ≈ 7.7 − 7.4 ppm with the integration values of 2:2:1. Two well-resolved sets of doublet were centered around *δ* ≈ 8.2 and 7.7 ppm with the integration values of 2:2, ascribing to the -CH- protons of second phenyl ring. Identical *J*-coupling values were used to distinguish different methine protons from imidazo[1,2-*a*]pyridine, biphenyl and phenyl ring systems. In addition, protons of -CH_3_ and -OCH_3_ groups were revealed in the up-field region near *δ* ≈ 2.5 and 3.9 ppm. The numbers of protons were in agreement with the proposed values based on the integration values.

The ^13^C NMR spectra of **2**(**a**-**o**) showed four distinct sets of imidazole carbon, aromatic carbon, adamantyl carbon and saturated carbon signals. In the down-field region, both *δ*(C = N) and *δ*(C-N) imidazole carbon signals were centered around *δ* ≈ 146 and 145 ppm, respectively. The aromatic carbon signals of biphenyl and benzene groups were found in the range of *δ* ≈ 140 to 108 ppm. The adamantyl carbon signals were centered at *δ* ≈ 42, 37, 34 and 28 ppm, while the -CH_3_ and -OCH_3_ saturated carbon signals were observed in the up-field region, centering at *δ* ≈ 20 and 55 ppm, respectively^18–20^. All spectra are included in Supplementary Information.

### X-ray crystal structure description

The asymmetric unit (Z’) of all studied compounds consist of a crystallographic independent molecule, except **2n** and **2o**, which consist of two molecules, while compound **2k** consists of three molecules. Besides, the molecules of **2b** and **2e** lay on the mirror plane, therefore the asymmetric unit of **2b** and **2e** consist of half molecule. In addition, compounds **2b**, **2c** and **2e** are crystallized with an extra water molecule as its solvate. Degree–of–freedom in the molecular conformations of the reported compounds can be characterized by the torsion angles between the imidazopyridine group with its attached adamantyl, biphenyl or phenyl groups C1–C7–C8–C9, τ1 (an extra torsion angle of C10–C11–C14–C15, τ2 for compounds containing biphenyl moiety, **2f**-**i**).

**Figure 1:**
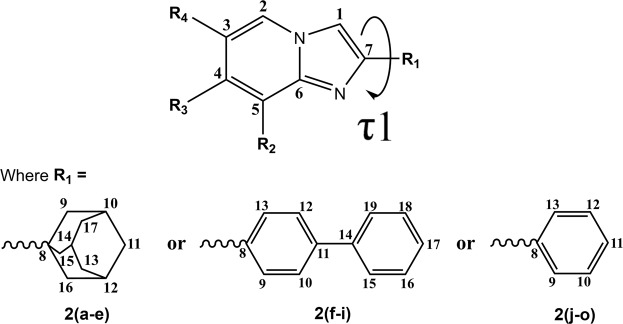
General scheme of imidazo[1,2-*a*]pyridine derivatives with torsion angle τ1.

For imidazo[1,2-*a*]pyridine derivatives with adamantyl side chain (**2a**–**e**), the torsion angles of C1–C7–C8–C9, τ1 are in the range of 0.0–7.1° (Fig. 1), while the imidazopyridine moiety is coplanar to the center of adamantyl (C9–C10–C13/14) with dihedral angle of 0.0–5.3°. Whereas, for compounds with biphenyl side chain (**2f**–**i**), the imidazopyridine and both phenyl rings are almost coplanar to each other with dihedral angles of 2.0–5.7° and 1.1–4.8°, respectively, as the torsion angle of τ1 and C10–C11–C14–C15, τ2 were nearly parallel with the range between 179.3–174.9° and 1.3–4.8°. As for imidazo[1,2-*a*]pyridine derivatives with phenyl side chain (**2j**-**o**), the phenyl ring had twisted away from the imidazopyridine ring, as the torsion angles of τ1 vary from 178.9° to 165.2°. Overall, the torsion angles in between imidazopyridine ring and its side chain (τ1) are always in *periplanar* conformation, although the dihedral angles have shown a discrepancy up to 25.7°. Selected torsional and dihedral angles for the reported compounds are tabulated in Table 1.

**Table 1:** Selected torsional* and dihedral^#^ angles (°) for compounds **2a**–**o**.

Compound	τ1	τ2	Dihedral 1	Dihedral 2
**2a**	−3.4	—	3.6	—
**2b**	0.0	—	0.0	—
**2c**	7.1	—	5.3	—
**2e**	0.0	—	0.0	—
**2f**	179.3	−4.7	3.5	4.00
**2g**	−174.9	−4.8	5.7	2.7
**2h**	178.4	2.6	2.0	1.09
**2i**	−176.1	1.3	3.6	4.83
**2j**	176.2	—	5.4	—
**2k**	−175.1, 166.6, −165.2	—	5.7, 13.6, 11.9	—
**2l**	167.6	—	12.44	—
**2m**	−169.0	—	10.0	—
**2n**	175.4, 177.4	—	6.0, 25.7	—
**2o**	−175.6, 178.9	—	4.9, 3.9	—

*τ1 = Torsion angle of C1–C7–C8–C9; τ2 = Torsion angle of C10–C11–C14–C15.

^#^Dihedral 1 represents the dihedral angle between the mean plane of imidazopyridine and center of adamantyl (C9–C10–C13/14) or the first phenyl ring. Dihedral 2 represents the dihedral angle between the mean planes of the imidazole and the second phenyl ring.

### Crystal packing similarity and structural occupancy

**Figure 2:**
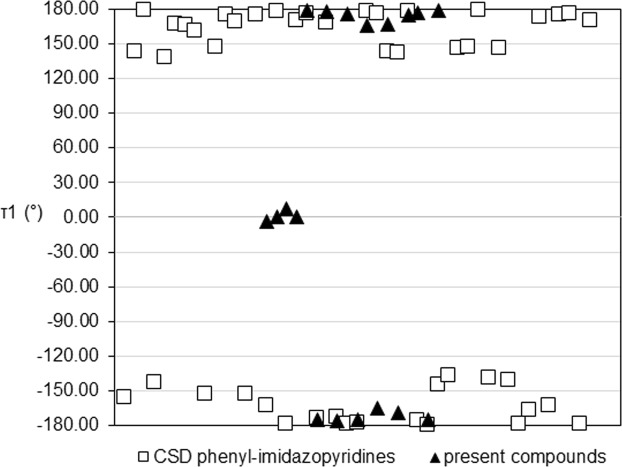
Graphical representation of the C1–C7–C8–C9 torsion angles, τ1, in present and previously reported compounds.

A Cambridge Structural Database (CSD, V5.38, last update Nov 2016^21^) search using 2-phenylimidazo[1,2*-a*]pyridine as skeleton was performed to locate previously reported phenyl-imidazopyridine derivatives and 36 similar structures were found. In order to identify the effect of the replacement of phenyl ring with relatively bulky adamantyl or electron-rich biphenyl moiety on the molecular conformation and structure occupancy, fourteen present imidazo[1,2-*a*]pyridine derivatives were compared with 36 reported phenyl-imidazopyridine derivatives. In the aspect of molecular conformation, it is noteworthy to observe that no *clinal* conformation (τ1 between ± 30 °– ± 150°) is adopted by current studied compounds, unlike the variations displayed in phenyl-imidazopyrine derivatives (Fig. 2). All present compounds adopt *periplanar* conformation (τ1 between 0–30° or 150°–180°) with C1–C7–C8–C9 torsion angles falling in the range from 0–7.1° degree and 165.2–179.3°, which is comparable to those previously reported phenyl-imidazolepyridine derivatives that adopt the same conformation (152.9–179.8°).

The comparison of crystal structure occupancy between the search results and the present compounds are listed in Table 2. In the previously reported phenacyl benzoate derivatives, the replacement of phenyl moiety with adamantyl moiety had reduced the occurrence of π···π interaction, hence reduced the crystal packing coefficient^22^. Whereas, the introduction of biphenyl moiety as a replacement for phenyl moiety in phenacyl benzoate derivative had encouraged the formation of weak intermolecular π···π and C-H···π interaction, thus increased their crystal packing coefficient^23^. In contrast to the previously reported phenacyl benzoate derivatives, replacement of phenyl moiety with the adamantyl or biphenyl moiety does not arise a direct impact on the crystal packing coefficient in the present imidazopyridine derivatives.

**Table 2:** List of structural occupancy of the present and reported compounds.

Compound	Packing coefficient (%)	Compound	Packing coefficient (%)	Compound	Packing coefficient (%)
**2a**	66.17	DABTEI^37^	65.79	OMIDEV^38^	62.42
**2b**	63.25	ECEGEA^39^	63.07	QODZUG^40^	67.41
**2c**	61.97	FEMQOF^41^	66.70	QODZUG01^40^	68.03
**2e**	64.10	HUPWIZ^42^	60.15	QODZUG02^40^	66.78
**2f**	64.32	HUPWIZ01^43^	62.60	QUQSEC.^44^	65.07
**2g**	63.17	HURZOL^45^	65.16	RELQUW^46^	64.98
**2h**	64.74	KABMIM^47^	66.21	RUJNEQ.^48^	64.61
**2i**	63.81	MIXZOJ^49^	65.44	TIDVIN^50^	66.07
**2j**	64.63	MIXZUP^51^	67.52	TUZYEU^52^	65.44
**2k**	65.36	MONREO^53^	66.12	UTITEX^54^	62.07
**2l**	67.34	NAGGEH^55^	65.27	VEGKAU^56^	64.34
**2m**	60.69	NAGGEH01^57^	64.84	WUHKER^58^	62.24
**2n**	62.87	NOGRIM^59^	65.44	YEDHIY^60^	63.06
**2o**	66.69	NONFOM^61^	64.30	ZUNVOV^62^	68.57
AHOMIV^63^	63.75	NONFOM01^61^	65.95	ZUPCOE^62^	66.04
BEGTUE^64^	62.92	NUBVUD^65^	63.87	ZUSSAJ^66^	63.31
CAJTIQ^67^	67.82	NUBWAK^65^	64.00		

**Figure 3:**
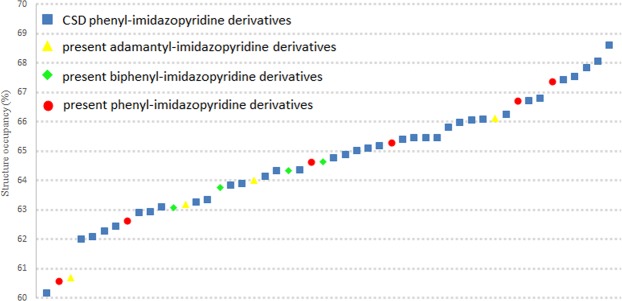
Structural occupancy comparison of the present and reported compound.

As Fig. 3 shows the structure occupancy percentage, there is no obvious pattern as their structure occupancy were scattered within the range of the reported imidazopyridine derivatives. These might arise from the existence of imidazopyridine moiety and the low degree-of-freedom in the present compounds. As mentioned above, crystal packing coefficient were under direct influence of weak π···π and C-H···π interaction. While in the present compound, the existence of imidazopyridine ring system had play a vital role in those π···π and C-H···π interaction, as it is involves in 9 out of 14 crystal packing of those compound. In addition, low degree-of-freedom had limited the packing patterns of the present compound. Therefore, molecules in the present compound tend to pack in a similar pattern with average crystal packing coefficient, which indicated by the occurrence of isostructural with 3D and 2D packing similarities.

**Figure 4:**
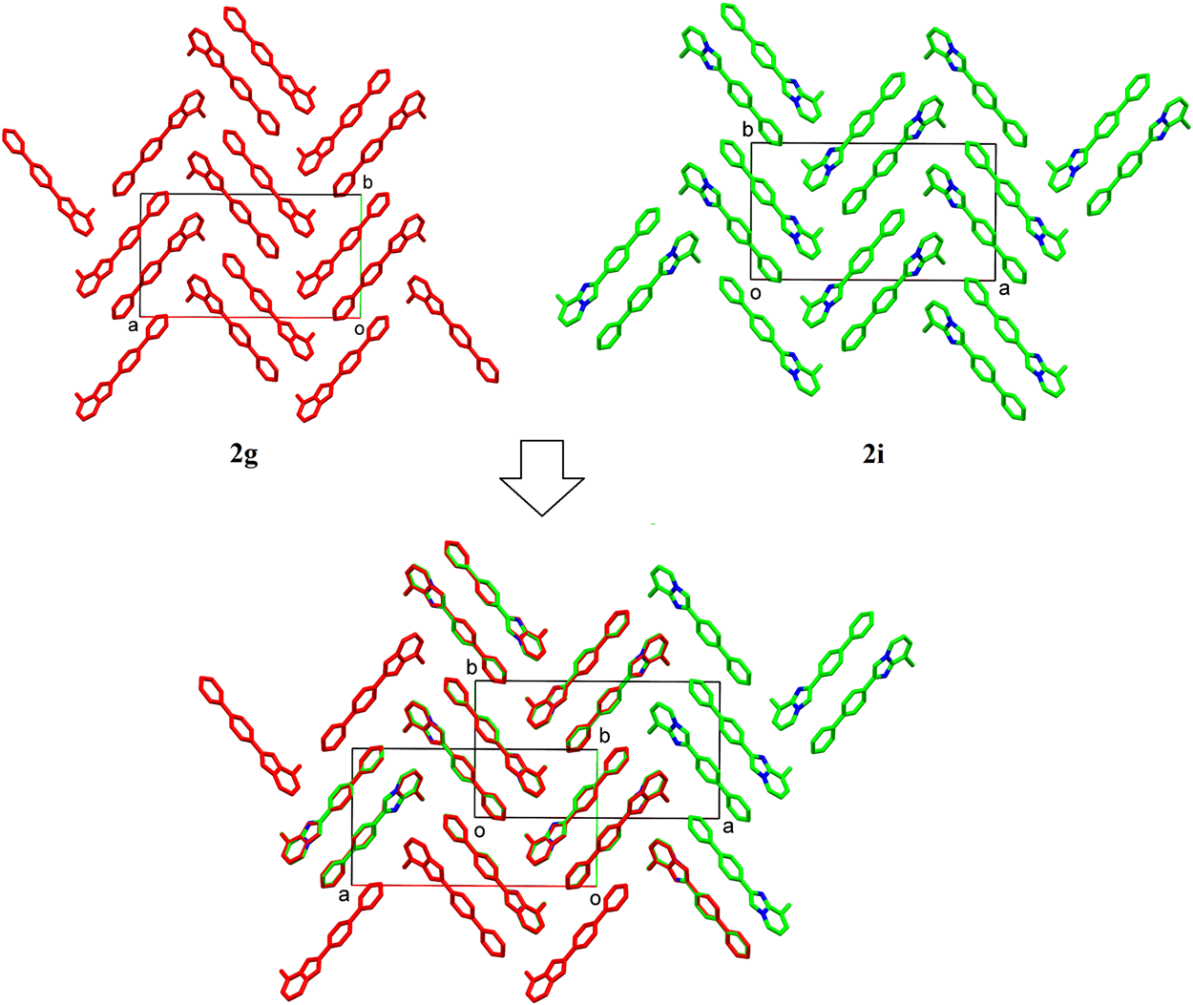
Crystal packing comparison between compounds **2g** and **2i**.

**Figure 5:**
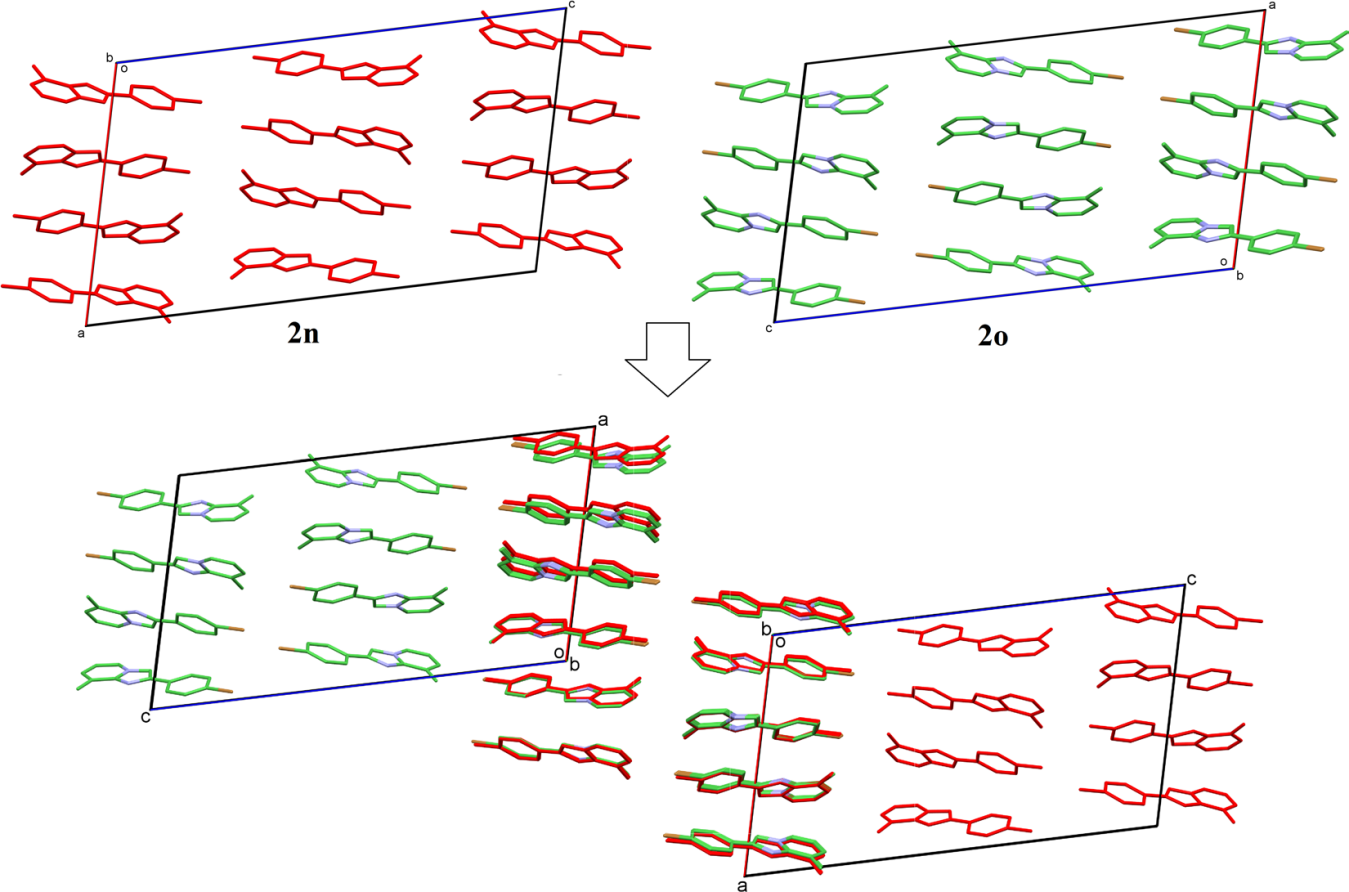
Crystal packing comparison between compounds **2n** and **2o**.

**Figure 6:**
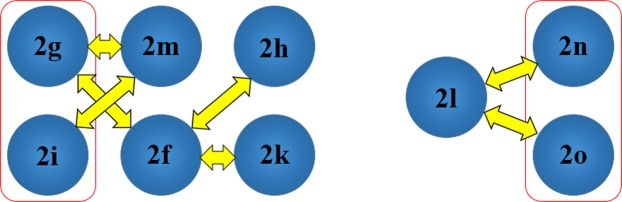
Crystal packing relationship in some studied compounds. Red boxes and yellow arrows indicate 3D and 2D similarities, respectively.

In the investigation of the crystal structural similarity among the present compounds, two pairs of compounds (**2g**/**2i** and **2n**/**2o**) were found to be crystallized in the same space group with similar lattice constants, these are the two main designations for 3D structural similarity. In fact, their crystal structures overlaid diagrams show that those two pairs of compounds as isostructure with similar packing pattern (Figs 4 and 5). Besides, 2D similarities are observed in between **2l**/**2n** and **2l**/**2o**, while compound **2f, 2g**, **2n**, **2i**, **2k** and **2m** shared also 2D similarity (Fig. 6).

### Cholinesterases Inhibitory Activities and Their Molecular Docking Studies

In the present study, all synthesized compounds were examined for their AChE and BChE inhibitory activities by Ellman’s assay^16^, and their IC_50_ values are tabulated in Table 3. The present imidazo[1,2-*a*]pyridine derivatives contain biologically active adamantyl, biphenyl or phenyl moiety as their side chains, which showed different levels of inhibitory activities towards AChE and BChE. Compounds **2a**, **2f**, **2j** and **2l** with unsubstituted imidazo[1,2-*a*]pyridine ring were synthesized as the reference compounds to accentuate the electronic effects of different substituents on the imidazo[1,2-*a*]pyridine ring, *i.e*. electron-donating (CH_3_) and electron-withdrawing (Cl) groups, upon their cholinesterase inhibitory activities.

**Table 3:** IC_50_ values of the AChE and BChE inhibitory effects of **2**(**a**–**o**).

Compounds	Substituents				IC_50_ (µM) AChE	IC_50_ (µM) BChE
**2a**	**R**_**1**_ = adamantan-1-yl	**R**_**2**_ = H	**R**_**3**_ = H	**R**_**4**_ = H	>2000	>2000
**2b**	**R**_**1**_ = adamantan-1-yl	**R**_**2**_ = CH_3_	**R**_**3**_ = H	**R**_**4**_ = H	657 ± 60	706 ± 37
**2c**	**R**_**1**_ = adamantan-1-yl	**R**_**2**_ = H	**R**_**3**_ = CH_3_	**R**_**4**_ = H	270 ± 21	>2000
**2d**	**R**_**1**_ = adamantan-1-yl	**R**_**2**_ = H	**R**_**3**_ = H	**R**_**4**_ = CH_3_	>2000	>2000
**2e**	**R**_**1**_ = adamantan-1-yl	**R**_**2**_ = H	**R**_**3**_ = Cl	**R**_**4**_ = H	>2000	>2000
**2f**	**R**_**1**_ = 1,1′-biphenyl	**R**_**2**_ = H	**R**_**3**_ = H	**R**_**4**_ = H	208 ± 17	>2000
**2g**	**R**_**1**_ = 1,1′-biphenyl	**R**_**2**_ = CH_3_	**R**_**3**_ = H	**R**_**4**_ = H	288 ± 48	315 ± 6
**2h**	**R**_**1** _=1,1′-biphenyl	**R**_**2** _=H	**R**_**3** _=H	**R**_**4** _=CH_3_	**79** ± **10**	496 ± 27
**2i**	**R**_**1**_ = 1,1′-biphenyl	**R**_**2**_ = Cl	**R**_**3**_ = H	**R**_**4**_ = H	253 ± 25	>2000
**2j**	**R**_**1**_ = 3,4-dichlorophenyl	**R**_**2**_ = H	**R**_**3**_ = H	**R**_**4**_ = H	>2000	**65** ± **13**
**2k**	**R**_**1**_ = 3,4-dichlorophenyl	**R**_**2**_ = CH_3_	**R**_**3**_ = H	**R**_**4**_ = H	>2000	191 ± 29
**2l**	**R**_**1**_ = 4-methoxyphenyl	**R**_**2**_ = H	**R**_**3**_ = H	**R**_**4**_ = H	>2000	>2000
**2m**	**R**_**1**_ = 4-methoxyphenyl	**R**_**2**_ = CH_3_	**R**_**3**_ = H	**R**_**4**_ = H	>2000	>2000
**2n**	**R**_**1**_ = 4-chlorophenyl	**R**_**2**_ = CH_3_	**R**_**3**_ = H	**R**_**4**_ = H	>2000	722 ± 48
**2o**	**R**_**1**_ = 4-bromophenyl	**R**_**2**_ = CH_3_	**R**_**3**_ = H	**R**_**4**_ = H	>2000	>2000
Tacrine					0.24 ± 0.04	0.03 ± 0.01

**Figure 7:**
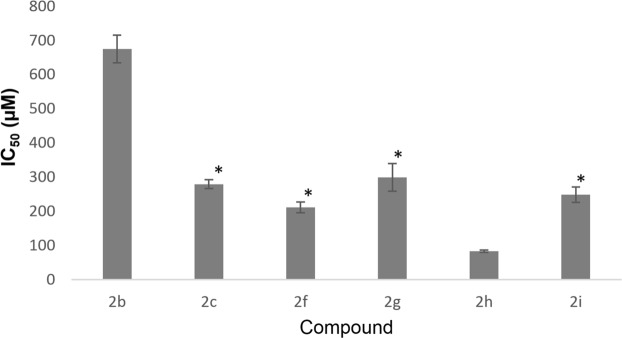
The IC_50_ for AChE inhibition in mean ± SD (n = 3). * indicate p < 0.05.

Compounds **2**(**f**-**i**) with biphenyl side chain showed good AChE inhibition, while compounds **2**(**j**-**o**) with phenyl side chain were inactive up to a concentration of 2000 μM, indicating the importance of biphenyl moiety in AChE inhibition. As compared to compound **2f** (IC_50_ = 208 µM) with unsubstituted imidazo[1,2-*a*]pyridine ring, compounds **2g** and **2i** which have substituent group at the position **R**_**2**_ showed lower AChE inhibitory effect (IC_50_ = 288 and 253 µM, respectively), whereas, compound **2h** with methyl substituent at the position **R**_**4**_ of imidazo[1,2-*a*]pyridine ring showed the highest AChE inhibition activity (IC_50_ = 79 µM). On the other hand, 2-(adamantan-1-yl)imidazo[1,2-*a*]pyridine derivatives **2**(**a**-**e**) exhibited moderate to weak AChE inhibition activities. Compound **2c** with methyl group substituted at the position **R**_**3**_ of imidazo[1,2-*a*]pyridine ring has an IC_50_ value of 270 µM, which is statistically comparable to compounds **2f**, **2g** and **2i** (Fig. 7). Whereas, compound **2b** with substituted methyl group at the position **R**_**2**_ of imidazo[1,2-*a*]pyridine ring showed even lower AChE inhibition activity (IC_50_ = 657 µM) as compared to **2c**.

In contrast to the inactive activity in AChE inhibition, derivatives with phenyl side chain gave better BChE inhibition as compared to the adamantyl or biphenyl side chain if their phenyl side chains are substituted with strong electron-withdrawing Cl group, *i.e*. compounds **2j**, **2k** and **2n**. Compounds **2j** and **2k** bearing with 3,4-dichlorophenyl side chain are two strongest BChE inhibitors with IC_50_ values of 65 and 191 µM, respectively. There is a significant difference in the BChE inhibition activities of compounds **2n** (IC_50_ = 722 µM) and **2o** (IC_50_ > 2000 µM) although their phenyl side chains are substituted with electron-withdrawing groups, *i.e*. 4-chloro and 4-bromo groups, respectively, deducing that the oversized Br atom enfeebled the BChE inhibition of **2o**. Meanwhile, compound **2m** with its phenyl side chain substituted with electron-donating methoxy (OCH_3_) group is inactive towards BChE inhibition up to a concentration of 2000 µM, which emphasizes the importance of electron-withdrawing Cl substituent in BChE inhibition. Similar to AChE inhibitory activities of compounds **2f** and **2g**, the methyl substituent at the position **R**_**2**_ of imidazo[1,2-*a*]pyridine ring gave a negative impact towards BChE inhibition. As the BChE inhibition activity of **2k** (IC_50_ = 191 µM) is two times lower than **2j** (IC_50_ = 65 µM).

Molecular docking studies of the potent imidazo[1,2-*a*]pyridine-based derivatives were performed to provide putative binding modes within the cholinesterase enzymes. The validation of the docking accuracy was carried out by docking the native co-crystallized ligand (tacrine) back into the parent enzymes and the conformation of the best-scored pose was compared with the bound ligand in the native crystal. The overall structure of human’s BChE is similar to a human’s AChE with phenylalanines (Phe) in the acyl binding pocket of AChE being replaced by leucine (Leu) and valine (Val) in BChE^24^.

**Figure 8:**
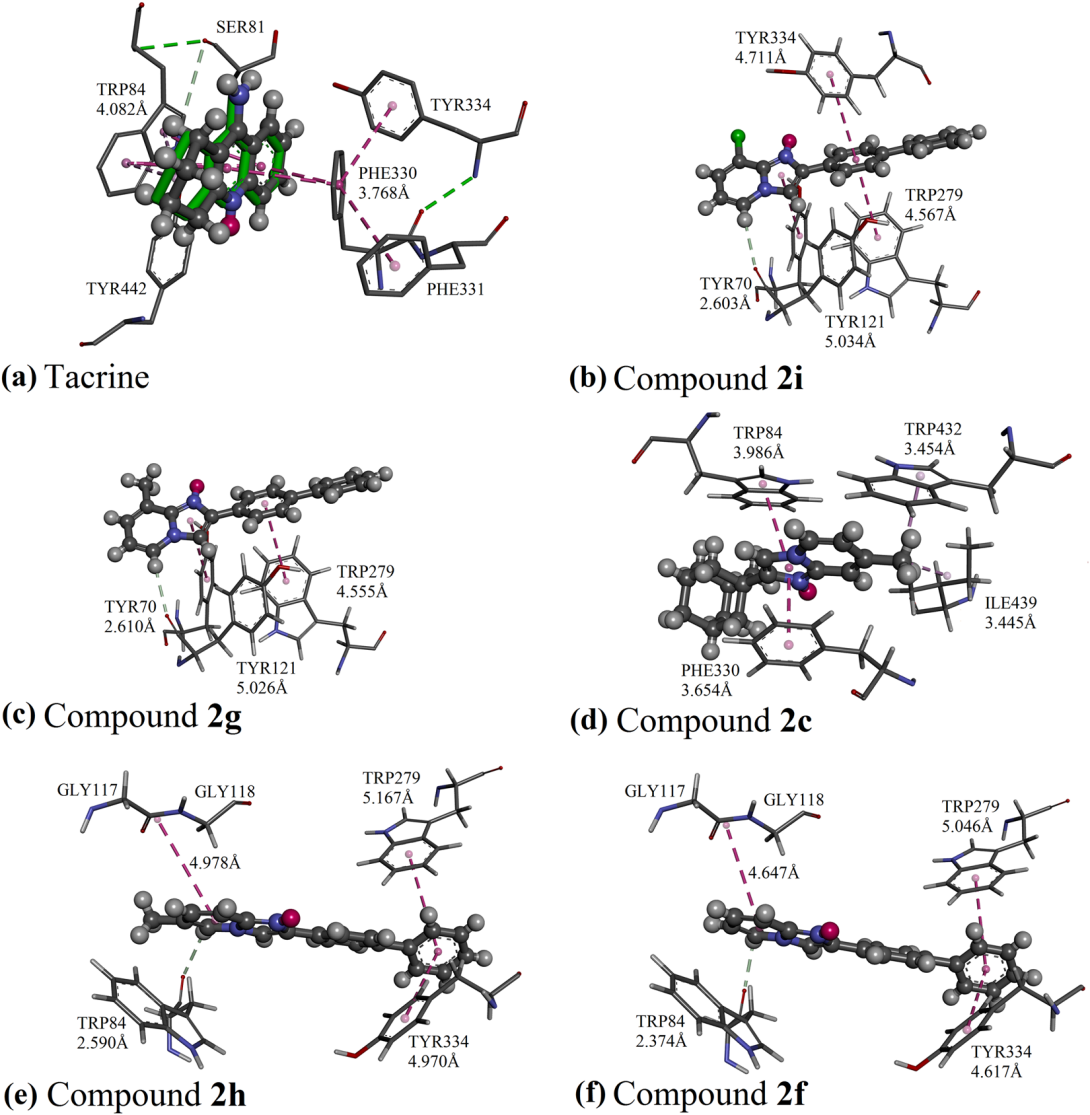
Differential validation in GOLD package by docking the native ligands of AChE into their binding sites (**a**). The native co-crystallized tacrine is represented as green sticks, while the docked ligands are shown in the form of balls and sticks, colored by elements. Putative binding modes of (**b**) compound **2i**; (**c**) compound **2g**; (**d**) compound **2c**; (**e**) compound **2h**; (**f**) compound **2f**.

In the protein crystal structure of 1ACJ, the inhibitor tacrine was bound to the catalytic active site of the AChE protein via π···π interactions, involving residues Trp84 and Phe330 (Fig. 8a). The docking models of active compounds **2i**, **2g**, **2c**, **2h** and **2f** are illustrated in Fig. 8b–f, respectively. The molecule of **2i** and **2g**, which bear substituted imidazo[1,2-*a*]pyridine ring at the position **R**_**2**_ were not penetrated to bound with any residue in the catalytic active site. Instead, the molecules of **2i** and **2g** only managed to bind only with the peripheral anionic site, which lies essentially on the surface of AChE^25^ by π···π and C-H···O interactions (involving residues Trp279, Tyr 334, Tyr112 and Tyr70 for **2i**, and residues Trp279, Tyr121 and Tyr70 for **2g**) (Fig. 8b,c) and resulted in weaker inhibitory effect. Similar to tacrine, the imidazole ring of molecule **2c** was bound to the catalytic active site through π···π interactions with residues Trp84 and Phe330. Meanwhile, its methyl group substituted at the position **R**_**3**_ was favored to bind with the residues Trp437 and Ile439 of the hydrophobic pocket *via* C-H···π and C-H···alkyl interactions (Fig. 8d). However, molecule **2c** failed to bind with any residues at the peripheral anionic site, which eventually leads to a moderate inhibition against AChE. As the most potent AChE inhibitors, the molecules of **2h** and **2f** were bound in the AChE enzyme with similar binding mode, involving both catalytic active site and peripheral anionic site. Their biphenyl side chains tend to form π···π interaction with residues Trp279 and Tyr334 at the peripheral anionic site^26^. The imidazo[1,2-*a*]pyridine rings of molecules **2h** and **2f** with unsubstituted position **R**_**2**_ were bound to the catalytic active site and oxyanion hole of the AChE enzyme through C-H···O and amide···π interactions, involving residues Trp84, Gly117 and Gly118 (Fig. 8e,f) and results in a stronger AChE inhibitory effect as compared to aforementioned compounds **2i**, **2g** and **2c**.

**Figure 9:**
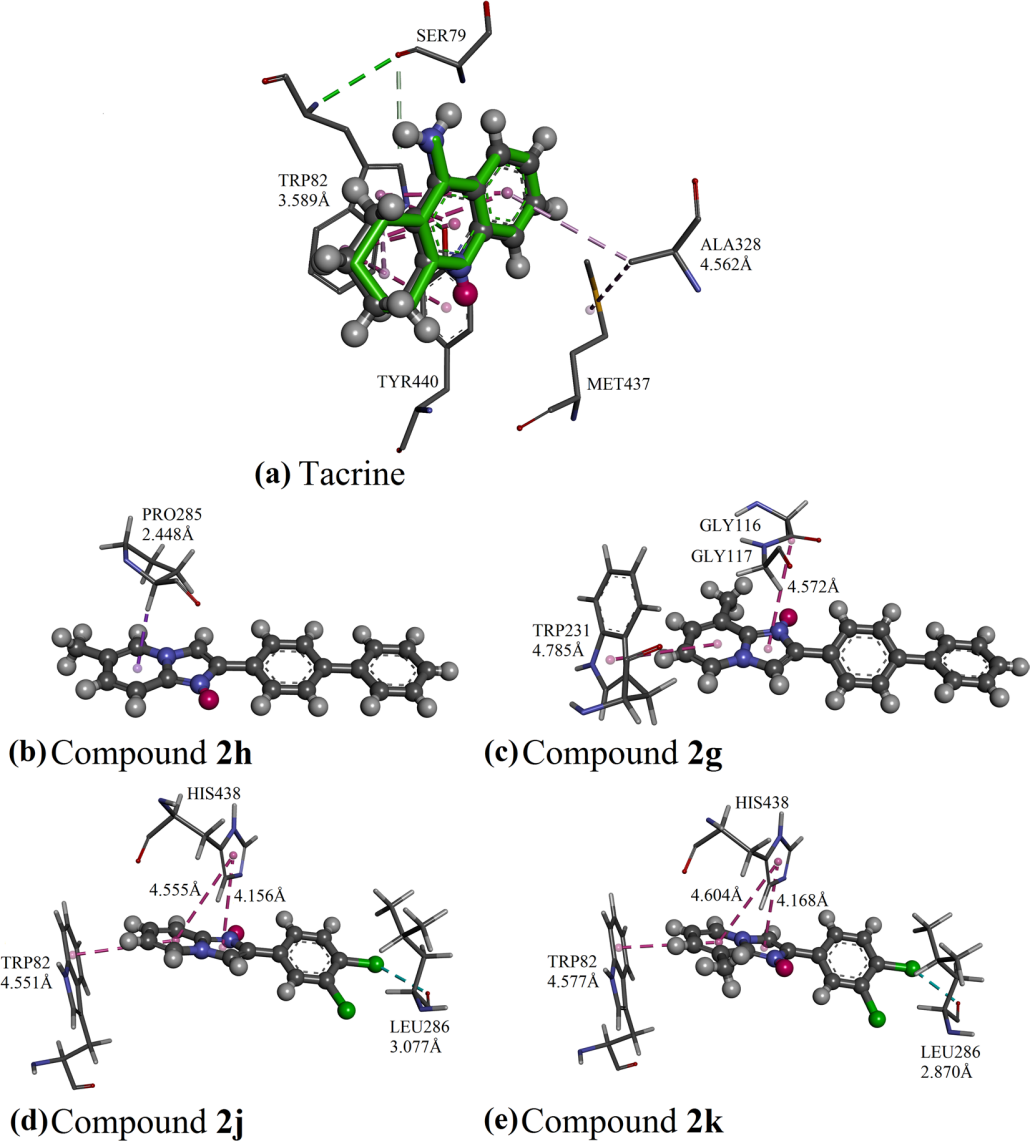
Differential validation in GOLD package by docking the native ligands of BChE into their binding sites (**a**). The native co-crystallized tacrine is represented as green sticks, while the docked ligands are shown in the form of balls and sticks, colored by elements. Putative binding modes of (**b**) compound **2h**; (**c**) compound **2g**; (**d**) compound **2j**; (**e**) compound **2k**.

In the BChE molecular docking study with the protein crystal structure 4BDS, tacrine was bound to the catalytic active site of BChE with residues Trp82 and Ala328 through alkyl···π and π···π interactions (Fig. 9a). For compounds **2g** and **2h** with moderate BChE inhibitory activity, their molecules were only favored to bind at the acyl pocket *via* π···π interaction, involving residues Trp211 and Pro285 for **2g** and **2h**, respectively (Fig. 9b,c). Compound **2g** (IC_50_ = 315 µM) which gives a better BChE inhibition than **2h** (IC_50_ = 496 µM), could form an extra amide···π interaction at the oxyanion hole with residues Gly116 and Gly117. Similar to the most potent AChE inhibitors (**2h** and **2f**), the imidazo[1,2-*a*]pyridine moieties of the most potent BChE inhibitors, **2j** (IC_50_ = 65 µM) and **2k** (IC_50_ = 191 µM) were able to bind at the catalytic active side through π···π interaction. The imidazo[1,2-*a*]pyridine rings of molecules **2j** and **2k** (Fig. 9d,e) were bound to two residues of the catalytic active site, *i.e*. Trp82 and His438. The dichloro-substituted phenyl side chains of **2j** and **2k** tend to bind tightly to the acyl pocket of the BChE enzyme, similar to a previously reported active compound, 2-(adamantan-1-yl)-2-oxoethyl 2,4-dichlorobenzoate^27^. The halogen···O interaction between the dichloro-substituted phenyl side chain and residue Leu286 of acyl pocket led to stronger BChE inhibitory effects of compounds **2j** and **2k** as compared to **2g** and **2h**.

As a summary, the molecular docking results showed that imidazo[1,2-*a*]pyridine ring was able to bind in the catalytic active site *via* π···π and C-H···O interactions (Trp84 in AChE; Trp82 and His438 in BChE). The finding reveals that AChE inhibitory effects are attributed to the biphenyl ring, which enable the imidazo[1,2-*a*]pyridine-based derivatives to bind in both catalytic active site and peripheral anionic site through π···π and C-H···O interactions. On the other hand, BChE inhibitory effects are mainly contributed by halogen···O interaction between the dichloro-substituted phenyl side chain and the residue of acyl pocket of BChE.

## Material and Methods

The reagents and solvents were obtained commercially from Sigma Aldrich and Merck Corporation, and were used without additional purification. Melting points were determined on Stuart (UK) SMP10 apparatus. ^1^H and ^13^C nuclear magnetic resonance (NMR) spectra were determined in CDCl_3_ at 500 MHz and 125 MHz, respectively, using Bruker Avance III 500 spectrometer. Fourier transform infrared spectroscopy (FTIR) spectra were recorded on Perkin Elmer Frontier FTIR spectrometer equipped with attenuated total reflection (ATR). Mass spectra were recorded using Agilent 5975C TAD Gas Chromatograph/Mass Selective Detector System with HP-5MS GC column.

The X-ray analysis of all target compounds excluding **2d** were performed using Bruker APEX II DUO CCD diffractometer employing Mo Kα radiation (λ = 0.71073 Å) with φ and ω scans. Data reduction and absorption correction were performed using SAINT and SADABS programs^28^. All structures were solved by direct methods and refined by full-matrix least-squares techniques on *F*^2^ using SHELXTL software package^29^. All non-hydrogen atoms were refined anisotropically (except for the minor disordered component of **2k** with site-occupancy less than 0.2) and all C-bound H atoms were calculated geometrically with isotropic displacement parameters set to 1.2 (or 1.5 for methyl group) times the equivalent isotropic displacement parameters of the parent carbon atoms. Crystallographic data are collected in Supplementary Information.

### Synthesis

**Figure 10:**
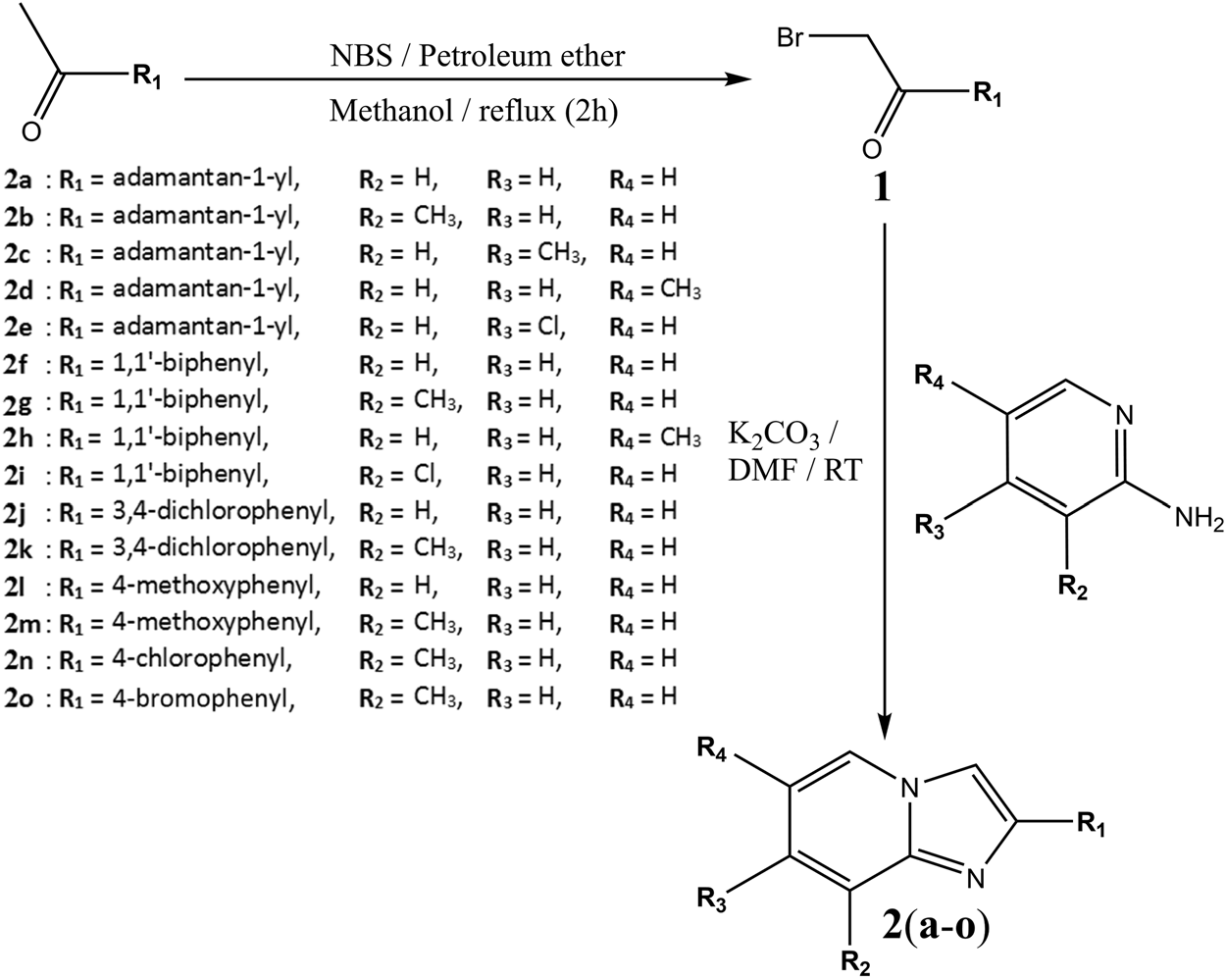
Reaction scheme for the synthesis of imidazo[1,2-*a*]pyridine derivatives **2**(**a-o**).

Target compounds were synthesized *via* a two-step reaction (Fig. 10). First, various substituted-ethanones were refluxed with *N*-bromosuccinimide (NBS) and petroleum ether in methanol at 333 K for two to five hours. Progress of the reactions were monitored by thin layer chromatography (TLC) plate using silica gel, with acetone:benzene (1:1) solvent system. The substituted-2-bromoethan-1-one (**1**) precipitate was filtered and recrystallized with ethanol. After that, **1** (0.002 mol) was reacted with 2-aminopyridines (0.003 mol) with the presence of potassium carbonate in DMF (8 ml) and stirred at room temperature for about three hours. The reaction progress was monitored by TLC method. After the reaction completed, the reaction mixture was poured into crushed ice. The precipitate formed, **2**(**a**–**o**) were filtered out and recrystallized using acetone after dried^30^. All target compounds were synthesized in good yield and high purity, their chemical structures were characterized by using FTIR, NMR, mass spectroscopy and single-crystal X-ray diffraction analysis.

#### *2-(adamantan-1-yl)imidazo[1,2-a]pyridine* (**2a**)

Solvent for growing crystal: acetone; Yield 82%; M.P. 457–459 K; FT-IR (ATR (solid) cm^−1^): 3103 (Ar, C-H, *v*), 2901, 2845 (C-H, *ν*), 1634, 1449 (Ar, C-C, *ν*), 1352, 1274 (C-N, *ν*), 728 (Ar, C-H, *ω*); ^1^H NMR (500 MHz, CDCl_3_): *δ* ppm 8.072–8.058 (d, 1 H, *J* = 7.0 Hz, ^2^CH), 7.680–7.591 (d, 1 H, *J* = 7.0 Hz, ^5^CH), 7.316 (s, 1 H, ^1^CH), 7.133–7.101 (t, 1 H, *J* = 7.0 Hz, ^4^CH), 6.735–6.708 (t, 1 H, *J* = 7.0 Hz, ^3^CH), 2.117 (bs, 3 H, ^10^CH, ^12^CH, ^14^CH), 2.070–2.064 (bd, 6 H, ^9^CH_2_, ^15^CH_2_, ^16^CH_2_), 1.836–1.824 (bt, 6 H, ^11^CH_2_, ^13^CH_2_, ^17^CH_2_); ^13^C NMR (125 MHz, CDCl_3_): *δ* ppm 157.34 (C^6^), 144.87 (C^7^), 125.46 (C^2^), 123.76 (C^4^), 117.25 (C^3^), 111.62 (C^5^), 106.59 (C^1^), 42.44 (C^9^, C^15^, C^16^), 36.93 (C^11^, C^13^, C^17^), 34.16 (C^8^), 28.64 (C^10^, C^12^, C^14^); GC-MS (EI) *m*/*z*: 252 (+M).

#### *2-(adamantan-1-yl)-8-methylimidazo[1,2-a]pyridine hydrate* (**2b**)

Solvent for growing crystal: acetone: methanol (1:1 v/v); Yield 78%; M.P. 363–365 K; FT-IR (ATR (solid) cm^−1^): 3326 (O-H, *v*), 3095 (Ar, C-H, *v*), 2899, 2847 (C-H, *ν*), 1632, 1492 (Ar, C-C, *ν*), 1355, 1271 (C-N, *ν*), 749 (C-H, *ω*); ^1^H NMR (500 MHz, CDCl_3_): *δ* ppm 7.947-7.934 (d, 1 H, *J* = 6.8 Hz, ^2^CH), 7.306 (s, 1 H, ^1^CH), 6.922-6.908 (d, 1 H, *J* = 6.8 Hz, ^4^CH), 6.655-6.628 (t, 1 H, *J* = 6.8 Hz, ^3^CH), 2.643 (s, 3 H, ^15^CH_3_), 2.117 (bs, 3 H, ^10^CH, ^12^CH, ^12A^CH), 2.086-2.081 (bd, 6 H, ^9^CH_2_, ^13^CH_2_, ^13A^CH_2_), 1.863-1.804 (bq, 6 H, ^11^CH_2_, ^11A^CH_2_, ^14^CH_2_); ^13^C NMR (125 MHz, CDCl_3_): *δ* ppm 156.53 (C^6^), 144.17 (C^7^), 127.07 (C^4^), 123.34 (C^2^), 122.85 (C^5^), 111.70 (C^5^), 107.12 (C^1^), 42.40 (C^9^, C^13^, C^13A^), 36.92 (C^11^, C^11A^, C^14^), 34.18 (C^8^), 28.65 (C^10^, C^12^, C^12A^), 17.49 (C^15^); GC-MS (EI) *m*/*z*: 266 (+M).

#### *2-(adamantan-1-yl)-7-methylimidazo[1,2-a]pyridine hydrate* (**2c**)

Solvent for growing crystal: acetone: methanol (1:1 v/v); Yield 79%; M.P. 409-411 K; FT-IR (ATR (solid) cm^−1^): 3388 (O-H, *v*), 3143 (Ar, C-H, *ν*), 2899, 2845 (C-H, *ν*), 1645, 1449 (Ar, C-C, *ν*), 1357, 1234 (C-N, *ν*), 779 (C-H, *ω*); ^1^H NMR (500 MHz, CDCl_3_): *δ* ppm 7.944-7.930 (d, 1 H, *J* = 6.9 Hz, ^2^CH), 7.353 (s, 1 H, ^1^CH), 7.223 (s, 1 H, ^5^CH), 6.566-6.552 (d, 1 H, *J* = 6.9 Hz, ^3^CH), 2.381 (s, 3 H, ^18^CH_3_), 2.108 (bs, 3 H, ^10^CH, ^12^CH, ^14^CH), 2.052-2.046 (bd, 6 H, ^9^CH_2_, ^15^CH_2_, ^16^CH_2_), 1.828-1.816 (bt, 6 H, ^11^CH_2_, ^13^CH_2_, ^17^CH_2_); ^13^C NMR (125 MHz, CDCl_3_): *δ* ppm 157.06 (C^6^), 145.37 (C^7^), 134.49 (C^4^), 124.70 (C^2^), 115.67 (C^5^), 114.21 (C^3^), 105.90 (C^1^), 42.44 (C^9^, C^15^, C^16^), 36.96 (C^11^, C^13^, C^17^), 34.09 (C^8^), 28.64 (C^10^, C^12^, C^14^), 21.30 (C^18^); GC-MS (EI) *m*/*z*: 266 (+M).

#### *2-(adamantan-1-yl)-6-methylimidazo[1,2-a]pyridine* (**2d**)

Yield 70%; M.P. 371-373 K; FT-IR (ATR (solid) cm^−1^): 3106 (Ar, C-H, *v*), 2896, 2847 (C-H, *ν*), 1632, 1446 (Ar, C-C, *ν*), 1355, 1274 (C-N, *ν*), 730 (C-H, *ω*); ^1^H NMR (500 MHz, CDCl_3_): *δ* ppm 7.754 (s, 1 H, ^2^CH), 7.412-7.394 (d, 1 H, *J* = 9.2 Hz, ^5^CH), 7.134 (s, 1 H, ^1^CH), 6.886-6.867 (d, 1 H, *J* = 9.2 Hz, ^4^CH), 2.211 (s, 3 H, ^18^CH_3_), 2.116 (bs, 3 H, ^10^CH, ^12^CH, ^14^CH), 1.959-1.953 (bd, 6 H, ^9^CH_2_, ^15^CH_2_, ^16^CH_2_), 1.733-1.721 (bt, 6 H, ^11^CH_2_, ^13^CH_2_, ^17^CH_2_); ^13^C NMR (125 MHz, CDCl_3_): *δ* ppm 157.00 (C^6^), 143.89 (C^7^), 126.96 (C^4^), 123.24 (C^2^), 121.15 (C^3^), 116.50 (C^5^), 106.32 (C^1^), 42.44 (C^9^, C^15^, C^16^), 36.93 (C^11^, C^13^, C^17^), 34.11 (C^8^), 28.63 (C^10^, C^12^, C^14^), 18.06 (C^18^); GC-MS (EI) *m*/*z*: 266 (+M).

#### *2-(adamantan-1-yl)-7-chloroimidazo[1,2-a]pyridine hydrate* (**2e**)

Solvent for growing crystal: acetone; Yield 85%; M.P. 395-397 K; FT-IR (ATR (solid) cm^−1^): 3299 (O-H, *v*), 3149 (Ar, C-H, *ν*), 2901, 2850 (C-H, *ν*), 1626, 1451 (Ar, C-C, *ν*), 1352, 1234 (C-N, *ν*), 790 (C-Cl, *v*), 774 (C-H, *ω*); ^1^H NMR (500 MHz, CDCl_3_): *δ* ppm 7.995-7.981 (d, 1 H, *J* = 7.2 Hz, ^2^CH), 7.603 (s, 1 H, ^5^CH), 7.293 (s, 1 H, ^1^CH), 6.743-6.729 (d, 1 H, *J* = 7.2 Hz, ^3^CH), 2.116 (bs, 3 H, ^10^CH, ^12^CH, ^12A^CH), 2.042-2.036 (bd, 6 H, ^9^CH_2_, ^13^CH_2_, ^13A^CH_2_), 1.831-1.818 (bt, 6 H, ^11^CH_2_, ^11A^CH_2_, ^14^CH_2_); ^13^C NMR (125 MHz, CDCl_3_): *δ* ppm 158.20 (C^6^), 142.25 (C^7^), 135.56 (C^4^), 125.59 (C^2^), 116.19 (C^5^), 113.35 (C^3^), 106.94 (C^1^), 42.29 (C^9^, C^13^, C^13A^), 36.88 (C^11^, C^11A^, C^14^), 34.32 (C^8^), 28.76 (C^10^, C^12^, C^12A^); GC-MS (EI) *m*/*z*: 286 (+M).

#### *2-([1,1′-biphenyl]-4-yl)imidazo[1,2-a]pyridine* (**2f**)

Solvent for growing crystal: acetone: methanol (1:1 v/v); Yield 77%; M.P. 472-474 K; FT-IR (ATR (solid) cm^−1^): 3129, 3030 (Ar, C-H, *ν*), 1634, 1476 (Ar, C-C, *ν*), 1373, 1252 (C-N, *ν*), 739 (Ar, C-H, *ω*); ^1^H NMR (500 MHz, CDCl_3_): *δ* ppm 8.278-8.261 (d, 1 H, *J* = 8.6 Hz, ^2^CH), 8.163-8.146 (d, 2 H, *J* = 8.4 Hz, ^9^CH, ^13^CH), 8.037 (s, 1 H, ^1^CH), 7.774-7.757 (d, 1 H, *J* = 8.6 Hz, ^5^CH), 7.751-7.734 (d, 2 H, *J* = 8.4 Hz, ^10^CH, ^12^CH), 7.706-7.689 (d, 1 H, *J* = 8.0 Hz, ^17^CH), 7.649-7.633 (d, 2 H, *J* = 8.0 Hz, ^15^CH, ^19^CH), 7.513-7.483 (t, 2 H, *J* = 8.0 Hz, ^16^CH, ^18^CH), 7.434-7.405 (t, 1 H, *J* = 8.6, ^4^CH), 7.230-7.203 (t, 1 H, *J* = 8.6 Hz, ^3^CH); ^13^C NMR (125 MHz, CDCl_3_): *δ* ppm 145.90 (C^6^), 144.48 (C^14^), 140.65 (C^7^), 139.80 (C^11^), 138.03 (C^8^), 128.99 (C^16^, C^18^), 128.84 (C^4^), 127.98 (C^9^, C^13^), 127.88 (C^17^), 127.15 (C^15^, C^19^), 127.03 (C^10^, C^12^), 126.57 (C^2^), 123.03 (C^5^), 113.31 (C^3^), 108.51 (C^1^); GC-MS (EI) *m*/*z*: 270 (+M).

#### *2-([1,1′-biphenyl]-4-yl)-8-methylimidazo[1,2-a]pyridine* (**2g**)

Solvent for growing crystal: acetone; Yield 85%; M.P. 411-413 K; FT-IR (ATR (solid) cm^−1^): 3037 (Ar, C-H, *v*), 2928 (C-H, *ν*), 1629, 1478 (Ar, C-C, *ν*), 1372, 1258 (C-N, *ν*), 736 (Ar, C-H, *ω*); ^1^H NMR (500 MHz, CDCl_3_): *δ* ppm 8.085-8.068 (d, 2 H, *J* = 8.5 Hz, ^9^CH, ^13^CH), 8.021-8.012 (d, 1 H, *J* = 6.8 Hz, ^2^CH), 7.898 (s, 1 H, ^1^CH), 7.714-7.697 (d, 2 H, *J* = 8.5 Hz, ^10^CH, ^12^CH), 7.691-7.667 (d, 2 H, *J* = 7.2 Hz, ^15^CH, ^19^CH), 7.498-7.467 (t, 2 H, *J* = 7.2 Hz, ^16^CH, ^18^CH), 7.396-7.367 (t, 1 H, *J* = 7.2 Hz, ^17^CH), 7.000-6.987 (d, 1 H, *J* = 6.8 Hz, ^4^CH), 6.731-7.714 (t, 1 H, *J* = 6.8 Hz, ^3^CH), 2.716 (s, 1 H, ^20^CH_3_); ^13^C NMR (125 MHz, CDCl_3_): *δ* ppm 146.20 (C^6^), 144.70 (C^14^), 140.86 (C^7^), 140.50 (C^11^), 132.95(C^8^), 128.78 (C^16^, C^18^), 127.75 (C^5^), 127.36 (C^9^, C^13^), 127.28 (C^17^), 126.99 (C^15^, C^19^), 126.58 (C^10^, C^12^), 123.52 (C^2^), 123.44 (C^4^), 112.50 (C^3^), 108.70 (C^1^) 17.16 (C^20^); GC-MS (EI) *m*/*z*: 284 (+M).

#### *2-([1,1′-biphenyl]-4-yl)-6-methylimidazo[1,2-a]pyridine* (**2h**)

Solvent for growing crystal: acetone; Yield 88%; M.P. 508-510 K; FT-IR (ATR (solid) cm^−1^): 3130, 3038 (Ar, C-H, *ν*), 2932 (C-H, *ν*), 1597, 1478 (Ar, C-C, *ν*), 1349, 1258 (C-N, *ν*), 733 (C-H, *ω*); ^1^H NMR (500 MHz, CDCl_3_): *δ* ppm 8.105-8.080 (d, 2 H, *J* = 8.5 Hz, ^9^CH, ^13^CH), 8.068 (s, 1 H, ^1^CH), 8.010-7.992 (d, 1 H, *J* = 9.1 Hz, ^5^CH), 7.921 (s, 1 H, ^2^CH), 7.720-7.703 (d, 2 H, *J* = 8.5 Hz, ^10^CH, ^12^CH), 7.657-7.642 (d, 2 H, *J* = 7.2 Hz, ^15^CH, ^19^CH), 7.508-7.478 (t, 2 H, *J* = 7.2 Hz, ^16^CH, ^18^CH), 7.421-7.392 (d, 1 H, *J* = 7.2 Hz, ^17^CH), 7.332-7.314 (d, 1 H, *J* = 9.1 Hz, ^4^CH), 2.394 (s, 3 H, ^20^CH3); ^13^C NMR (125 MHz, CDCl_3_): *δ* ppm 145.49 (C^6^), 142.22 (C^14^), 140.10 (C^7^), 137.48 (C^11^), 136.99 (C^8^), 128.93 (C^16^, C^18^), 128.81 (C^4^), 127.80 (C^3^), 127.69 (C^9^, C^13^), 127.22 (C^17^), 126.98 (C^15^, C^19^), 126.83 (C^10^, C^12^), 126.04 (C^2^), 124.07 (C^5^), 108.14 (C^1^), 18.23 (C^20^); GC-MS (EI) *m*/*z*: 284 (+M).

#### *2-([1,1′-biphenyl]-4-yl)-8-chloroimidazo[1,2-a]pyridine* (**2i**)

Solvent for growing crystal: acetone: methanol: ethanol (1:1:1 v/v/v); Yield 70%; M.P. 491-493 K; FT-IR (ATR (solid) cm^−1^): 3135, 3063 (Ar, C-H, *v*), 1626, 1476 (Ar, C-C, *ν*), 1368, 1258 (C-N, *ν*), 784 (C-Cl, *ν*), 736 (C-H, *ω*); ^1^H NMR (500 MHz, CDCl_3_): *δ* ppm 8.316-8.299 (d, 2 H, *J* = 8.4 Hz, ^9^CH, ^13^CH), 8.203-8.191 (d, 1 H, *J* = 7.0 Hz, ^2^CH), 8.178 (s, 1 H, ^1^CH), 7.787-7.770 (d, 2 H, *J* = 8.4 Hz, ^10^CH, ^12^CH), 7.761-7.744 (d, 1 H, *J* = 7.0 Hz, ^4^CH), 7.703-7.687 (d, 2 H, *J* = 8.0 Hz, ^15^CH, ^19^CH), 7.514-7.484 (t, 2 H, *J* = 8.0 Hz, ^16^CH, ^18^CH), 7.420-7.391 (t, 2 H, *J* = 8.0 Hz, ^17^CH), 7.061-7.032 (t, 1 H, *J* = 7.0 Hz, ^3^CH); ^13^C NMR (125 MHz, CDCl_3_): *δ* ppm 146.25 (C^6^), 144.82 (C^14^), 140.89 (C^7^), 140.50 (C^11^), 132.99(C^8^), 130.53 (C^5^), 128.79 (C^16^, C^18^), 127.54 (C^4^), 127.36 (C^9^, C^13^), 127.28 (C^17^), 126.98 (C^15^, C^19^), 126.58 (C^10^, C^12^), 123.52 (C^3^), 112.50 (C^2^), 108.70 (C^1^); GC-MS (EI) *m*/*z*: 304 (+M).

#### *2-(3,4-dichlorophenyl)imidazo[1,2-a]pyridine* (**2j**)

Solvent for growing crystal: acetone: methanol (1:1 v/v); Yield 81%; M.P. 439-441 K; FT-IR (ATR (solid) cm^−1^): 3122, 3033 (Ar, C-H, *ν*), 1637, 1451 (Ar, C-C, *ν*), 1371, 1252 (C-N, *ν*), 825 (C-Cl, *ν*), 744 (C-H, *ω*); ^1^H NMR (500 MHz, CDCl_3_): *δ* ppm 8.145-8.135 (d, 1 H, *J* = 7.0 Hz, ^2^CH), 8.088 (s, 1 H, ^9^CH), 7.874 (s, 1 H, ^1^CH), 7.798-7.777 (d, 1 H, *J* = 9.0 Hz, ^13^CH), 7.655-7.637 (d, 1 H, *J* = 9.0 Hz, ^12^CH), 7.519-7.503 (d, 1 H, *J* = 7.0 Hz, ^5^CH), 7.245-7.213 (t, 1 H, *J* = 7.0 Hz, ^4^CH) 6.848-6.821 (t, 1 H, *J* = 7.0 Hz, ^3^CH); ^13^C NMR (125 MHz, CDCl_3_): *δ* ppm 145.79 (C^6^), 143.50 (C^7^), 133.91 (C^10^), 131.68 (C^11^), 130.68 (C^12^), 127.80 (C^9^), 125.70 (C^13^), 125.27 (C^2^), 125.14 (C^4^), 117.66 (C^5^), 112.87 (C^3^), 108.59 (C^1^); GC-MS (EI) *m*/*z*: 262 (+M).

#### *2-(3,4-dichlorophenyl)-8-methylimidazo[1,2-a]pyridine* (**2k**)

Solvent for growing crystal: acetone: methanol (1:1 v/v); Yield 80%; M.P. 410-412 K; FT-IR (ATR (solid) cm^−1^): 3114, 3076 (Ar, C-H, *ν*), 2923 (C-H, *ν*), 1632, 1457 (Ar, C-C, *ν*), 1372, 1136 (C-N, *ν*), 784 (C-Cl, *ν*), 741 (C-H, *ω*); ^1^H NMR (500 MHz, CDCl_3_): *δ* ppm 8.105 (s, 1 H, ^1^CH), 8.020-8.007 (d, 1 H, *J* = 6.8 Hz, ^2^CH), 7.857 (s, 1 H, ^13^CH), 7.825-7.804 (d, 1 H, *J* = 8.4 Hz, ^9^CH), 7.516-7.499 (d, 1 H, *J* = 8.4 Hz, ^10^CH), 7.021-7.007 (d, 1 H, *J* = 6.8 Hz, ^4^CH), 6.758-6.730 (t, 1 H, *J* = 6.8 Hz, ^3^CH), 2.678 (s, 3 H, ^14^CH_3_); ^13^C NMR (125 MHz, CDCl_3_): *δ* ppm 146.28 (C^6^), 142.82 (C^7^), 134.15 (C^8^), 132.84 (C^11^), 131.48 (C^12^), 130.62 (C^9^, C^13^), 127.89 (C^5^), 125.28 (C^10^), 123.94 (C^2^), 123.51 (C^4^), 112.87 (C^3^), 109.07 (C^1^), 17.07 (C^14^); GC-MS (EI) *m*/*z*: 276 (+M).

#### *2-(4-methoxyphenyl)imidazo[1,2-a]pyridine* (**2l**)

Solvent for growing crystal: acetone; Yield 85%; M.P. 402-404 K; FT-IR (ATR (solid) cm^−1^): 3135, 3006 (Ar, C-H, *ν*), 2966 (C-H, *ν*), 1634, 1481 (Ar, C-C, *ν*), 1371, 1172 (C-N, *ν*), 1239, 1029 (C-O, *ν*), 744 (C-H, *ω*); ^1^H NMR (500 MHz, CDCl_3_): *δ* ppm 8.133-8.119 (d, 1 H, *J* = 7.0 Hz, ^2^CH), 7.921-7.904 (d, 2 H, *J* = 8.9 Hz, ^9^CH, ^13^CH), 7.801 (s, 1 H, ^1^CH), 7.651-7.632 (d, 1 H, *J* = 7.0 Hz, ^5^CH), 7.195-7.163 (t, 1 H, *J* = 7.0 Hz, ^4^CH), 7.009-6.992 (d, 2 H, *J* = 8.9 Hz, ^10^CH, ^12^CH) 6.800-6.773 (t, 1 H, *J* = 7.0 Hz, ^3^CH), 3.879 (s, 3 H, ^14^CH_3_); ^13^C NMR (125 MHz, CDCl_3_): *δ* ppm 159.61 (C^11^), 145.73 (C^6^), 145.62 (C^7^), 127.32 (C^9^, C^13^), 126.44 (C^8^), 125.46 (C^2^), 124.50 (C^4^), 117.31 (C^5^), 114.16 (C^10^, C^12^), 112.30 (C^3^), 107.23 (C^5^), 55.33 (C^14^); GC-MS (EI) *m*/*z*: 224 (+M).

#### *2-(4-methoxyphenyl)-8-methylimidazo[1,2-a]pyridine* (**2m**)

Solvent for growing crystal: acetone; Yield 88%; M.P. 391-393 K; FT-IR (ATR (solid) cm^−1^): 3129, 3010 (Ar, C-H, *ν*), 2960, 2838 (C-H, *ν*), 1611, 1486 (Ar, C-C, *ν*), 1370, 1173 (C-N, *ν*), 1248, 1030 (C-O, *ν*), 776 (C-H, *ω*); ^1^H NMR (500 MHz, CDCl_3_): *δ* ppm 8.013-7.999 (d, 1 H, *J* = 6.8 Hz, ^2^CH), 7.938-7.921 (d, 2 H, *J* = 8.9 Hz, ^9^CH, ^13^CH), 7.782 (s, 1 H, ^1^CH), 7.003-6.973 (m, 3 H, ^4^CH, ^10^CH, ^12^CH), 6.723-6.696 (t, 1 H, *J* = 6.8 Hz, ^3^CH), 3.877 (s, 3 H, ^15^CH_3_) 2.668 (s, 3 H, ^14^CH_3_); ^13^C NMR (125 MHz, CDCl_3_): *δ* ppm 159.54 (C^11^), 145.89 (C^6^), 144.89 (C^7^), 132.33 (C^8^), 127.51 (C^9^, C^13^), 126.47 (C^5^), 123.54 (C^2^), 123.36 (C^4^), 114.11 (C^10^, C^12^), 112.42 (C^3^), 107.77 (C^1^), 55.33 (C^15^) 17.18 (C^14^); GC-MS (EI) *m*/*z*: 238 (+M).

#### *2-(4-chlorophenyl)-8-methylimidazo[1,2-a]pyridine* (**2n**)

Solvent for growing crystal: acetone: methanol (1:1 v/v); Yield 82%; M.P. 417–419 K; FT-IR (ATR (solid) cm^−1^): 3034 (Ar, C-H, *ν*), 2923 (C-H, *ν*), 1629, 1473 (Ar, C-C, *ν*), 1367, 1256 (C-N, *ν*), 845 (C-Cl, *ν*), 736 (C-H, *ω*); ^1^H NMR (500 MHz, CDCl_3_): *δ* ppm 8.002-7.988 (d, 1 H, *J* = 6.8 Hz, ^2^CH), 7.935-7.918 (d, 2 H, *J* = 8.6 Hz, ^9^CH, ^13^CH), 7.830 (s, 1 H, ^1^CH), 7.424-7.407 (d, 2 H, *J* = 8.6 Hz, ^10^CH, ^12^CH), 6.993-6.980 (d, 1 H, *J* = 6.8 Hz, ^4^CH), 6.727-6.699 (t, 1 H, *J* = 6.8 Hz, ^3^CH), 2.677 (s, 3 H, ^14^CH_3_); ^13^C NMR (125 MHz, CDCl_3_): *δ* ppm 146.24 (C^6^), 144.04 (C^7^), 133.48 (C^8^), 132.57 (C^11^), 128.84 (C^9^, C^13^), 127.61 (C^5^), 127.39 (C^10^, C^12^), 123.62 (C^2^), 123.45 (C^4^), 112.60 (C^3^), 108.67 (C^1^), 17.10 (C^14^); GC-MS (EI) *m*/*z*: 242 (+M).

#### *2-(4-bromophenyl)-8-methylimidazo[1,2-a]pyridine* (**2o**)

Solvent for growing crystal: acetone; Yield 85%; M.P. 405–407 K; FT-IR (ATR (solid) cm^−1^): 3135, 3037 (Ar, C-H, *ν*), 2920 (C-H, *ν*), 1629, 1468 (Ar, C-C, *ν*), 1370, 1253 (C-N, *ν*), 826 (C-Cl, *ν*), 733 (C-H, *ω*); ^1^H NMR (500 MHz, CDCl_3_): *δ* ppm 7.973-7.960 (d, 1 H, *J* = 6.8 Hz, ^2^CH), 7.861-7.844 (d, 2 H, *J* = 8.6 Hz, ^9^CH, ^13^CH), 7.810 (s, 1 H, ^1^CH), 7.566-7.549 (d, 2 H, *J* = 8.6 Hz, ^10^CH, ^12^CH), 6.980-6.966 (d, 1 H, *J* = 6.8 Hz, ^4^CH), 6.709-6.682 (t, 1 H, *J* = 6.8 Hz, ^3^CH), 2.668 (s, 3 H, ^14^CH_3_); ^13^C NMR (125 MHz, CDCl_3_): *δ* ppm 146.27 (C^6^), 144.04 (C^7^), 133.07 (C^8^), 131.76 (C^9^, C^13^), 127.67 (C^10^, C^12^), 127.61 (C^11^), 123.60 (C^2^), 123.45 (C^4^), 121.65 (C^5^), 112.58 (C^3^), 108.71 (C^1^), 17.10 (C^14^); GC-MS (EI) *m*/*z*: 286 (+M).

### Cholinesterase inhibition assay

Ellman’s colorimetric method with minor modification was employed in the determination of AChE and BChE inhibitory activities^16^. Electric eel acetylcholinesterase (AChE, EC 3.1.1.7), equine serum butyrylcholinesterase (BChE, EC 3.1.1.8), 5,5′-Dithiobis(2-nitrobenzoic acid) (DTNB) and sodium phosphate dibasic (Na_2_HPO_4_) were purchased from Sigma-Aldrich (USA). S-butyrylthiocholine chloride (BTCC) from Sigma-Aldrich (Switzerland), acetylthiocholine iodide (ATCI) from Sigma-Aldrich (United Kingdom) and sodium phosphate monobasic (NaH_2_PO_4_) from Sigma-Aldrich (Germany) were purchased. Tacrine hydrochloride was purchased from Cayman Chemical Company and absolute ethanol from Merck Emsure® Germany was used as the solvent. Ethanol was used to replace the sample as a control in this assay^31^. Tacrine hydrochloride was used as the reference drug in the inhibition assays of AChE and BChE.

### Statistical analysis

The results of cholinesterase inhibitory assay were expressed as mean ± standard deviation (SD) and were labeled if p < 0.05 by using ANOVA of IBM SPSS Statistics for Windows, Version 23.0 (IBM, New York).

### Docking protocol

Molecular docking was performed for the most potent inhibitors using Genetic Optimization for Ligand Docking (GOLD) package 5.4.1^32–34^. Crystal structures of AChE (PDB ID: 1ACJ^35^) and BChE (PDB ID: 4BDS^36^) isolated from Teronarce californica and Homo sapiens, respectively, with tacrine were obtained from Protein Data Bank. Ligand-protein binding space and the conformational flexibility of ligand inside the protein were explored by Genetic Algorithm (GA). A spherical binding site with a radius of 15 Å was used, it lays around the binding site of tacrine covering both catalytic anionic and peripheral anionic sites. 100 GA runs were carried out and the top 100 ranked docking poses were scored using the Piecewise Linear Potential (PLP) scoring function. Default values were used for all other parameters. The intermolecular interactions of the best scored pose of each ligand were analyzed and illustrated using Discovery Studio 4.5 software (Discovery studio v4.5.0.15071. Accelrys Inc 2015).

## Conclusion

A series of imidazo[1,2-*a*]pyridine-based derivatives **2**(**a**-**o**) were synthesized and characterized by FTIR, NMR and GCMS spectroscopy analyses. The 3D structures of these derivatives were further confirmed by single crystal X-ray diffraction studies and the result showed that they tend to adapt *periplanar* conformation with its substituted side chain at the position **R**_**1**_. With the presence of imidazo[1,2-*a*]pyridine ring and the low degree-of-freedom, the introduction of adamantyl or biphenyl does not affect their structural occupancies. Meanwhile, the present compounds tend to pack in a similar pattern with π···π and C-H···π interactions, involving the imidazo[1,2-*a*]pyridine ring, these had led to redundant 2D or 3D structural similarity in imidazo[1,2-*a*]pyridine-based derivatives. The AChE inhibitory effects of imidazo[1,2-*a*]pyridine-based derivatives increased in the order of phenyl <adamantyl < biphenyl side chain substituted at the position **R**_**1**_. Those derivatives with methyl substituent at the position **R**_**2**_ of the imidazo[1,2-*a*]pyridine ring exhibited weaker AChE and BChE inhibitory effects. The molecular docking studies of the imidazo[1,2-*a*]pyridine-based derivatives revealed that the π···π or halogen···O interaction between the active compounds and their surrounding residues in the peripheral anionic site or acyl pocket of the target enzymes are dominant as compared to the hydrophobic interaction in the catalytic active site.

## Supplementary information

SUPPLEMENTARY INFO

## Data Availability

The crystallographic data for **2**(**a**, **b**, **c** and **e**-**o**) were deposited with the Cambridge Crystallographic Data Centre as supplementary publications with CCDC no. 1055984-1055988 and 1541130-1541138, respectively. Copies of available materials can be obtained free of charge on application to CCDC, 12 Union Road, Cambridge CB2 1EZ, UK, (Fax: + 44-(0)1223-336033 or e-mail: deposit@ccdc.cam.ac.uk).
