# Supplementary material for: Crystal Correlation Of Heterocyclic Imidazo[1,2-a]pyridine Analogues and Their Anticholinesterase Potential Evaluation
Source: Sci Rep. 2019 Jan 30;9:926. doi: 10.1038/s41598-018-37486-7 (PMC6354011; doi:10.1038/s41598-018-37486-7)
Supplement: Supplementary file 1 — SUPPLEMENTARY INFO [file 41598_2018_37486_MOESM1_ESM.pdf]

Supporting data

Crystal Correlation Of Heterocyclic Imidazo[1,2-a]pyridine Analogues And Their Anticholinesterase Potential Evaluation

Huey Chong Kwong[1], C. S. Chidan Kumar[2#], Siau Hui Mah[3], Yew Leng Mah[4], Tze Shyang Chia[5], Ching Kheng Quah[5*], Gin Keat Lim[1] and Siddegowda Chandraju[6]

[1]School of Chemical Sciences, Universiti Sains Malaysia, Penang 11800 USM, Malaysia
[2]Department of Engineering Chemistry, Vidya Vikas Institute of Engineering & Technology, Visvesvaraya Technological University, Alanahalli, Mysuru 570028, Karnataka, India
[3]School of Biosciences, Taylor's University, Lakeside Campus, 47500 Subang Jaya, Selangor, Malaysia
[4]Jagiellonian University Medical College, Krakow, Poland
[5]X-ray Crystallography Unit, School of Physics, Universiti Sains Malaysia, Penang 11800 USM, Malaysia
[6]Department of Chemistry, Alva's Institute of Engineering & Technology, Mijar, Moodbidri 574225, India

*Corresponding author email: ckquah@usm.my
[#]Co-corresponding author email: stonekg01@gmail.com
Tel.: +604 653 3888 Ext. 3690; Fax: +6046579150.

Contents

1.0 ^{1}H NMR, ^{13}C NMR, FTIR and GCMS spectra 3

1.1 2-(adamantan-1-yl)imidazo[1,2-a]pyridine (**2a**) 3

1.2 2-(adamantan-1-yl)-8-methylimidazo[1,2-a]pyridine (**2b**) 6

1.3 2-(adamantan-1-yl)-7-methylimidazo[1,2-a]pyridine (**2c**) 9

1.4 2-(adamantan-1-yl)-6-methylimidazo[1,2-a]pyridine (**2d**) 12

1.5 2-(adamantan-1-yl)-7-chloroimidazo[1,2-a]pyridine (**2e**) 15

1.6 2-([1,1'-biphenyl]-4-yl)imidazo[1,2-a]pyridine (**2f**) 18

1.7 2-([1,1'-biphenyl]-4-yl)-8-methylimidazo[1,2-a]pyridine (**2g**) 21

1.8 2-([1,1'-biphenyl]-4-yl)-6-methylimidazo[1,2-a]pyridine (**2h**) 24

1.9 2-([1,1'-biphenyl]-4-yl)-8-chloroimidazo[1,2-a]pyridine (**2i**) 27

1.10 2-(3,4-dichlorophenyl)imidazo[1,2-a]pyridine (**2j**) 30

1.11 2-(3,4-dichlorophenyl)-8-methylimidazo[1,2-a]pyridine (**2k**) 33

1.12 2-(4-methoxyphenyl)imidazo[1,2-a]pyridine (**2l**) 36

1.13 2-(4-methoxyphenyl)-8-methylimidazo[1,2-a]pyridine (**2m**) 39

1.14 2-(4-chlorophenyl)-8-methylimidazo[1,2-a]pyridine (**2n**) 42

1.15 2-(4-bromophenyl)-8-methylimidazo[1,2-a]pyridine (**2o**) 45

2.0 Single crystal X-ray crystallography data 48

2.1 Crystal data and parameters 48

2.3 Ortep diagram and atom numbering 50
2.4 Supramolecular feature 52
2.1 Hydrogen bonding geometries 58
3.0 Raw data for Anti-cholinesterase Assay 59
3.1 AChE inhibition 59
3.1.1 Raw data for IC_{50} calculation of compound **2b**. 59
3.1.2 Raw data for IC_{50} calculation of compound **2c**. 62
3.1.3 Raw data for IC_{50} calculation of compound **2f**. 65
3.1.4 Raw data for IC_{50} calculation of compound **2g**. 68
3.1.5 Raw data for IC_{50} calculation of compound **2h**. 71
3.1.6 Raw data for IC_{50} calculation of compound **2i**. 74
3.1.7 Summary of AChE IC_{50}. 77
3.1.8 Statistical analysis for the IC_{50} of the test compound (AChE). 78
3.2 BChE inhibition 79
3.2.1 Raw data for IC_{50} calculation of compound **2b**. 79
3.2.2 Raw data for IC_{50} calculation of compound **2g**. 82
3.2.3 Raw data for IC_{50} calculation of compound **2h**. 85
3.2.4 Raw data for IC_{50} calculation of compound **2j**. 88
3.2.5 Raw data for IC_{50} calculation of compound **2k**. 91
3.2.6 Raw data for IC_{50} calculation of compound **2n**. 94
3.2.7 Summary of BChE IC_{50}. 97
3.2.8 Statistical analysis for the IC_{50} of the test compound (BChE). 98

1.0 ^{1}H NMR, ^{13}C NMR, FTIR and GCMS spectra

1.1 2-(adamantan-1-yl)imidazo[1,2-a]pyridine (**2a**)

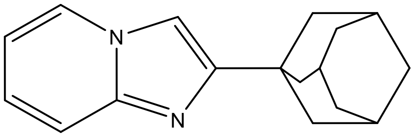

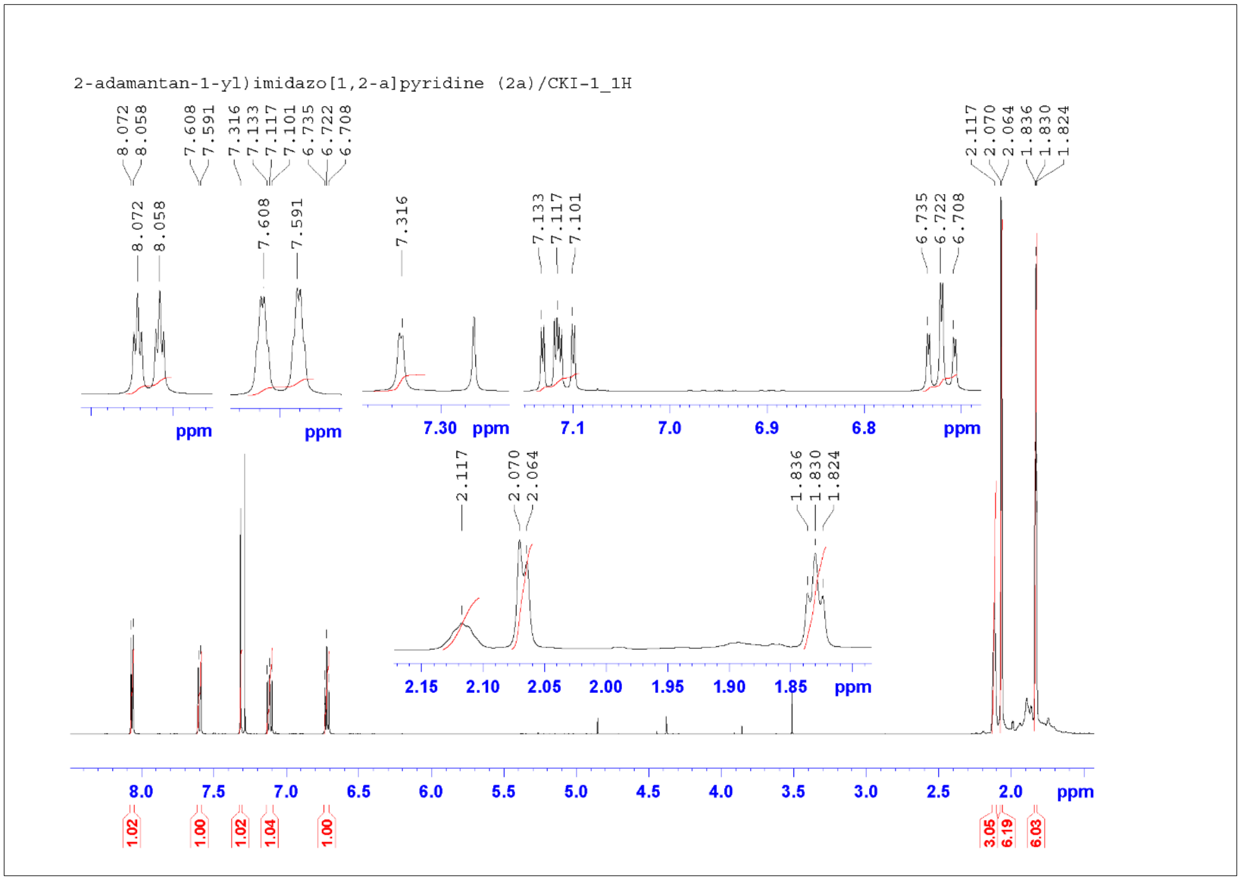

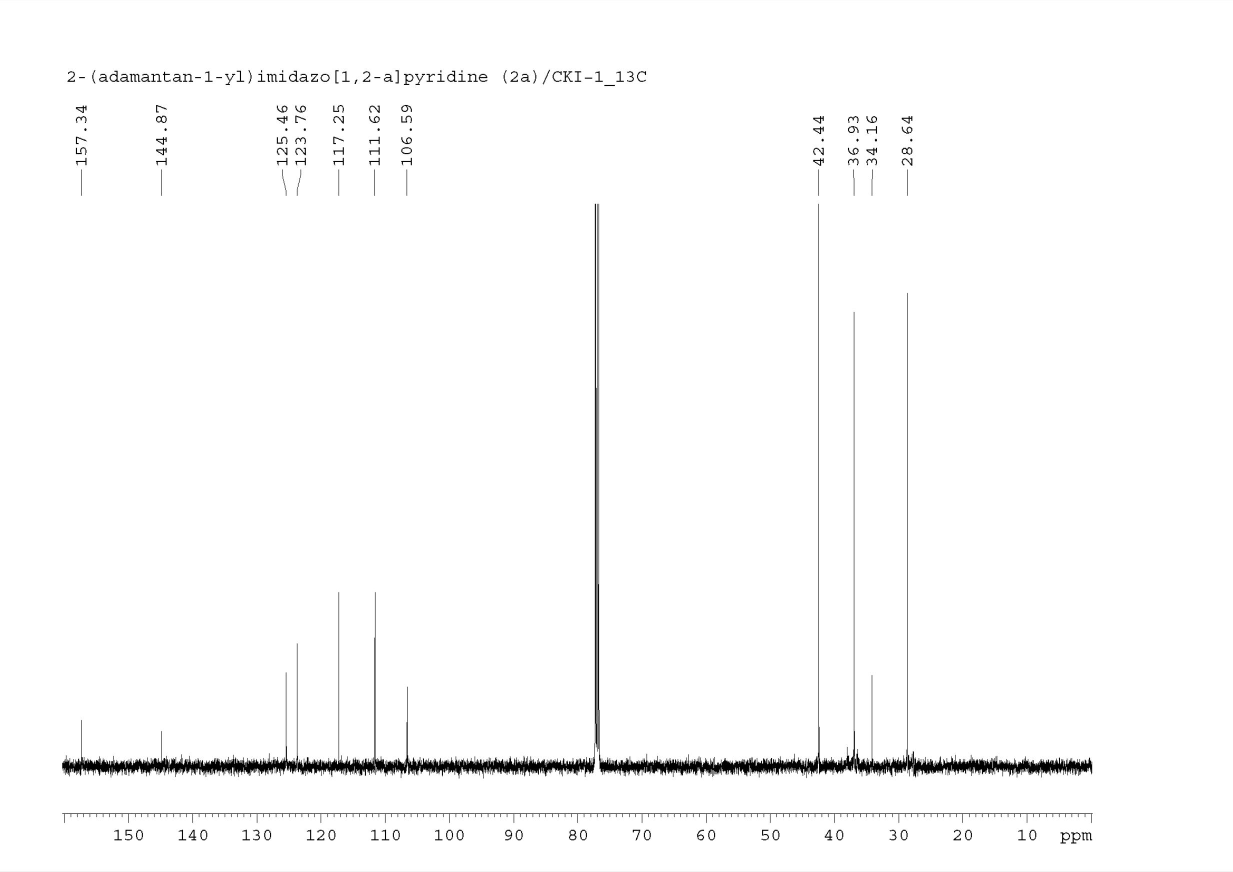

2-(adamantan-1-yl)imidazo[1,2-a]pyridine (**2a**)/CKI_1_FTIR

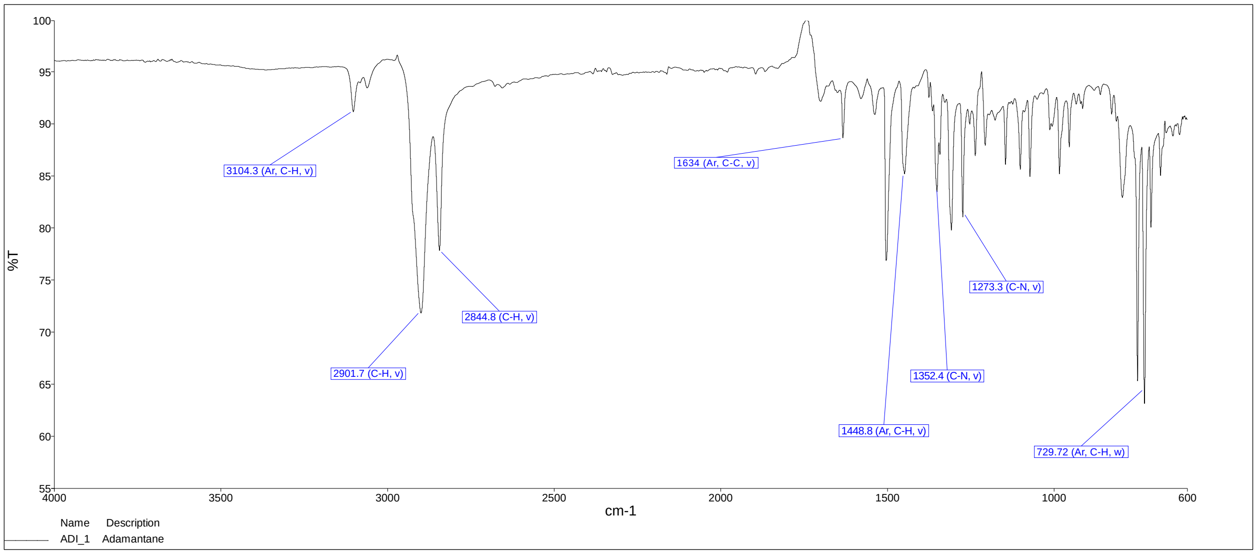

2-(adamantan-1-yl)imidazo[1,2-a]pyridine (**2a**)/CKI_1_GCMS

```
File       :C:\Users\mphslab\Desktop\zh\20161227\ADI 1 (2).D
Operator   :
Acquired   : 27 Dec 2016  16:23     using AcqMethod 20161227_SYNTHESIS_SPLIT100_1.M
Instrument :    5975C MSD
Sample Name: ADI 1 (2)
Misc Info  :
Vial Number: 2
```

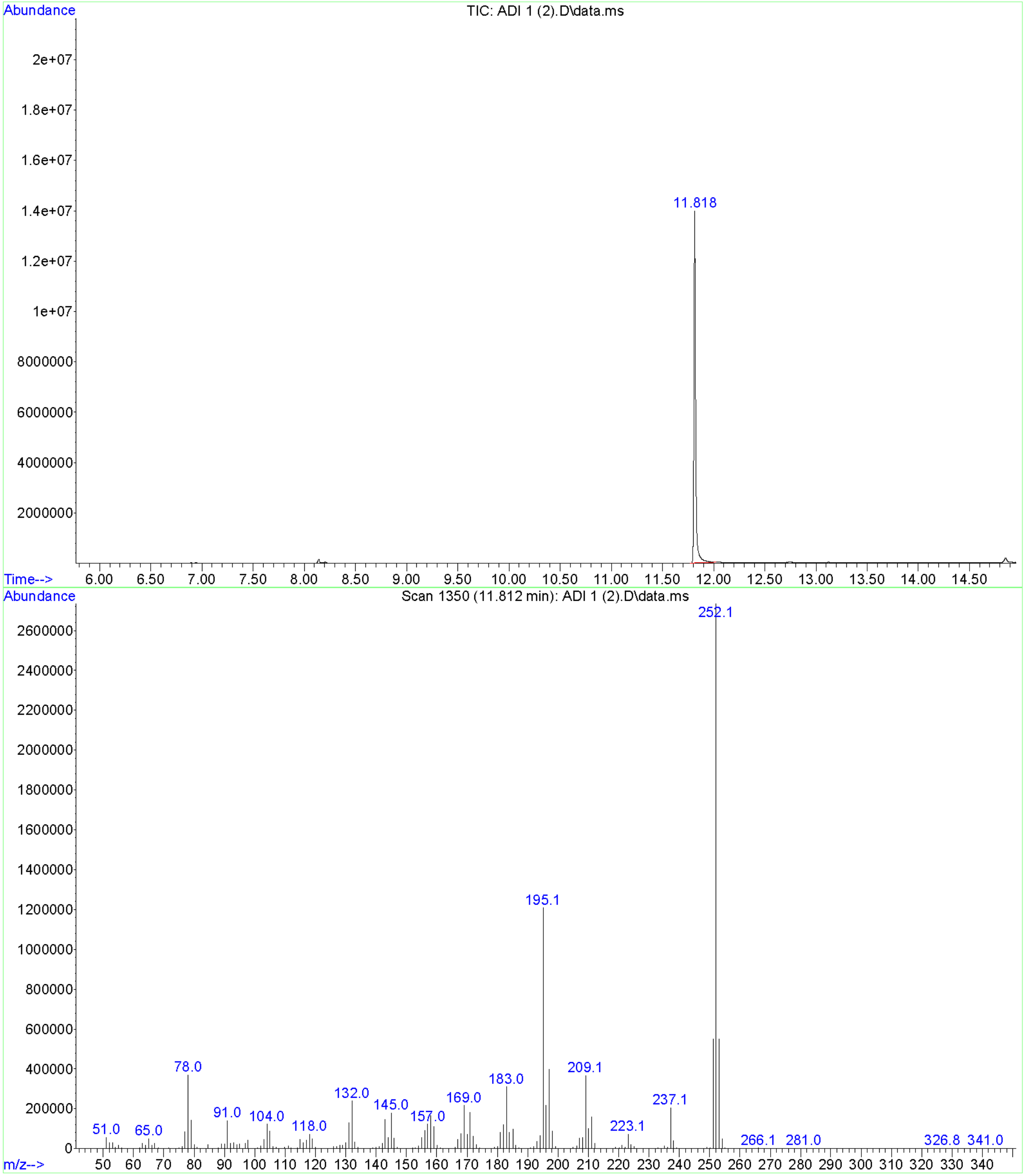

1.2 2-(adamantan-1-yl)-8-methylimidazo[1,2-a]pyridine (**2b**)

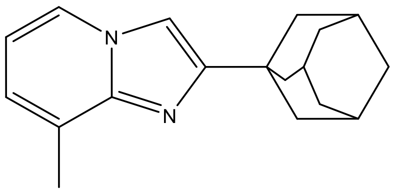

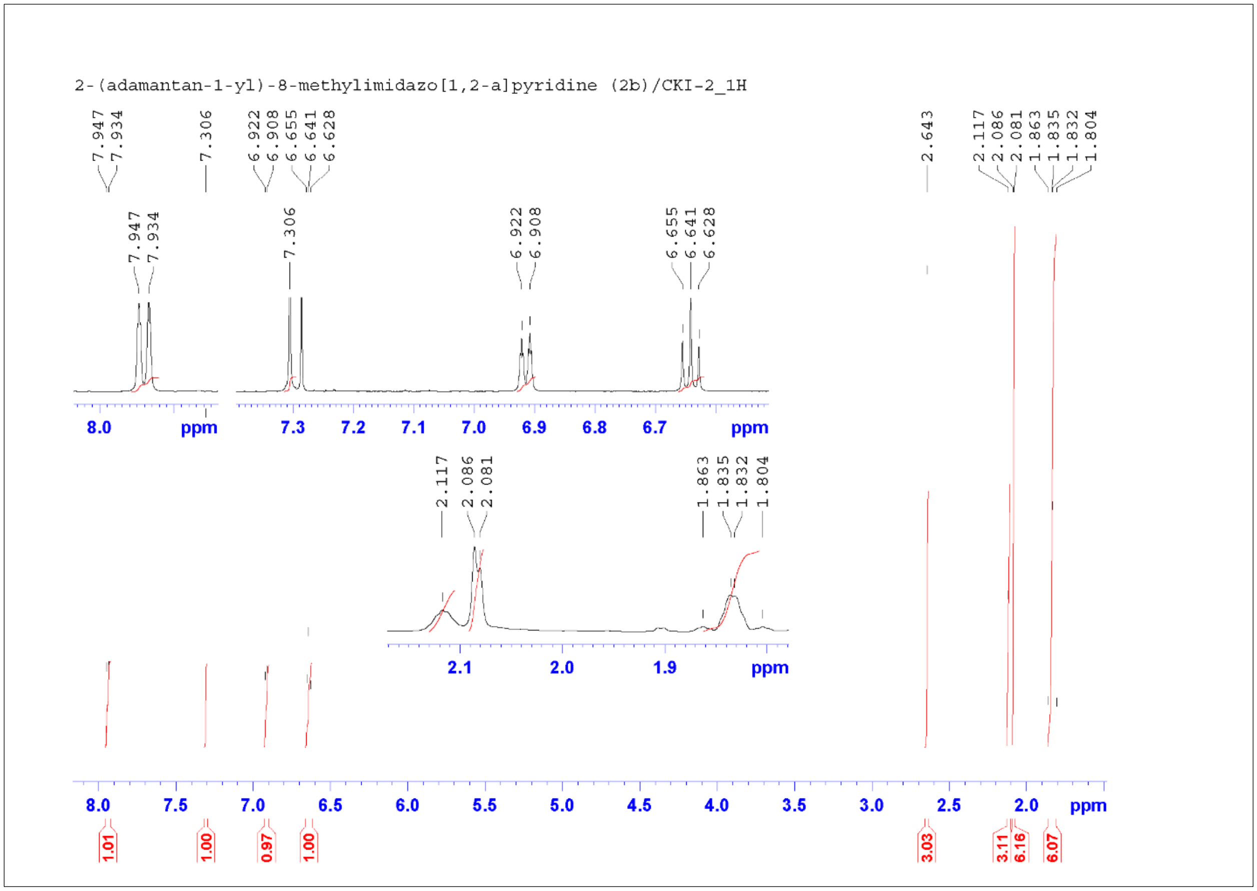

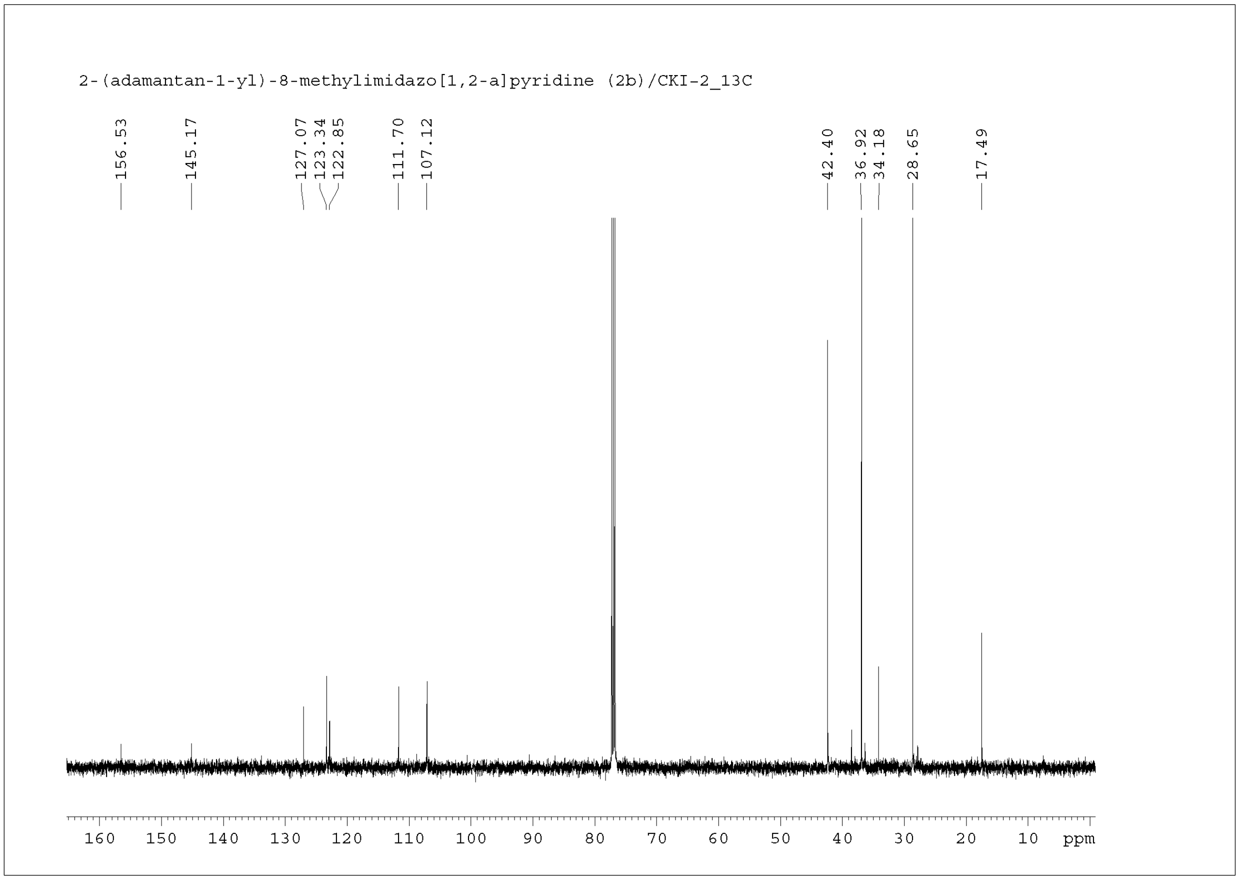

2-(adamantan-1-yl)-8-methylimidazo[1,2-a]pyridine (**2b**)/ CKI_2_FTIR

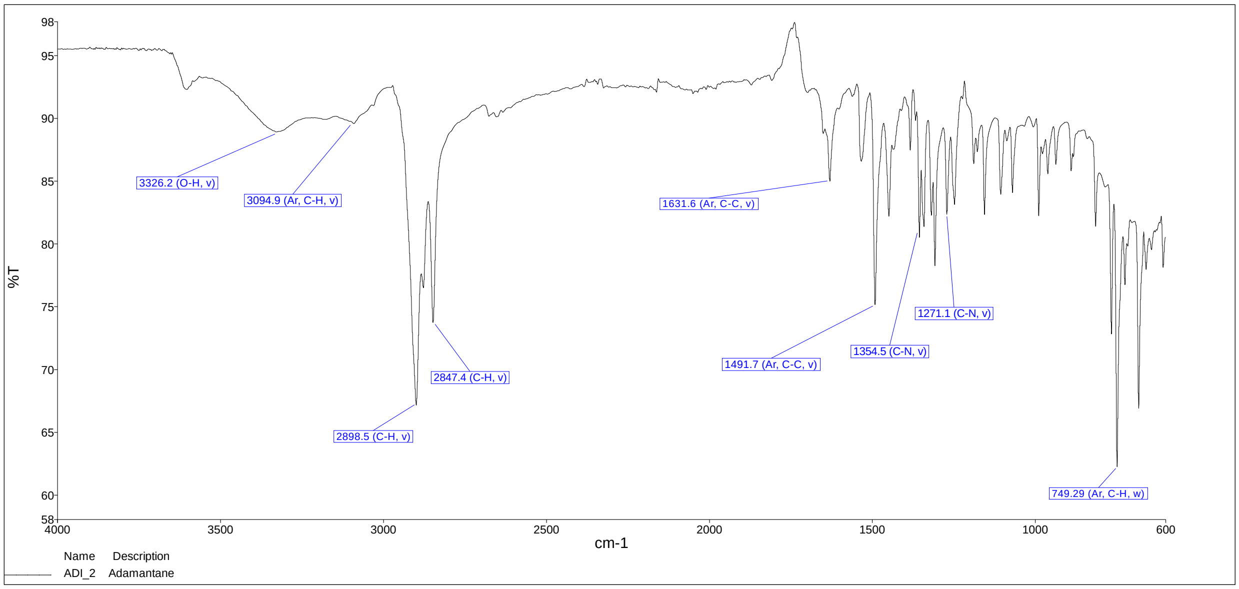

2-(adamantan-1-yl)-8-methylimidazo[1,2-a]pyridine (**2b**)/ CKI_2_GCMS

```
File       :C:\Users\mphslab\Desktop\zh\20161227\ADI 2 (2).D
Operator   :
Acquired   : 27 Dec 2016  16:46     using AcqMethod 20161227_SYNTHESIS_SPLIT100_1.M
Instrument :    5975C MSD
Sample Name: ADI 2 (2)
Misc Info  :
Vial Number: 3
```

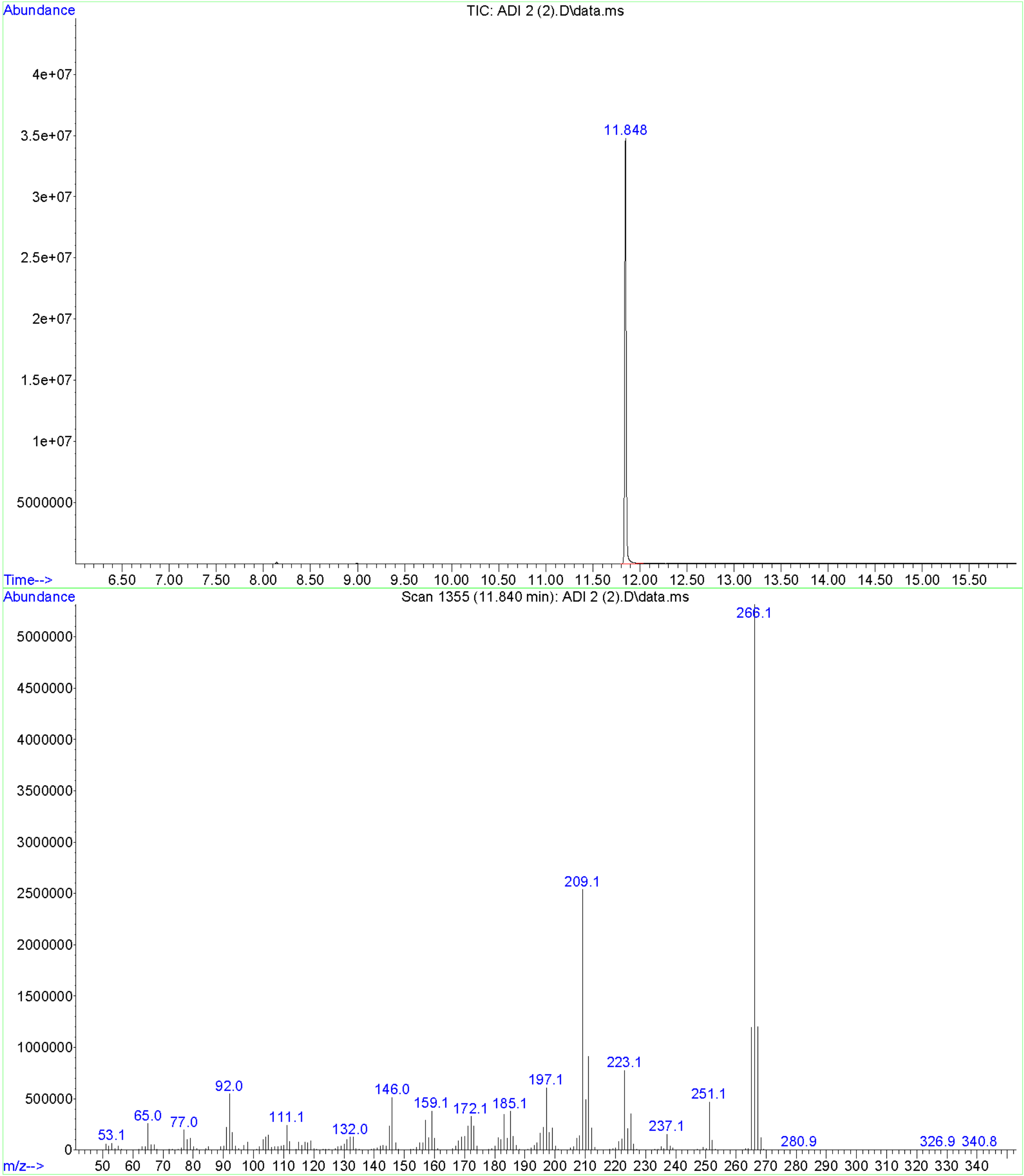

1.3 2-(adamantan-1-yl)-7-methylimidazo[1,2-a]pyridine (**2c**)

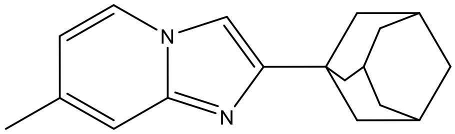

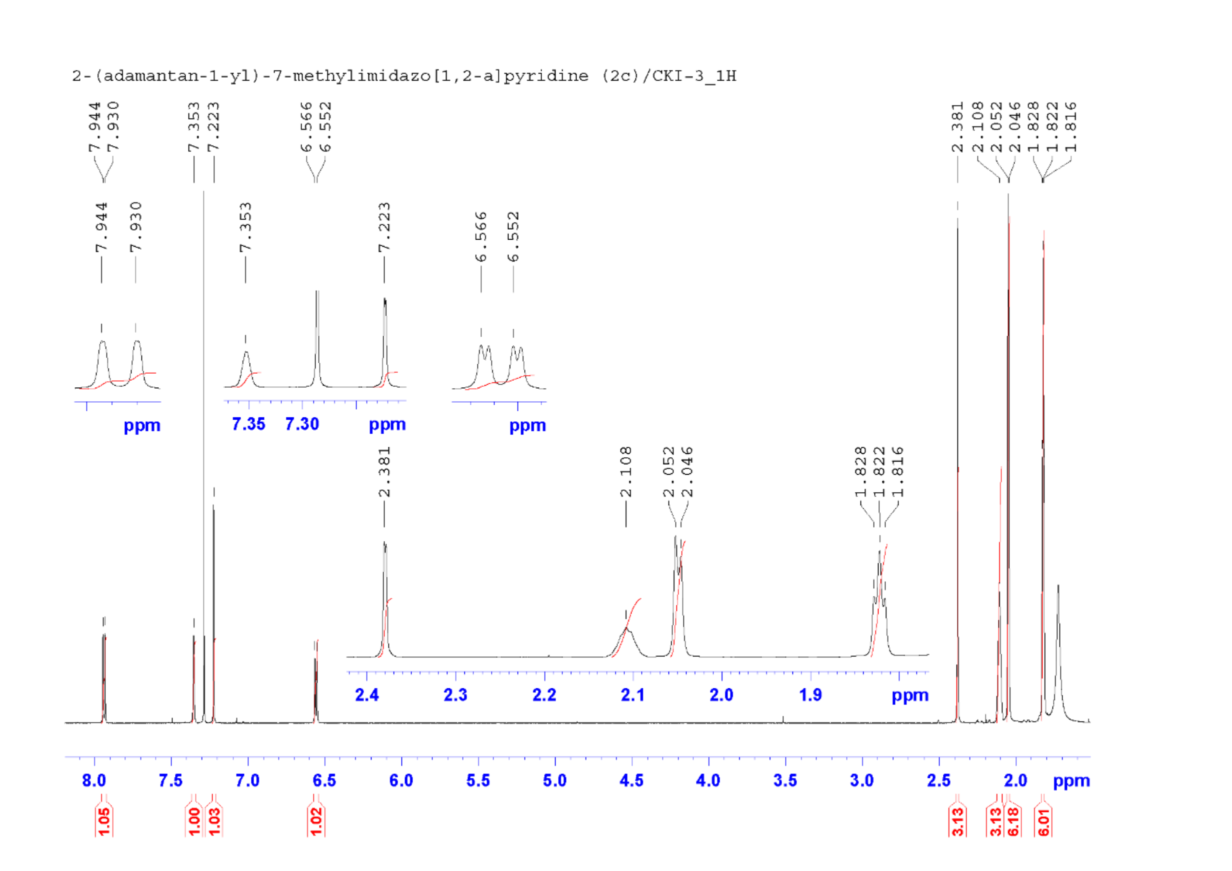

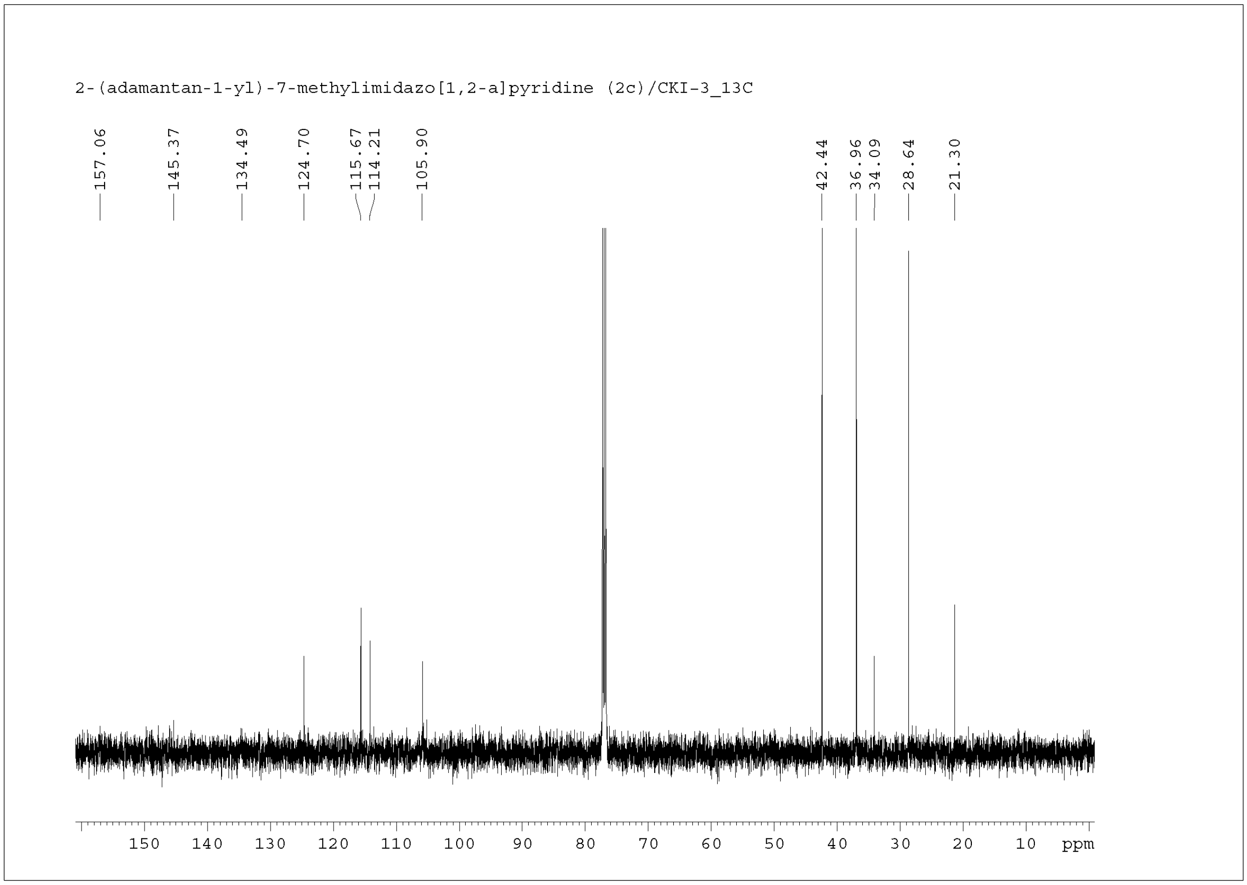

2-(adamantan-1-yl)-8-methylimidazo[1,2-a]pyridine (**2c**)/ CKI_3_FTIR

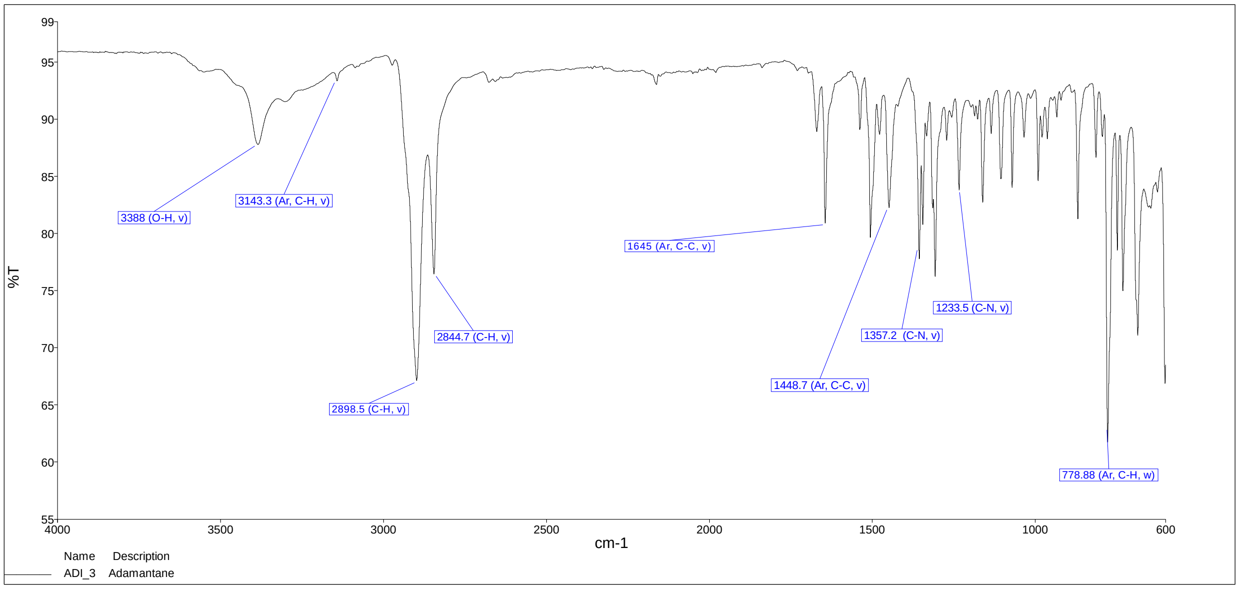

2-(adamantan-1-yl)-8-methylimidazo[1,2-a]pyridine (**2c**)/ CKI_3_GCMS

```
File        :C:\Users\mphslab\Desktop\zh\20161227\ADI 3 (2).D
Operator    :
Acquired    : 27 Dec 2016  17:09     using AcqMethod 20161227_SYNTHESIS_SPLIT100_1.M
Instrument  :    5975C MSD
Sample Name: ADI 3 (2)
Misc Info   :
Vial Number: 4
```

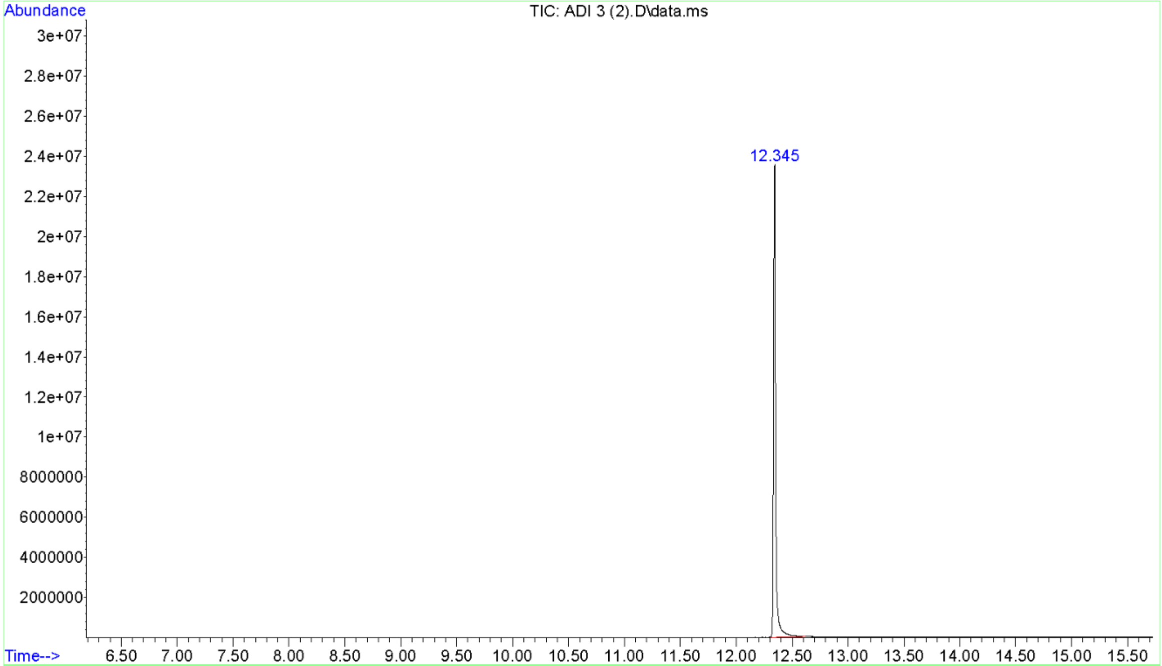

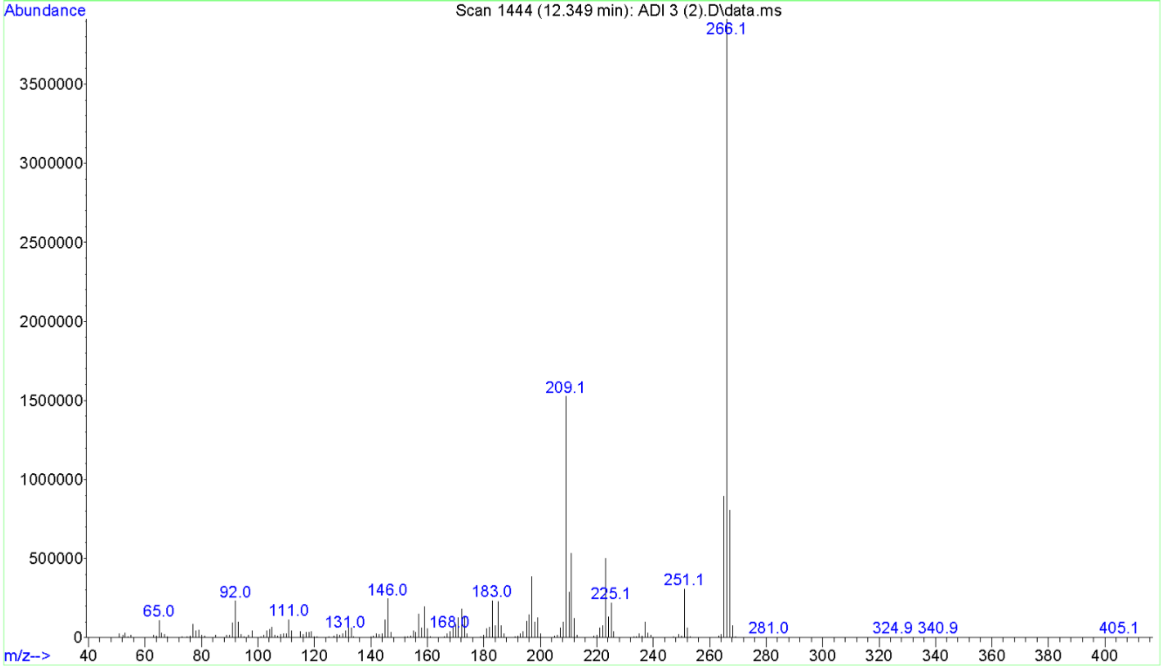

1.4 2-(adamantan-1-yl)-6-methylimidazo[1,2-a]pyridine (**2d**)

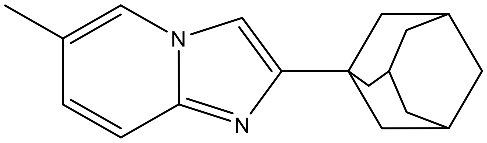

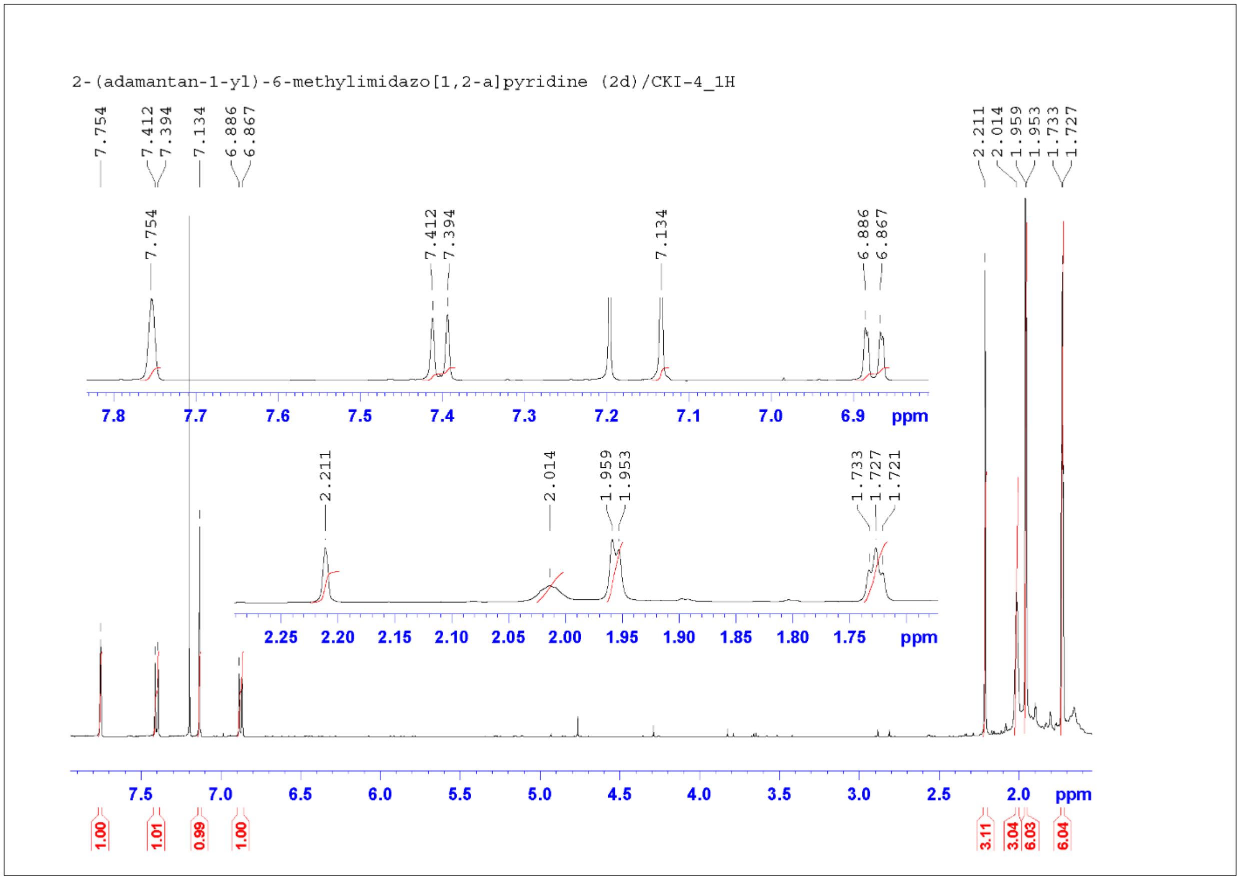

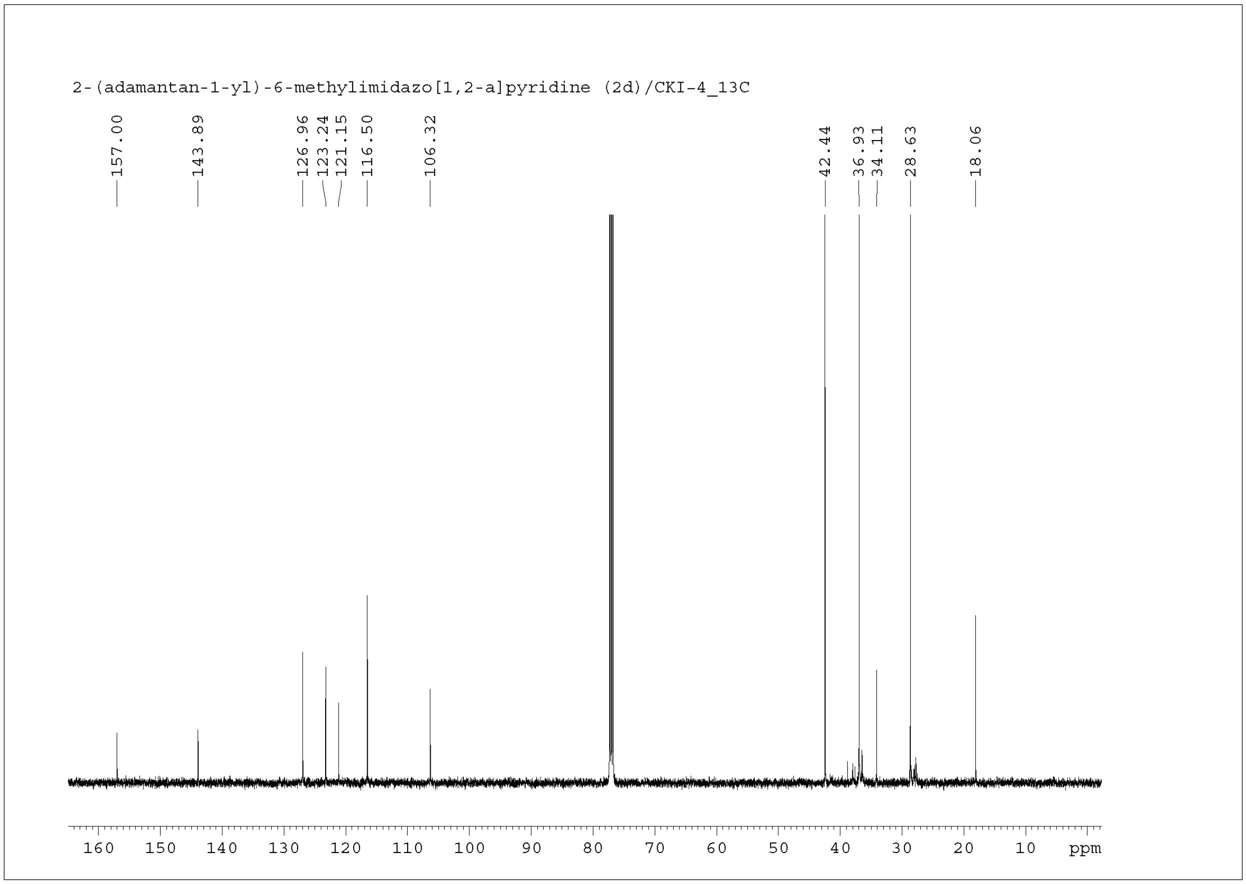

2-(adamantan-1-yl)-6-methylimidazo[1,2-a]pyridine (**2d**)/CKI_4_FTIR

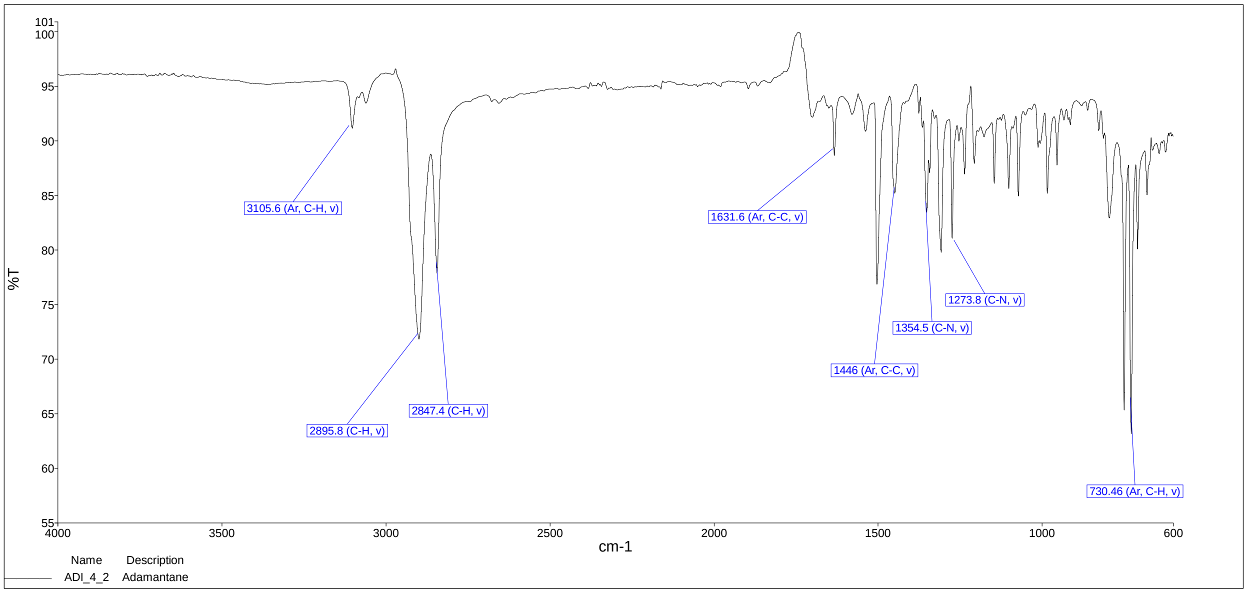

2-(adamantan-1-yl)-6-methylimidazo[1,2-a]pyridine (**2d**)/CKI_4_GCMS

```
File        :C:\Users\mphslab\Desktop\zh\20161227\ADI 4.D
Operator    :
Acquired    : 27 Dec 2016  15:49     using AcqMethod 20161227_SYNTHESIS_SPLIT100_1.M
Instrument  :    5975C MSD
Sample Name: ADI 4
Misc Info   :
Vial Number: 6
```

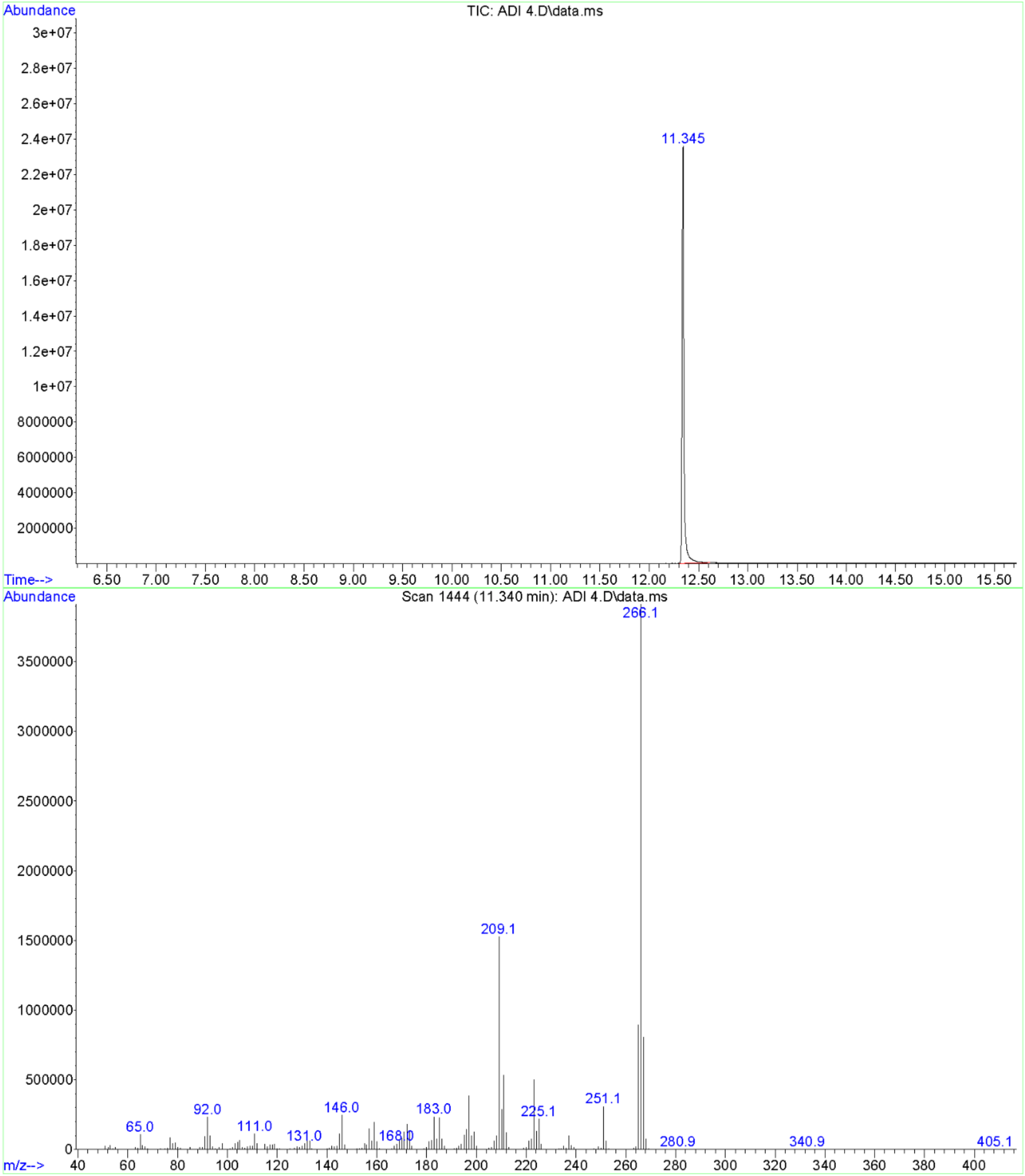

1.5 2-(adamantan-1-yl)-7-chloroimidazo[1,2-a]pyridine (**2e**)

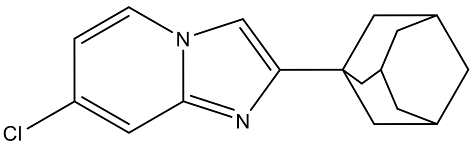

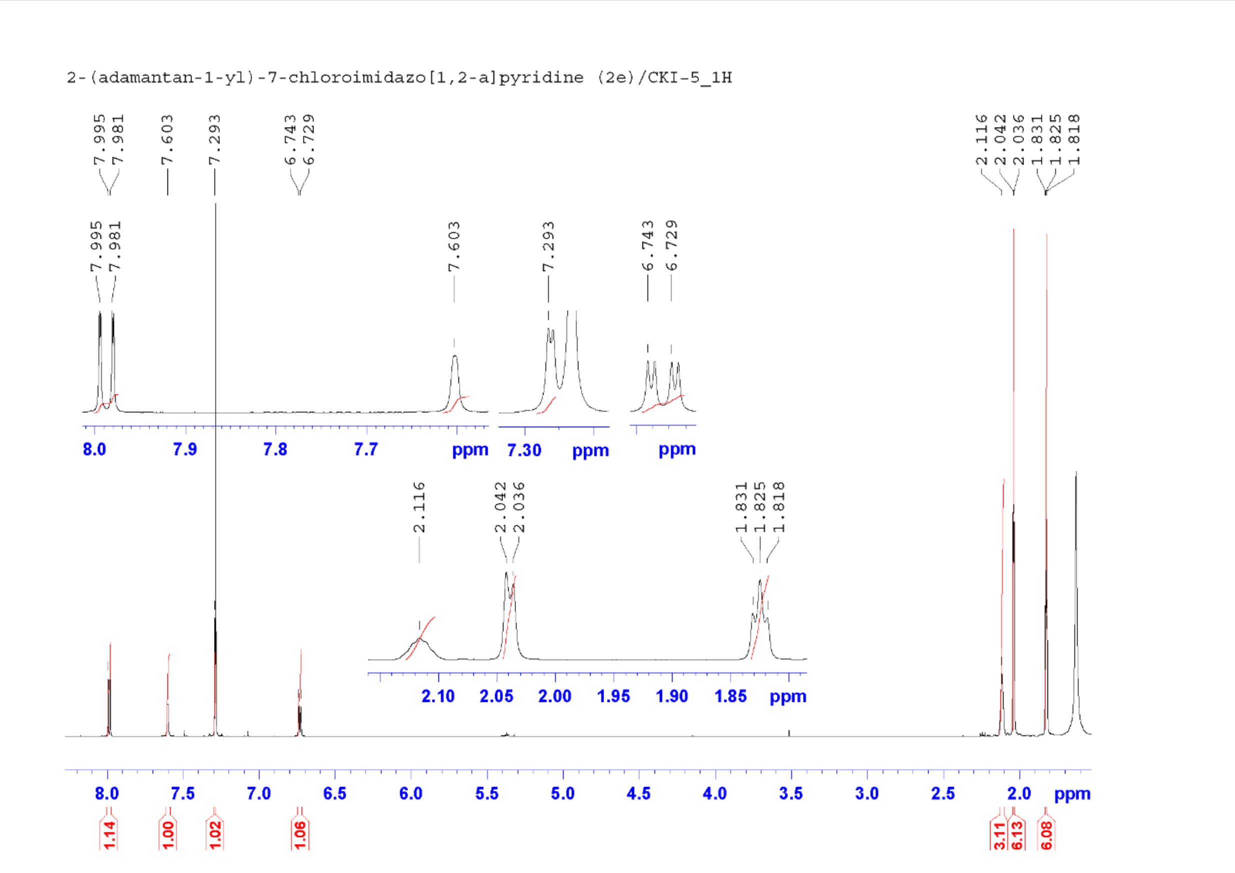

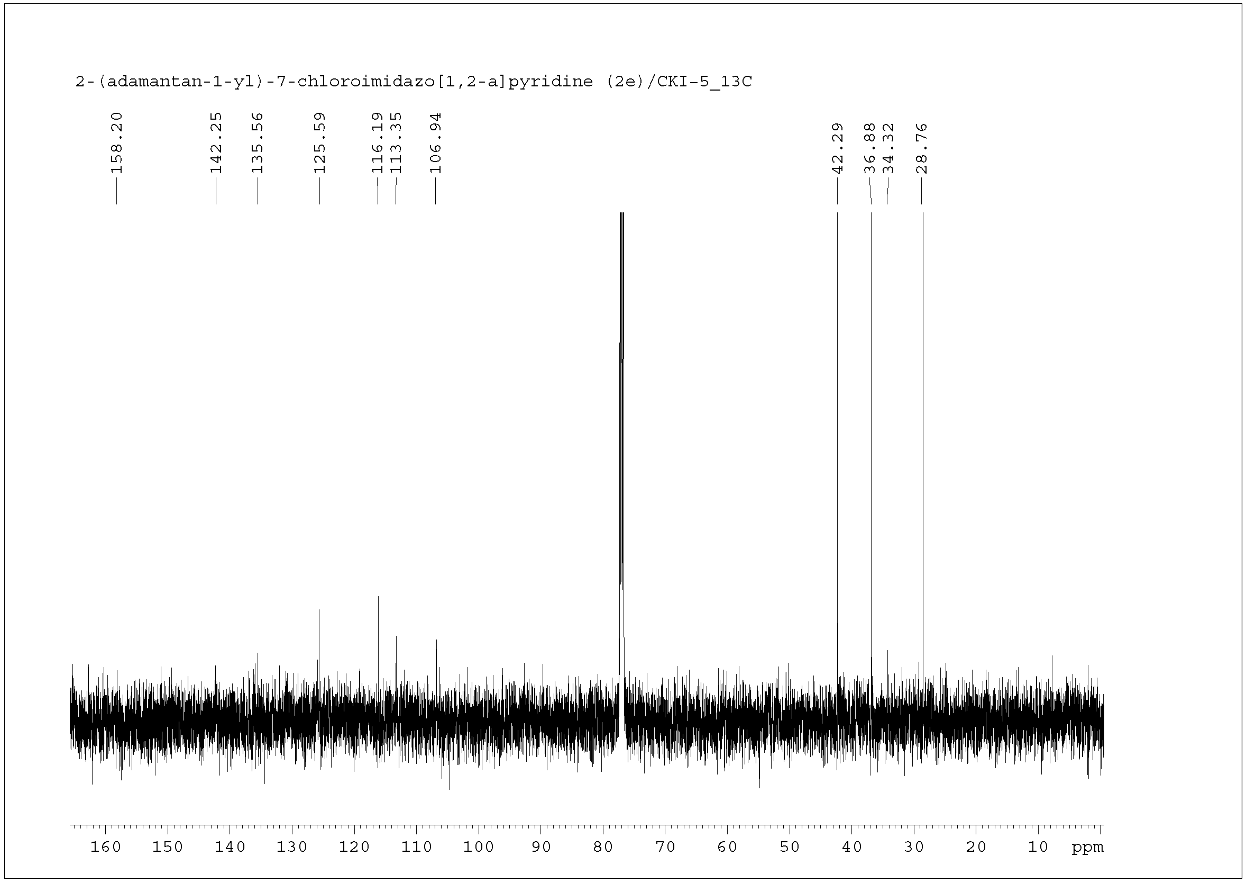

2-(adamantan-1-yl)-7-chloroimidazo[1,2-a]pyridine (**2e**)/CKI_5_FTIR

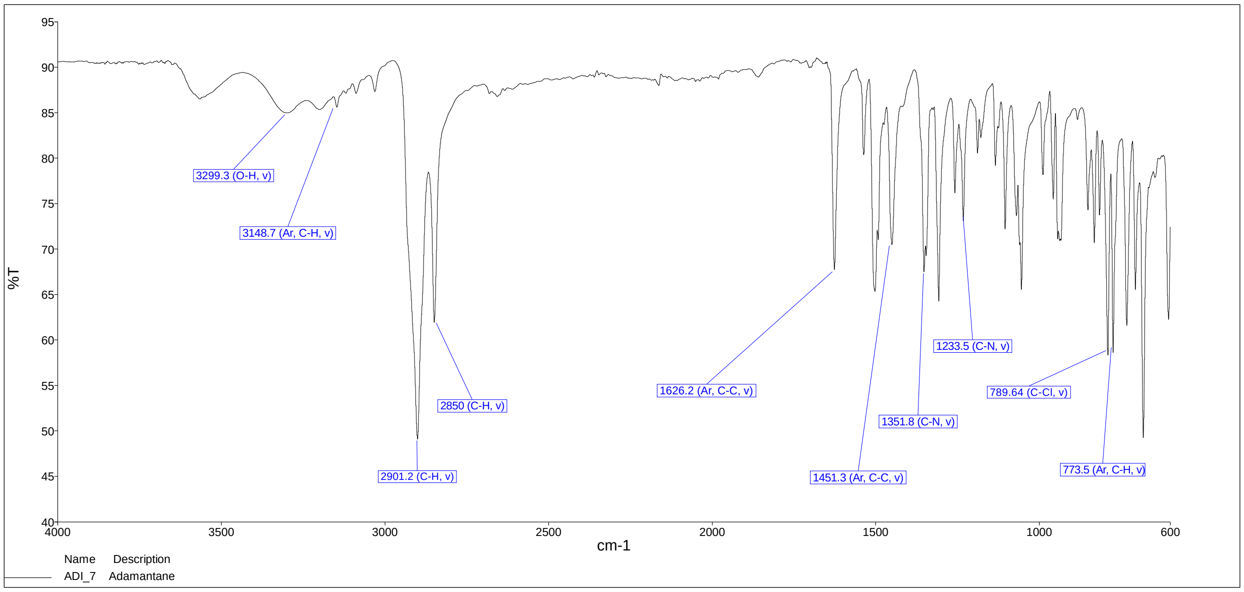

2-(adamantan-1-yl)-7-chloroimidazo[1,2-a]pyridine (**2e**)/CKI_5_GCMS

```
File        :C:\Users\mphslab\Desktop\zh\20161227\ADI 7.D
Operator    :
Acquired    : 27 Dec 2016  15:23     using AcqMethod 20161227_SYNTHESIS_SPLITLESS.M
Instrument  :    5975C MSD
Sample Name: ADI 7
Misc Info   :
Vial Number: 5
```

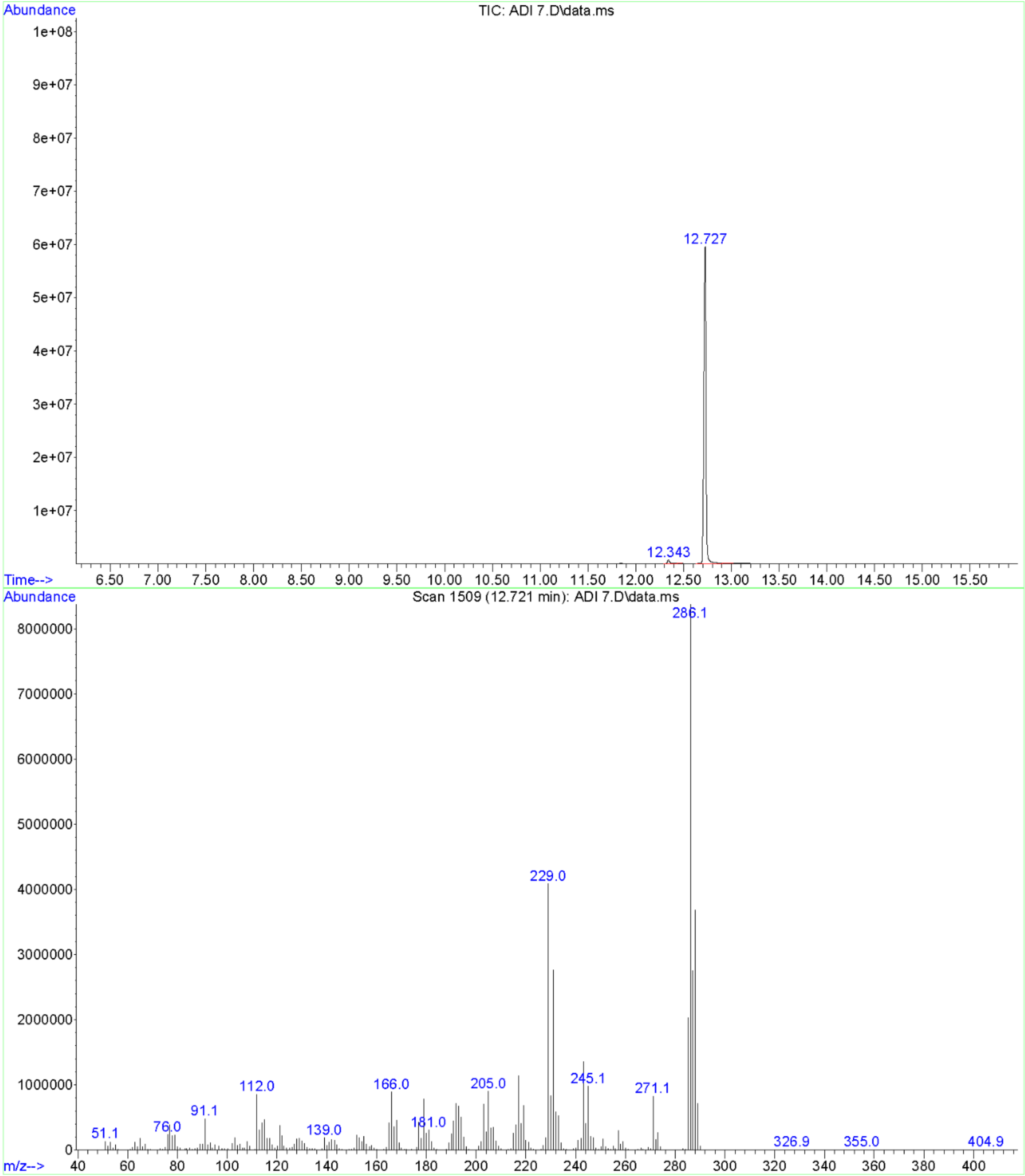

1.6 2-([1,1'-biphenyl]-4-yl)imidazo[1,2-a]pyridine (**2f**)

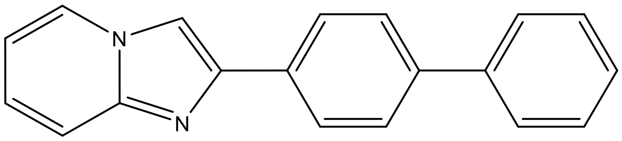

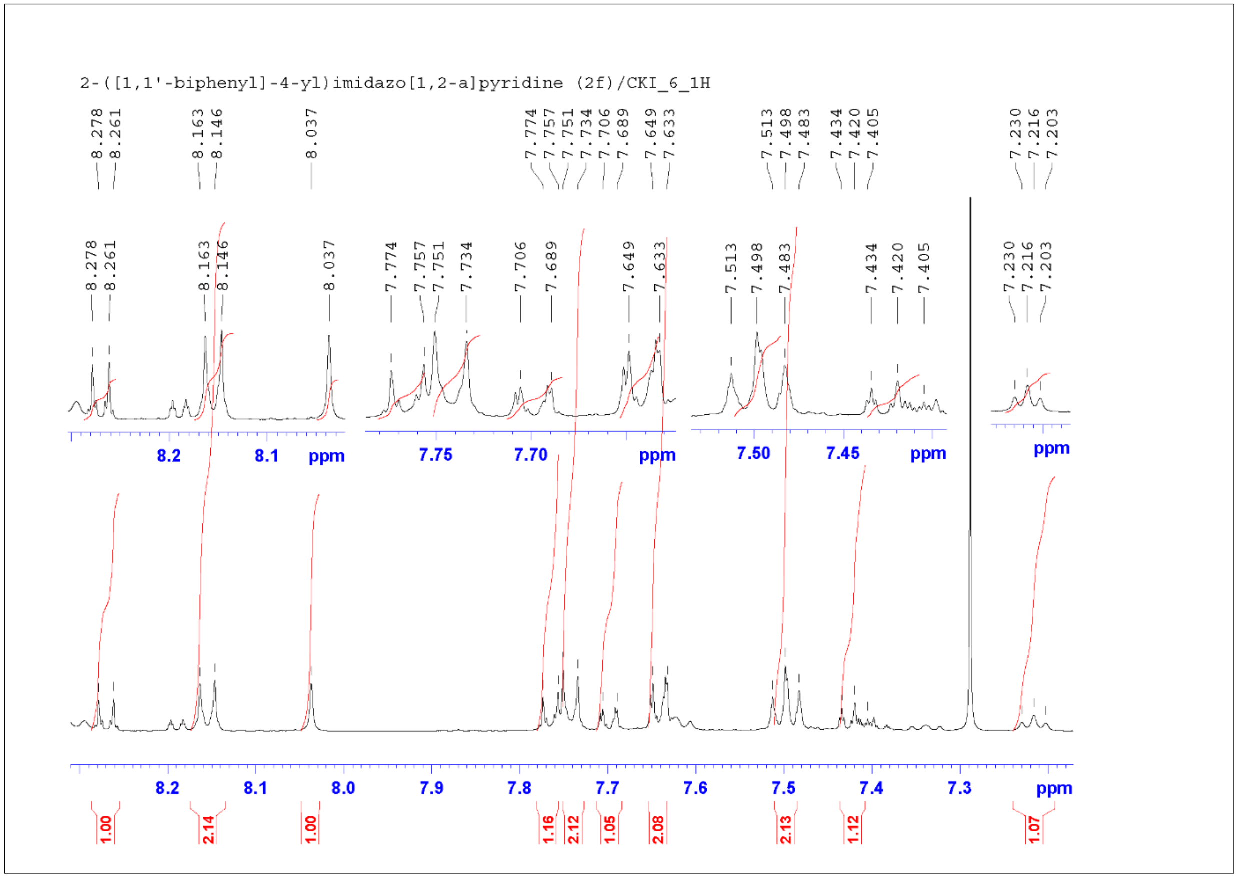

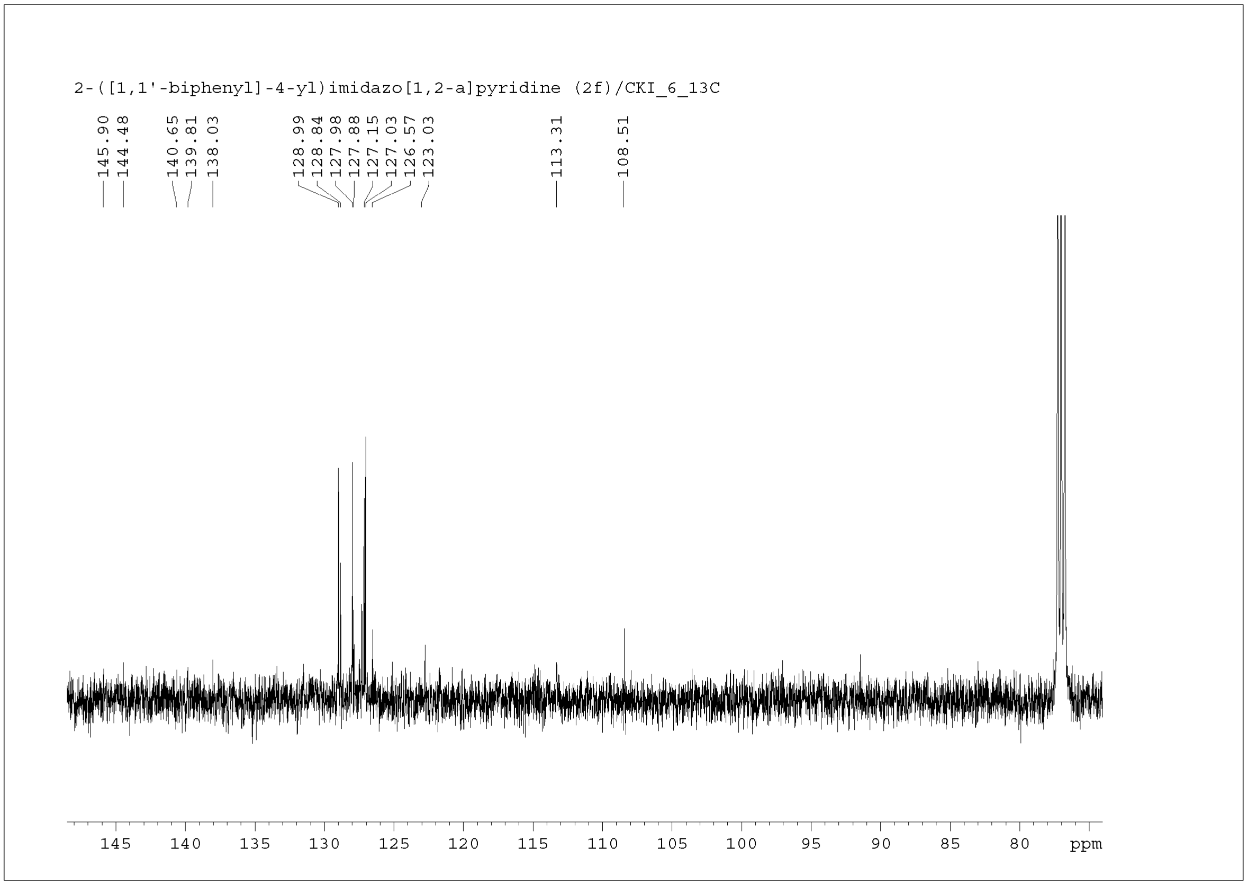

2-([1,1'-biphenyl]-4-yl)imidazo[1,2-a]pyridine (**2f**)/CKI_6_FTIR

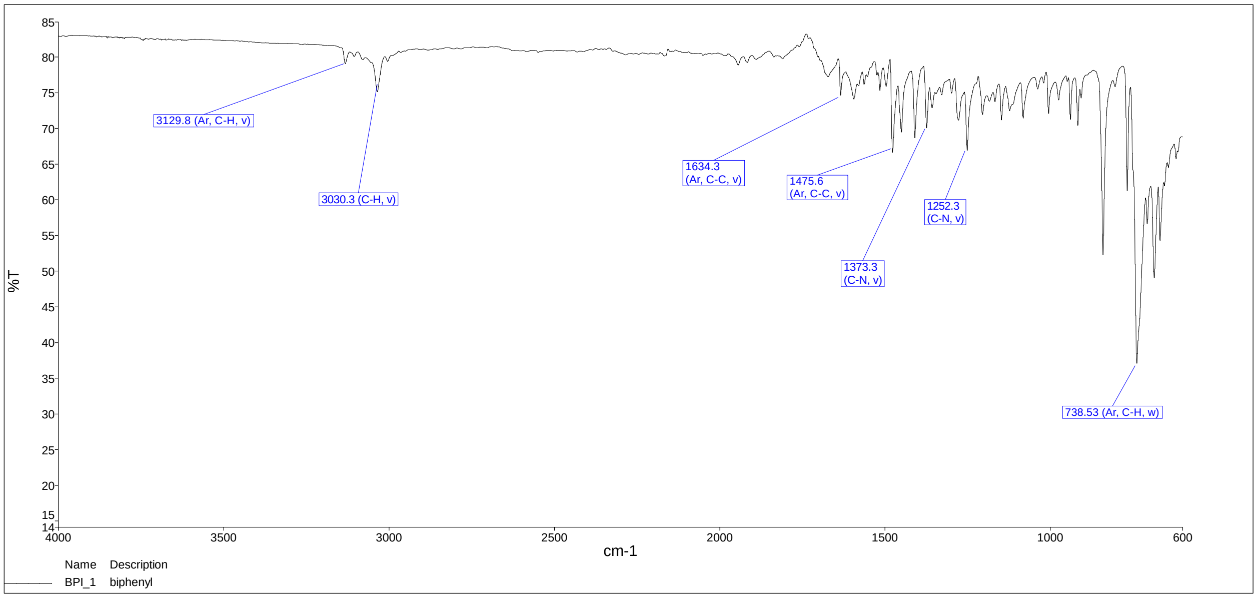

2-([1,1'-biphenyl]-4-yl)imidazo[1,2-a]pyridine (**2f**)/CKI_6_GCMS

```
File        :C:\Users\mphslab\Desktop\zh\20160412_B1.D
Operator    :
Acquired    : 12 Apr 2016  12:48     using AcqMethod SYNTHESIS_SPLITRATIO100_1.M
Instrument  :    5975C MSD
Sample Name:
Misc Info   :
Vial Number: 3
```

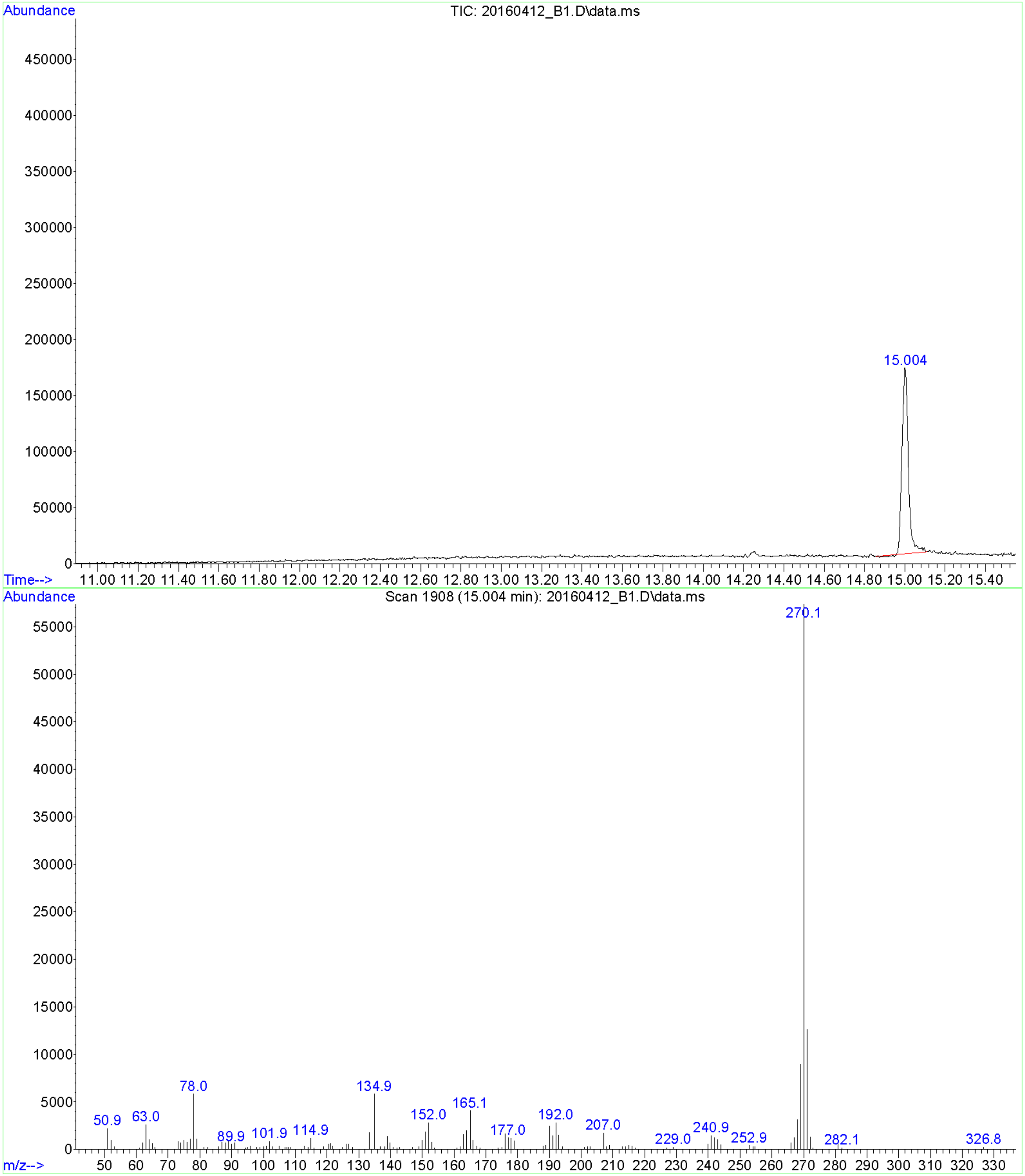

1.7 2-([1,1'-biphenyl]-4-yl)-8-methylimidazo[1,2-a]pyridine (**2g**)

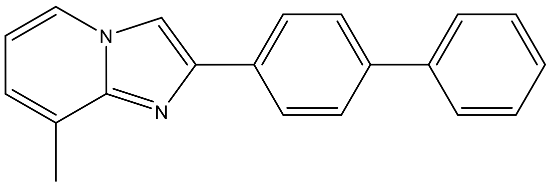

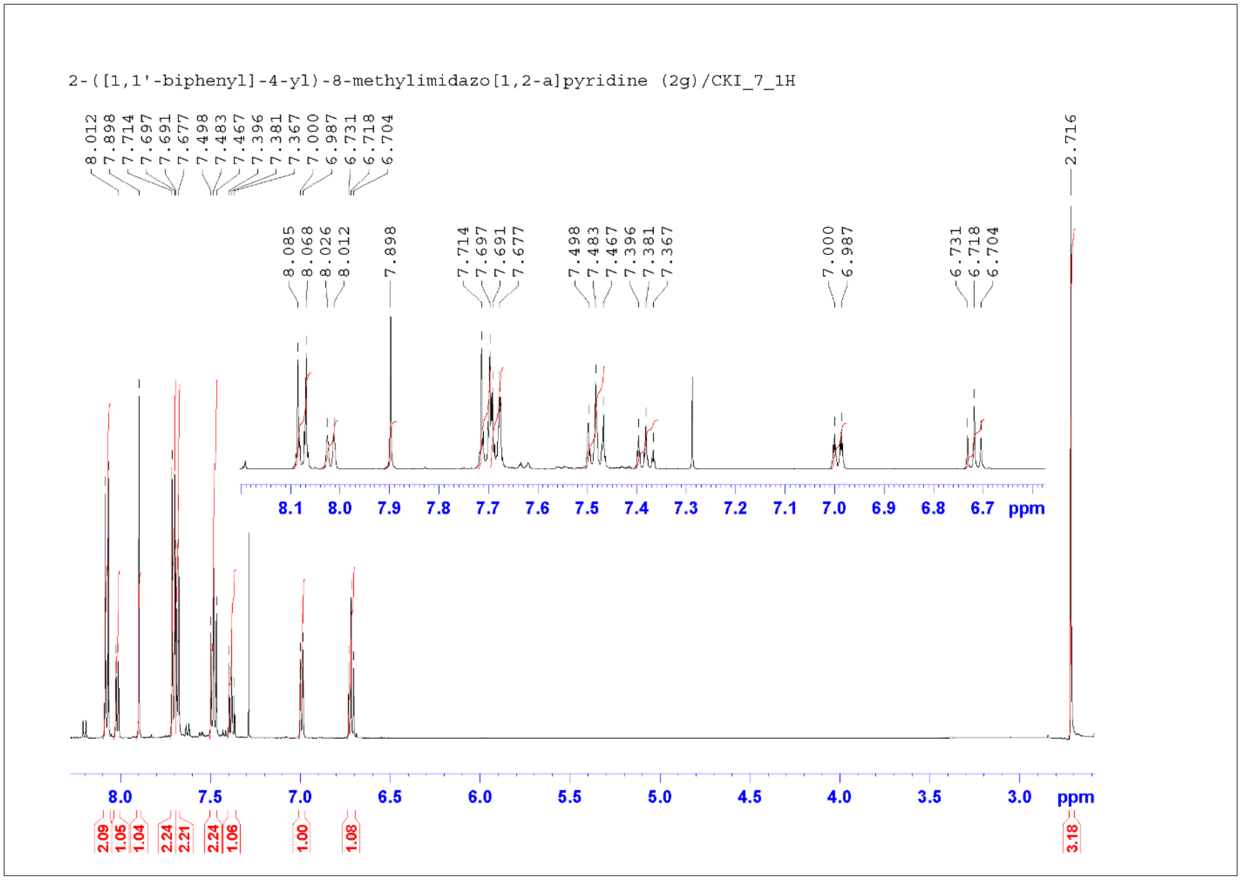

2-([1,1'-biphenyl]-4-yl)-8-methylimidazo[1,2-a]pyridine (2g)/CKI_7_13C

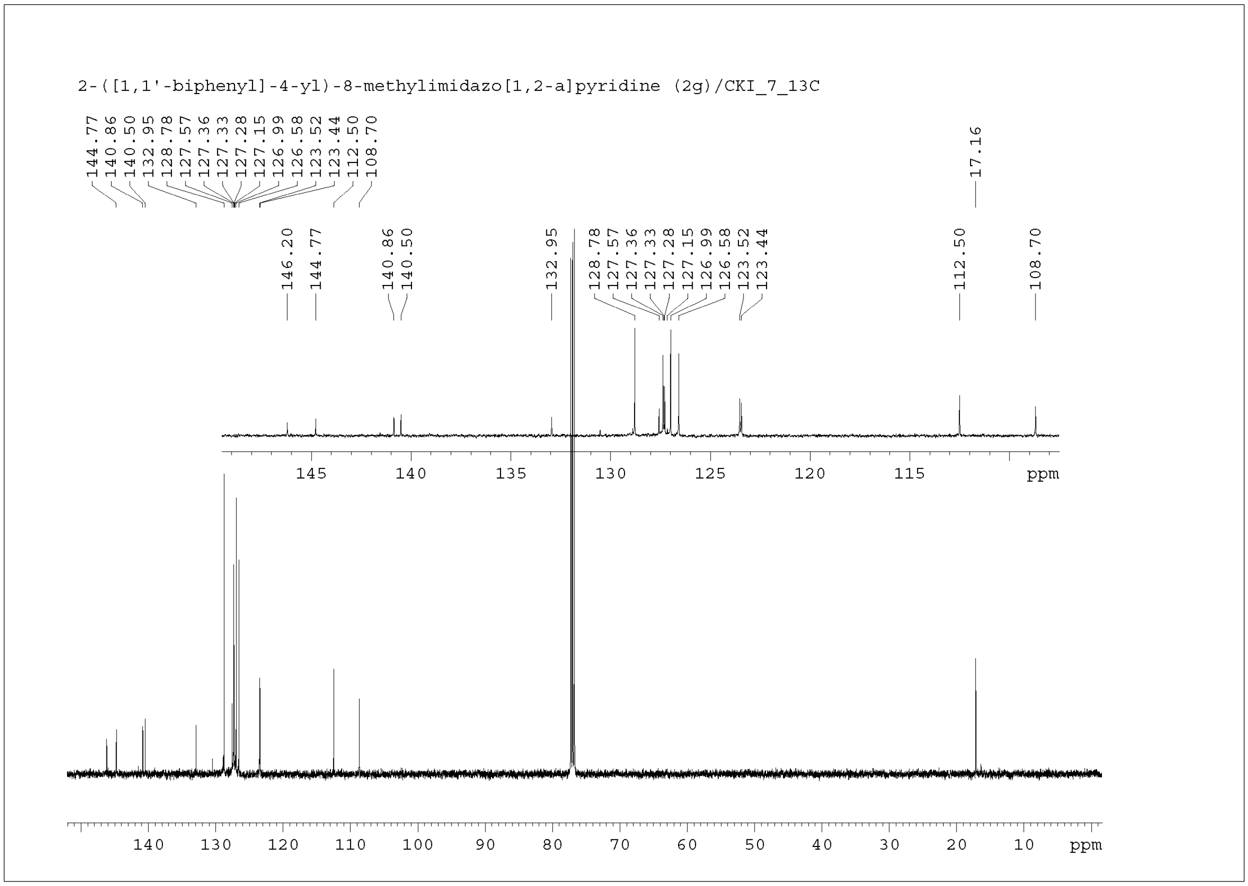

2-([1,1'-biphenyl]-4-yl)-8-methylimidazo[1,2-a]pyridine (**2g**)/CKI_7_FTIR

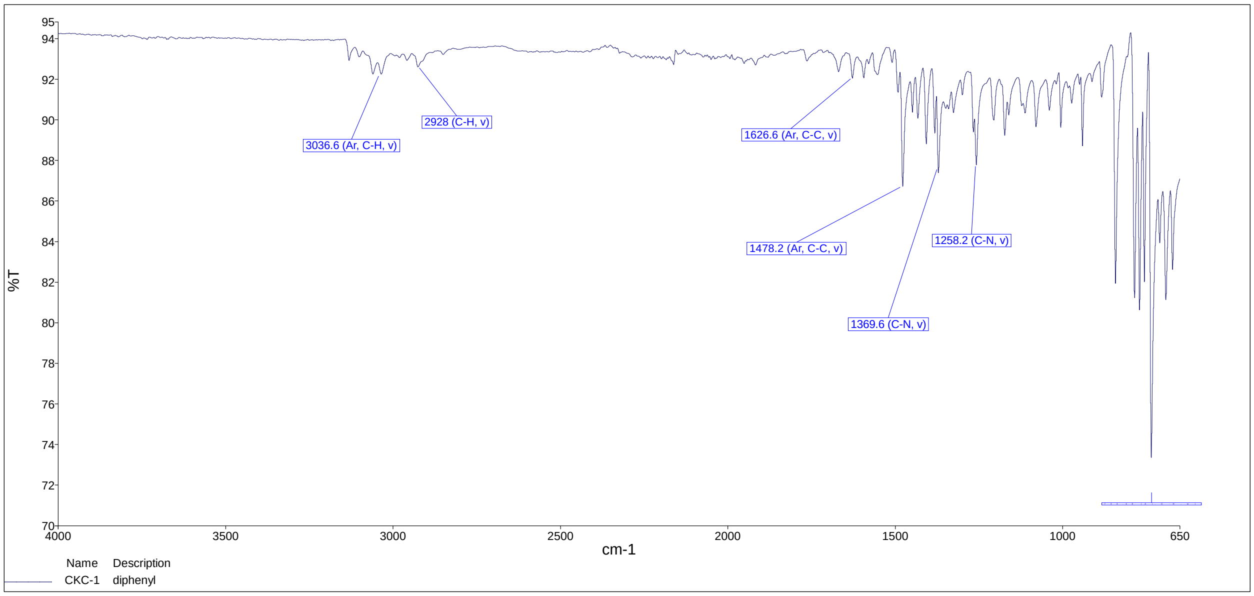

2-([1,1'-biphenyl]-4-yl)-8-methylimidazo[1,2-a]pyridine (**2g**)/CKI_7_GCMS

```
File        :C:\Users\mphslab\Desktop\zh\20160413_B2.D
Operator    : ZH
Acquired    : 13 Apr 2016  11:19     using AcqMethod PLANT EXTRACT SPLITLESS.M
Instrument :    5975C MSD
Sample Name: 20160413 B2
Misc Info  :
Vial Number: 4
```

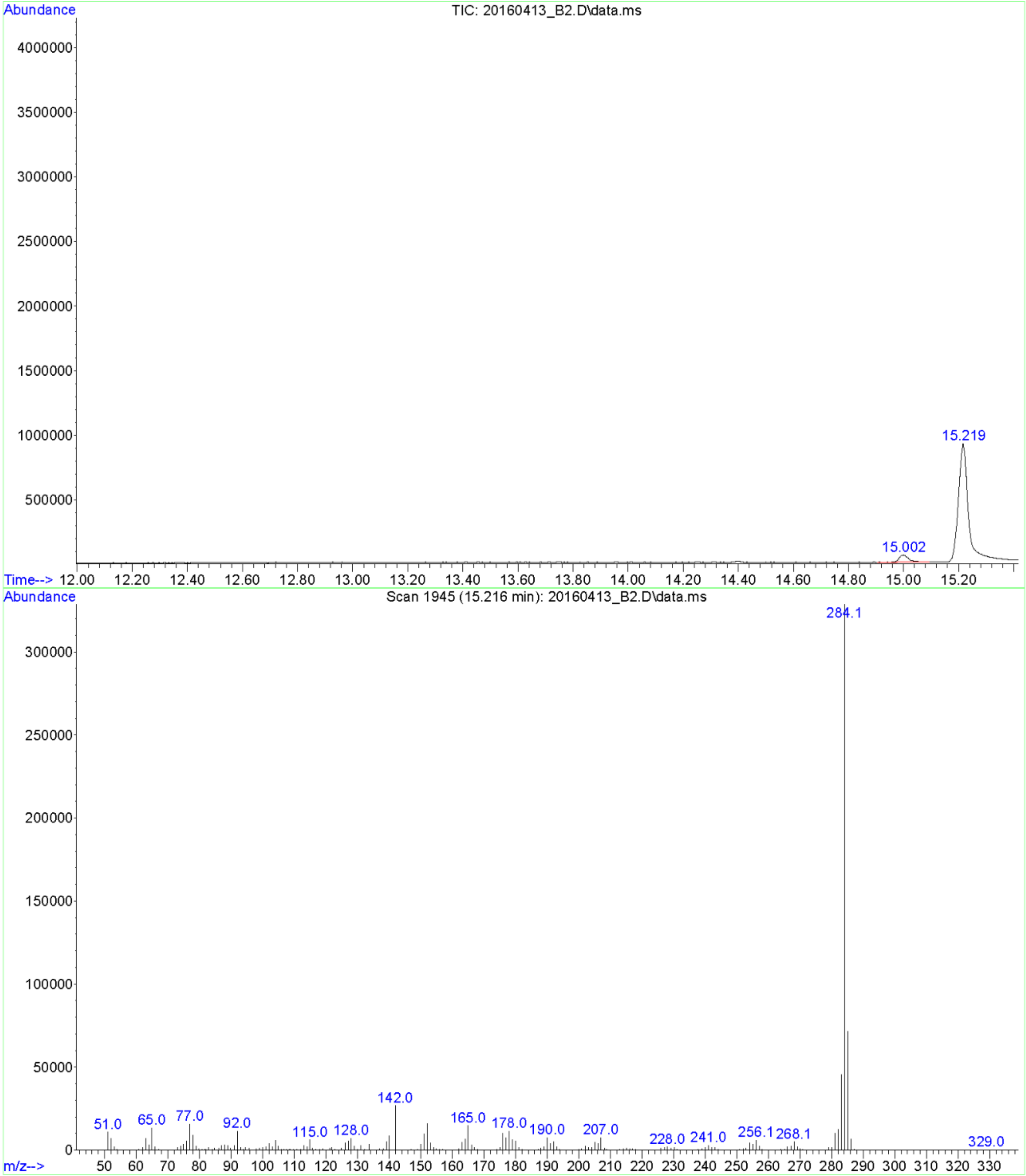

1.8 2-([1,1'-biphenyl]-4-yl)-6-methylimidazo[1,2-a]pyridine (**2h**)

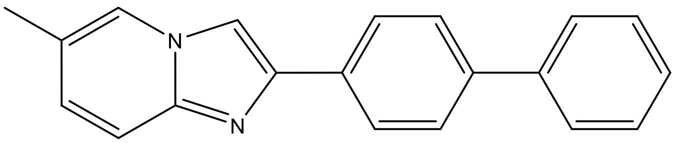

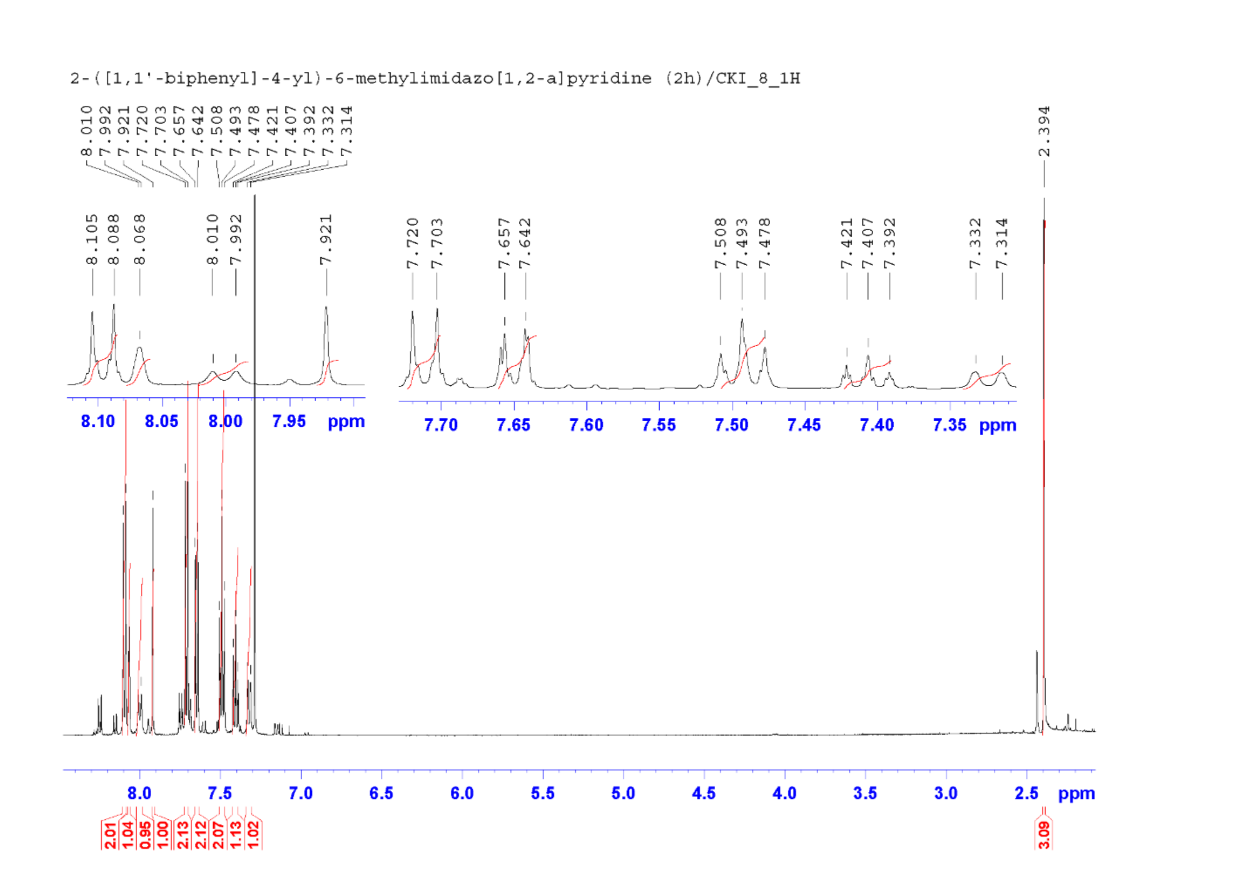

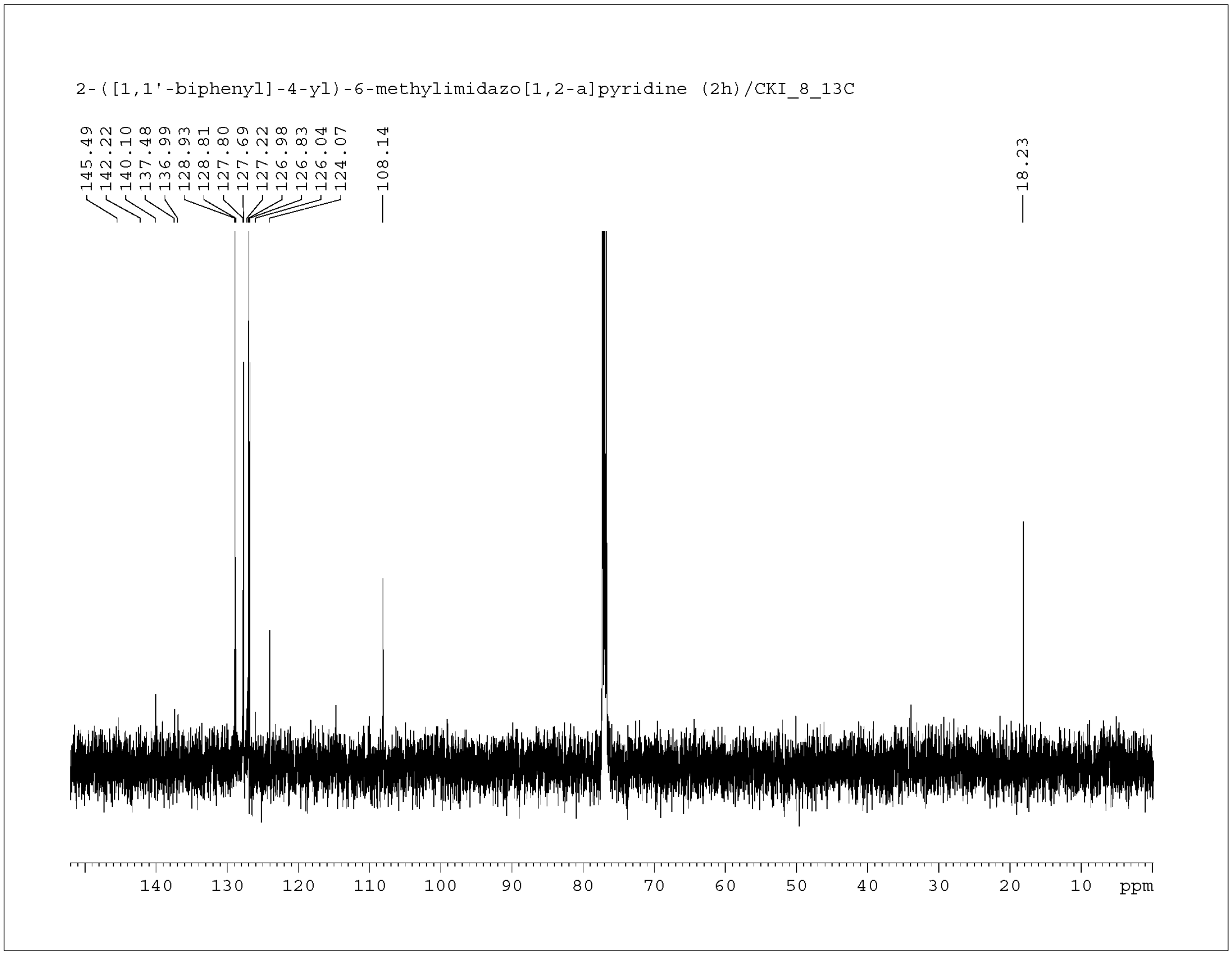

2-([1,1'-biphenyl]-4-yl)-6-methylimidazo[1,2-a]pyridine (**2h**)/CKI_8_FTIR

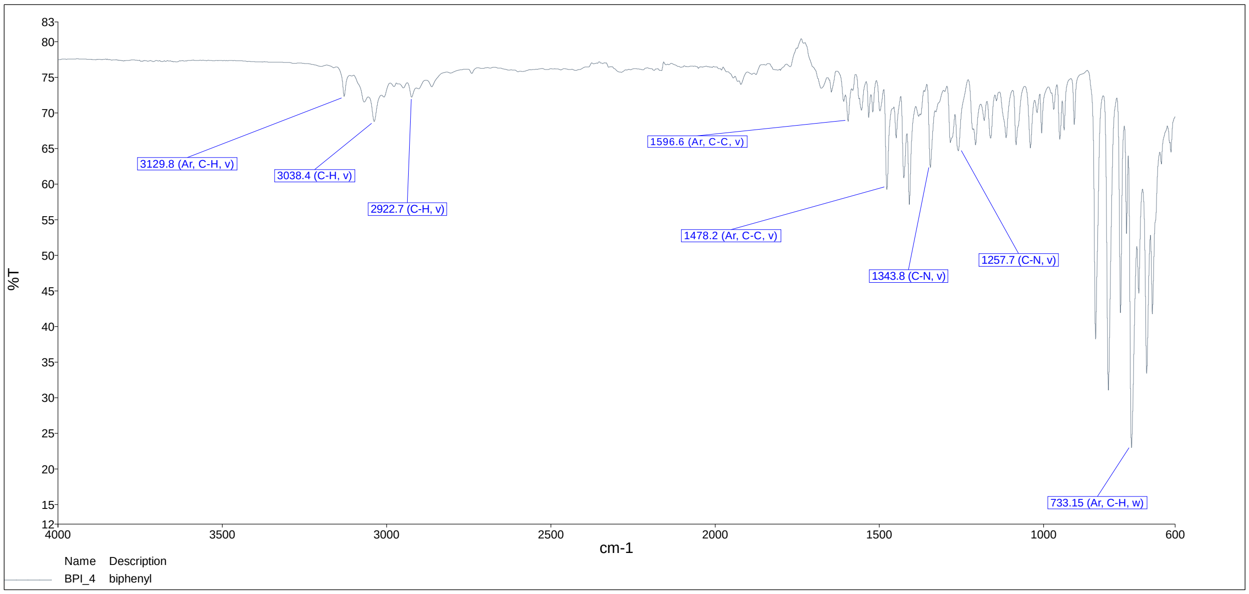

2-([1,1'-biphenyl]-4-yl)-6-methylimidazo[1,2-a]pyridine (**2h**)/CKI_8_GCMS

```
File       :C:\Users\mphslab\Desktop\zh\20160503_B4 splitless.D
Operator   : ZH
Acquired   :  3 May 2016  15:50     using AcqMethod PLANT EXTRACT SPLITLESS.M
Instrument :    5975C MSD
Sample Name: 20160503 B4 splitless
Misc Info  :
Vial Number: 1
```

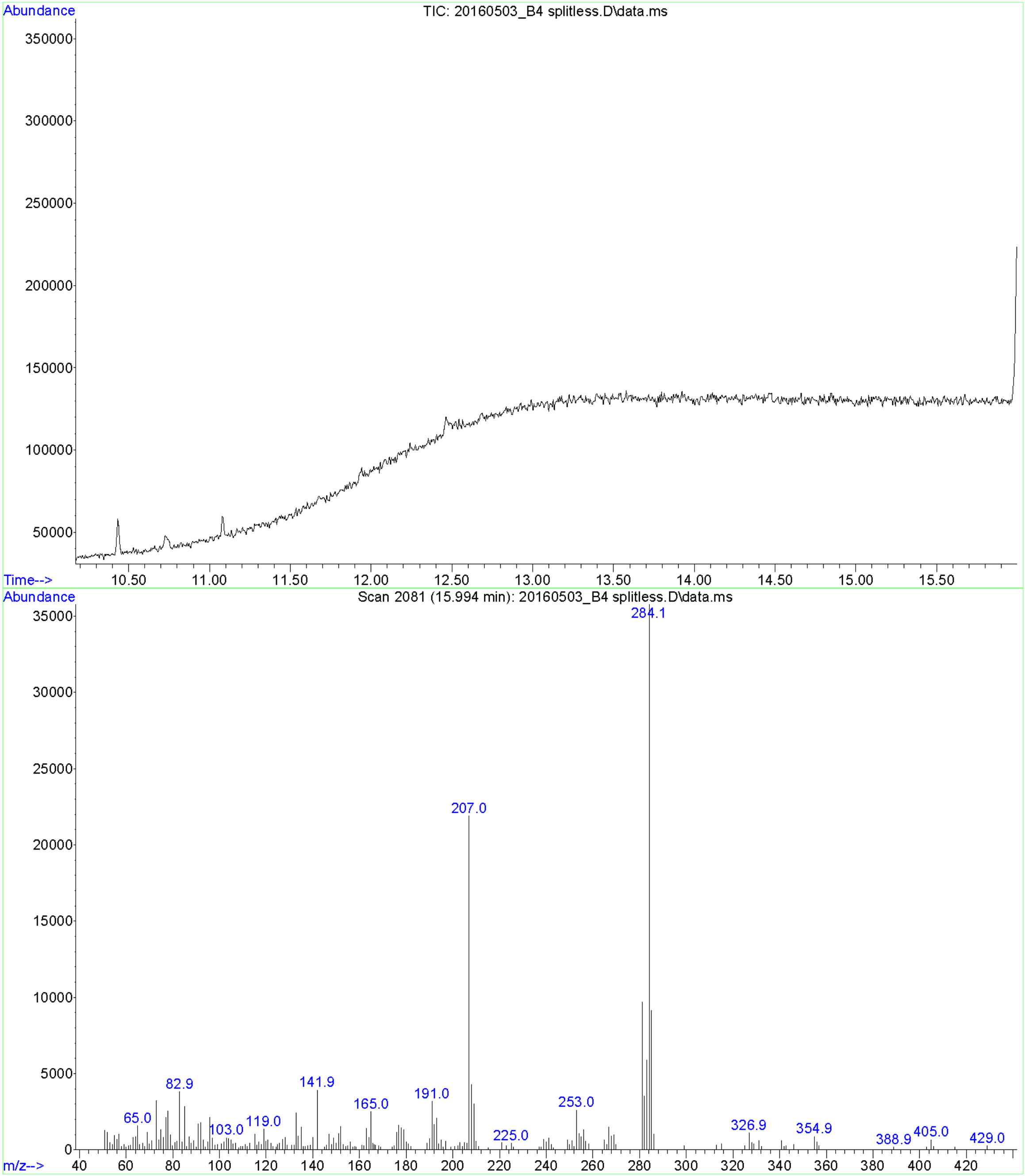

1.9 2-([1,1'-biphenyl]-4-yl)-8-chloroimidazo[1,2-a]pyridine (**2i**)

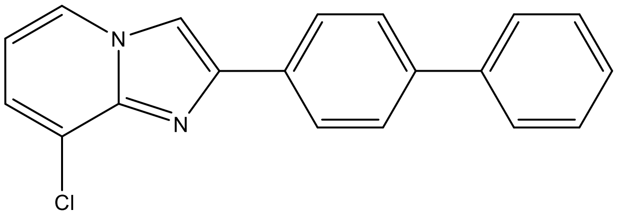

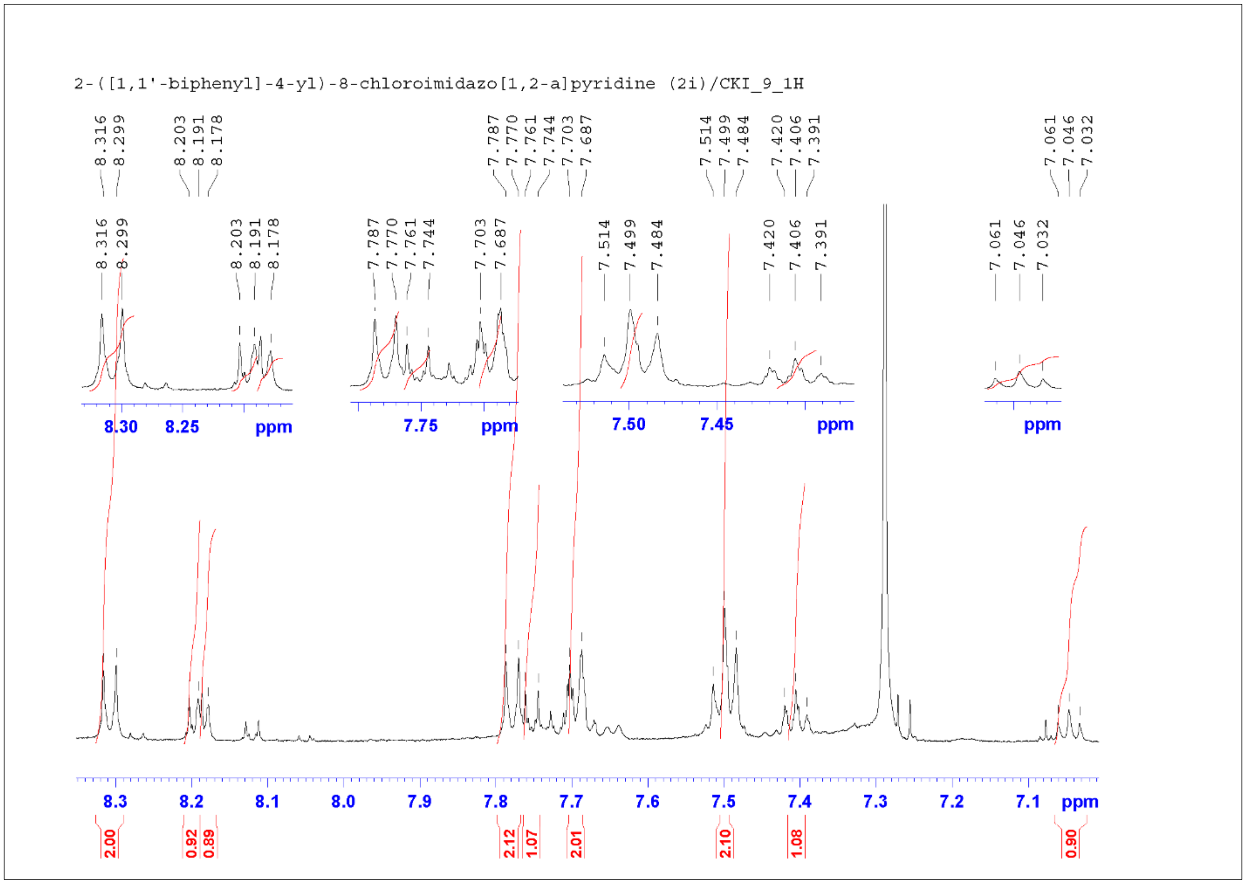

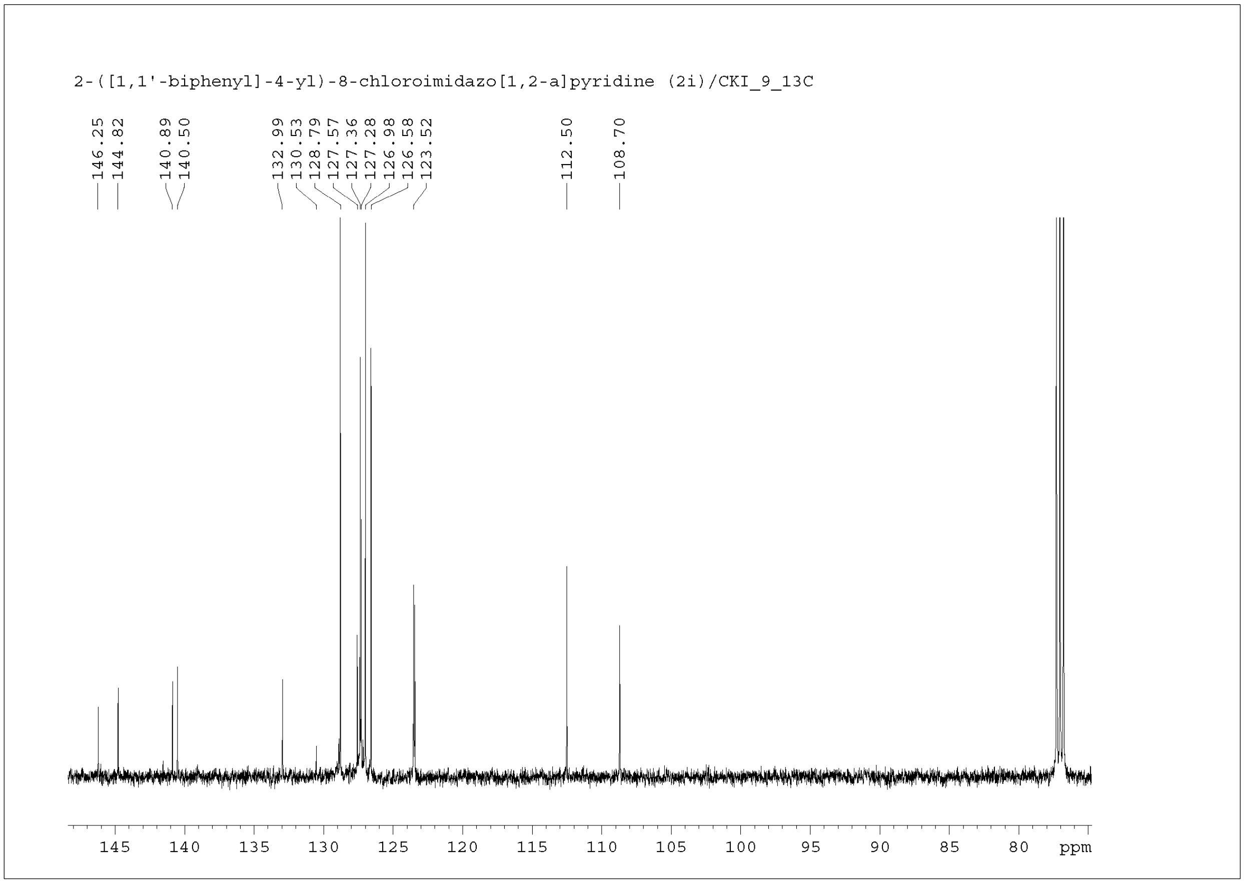

2-([1,1'-biphenyl]-4-yl)-8-chloroimidazo[1,2-a]pyridine (**2i**)/CKI_9_FTIR

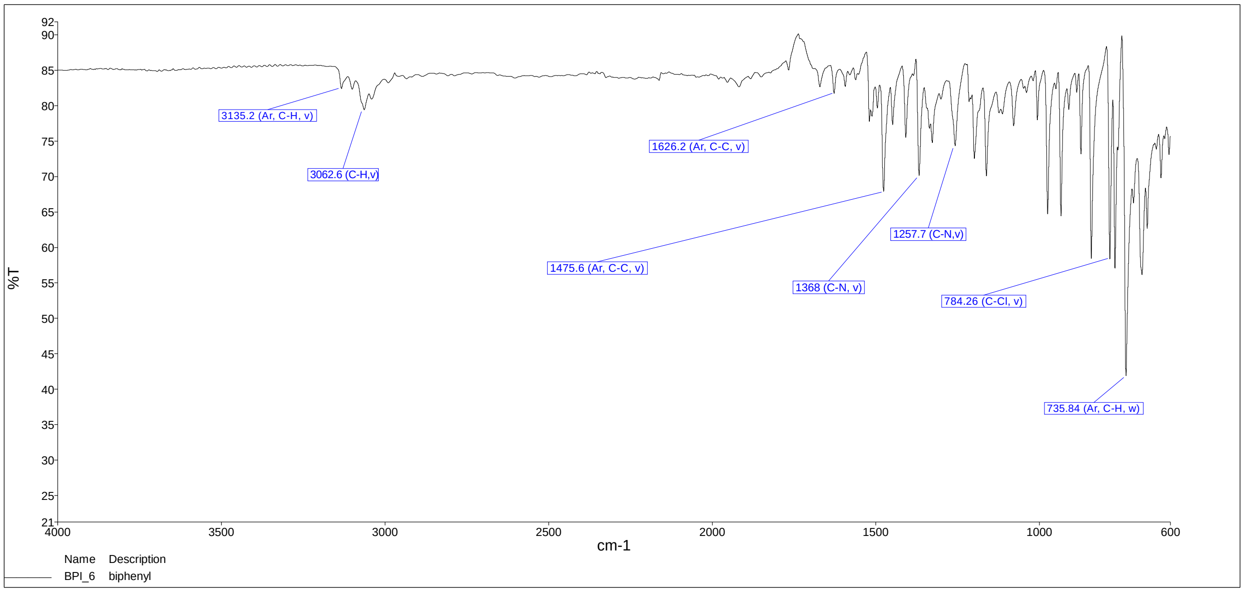

2-([1,1'-biphenyl]-4-yl)-8-chloroimidazo[1,2-a]pyridine (**2i**)/CKI_9_GCMS

```
File        :C:\Users\mphslab\Desktop\zh\20160511_B6(1) SPLITLESS.D
Operator    :
Acquired    : 11 May 2016  12:06     using AcqMethod PLANT EXTRACT SPLITLESS 20MINS.M
Instrument  :    5975C MSD
Sample Name:
Misc Info   :
Vial Number: 7
```

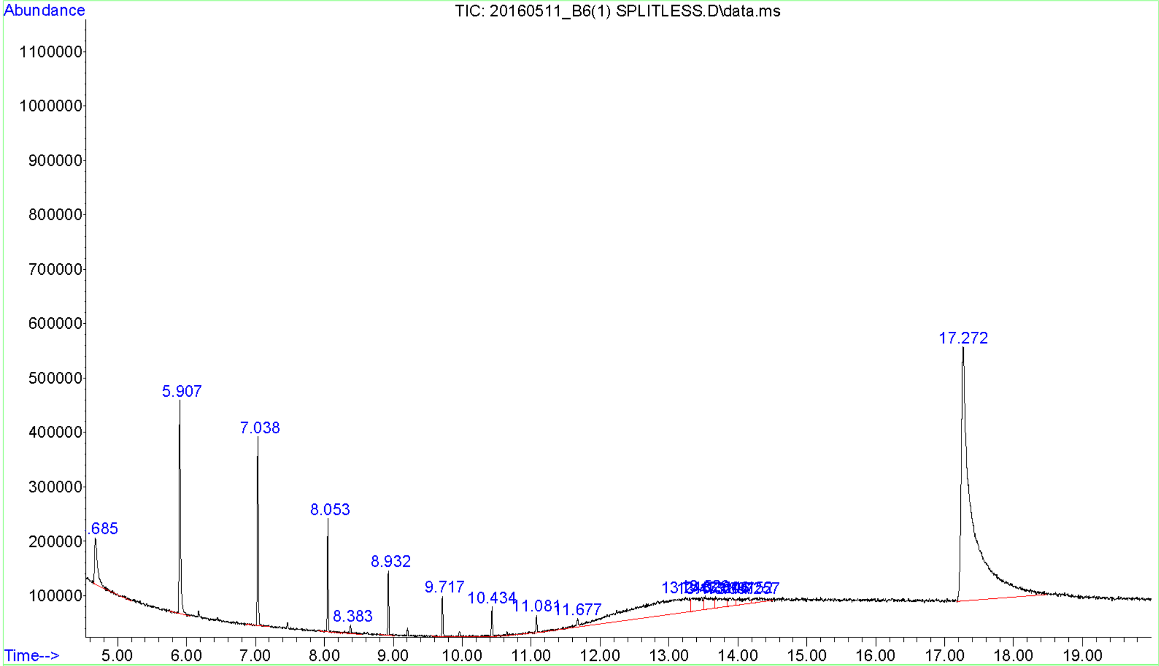

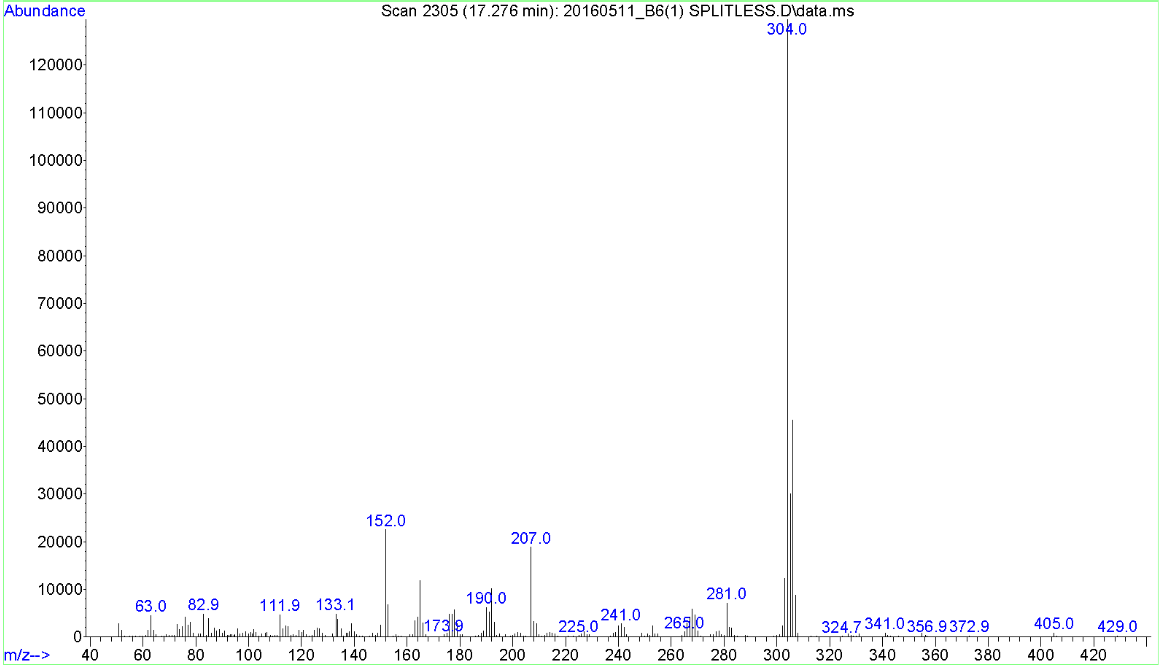

1.10 2-(3,4-dichlorophenyl)imidazo[1,2-a]pyridine (**2j**)

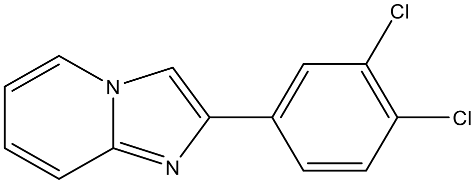

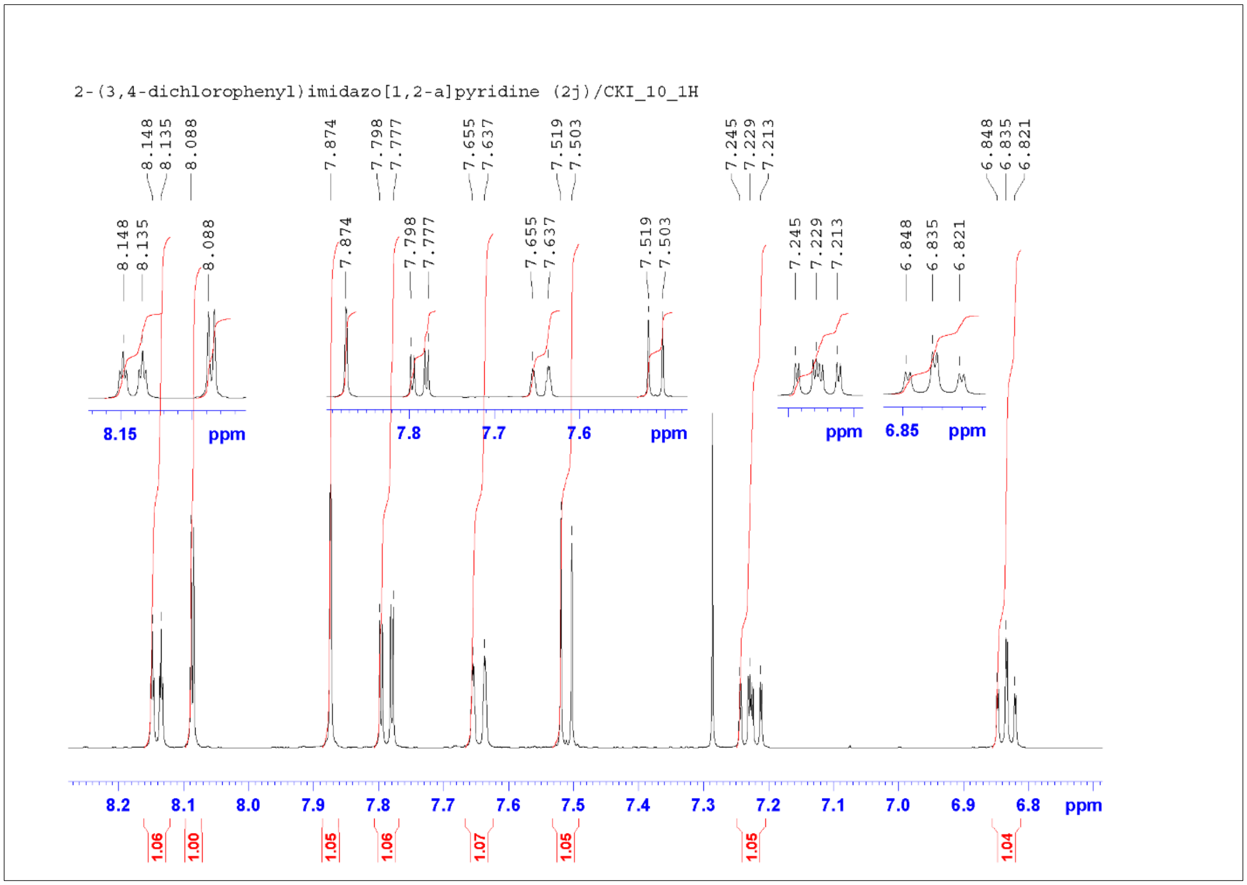

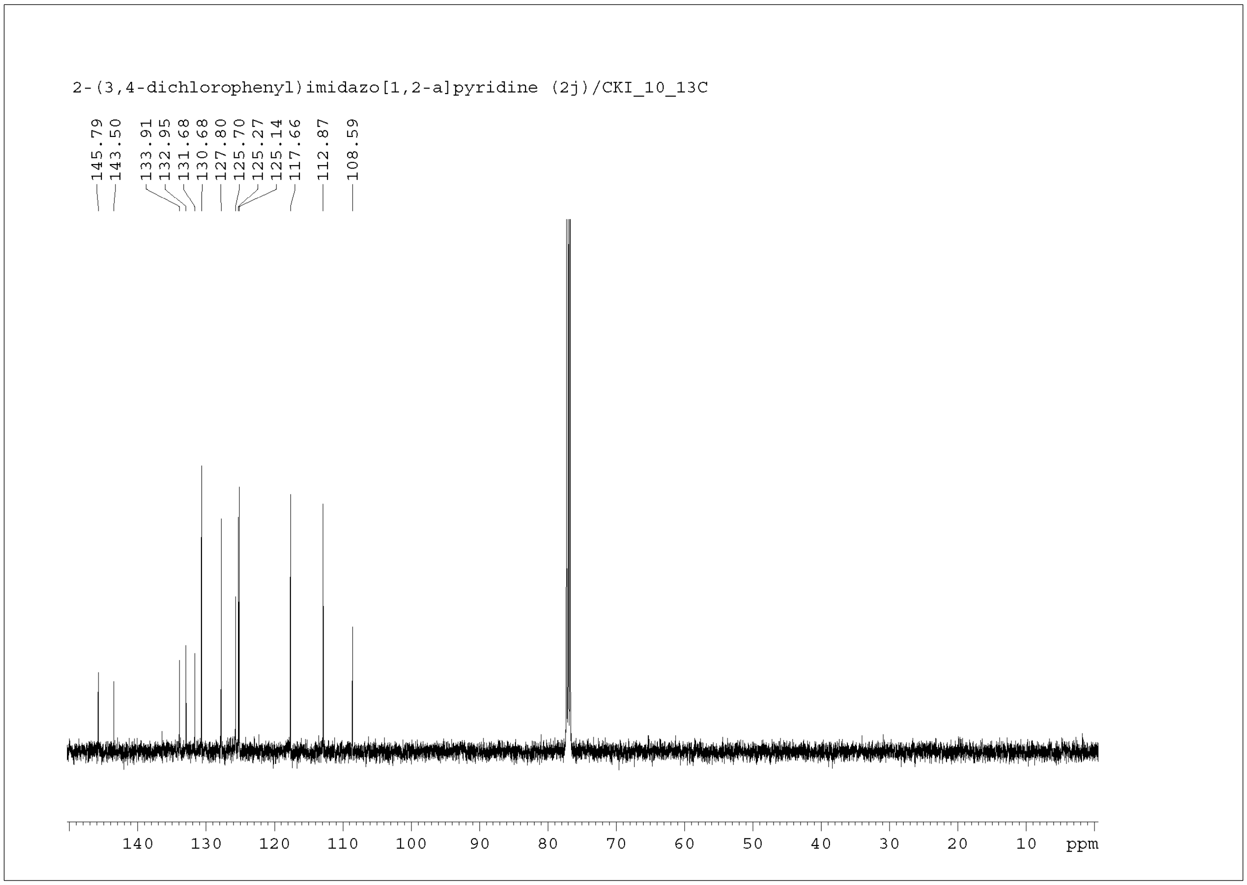

2-(3,4-dichlorophenyl)imidazo[1,2-a]pyridine (**2j**)/CKI_10_FTIR

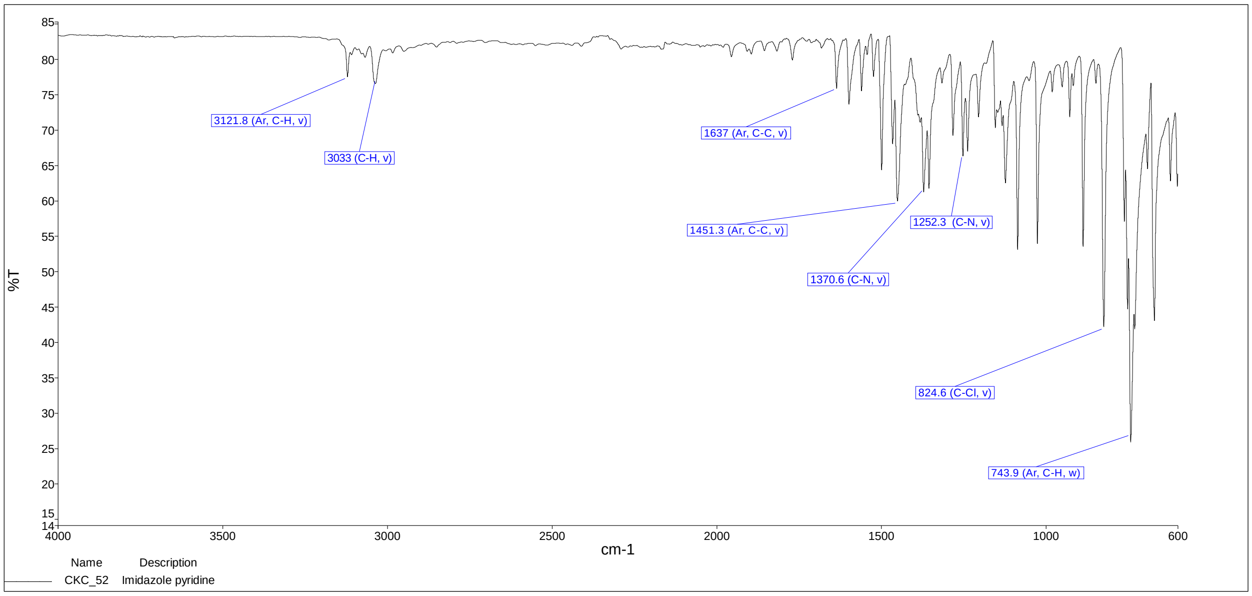

2-(3,4-dichlorophenyl)imidazo[1,2-a]pyridine (**2j**)/CKI_10_GCMS

```
File        :C:\Users\mphslab\Desktop\zh\20160407\20160407_C6.D
Operator    : ZH
Acquired    :  7 Apr 2016  12:18     using AcqMethod SYNTHESIS_SPLITRATIO100_1.M
Instrument  :    5975C MSD
Sample Name: 20160407 C6
Misc Info   :
Vial Number: 5
```

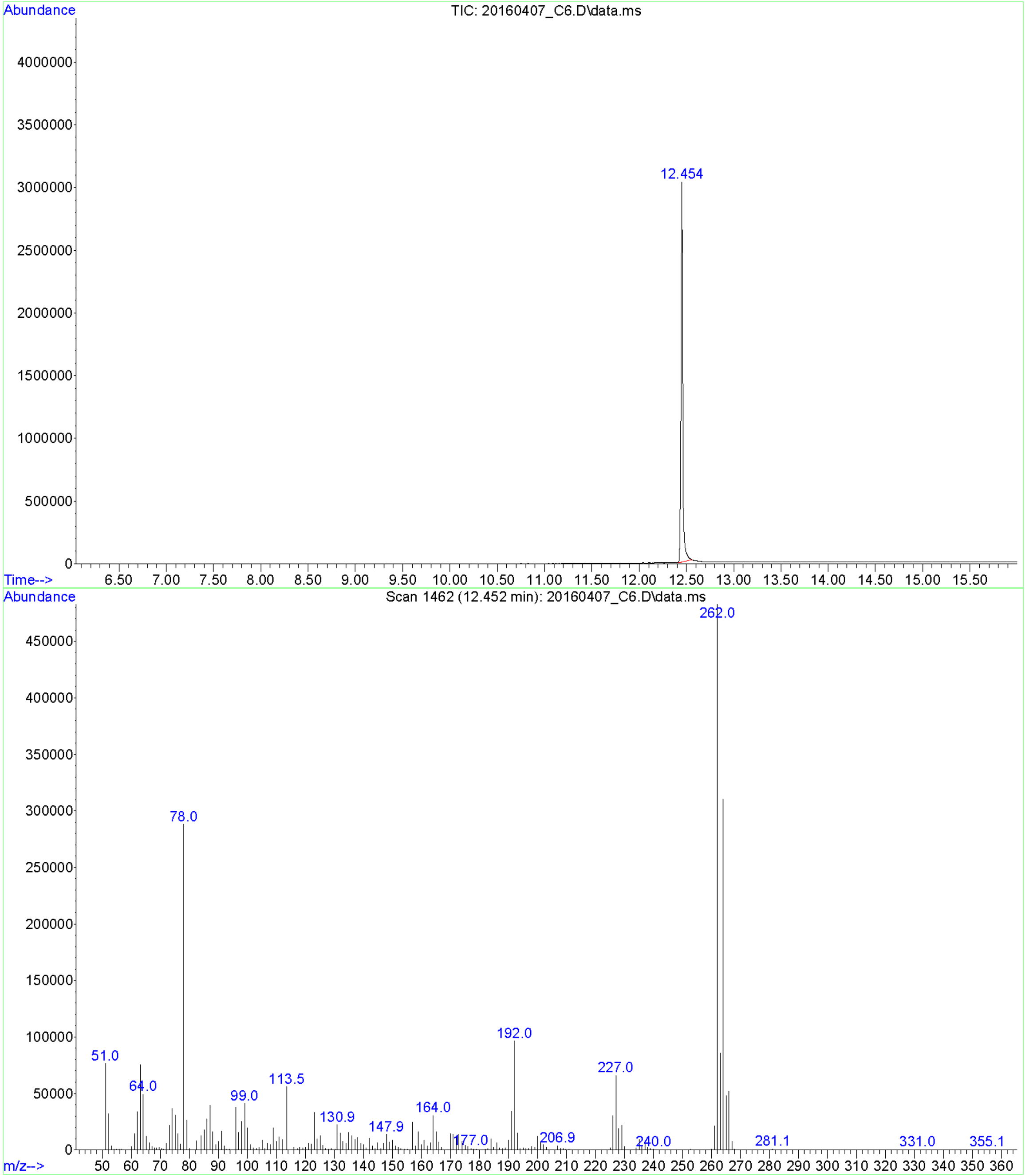

1.11 2-(3,4-dichlorophenyl)-8-methylimidazo[1,2-a]pyridine (**2k**)

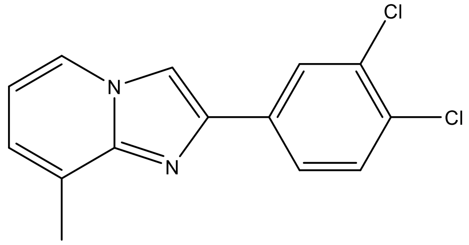

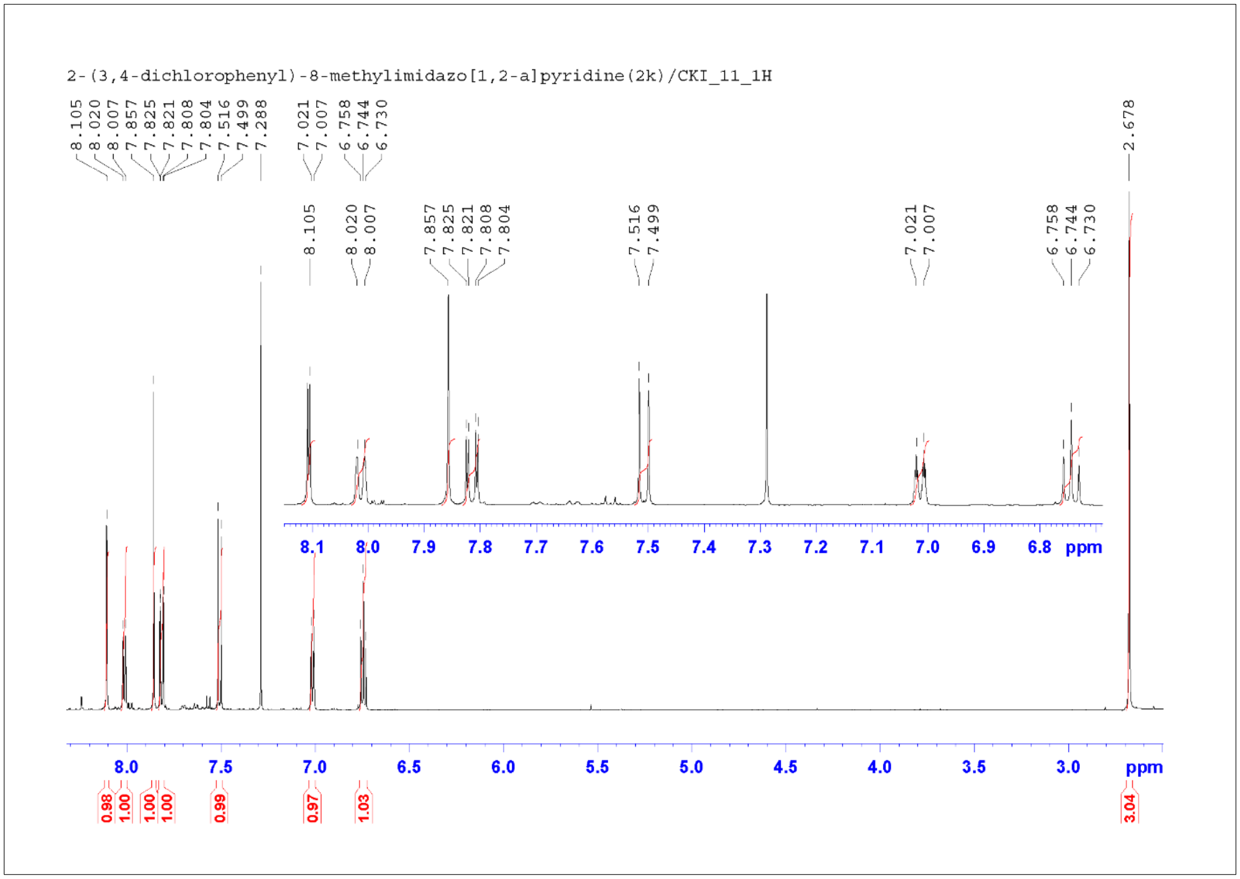

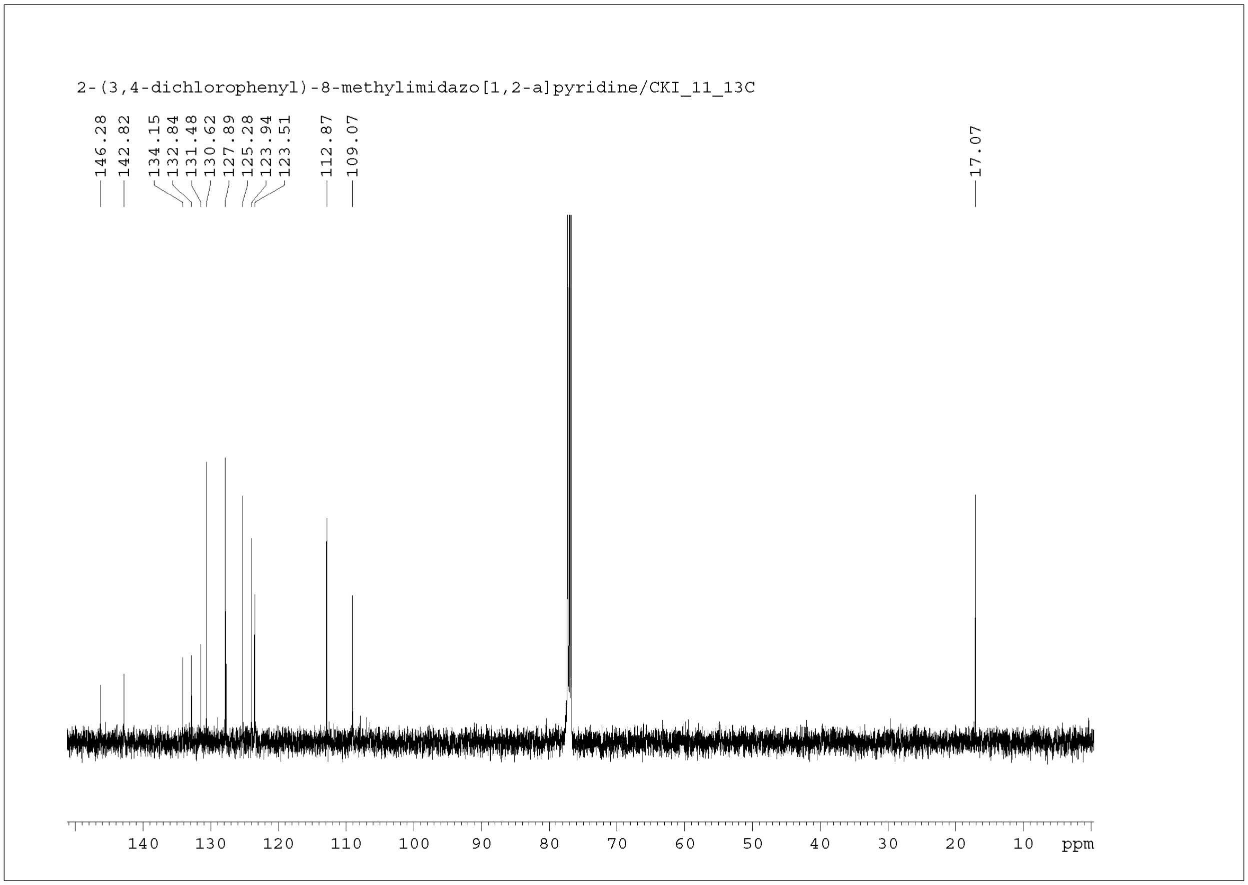

2-(3,4-dichlorophenyl)-8-methylimidazo[1,2-a]pyridine (**2k**)/CKI_11_FTIR

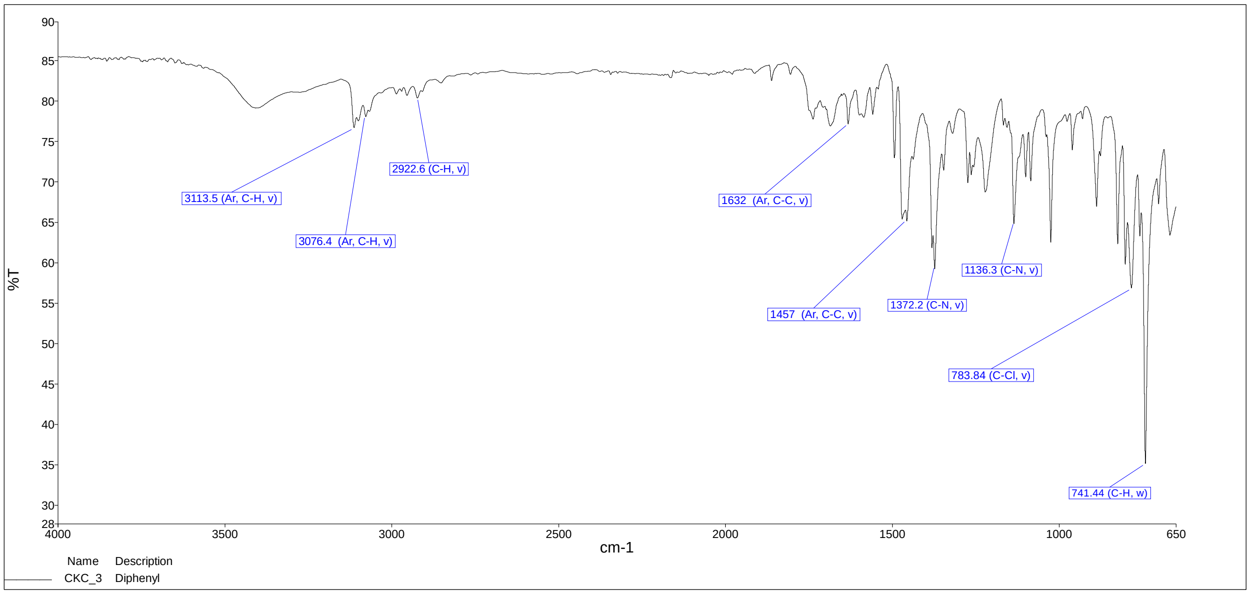

2-(3,4-dichlorophenyl)-8-methylimidazo[1,2-a]pyridine (**2k**)/CKI_11_GCMS

```
File        :C:\Users\mphslab\Desktop\zh\20160407\20160407_C3.D
Operator    : ZH
Acquired    :  7 Apr 2016  11:08     using AcqMethod SYNTHESIS_SPLITRATIO100_1.M
Instrument  :    5975C MSD
Sample Name: 20160407 C3
Misc Info   :
Vial Number: 2
```

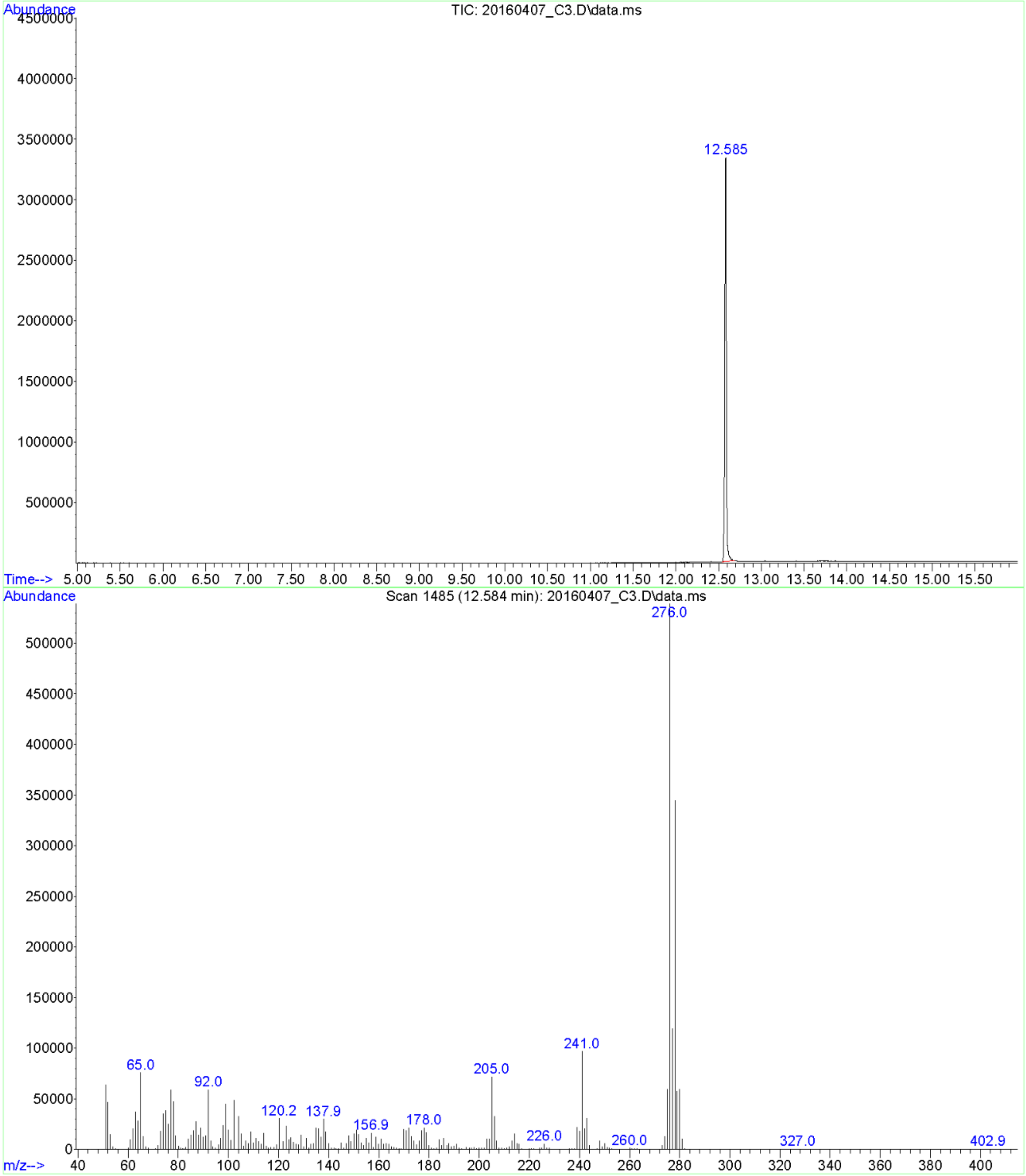

1.12 2-(4-methoxyphenyl)imidazo[1,2-a]pyridine (**2l**)

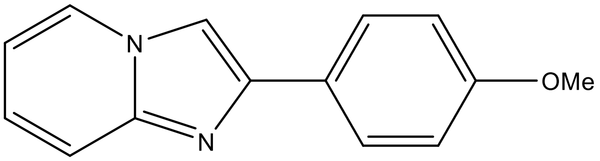

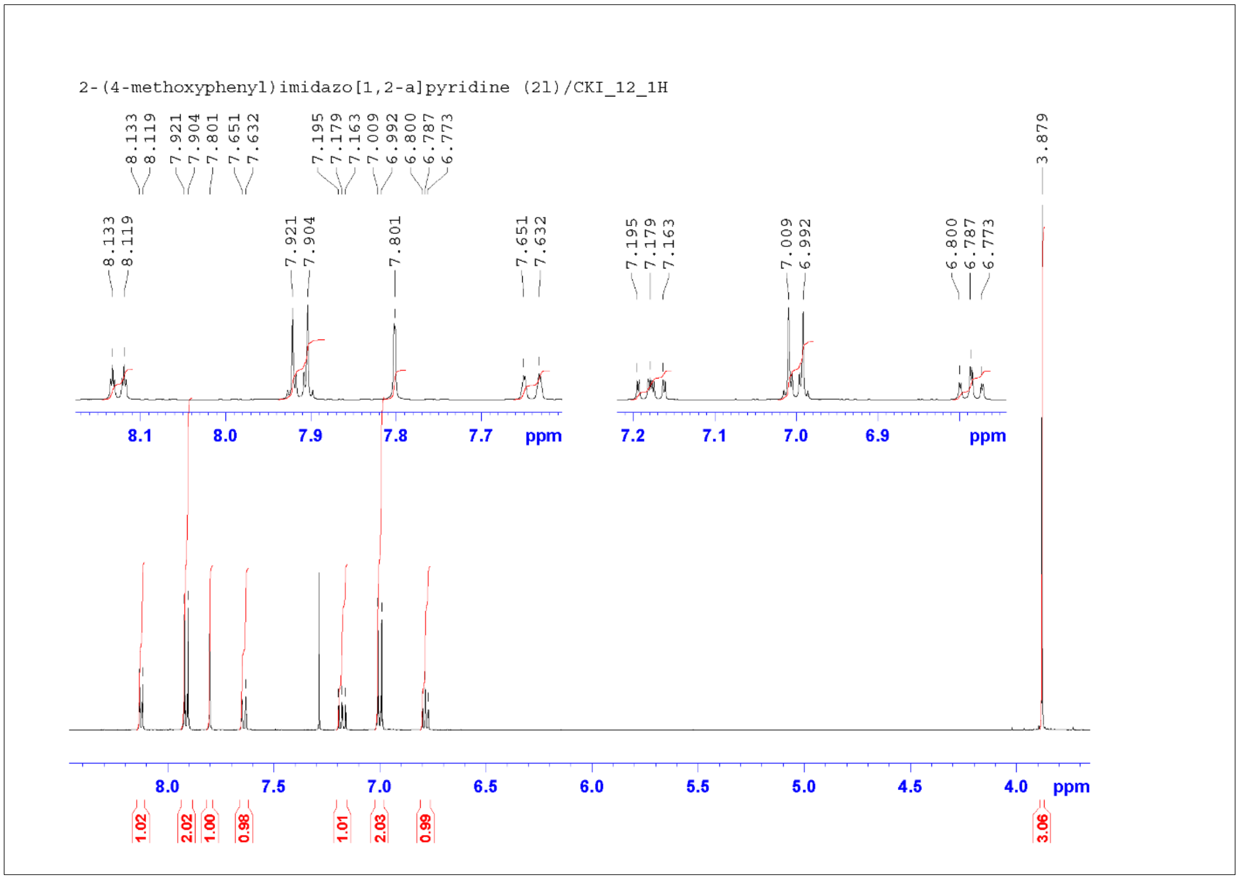

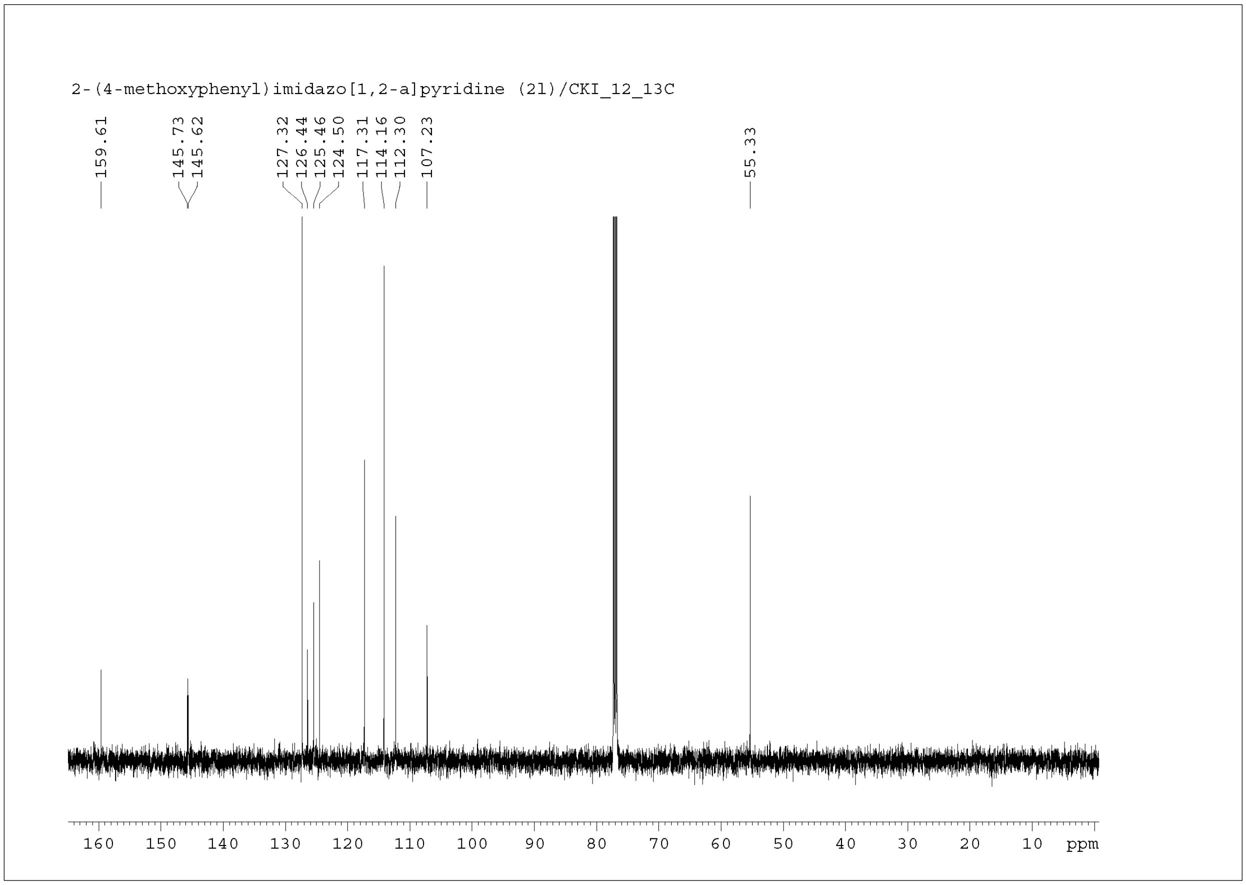

2-(4-methoxyphenyl)imidazo[1,2-a]pyridine (**2l**)/CKI_12_FTIR

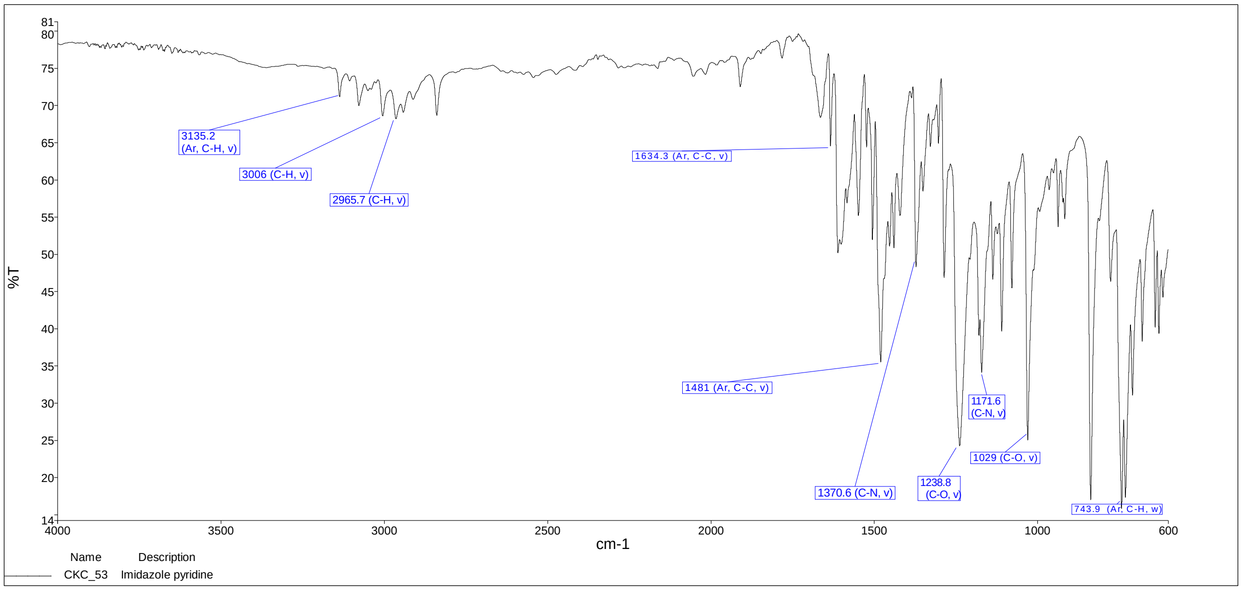

2-(4-methoxyphenyl)imidazo[1,2-a]pyridine (**2l**)/CKI_12_GCMS

```
File        :C:\Users\mphslab\Desktop\zh\20160407\20160407_C7.D
Operator    : ZH
Acquired    :  7 Apr 2016  12:41     using AcqMethod SYNTHESIS_SPLITRATIO100_1.M
Instrument  :    5975C MSD
Sample Name: 20160407 C7
Misc Info   :
Vial Number: 6
```

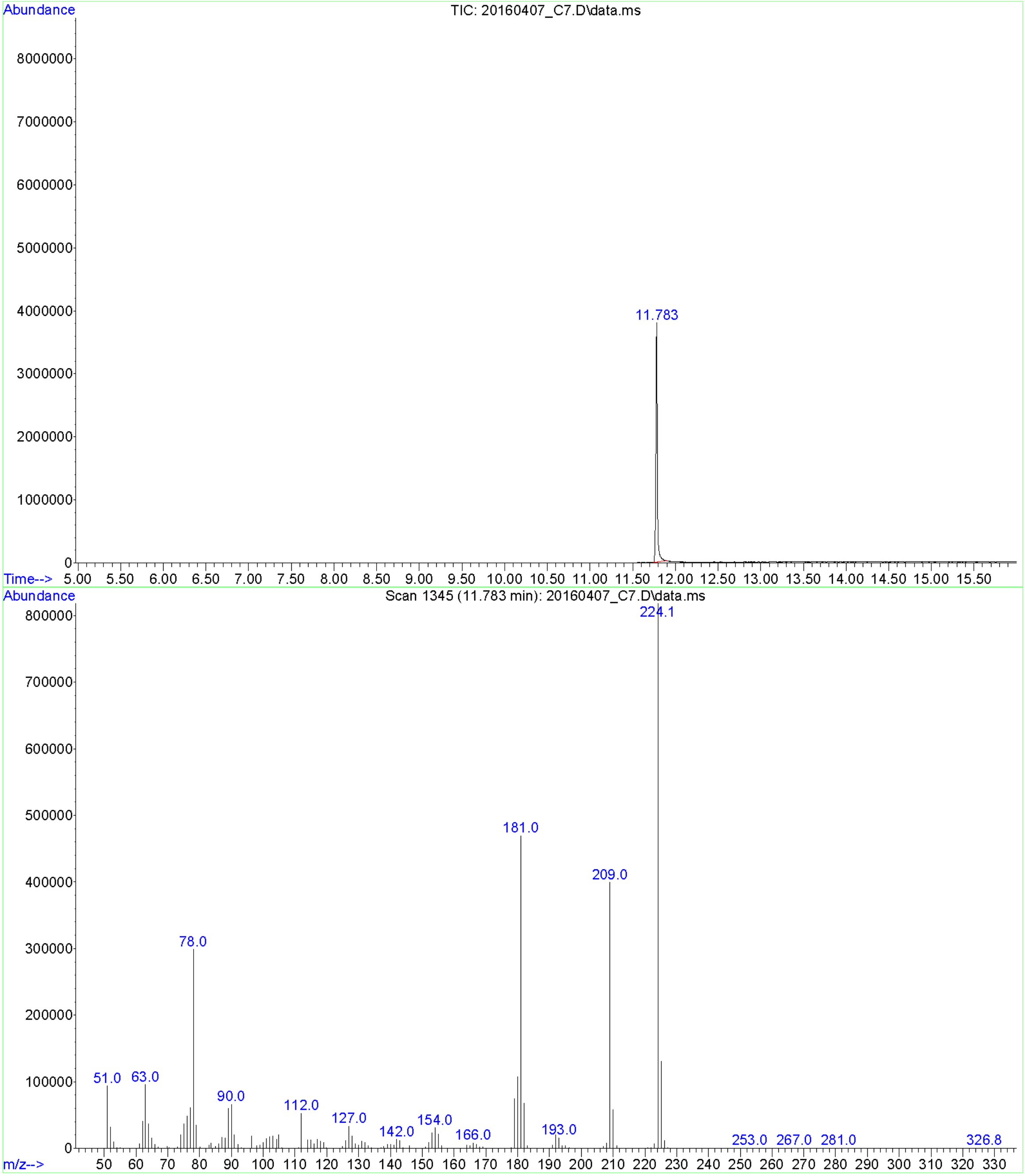

1.13 2-(4-methoxyphenyl)-8-methylimidazo[1,2-a]pyridine (**2m**)

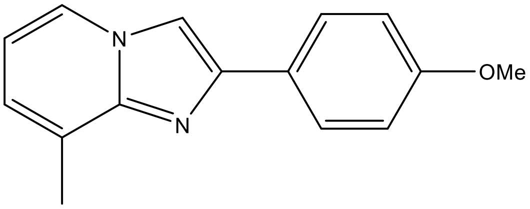

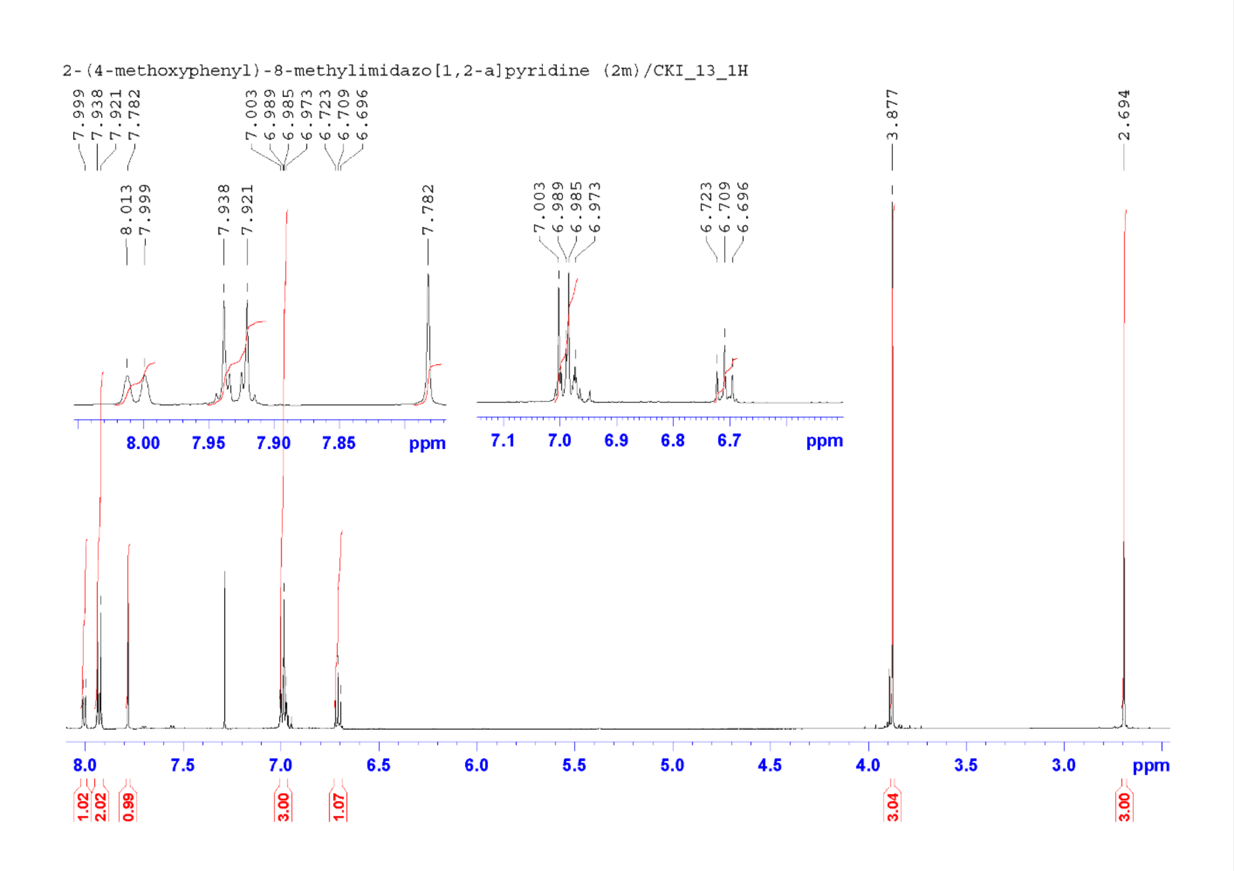

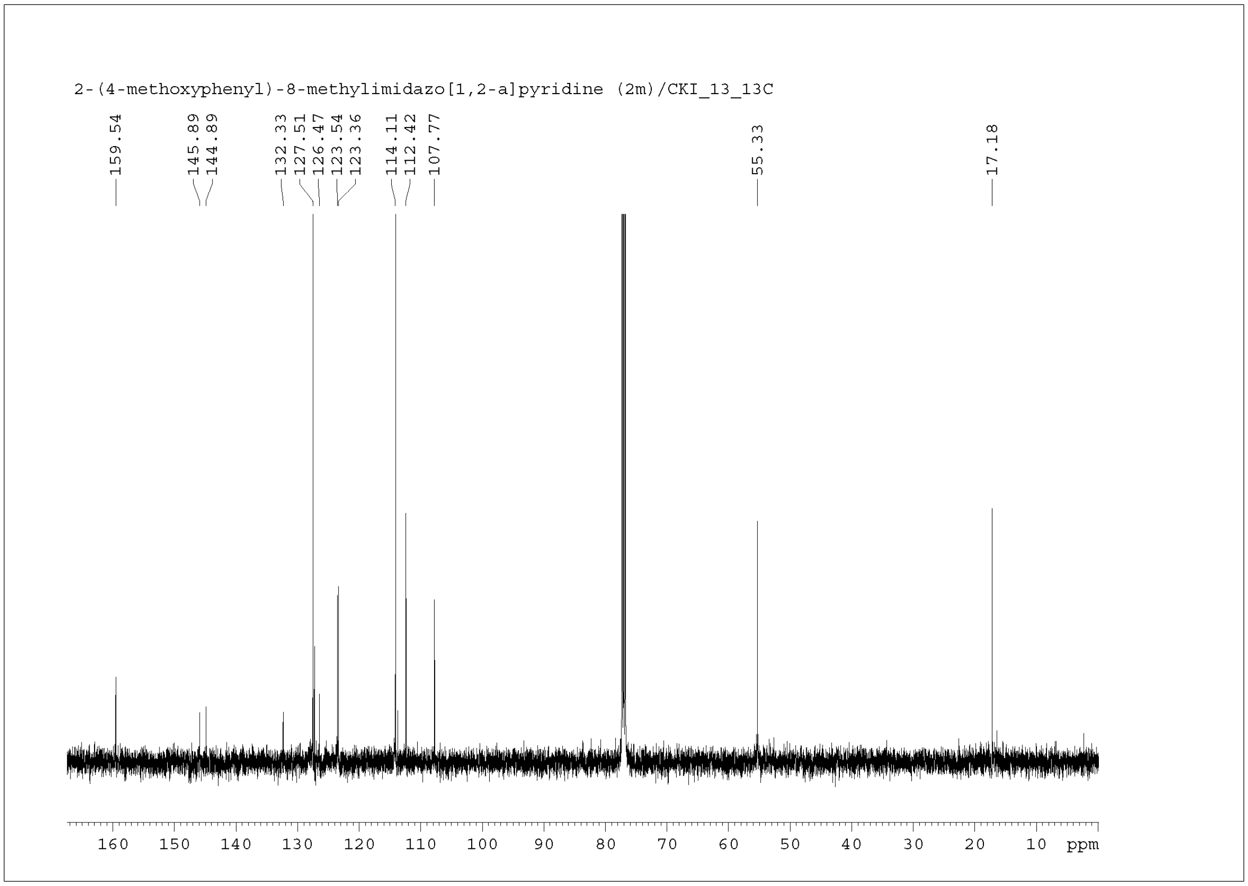

2-(4-methoxyphenyl)-8-methylimidazo[1,2-a]pyridine (**2m**)/CKI_13_FTIR

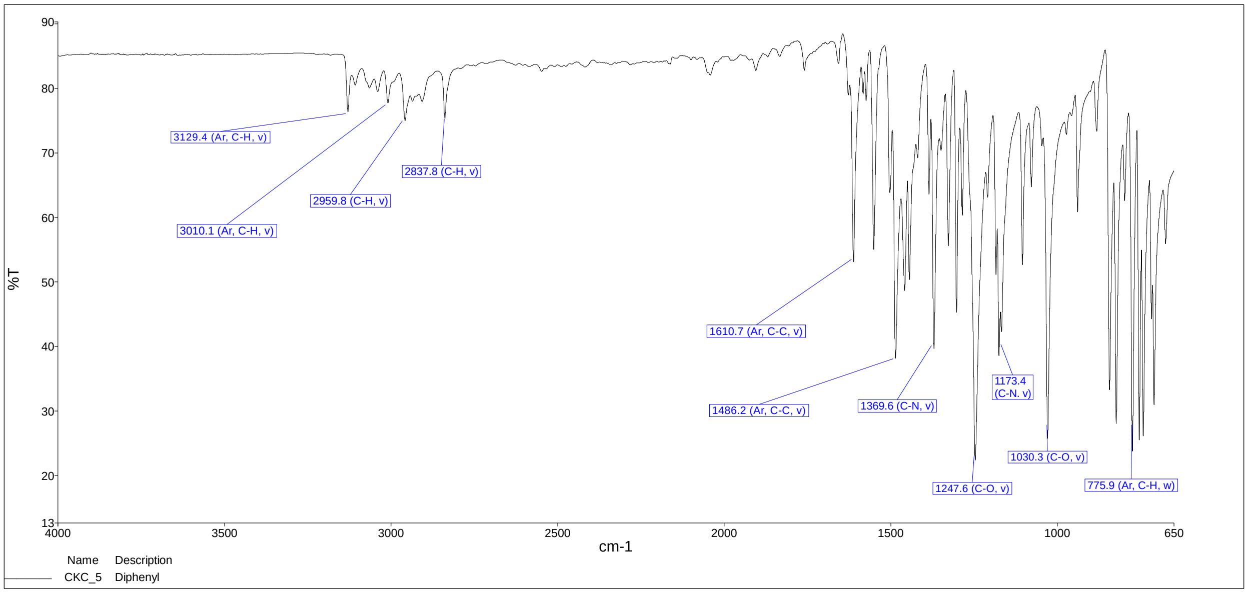

2-(4-methoxyphenyl)-8-methylimidazo[1,2-a]pyridine (**2m**)/CKI_13_GCMS

```
File        :C:\Users\mphslab\Desktop\zh\20160407\20160407_C5.D
Operator    : ZH
Acquired    :  7 Apr 2016  11:54      using AcqMethod SYNTHESIS_SPLITRATIO100_1.M
Instrument :    5975C MSD
Sample Name: 20160407 C5
Misc Info  :
Vial Number: 4
```

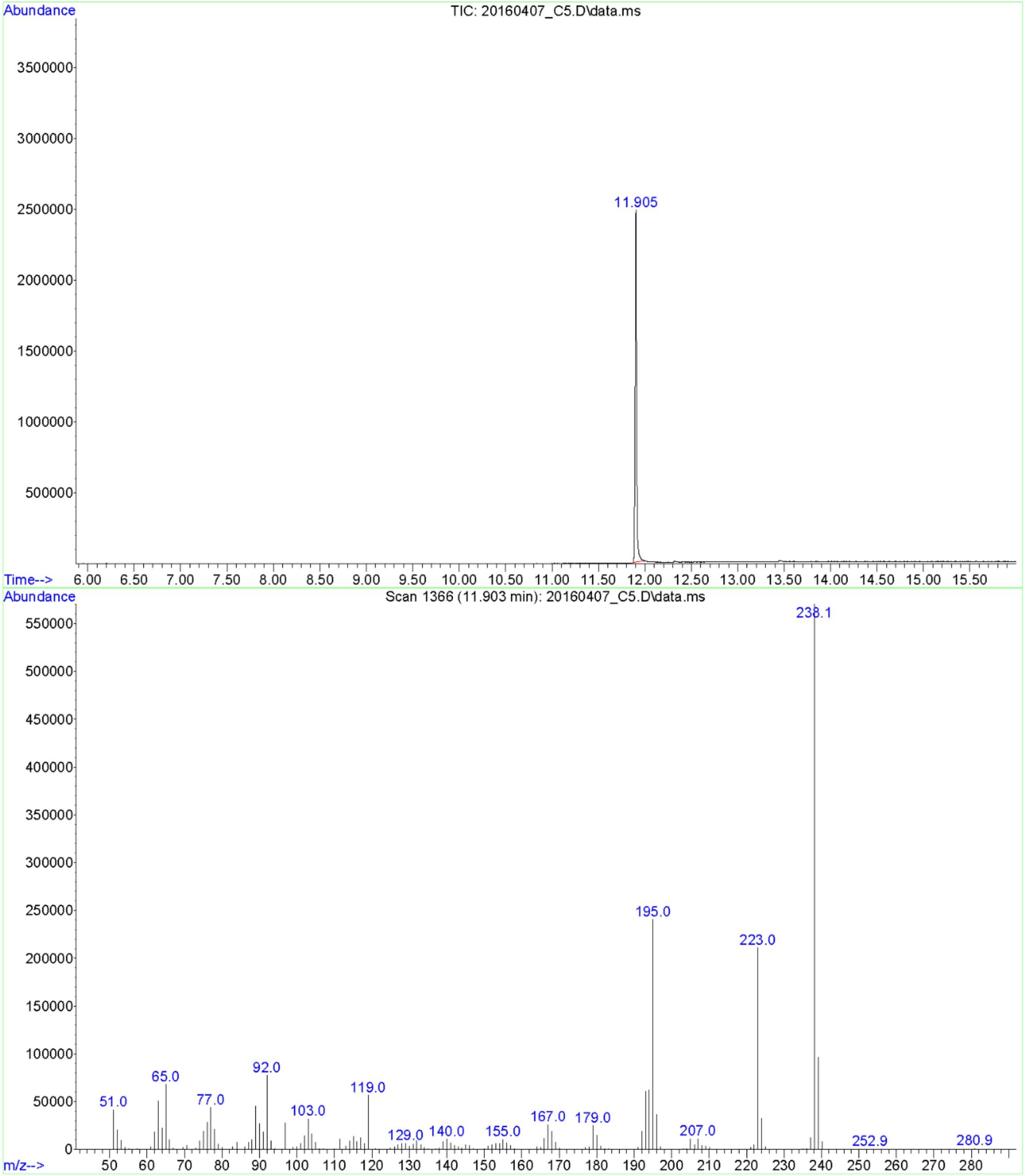

1.14 2-(4-chlorophenyl)-8-methylimidazo[1,2-a]pyridine (**2n**)

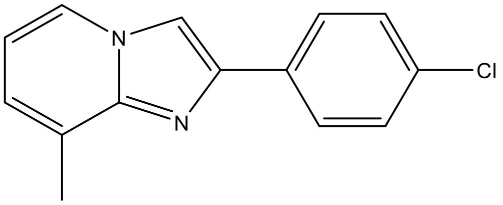

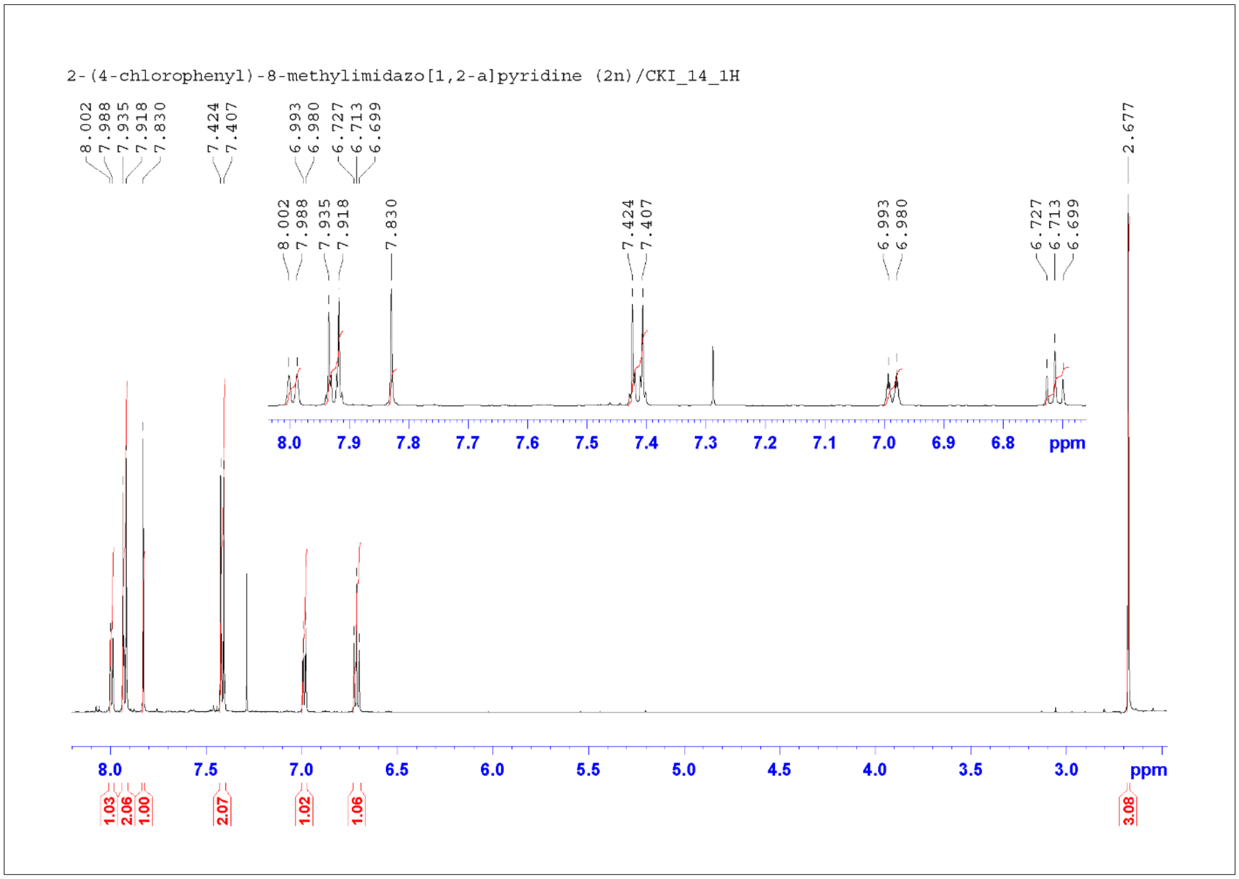

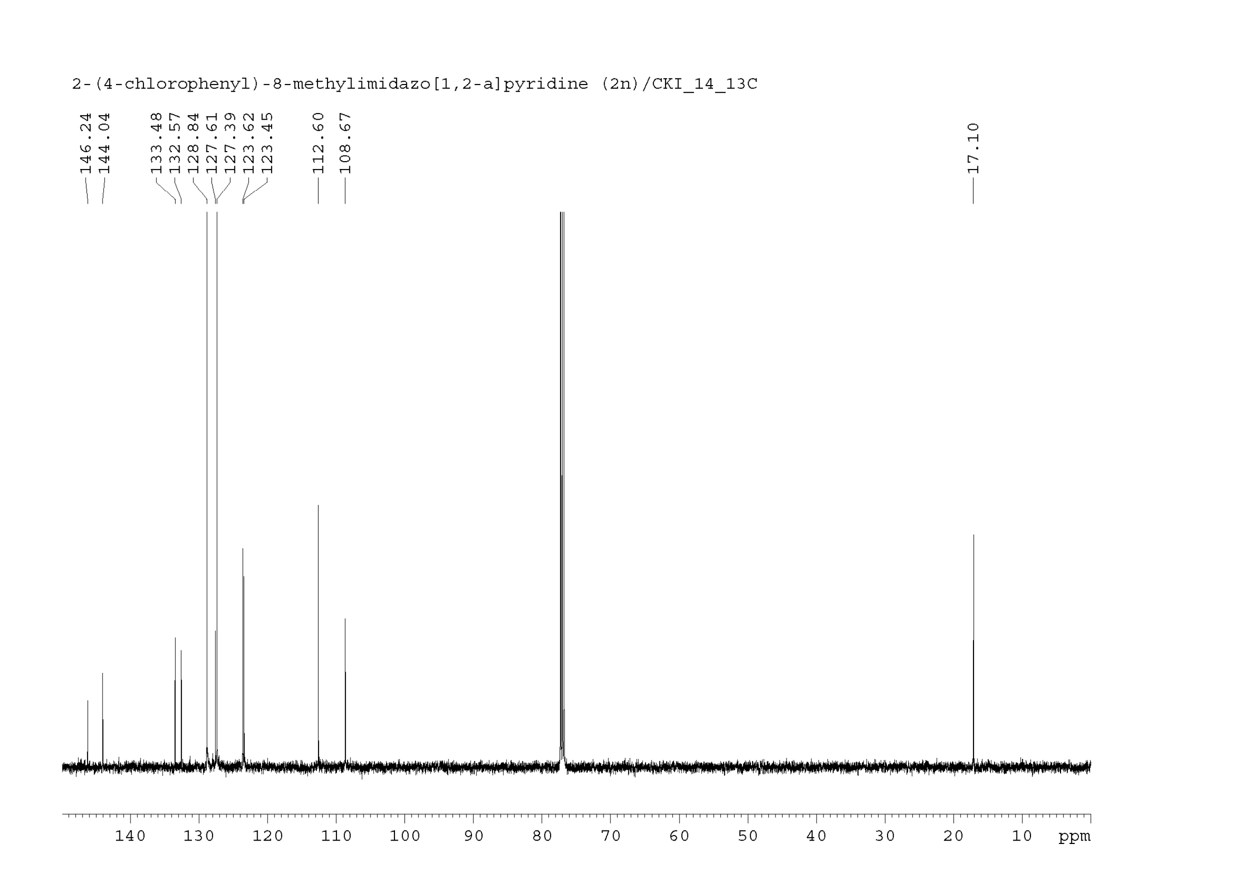

2-(4-chlorophenyl)-8-methylimidazo[1,2-a]pyridine (**2n**)/CKI_14_FTIR

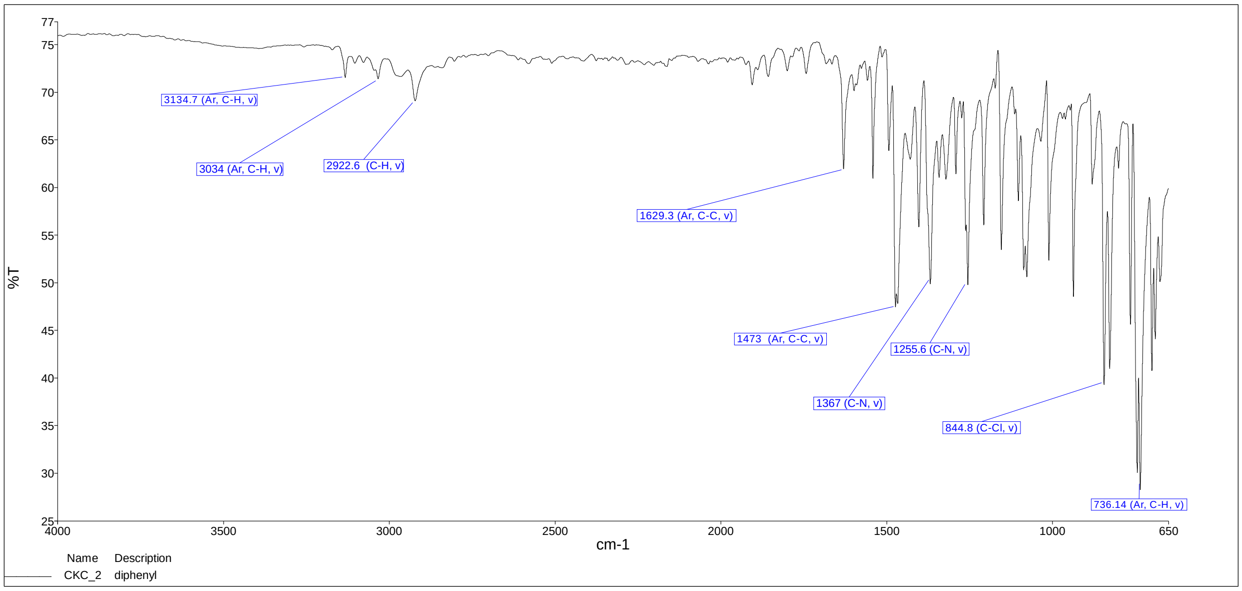

2-(4-chlorophenyl)-8-methylimidazo[1,2-a]pyridine (**2n**)/CKI_14_GCMS

```
File        :C:\Users\mphslab\Desktop\zh\20160407\20160407_C2.D
Operator    :
Acquired    :  7 Apr 2016  10:35     using AcqMethod SYNTHESIS_SPLITRATIO100_1.M
Instrument :     5975C MSD
Sample Name:
Misc Info   :
Vial Number: 1
```

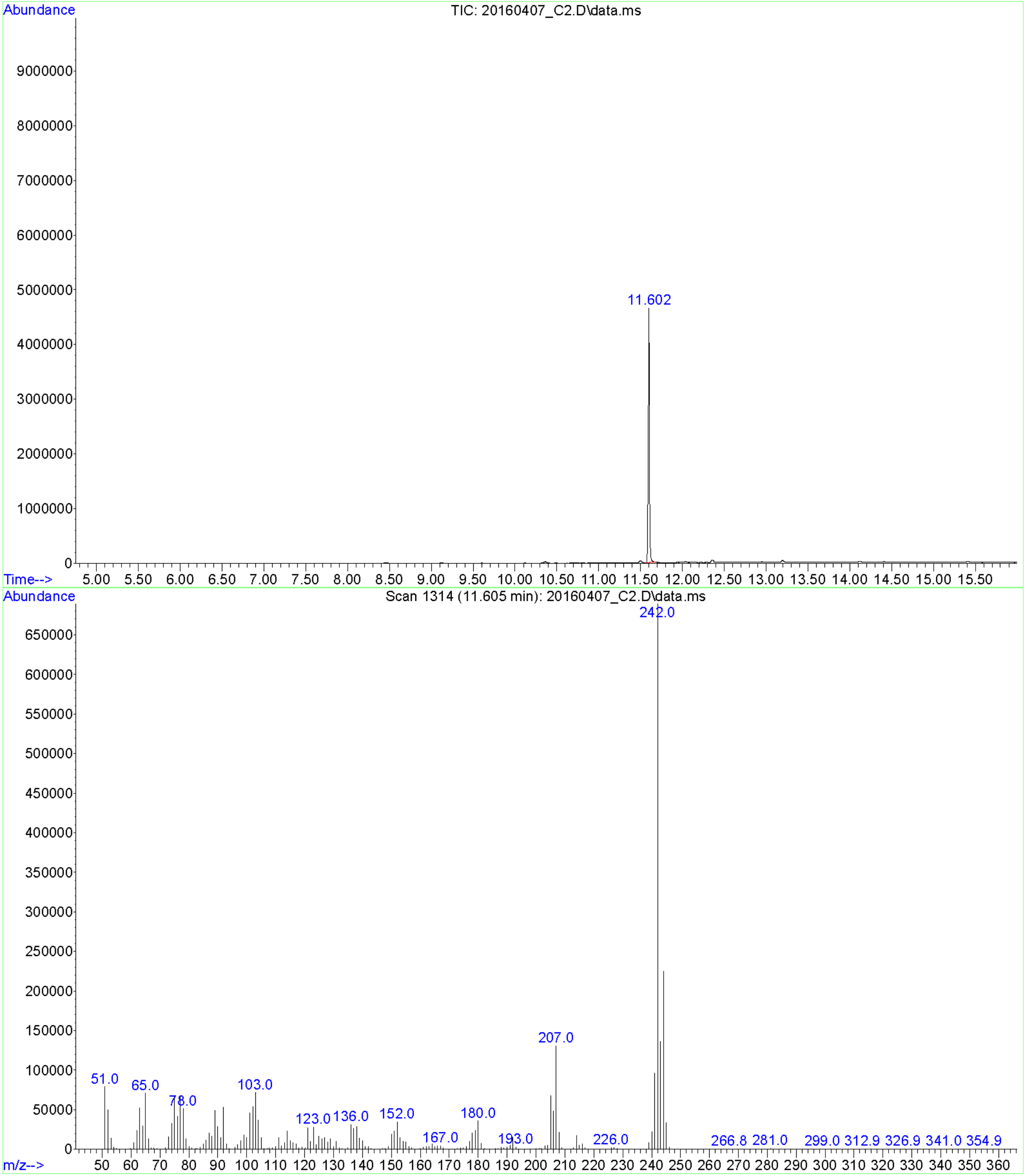

1.15 2-(4-bromophenyl)-8-methylimidazo[1,2-a]pyridine (**2o**)

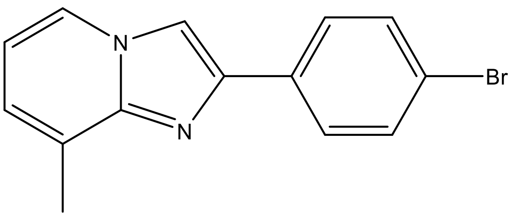

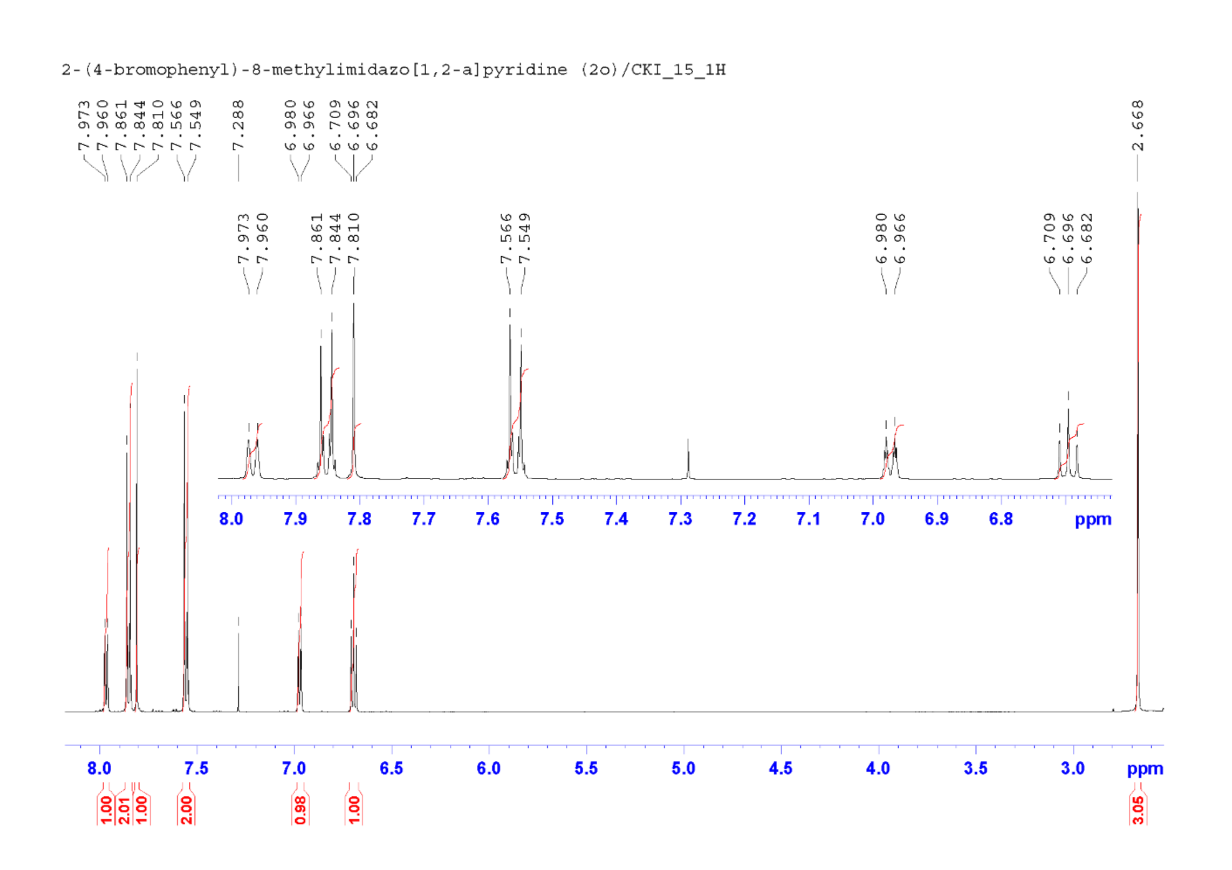

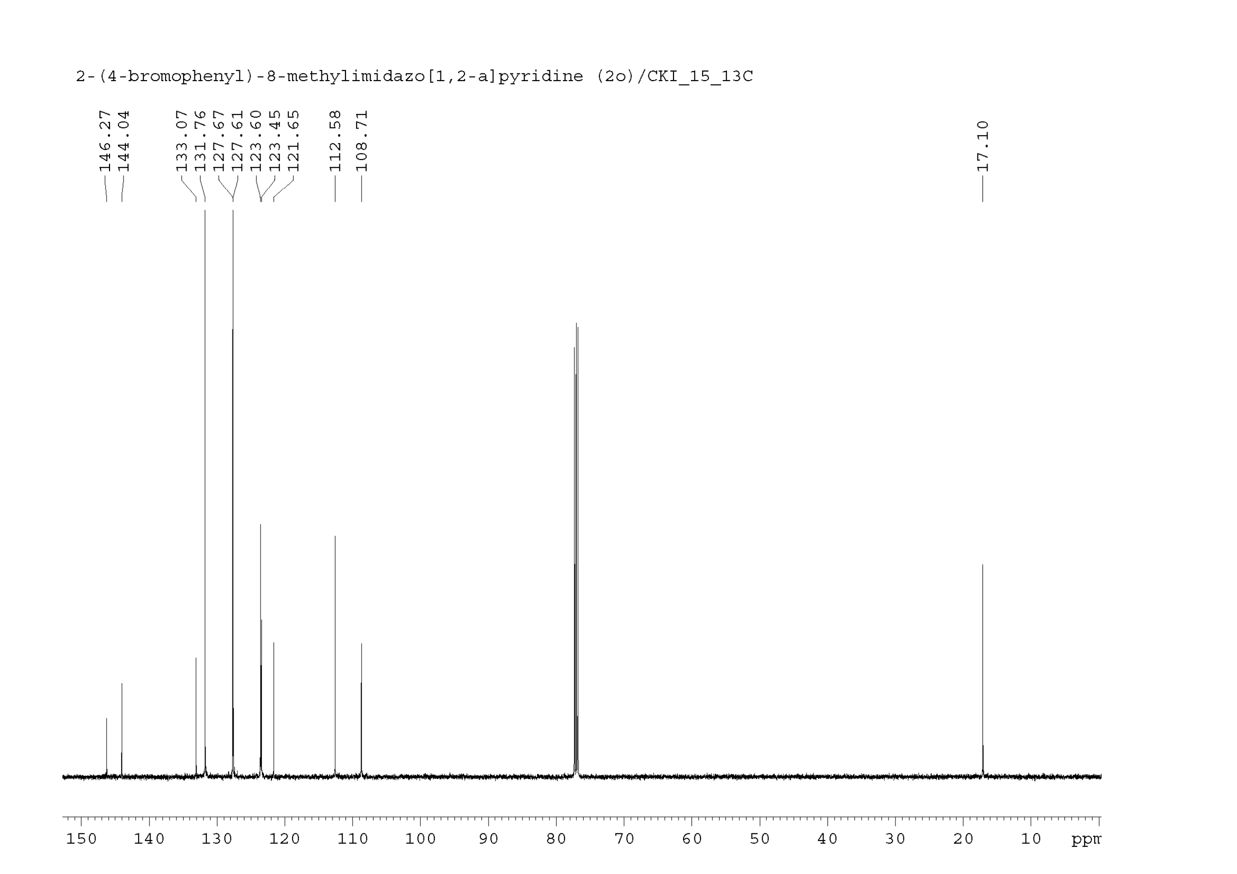

2-(4-bromophenyl)-8-methylimidazo[1,2-a]pyridine (**2o**)/CKI_15_FTIR

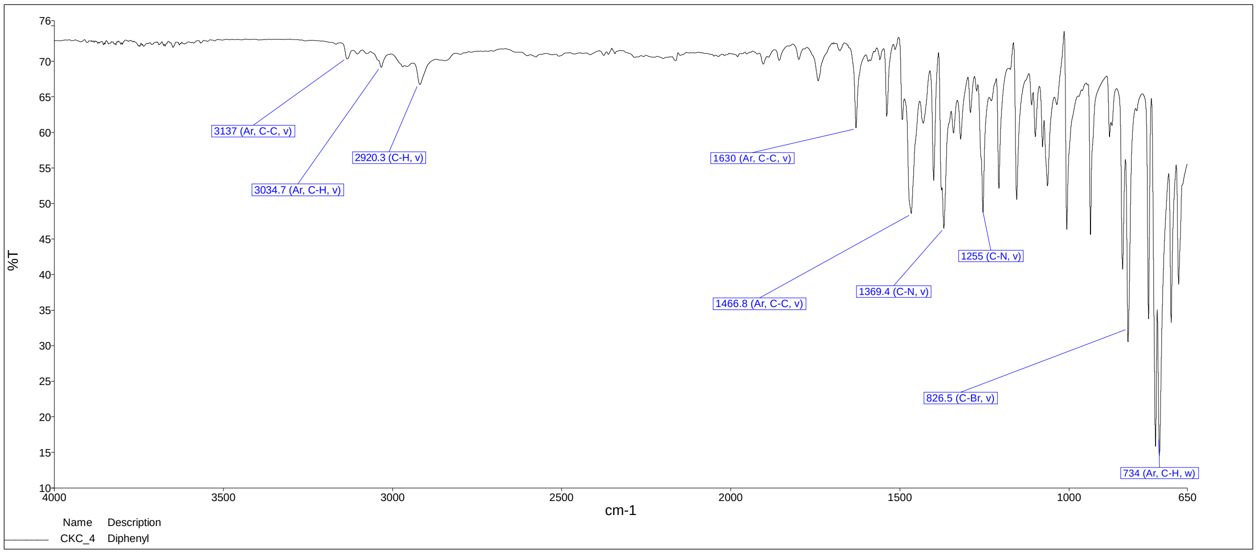

2-(4-bromophenyl)-8-methylimidazo[1,2-a]pyridine (**2o**)/CKI_15_GCMS

```
File        :C:\Users\mphslab\Desktop\zh\20160407\20160407_C4.D
Operator    : ZH
Acquired    :  7 Apr 2016  11:31     using AcqMethod SYNTHESIS_SPLITRATIO100_1.M
Instrument  :    5975C MSD
Sample Name: 20160407 C4
Misc Info   :
Vial Number: 3
```

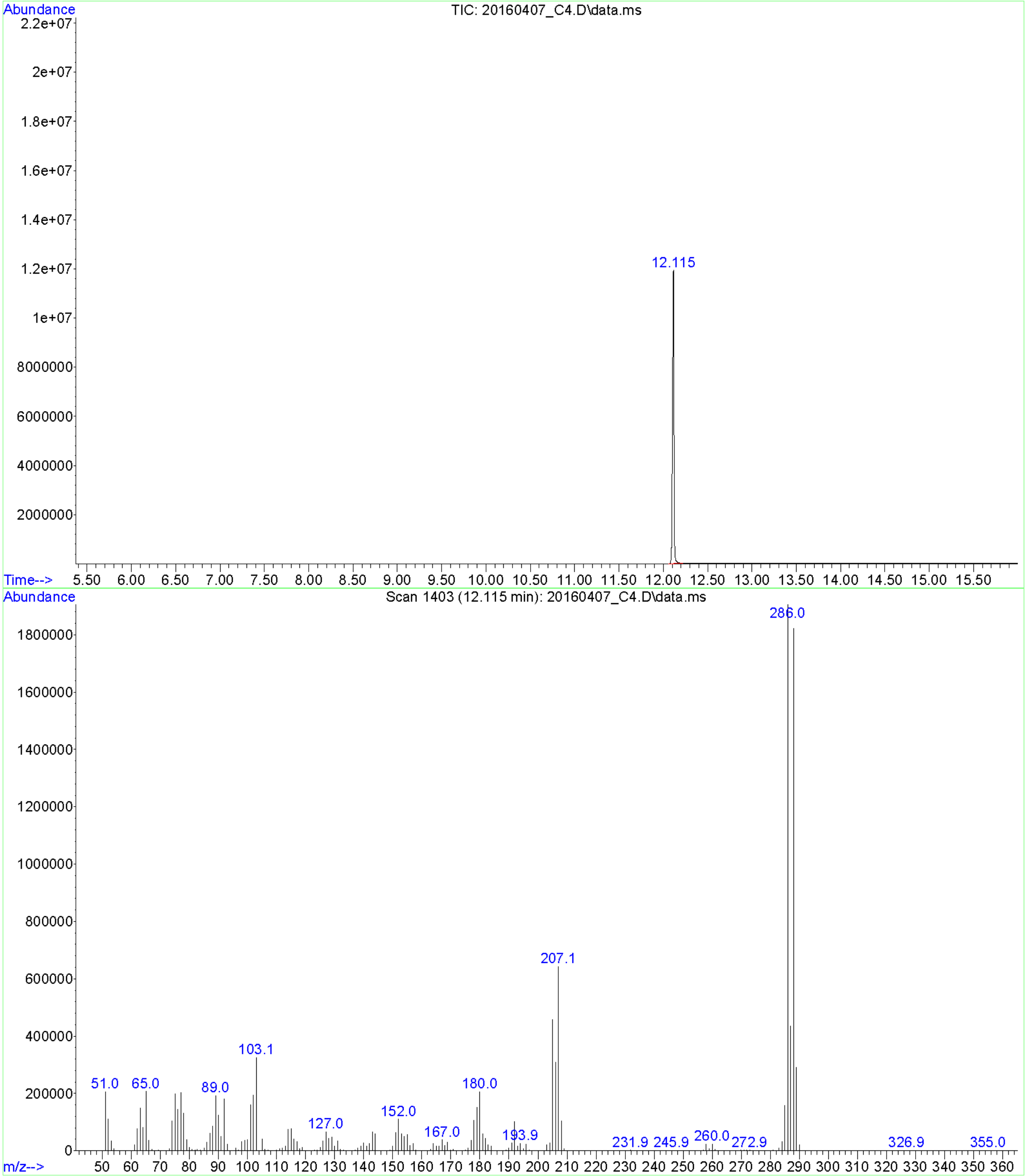

2.0 Single crystal X-ray crystallography data

2.1 Crystal data and parameters

Table 2.1: Crystal data and parameters for structure refinement for **2**(**a**-**c**, **e**-**h**)

Compound	**2a**	**2b**	**2c**	**2e**	**2f**	**2g**	**2h**
CCDC deposition numbers	1541130	1541132	1541131	1541134	1541133	1055984	1541135
Molecular formula	$C_{17}H_{20}N_2$	$C_{18}H_{23}N_2O$	$C_{36}H_{46}N_4O$	$C_{34}H_{39}Cl_2N_4O$	$C_{19}H_{14}N_2$	$C_{20}H_{16}N_2$	$C_{20}H_{16}N_2$
Molecular weight	252.35	283.38	550.77	590.59	270.32	284.35	284.35
Crystal system	Monoclinic	Monoclinic	Monoclinic	Monoclinic	Monoclinic	Orthorhombic	Monoclinic
Space group	*P21/c*	*P21/m*	*P21/c*	*P21/m*	*P21/c*	*Pna21*	*P*21/c
a (Å)	12.712 (2)	9.4502 (12)	12.290 (4)	14.7232 (17)	11.363 (4)	21.744 (7)	14.265 (2)
b (Å)	10.7467 (18)	6.7399 (8)	6.482 (2)	6.7471 (8)	5.6367 (16)	12.142 (4)	5.7143 (8)
c (Å)	11.2151 (19)	12.4416 (12)	18.898 (6)	15.5978 (18)	21.519 (6)	5.6932 (17)	17.846 (3)
α (°)	90	90	90	90	90	90	90
β (°)	116.211 (3)	103.486 (2)	101.656 (7)	109.087 (3)	94.307 (6)	90	92.620 (3)
γ (°)	90	90	90	90	90	90	90
V ($Å^3$)	1374.6 (4)	770.60 (15)	1474.4 (8)	1464.3 (3)	1374.4 (7)	1503.1 (8)	1453.2 (4)
Z	4	2	2	2	4	4	4
D_{calc} (g cm^{-3})	1.219	1.221	1.241	1.339	1.384	1.257	1.3
Crystal dimensions (mm)	0.70 × 0.38 × 0.07	0.56 × 0.49 × 0.08	0.27 × 0.15 × 0.10	0.37 × 0.05 × 0.03	0.66 × 0.19 × 0.10	0.24 × 0.23 × 0.10	0.72 × 0.21 × 0.07
μ (mm^{-1})	0.07	0.08	0.08	0.26	0.08	0.07	0.08
T_{min}/T_{max}	0.793/0.968	0.703/0.960	0.679/0.991	0.878/0.961	0.842/0.967	0.844/0.992	0.878/0.971
Reflections measured	14470	7019	11384	33440	9623	7699	11496
Ranges/indices (*h*, *k*, *l*)	−17→17; −12→14; −15→15	−10→12; −8→8; −16→16	−15→15; −7→7; −23→23	−17→17; −8→8; −18→18	−13→13; −6→6; −26→25	−28→22; −15→13; −7→7	−17→17; −7→7; −22→22
θ limit (°)	1.9-29.1	1.7-27.5	1.7-26.0	1.4-25.2	1.8-25.6	1.9-27.4	1.4-26.6
Unique reflections	3672	1910	2887	2863	2560	3259	3025
Observed reflections [$I > 2\sigma(I)$]	2402	1302	1614	1635	1724	1335	1657
Parameters	174	121	191	233	190	200	200
Restraints	0	0	0	0	0	1	0
Goodness of fit on F^2	1.05	1.07	1.04	1.03	1.00	0.95	1.10
R_1, wR_2 [$I \geq 2\sigma(I)$]	0.066, 0.216	0.052, 0.163	0.073, 0.231	0.056, 0.181	0.050, 0.199	0.054, 0.162	0.049, 0.171

Table 2.2: Crystal data and parameters for structure refinement for **2**(**i-o**)

Compound	**2i**	**2j**	**2k**	**2l**	**2m**	**2n**	**2o**
CCDC deposition numbers	1541136	1541137	1055986	1541138	1055988	1055985	1055987
Molecular formula	$C_{19}H_{13}ClN_2$	$C_{13}H_8Cl_2N_2$	$C_{14}H_{10}Cl_2N_2$	$C_{14}H_{12}N_2O$	$C_{15}H_{14}N_2O$	$C_{14}H_{11}ClN_2$	$C_{14}H_{11}BrN_2$
Molecular weight	304.76	263.11	277.14	224.26	238.28	242.7	287.16
Crystal system	Orthorhombi c	Monoclinic	Monoclinic	Orthorhombic	Orthorhombic	Monoclinic	Monoclinic
Space group	*Pna21*	*P21/c*	*P*21/c	*Pna*2$_1$	*Pna*2$_1$	*P*21/n	*P21/n*
a (Å)	21.712 (3)	11.8803 (14)	13.5437 (10)	6.3045 (9)	16.985 (5)	14.351 (2)	13.942 (2)
b (Å)	12.1243 (18)	5.6138 (7)	21.9519 (17)	7.0789 (10)	12.860 (4)	6.9258 (9)	6.9633 (11)
c (Å)	5.6485 (9)	17.853 (2)	13.8853 (11)	24.459 (3)	5.8397 (19)	24.557 (3)	24.747 (4)
α (°)	90	90	90	90	90	90	90
β (°)	90	104.890 (2)	114.3697 (14)	90	90	103.512 (3)	103.570 (2)
γ (°)	90	90	90	90	90	90	90
V (Å^3)	1486.9 (4)	1150.7 (2)	3760.4 (5)	1091.6 (3)	1275.5 (7)	2373.2 (6)	2335.5 (6)
Z	4	4	12	4	4	8	8
D_{calc} (g cm^{-3})	1.361	1.519	1.469	1.365	1.241	1.359	1.633
Crystal dimensions (mm)	0.78 × 0.13 × 0.05	0.33 × 0.24 × 0.08	0.31 × 0.21 × 0.21	0.35 × 0.24 × 0.17	0.61 × 0.11 × 0.06	0.50 × 0.44 × 0.07	0.47 × 0.45 × 0.36
μ (mm^{-1})	0.25	0.54	0.5	0.09	0.08	0.30	3.5
T_{min}/T_{max}	0.863/0.924	0.733/0.834	0.826/0.905	0.881/0.960	0.781/0.995	0.694/0.872	0.060/0.110
Reflections measured	12275	10230	38702	7969	6174	18939	21595
Ranges/indices (*h*, *k*, *l*)	−29→29; −16→13; −7→7	−13→15; −7→7; −23→22	−18→18; −30→29; −18→18	−8→8; −9→9; −30→32	−21→20; −16→13; −7→7	−17→17; −8→8; −30→30	−19→15; −9→9; −32→33
θ limit (°)	1.9-29.1	1.8-27.5	1.90-29.1	1.7-28.1	2.0-27.5	1.5-26.0	3.1-29.2
Unique reflections	3955	2640	9999	2652	2791	4656	6286
Observed reflections [*I* > 2σ(*I*)]	2190	1938	5537	2308	1184	2722	4652
Parameters	199	154	495	155	166	309	309
Restraints	1	0	1	1	1	0	0
Goodness of fit on F^2	1.01	1.02	1.01	1.05	0.96	1.04	1.04
R_1, wR_2 [$I \geq 2\sigma(I)$]	0.049, 0.133	0.041, 0.128	0.051, 0.177	0.039, 0.085	0.059, 0.189	0.064, 0.206	0.048, 0.132

2.3 Ortep diagram and atom numbering

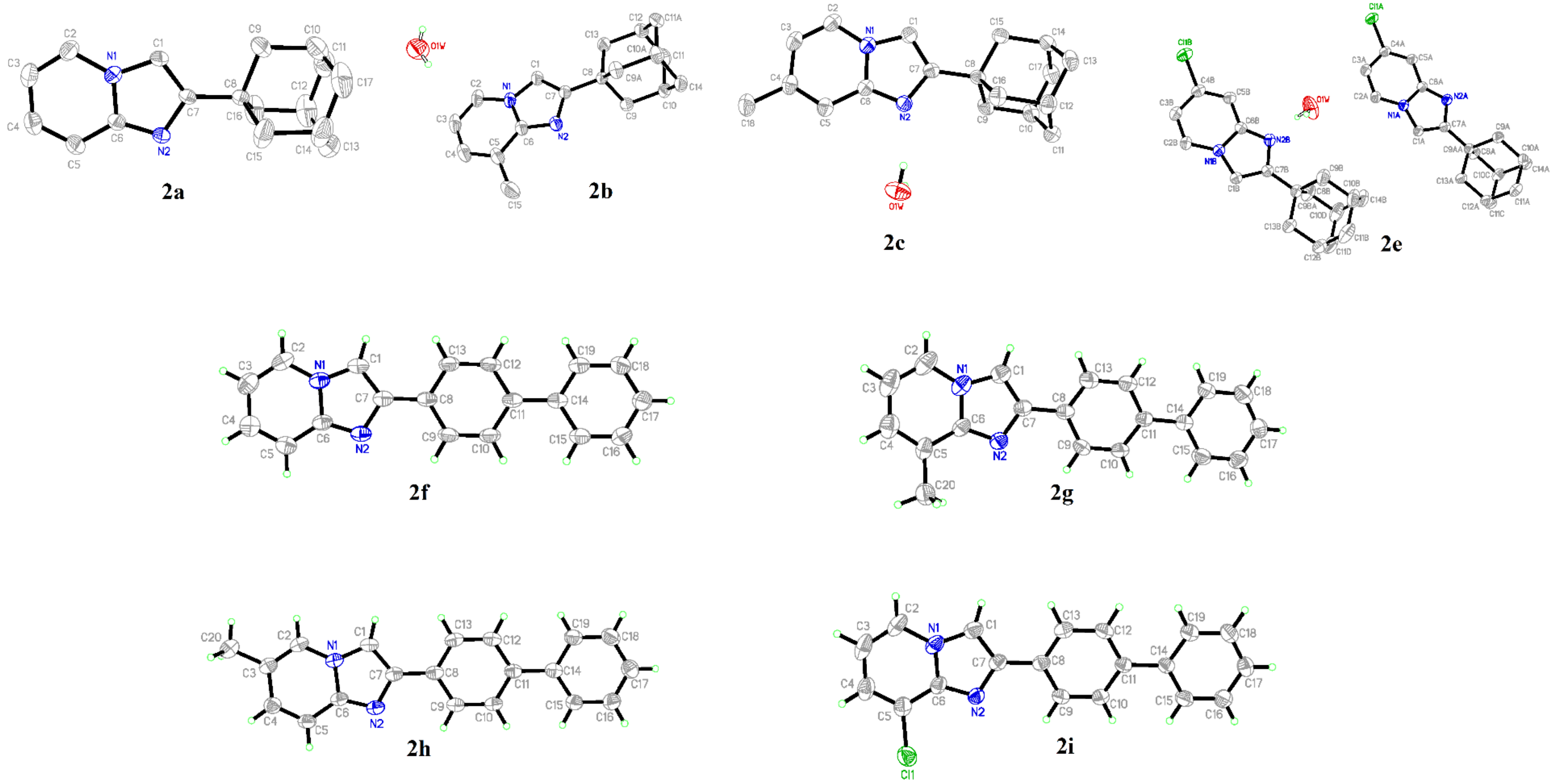

Figure 2.1: The molecular structures of **2**(**a-c**) and **2**(**e-i**) with atom numbering schemes and 30% probability displacement ellipsoids. Hydrogen atoms for structure containing adamantly moiety (**2a-c** and **2e**) were omitted for clarity.

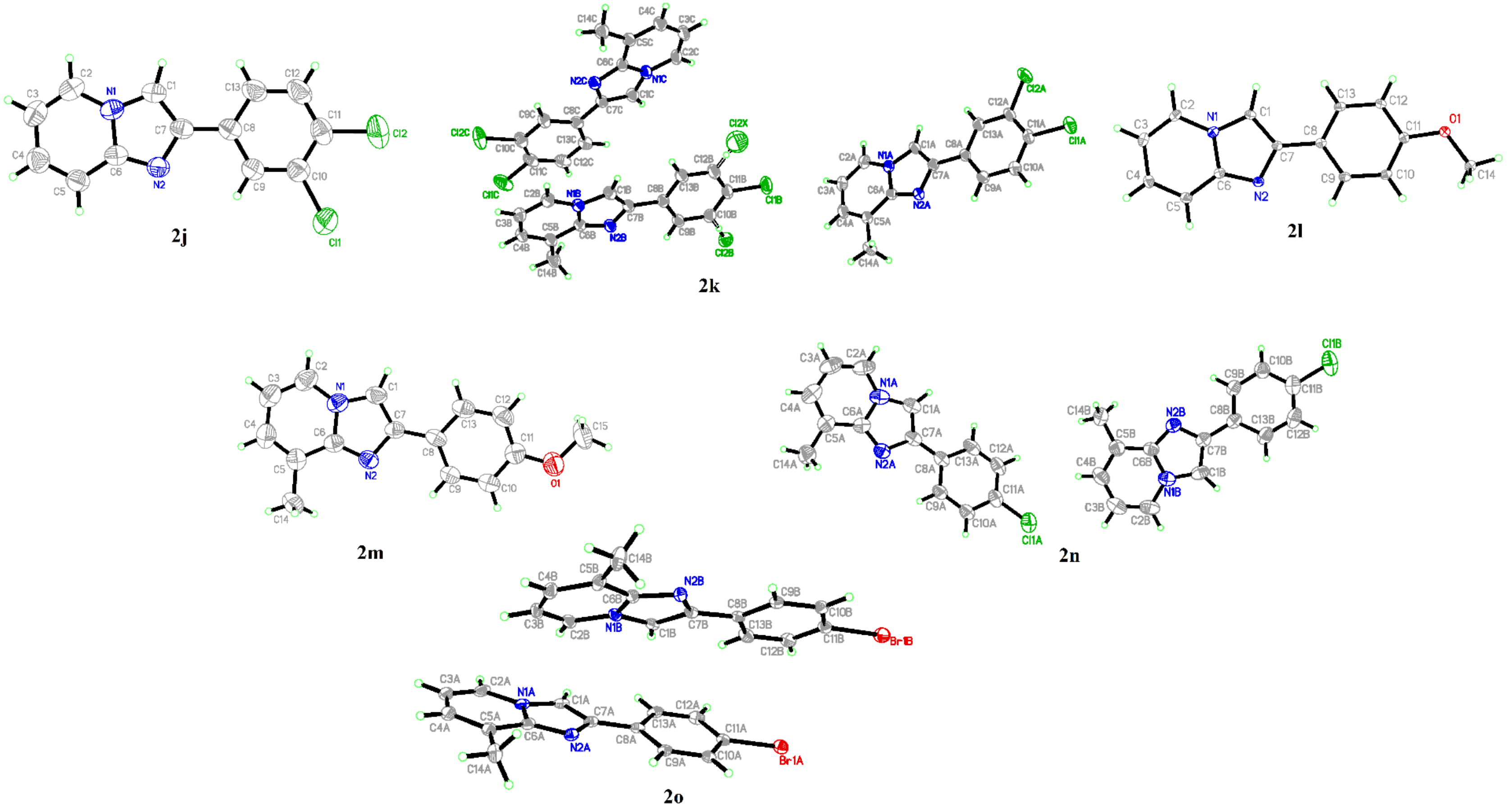

Figure 2.2: The molecular structures of **2**(**j**-**o**) with atom numbering schemes and 30% probability displacement ellipsoids.

2.4 Supramolecular feature

In the crystal of **2a**, molecules connected into an infinite linear chain, running along the *c*-axis *via* weak C1—H1*A*···N2 hydrogen bond. While these individual chains were further consolidated by π···π interaction involving the centroid of the pyridine ring (*Cg*1 = centroid of N1—C2—C3—C4—C5—C6 ring) (Figure 2.3). For hydrated compounds **2b**, **2c** and **2e**, all their water solvate are lie on the 2-fold axis (Figure 2.4). In the supramolecular assemble of **2c**, the only intermolecular interaction is the O1W—H1*W1*···N2 hydrogen bond which two main molecules are connected by the water molecule. However, the water molecule played an important rules in the crystal packing of compound **2b** and **2e**. In both of their crystal packing, water molecules are interconnected with each other into a chain parallel to the 2-fold axis. Meanwhile, the main molecules are connected to the water molecules through hydrogen bond [O1W—H2*W*···N2 and C13—H13*A*···O1W in **2b** (Figure 2.5a); C2A—H2*A*···O1W in **2e** (Figure 2.5b)] into a zig-zag chain propagating along *b*-axis. Furthermore, molecule A and molecule B in compound **2e** are further stabilized via π···π interaction where centroid of their pyridine ring are stacked together (Figure 2.6).

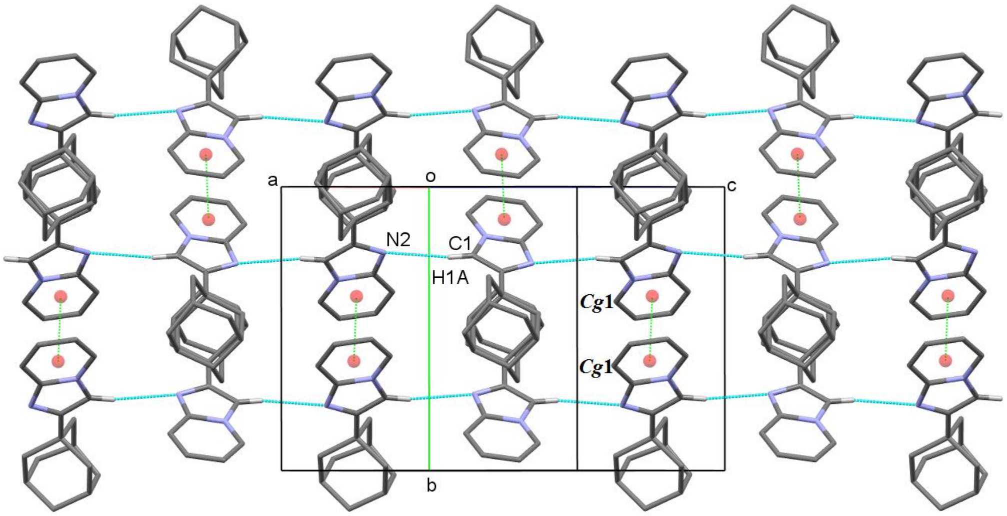

Figure 2.3: Partial packing diagram of **2a** shows hydrogen-bonded chains along *c*-axis (blue dashed lines), while further connected by π ···π interactions (green dashed lines).

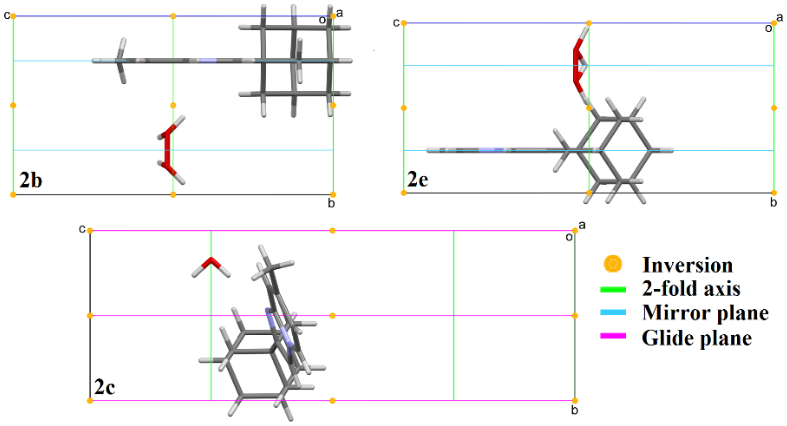

Figure 2.4: Symmetry elements in compound **2b**, **2c** and **2e**.

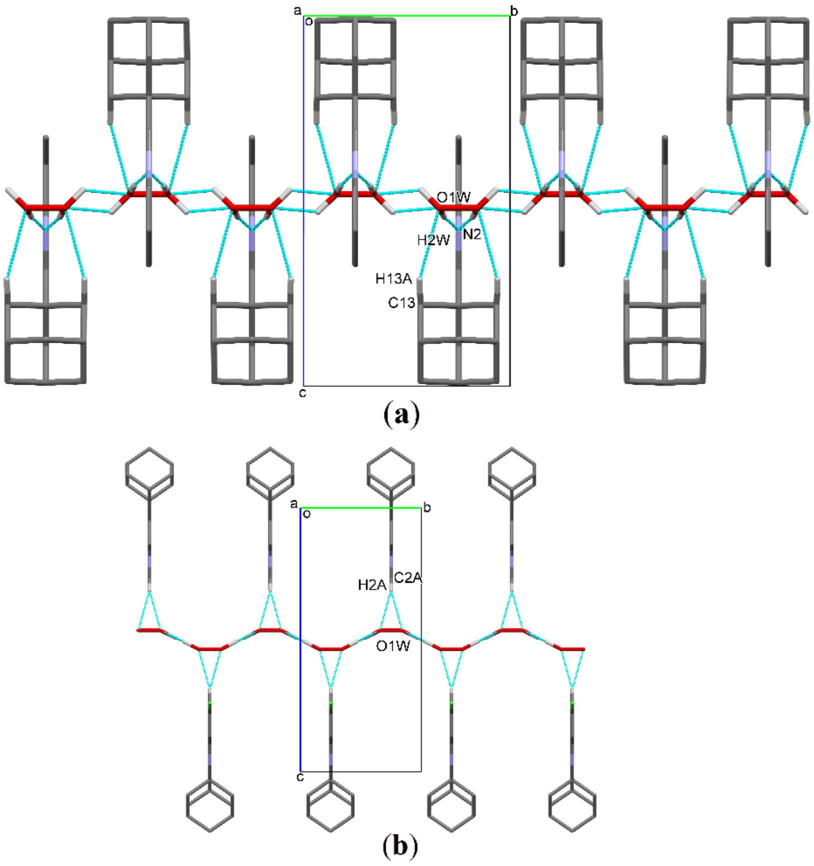

Figure 2.5: Partial packing diagram of **2b** and **2e** show hydrogen-bonded chains along *b*-axis (blue dashed lines).

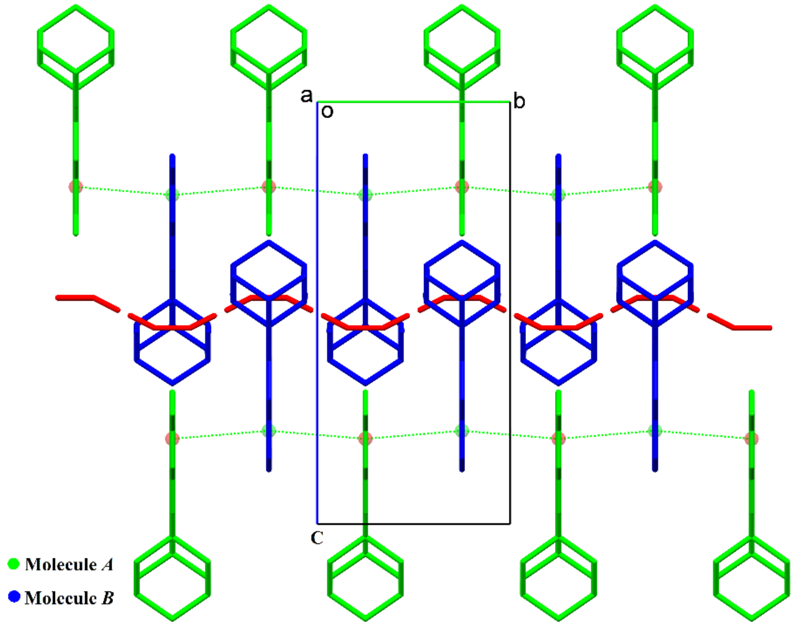

Figure 2.6: Partial packing diagram of **2e** shows π⋯π interaction chains along *b*-axis (green dashed lines).

As compound **2f** and **2h** shared 2D similarity, both their crystal packing display comparable pattern. Both their molecules were linked into a centrosymmetric dimer through C5—H5*A*···N2 hydrogen bonds which displayed a $R_2^2(8)$ ring motif (Figure 2.7). Further, those dimers were connected by C—H···π (C10—H19*A*···*Cg*2 and C13—H13*A*···*Cg*3 in **2f**; and C20—H20*A*···*Cg*4 in **2h**) into a 2D plate parallel to [0 $\bar{1}$ 1] direction (Figure 2.8). Where *Cg*2 is the centroid of the imidazole ring (*Cg*2 = N1—C1—C7—N2—C6), *Cg*3 and *Cg*4 are the centroid of the individual benzene ring from the biphenyl moiety (*Cg*3 = C8-C13, *Cg*4 = C14-C19). As for the isostructure **2g** and **2i**, both their molecules were interconnected into a fish-bone chain via C3—H3A···N2 hydrogen bond which elongated along [011] and [0 $\bar{1}$ 1] direction, respectively. Meanwhile, those chains were connected into 3D network through C—H···π interaction (C2—H2A···*Cg*4, C19—H19A···*Cg*2 in **2g**; and C2—H2A···*Cg*4 in **2i**) (Figure 2.9).

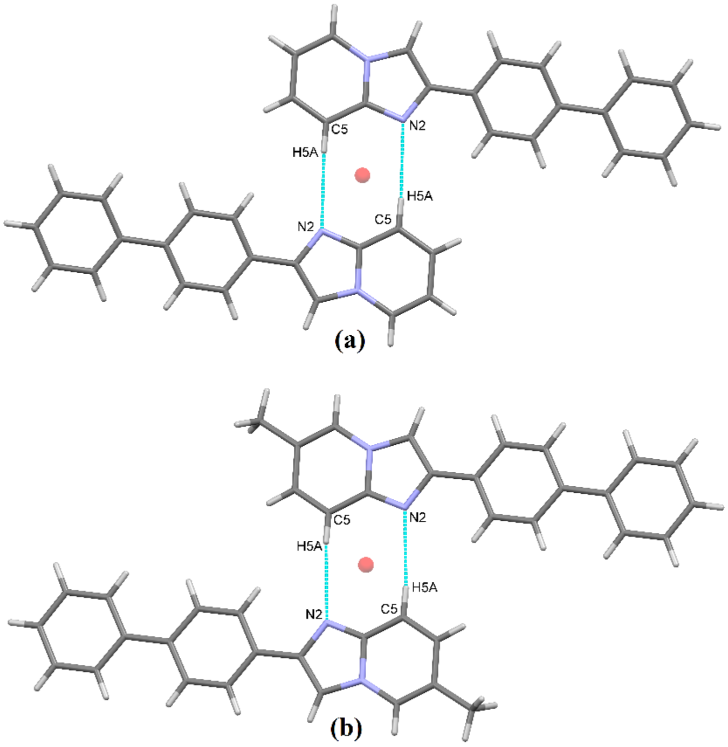

Figure 2.7: Centrosymmetric dimer of **(a) 2f** and **(b) 2h** which interconnected by C5—H5*A*···N2 hydrogen bonds

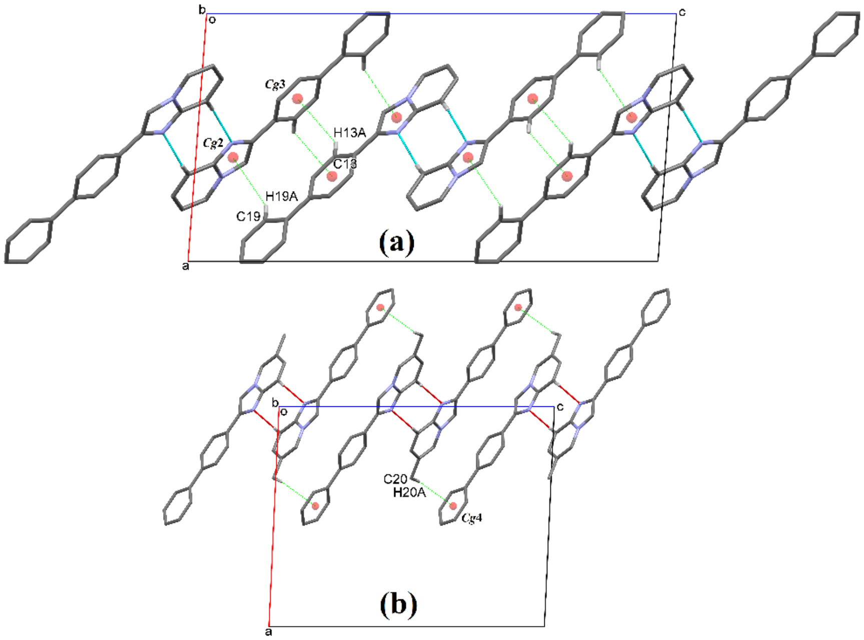

Figure 2.8: Packing diagrams of **(a) 2f** and **(b) 2h** viewed along *b*-axis. Hydrogen atoms that are not involved in hydrogen bonds were omitted for clarity.

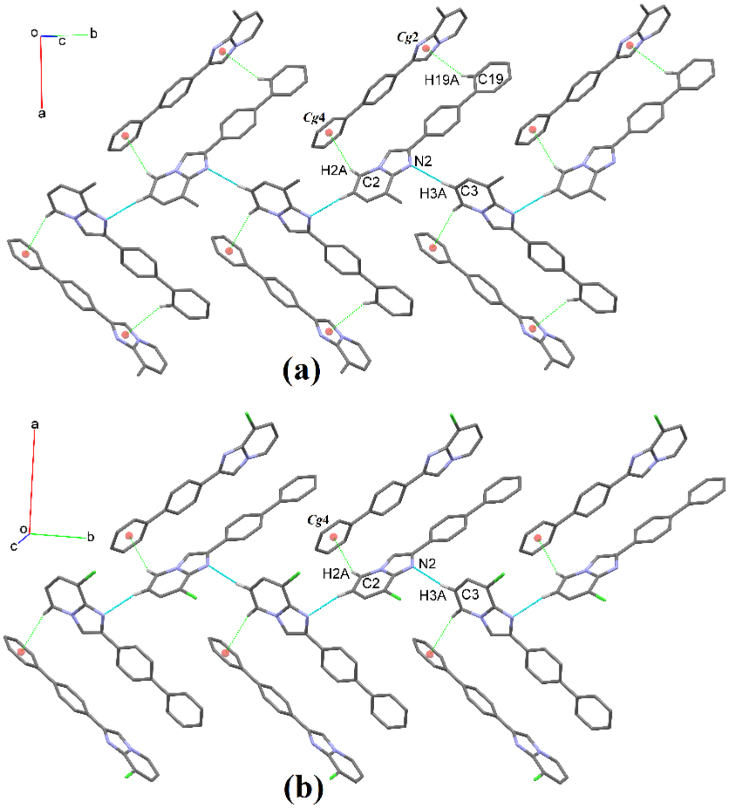

Figure 2.9: (**a**) Partial packing diagram of **2g** showing a [011] chain. (b) Partial packing diagram of **2i** showing a $[0\bar{1}1]$ chain (blue dashed lines indicated hydrogen bonds and C—H···π interactions are represented by green dashed lines).

No significant hydrogen bonding has been observed in **2j**, **2l** and **2m**, but molecules of **2j** are linked into zig-zag chains by C—H··· π interaction involving the centroid of the imidazole moiety (Figure 2.10), while molecules in **2l** were connected into a 2D plate parallel to *a*,*b*-plane *via* C10—H10A···*Cg*3 and C13—H13A···*Cg*3 interaction (Figure 2.11). In compound **2k**, the *meta*-chlorine atom of molecule *B* experienced a two-fold rotational disorder (*ca*. 180°) with a refined site-occupancy ratio of 0.872 (3) : 0.128 (3). In the crystal packing of **2k** molecules *A* and molecules *C* are interconnected by C1A—H1*AA*···N2C hydrogen bond and the structure is stabilized by several π···π and C—H···π interactions. Molecules pairs of *A*:*A* and *B*:*C* are both in face-to-face formation whereas *A*:*C* and *A*:*B* are in edge-to-face formation (Figure 2.12).

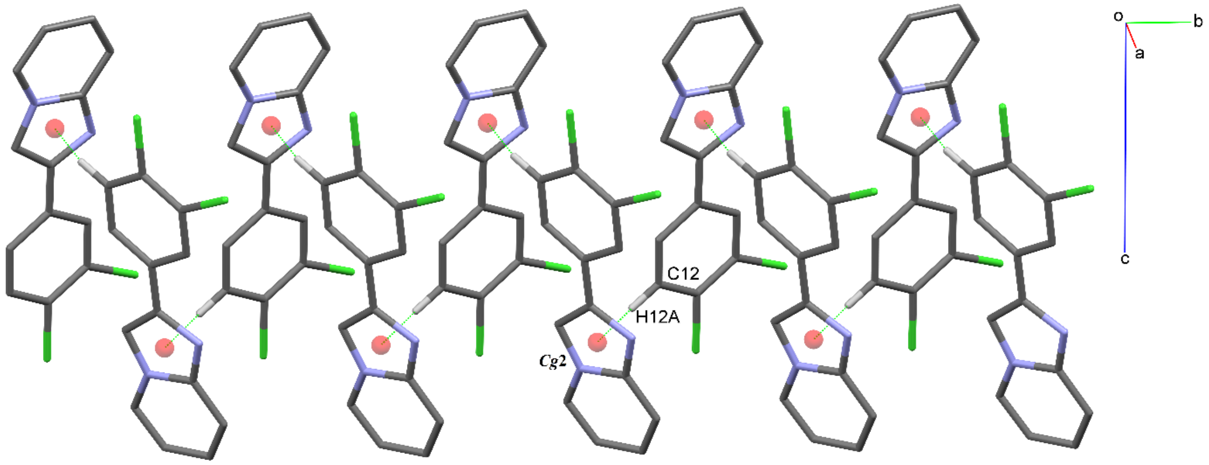

Figure 2.10: Partial packing diagram of **2j**, showing C12—H12A···Cg2 interaction which linked molecules into chain parallel to *b*-axis.

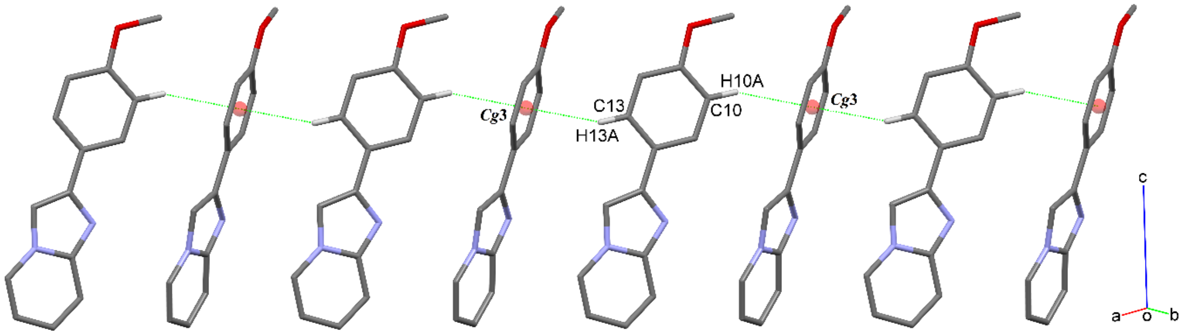

Figure 2.11: Partial packing diagram of **2j**, showing C10—H10A···*Cg*3 and C13—H13A···*Cg*3 interaction.

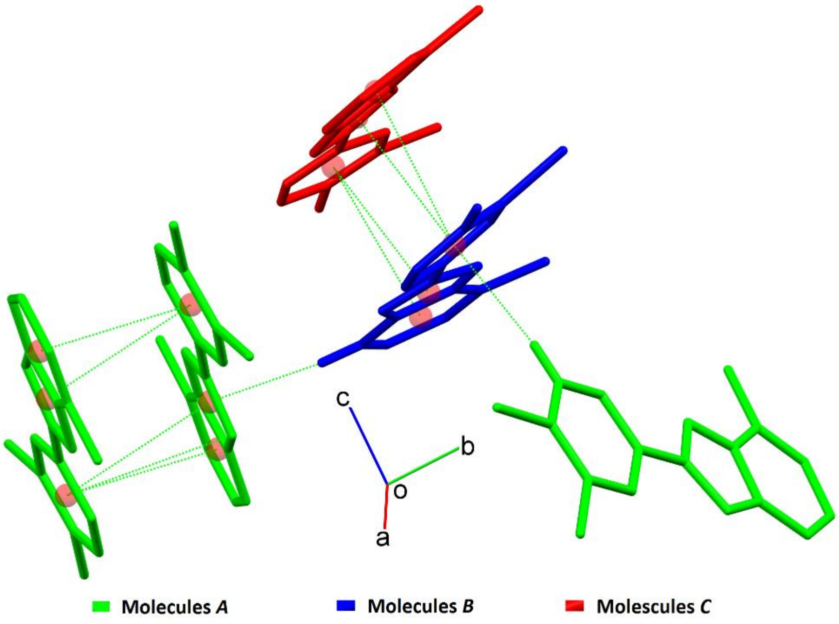

Figure 2.12: Partial packing of compound **2k** with C—H···π and π···π interactions (green lines). Hydrogen atoms that are not involved in hydrogen bonds were omitted for clarity.

Compounds **2n** and **2o** differ structurally from each other with chloro-bromo exchange at *para*-substituent of the phenyl rings. Compounds **2n** and **2o** are isomorphic structures as indicated by similar cell lattice parameters, space group and crystal packing pattern. In both crystals, molecules *A* and *B* are stabilized by an edge-to-face C—H···π interaction while two face-to-face π···π interactions are observed in between two molecules *A* or two molecules *B* (Figure 2.13). All hydrogen bonds, π···π and C—H···π interactions were summarized in Table 2.

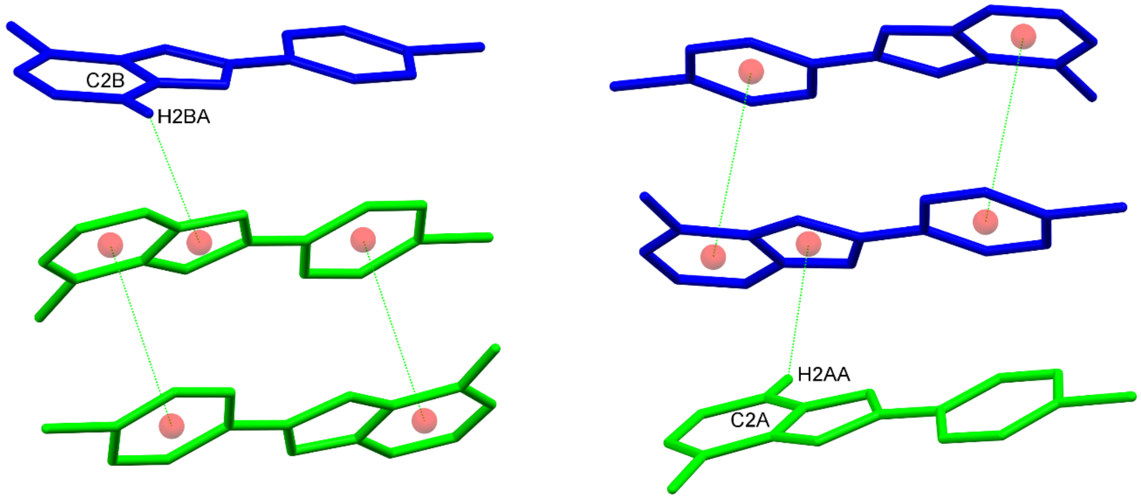

Figure 2.13: Partial packing of compounds **2n** and **2o** with C—H···π and π···π interactions. Hydrogen atoms that are not involved in hydrogen bonds were omitted for clarity.

2.1 Hydrogen bonding geometries

Table 2.4: Hydrogen bond geometries for **2**(**a-c**, **e-o**).

D—H···*A*	D—H (Å)	H···*A* (Å)	D···*A* (Å)	D—H···*A* angles (˚)	Symmetry code
**2a**					
C1—H1*A*···N2	0.93	2.54	3.384	152	*x*,1/2-*y*,-1/2+*z*
**2b**					
O1*W*—H2*W*···N2	0.83	2.07	2.902	172	-*x*,1/2+*y*,1-*z*
O1*W*—H1*W*···O1*W*	0.83	1.66	2.287	130	
C13—H13*A*···O1*W*	0.97	2.58	3.513	161	-*x*,1-*y*,1-*z*
**2c**					
O1*W*—H1*W1*···N2	0.87	2.04	2.903	169	1+*x*, -1+*y*, *z*
C10—H10*A*···*Cg*1[1]	0.98	2.92	3.714	139	
C16—H16*A*···*Cg*2[2]	0.97	2.93	3.807	151	
**2e**					
C2*A*—H2*A*···O1*W*	0.93	2.343	2.993	127	
O1*W*—H2*W*···O1*W*	0.95	1.57	2.05	167	1-*x*,1-*y*,1-*z*
**2f**					
C5—H5*A*···N2	0.93	2.61	3.521	166	1-*x*,1-*y*,-*z*
C13—H13*A*···*Cg*3[3]	0.93	2.92	3.633	134	1-*x*,1/2+*y*,1/2-*z*
C19—H18*B*···*Cg*2	0.93	2.89	3.646	140	1-*x*,1/2+*y*,1/2-*z*
**2g**					
C3—H3*A*···N2	0.93	2.48	3.396	169	1/2-*x*,-1/2+*y*,-1/2+*z*
C2—H2*A*···*Cg*4[4]	0.93	2.95	3.765	147	1-*x*,2-*y*,-1/2+*z*
C19—H19*A*···*Cg*2	0.93	2.97	3.753	142	1-*x*,2-*y*,-1/2+*z*
**2h**					
C5—H5*A*···N2	0.93	2.57	3.479	164	-*x*,2-*y*,1-*z*
C20—H20*A*···*Cg*4	0.96	2.92	3.721	141	-*x*,-1/2+*y*,1/2-*z*
**2i**					
C3—H3*A*···N2	0.95	2.46	3.391	165	1/2-*x*,-1/2+*y*,1/2+*z*
C2—H2*A*···*Cg*4	0.95	2.86	3.696	147	-*x*,1-*y*,1/2+*z*
**2j**					
C12—H12*A*···*Cg*2	0.93	2.86	3.459	123	1-*x*,-1/2+*y*,1/2-*z*
**2k**					
C1*A* —H1*AA* ···N2*C*	0.93	2.54	3.410	156	1+*x*,*y*,*z*
C2*B* —H2*BA* ···*Cg*4	0.93	2.54	3.463	175	
C10*A* —H10*A* ···*Cg*3	0.93	2.76	3.679	169	*x*,1/2-*y*,1/2+*z*
**2l**					
C10—H10*A*···*Cg*3	0.95	2.77	3.540	139	-1/2+*x*,1/2-*y*,*z*
C13—H13*A*···*Cg*3	0.95	2.66	3.445	140	1/2+*x*,-1/2-*y*,*z*
**2n**					
C2*A*—H2*AA*···*Cg*2	0.93	2.73	3.371	127	-1/2+*x*,1/2-*y*,-1/2+*z*
C2*B*—H2*BA*···*Cg*2	0.93	2.80	3.472	130	1/2+*x*,3/2-*y*,1/2+*z*
**2o**					
C2*A*—H2*AA*···*Cg*2	0.93	2.62	3.243	123	3/2-*x*,1/2+*y*,1/2-*z*
C2*B*—H2*BA*···*Cg*2	0.93	2.74	3.420	130	3/2-*x*,1/2+*y*,1/2-*z*

*Cg*1 = N1-C2-C3-C4-C5-C6/N1A-C2A-C3A-C4A-C5A-C6A pyridine ring.

*Cg*2 = N1-C1-C7-N2-C6/N1B-C1B-C7B-N2B-C6B imidazole ring.

*Cg*3 = C8-C9-C10-C11-C12-C13 phenyl ring.

*Cg*4 = C14-C15-C16-C17-C18-C19 phenyl ring.

3.0 Raw data for Anti-cholinesterase Assay

3.1 AChE inhibition

3.1.1 Raw data for IC_{50} calculation of compound **2b**.

First read									
			**50**	**100**	**150**	**200**	**250**	**300**
**Time**	**Control**	**tacrine**	**a**	**b**	**c**	**d**	**e**	**f**
0	0.062	0.051	0.055	0.053	0.055	0.057	0.056	0.059
1	0.069	0.052	0.060	0.057	0.059	0.060	0.059	0.061
2	0.076	0.053	0.065	0.062	0.062	0.063	0.060	0.062
3	0.083	0.055	0.069	0.066	0.066	0.065	0.063	0.064
4	0.089	0.055	0.074	0.071	0.070	0.069	0.065	0.066
5	0.096	0.057	0.080	0.076	0.074	0.072	0.068	0.067
6	0.103	0.058	0.084	0.081	0.078	0.076	0.070	0.069
7	0.109	0.059	0.089	0.085	0.082	0.080	0.073	0.070
8	0.115	0.060	0.094	0.090	0.088	0.082	0.076	0.072
9	0.120	0.062	0.099	0.095	0.090	0.082	0.080	0.073
10	0.126	0.063	0.105	0.099	0.094	0.088	0.081	0.075
**Gradient**	**0.0065**	**0.0012**	**0.0049**	**0.0047**	**0.0040**	**0.0031**	**0.0026**	**0.0016**
**% inhibition**		**81.43**	**23.63**	**27.71**	**38.54**	**52.60**	**60.34**	**75.67**

**conc**	**% inhibition**
50	23.6287
100	27.7075
150	38.5373
200	52.60
250	60.3376
300	75.67

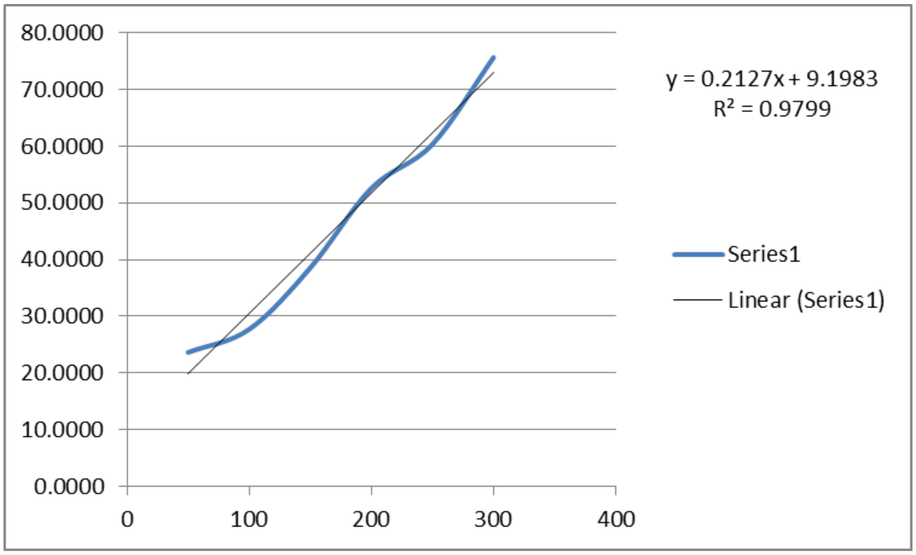

IC_{50}= (50-9.1983)/0.2127 = 191.8274565

Second read									
			**50**	**100**	**150**	**200**	**250**	**300**
**Time**	**Control**	**tacrine**	**a**	**b**	**c**	**d**	**e**	**f**
0	0.062	0.052	0.056	0.052	0.055	0.058	0.056	0.055
1	0.069	0.053	0.062	0.055	0.059	0.061	0.058	0.057
2	0.076	0.054	0.067	0.059	0.062	0.063	0.060	0.058
3	0.083	0.055	0.072	0.063	0.065	0.066	0.063	0.060
4	0.089	0.056	0.076	0.067	0.068	0.069	0.065	0.061
5	0.096	0.057	0.083	0.070	0.071	0.072	0.067	0.063
6	0.103	0.058	0.087	0.075	0.074	0.075	0.069	0.065
7	0.109	0.059	0.092	0.080	0.078	0.078	0.071	0.066
8	0.115	0.060	0.096	0.081	0.081	0.081	0.074	0.068
9	0.120	0.061	0.104	0.087	0.085	0.083	0.076	0.069
10	0.126	0.063	0.109	0.093	0.088	0.088	0.078	0.070
**Gradient**	**0.0065**	**0.0010**	**0.0052**	**0.0040**	**0.0033**	**0.0029**	**0.0022**	**0.0015**
**% inhibition**		**83.8256**	**19.6906**	**37.97**	**49.65**	**54.71**	**65.68**	**76.23**

**conc**	**% inhibition**
50	19.6906
100	37.97
150	49.65
200	54.71
250	65.68
300	76.23

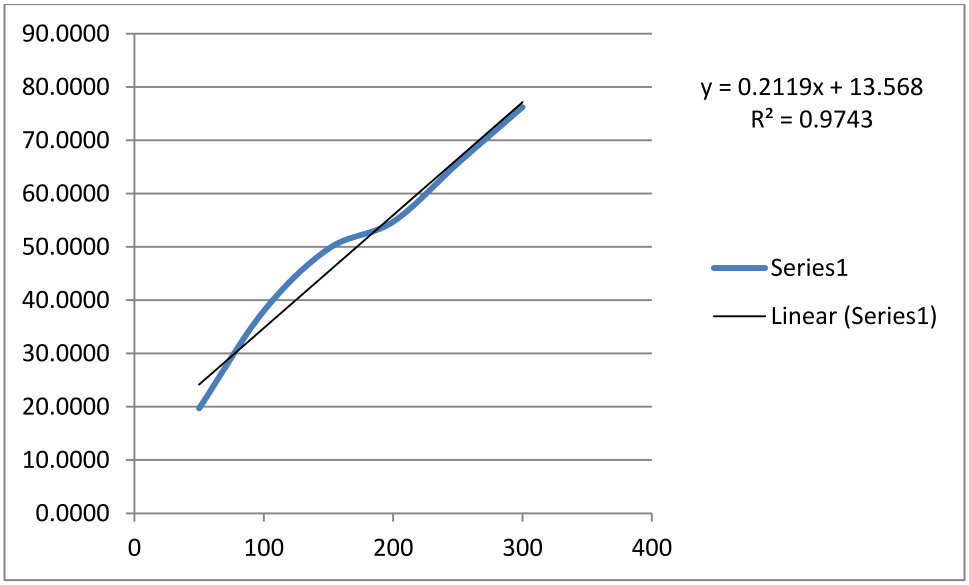

IC_{50}= (50-13.568)/0.2119 = 171.9301557

Third read								
			**50**	**100**	**150**	**200**	**250**	**300**
**Time**	**Control**	**tacrine**	**a**	**b**	**c**	**d**	**e**	**f**
0	0.062	0.052	0.056	0.052	0.055	0.056	0.055	0.055
1	0.069	0.053	0.061	0.057	0.058	0.059	0.057	0.057
2	0.076	0.054	0.066	0.061	0.061	0.061	0.059	0.058
3	0.083	0.055	0.070	0.065	0.064	0.064	0.061	0.059
4	0.089	0.056	0.075	0.069	0.067	0.066	0.063	0.061
5	0.096	0.058	0.079	0.073	0.070	0.068	0.065	0.063
6	0.103	0.059	0.082	0.079	0.073	0.071	0.067	0.065
7	0.109	0.060	0.088	0.082	0.077	0.074	0.070	0.067
8	0.115	0.061	0.091	0.086	0.079	0.075	0.073	0.070
9	0.120	0.062	0.097	0.091	0.081	0.079	0.075	0.070
10	0.126	0.064	0.099	0.094	0.083	0.081	0.078	0.071
**Gradient**	**0.0065**	**0.0012**	**0.0043**	**0.0042**	**0.0034**	**0.0025**	**0.0023**	**0.0017**
**% inhibition**		**81.72**	**32.91**	**34.60**	**47.82**	**61.74**	**64.70**	**73.56**

**conc**	**% inhibition**
50	32.91
100	34.60
150	47.82
200	61.74
250	64.70
300	73.56

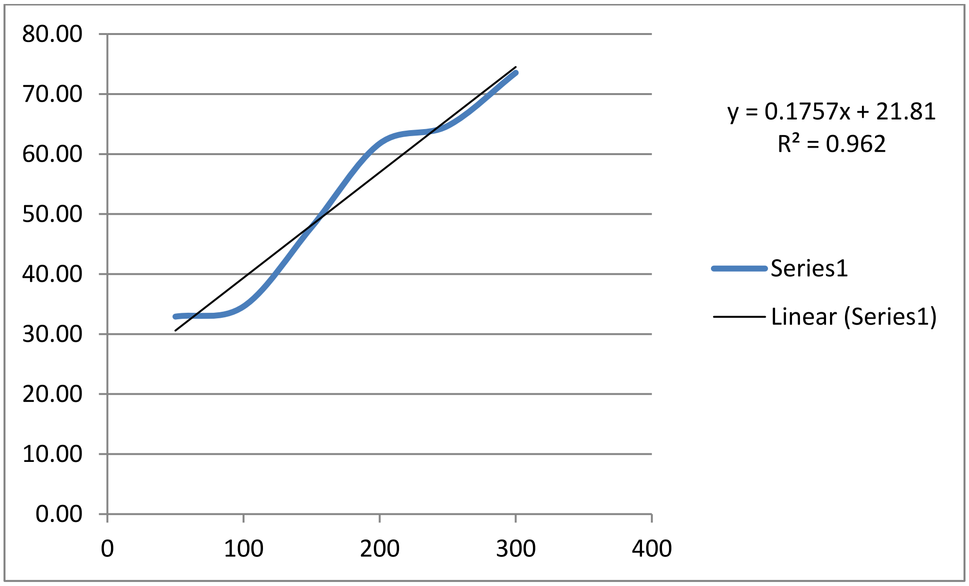

IC_{50}= (50-21.81)/0.1757 = 160.4439385

3.1.2 Raw data for IC_{50} calculation of compound **2c**.

First read									
			**25**	**50**	**100**	**150**	**200**	**250**
**Time**	**Control**	**tacrine**	**a**	**b**	**c**	**d**	**e**	**f**
0	0.072	0.052	0.058	0.054	0.057	0.055	0.057	0.055
1	0.084	0.054	0.066	0.061	0.061	0.058	0.059	0.057
2	0.093	0.055	0.073	0.067	0.065	0.061	0.062	0.059
3	0.105	0.057	0.082	0.073	0.070	0.064	0.065	0.062
4	0.113	0.058	0.089	0.078	0.074	0.068	0.067	0.064
5	0.127	0.060	0.096	0.083	0.078	0.070	0.070	0.066
6	0.132	0.061	0.106	0.088	0.082	0.073	0.072	0.070
7	0.142	0.063	0.110	0.095	0.086	0.076	0.075	0.070
8	0.149	0.064	0.119	0.099	0.089	0.078	0.078	0.073
9	0.157	0.066	0.124	0.103	0.093	0.083	0.081	0.075
10	0.164	0.067	0.130	0.109	0.096	0.083	0.083	0.076
**Gradient**	**0.0092**	**0.0015**	**0.0073**	**0.0054**	**0.0040**	**0.0029**	**0.0026**	**0.0022**
**% inhibition**		**83.67**	**20.55**	**41.33**	**56.96**	**68.34**	**71.21**	**76.15**

**conc**	**% inhibition**
25	20.5475
50	41.3259
100	56.9591
150	68.34
200	71.2071
250	76.15

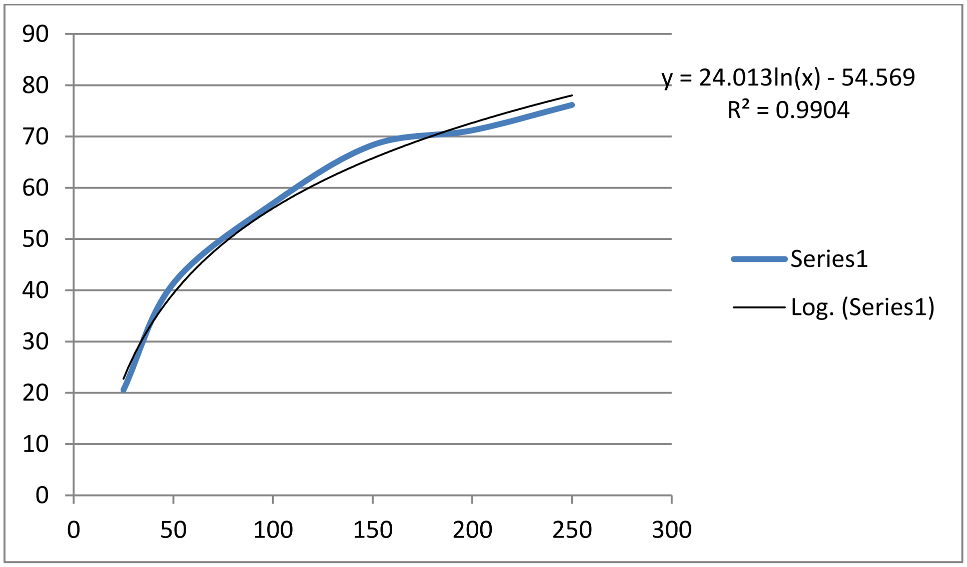

IC_{50}=EXP((50+54.569)/24.013) = 77.8421361

Second read								
			**25**	**50**	**100**	**150**	**200**	**250**
**Time**	**Control**	**tacrine**	**a**	**b**	**c**	**d**	**e**	**f**
0	0.072	0.052	0.056	0.055	0.062	0.055	0.057	0.053
1	0.084	0.054	0.064	0.061	0.067	0.058	0.060	0.055
2	0.093	0.056	0.070	0.066	0.073	0.060	0.063	0.057
3	0.105	0.057	0.077	0.071	0.078	0.063	0.066	0.060
4	0.113	0.058	0.084	0.075	0.083	0.066	0.069	0.061
5	0.127	0.060	0.088	0.080	0.087	0.068	0.072	0.063
6	0.132	0.062	0.098	0.085	0.092	0.072	0.074	0.065
7	0.142	0.063	0.105	0.092	0.098	0.073	0.077	0.068
8	0.149	0.065	0.112	0.094	0.102	0.076	0.080	0.069
9	0.157	0.066	0.113	0.100	0.105	0.078	0.082	0.071
10	0.164	0.068	0.125	0.103	0.109	0.082	0.085	0.072
**Gradient**	**0.0092**	**0.0016**	**0.0067**	**0.0048**	**0.0048**	**0.0026**	**0.0028**	**0.0020**
**% inhibition**		**83.0805**	**27.0778**	**47.36**	**48.25**	**71.41**	**69.72**	**78.73**

**conc**	**% inhibition**
25	27.0778
50	47.36
100	48.25
150	71.41
200	69.72
250	78.73

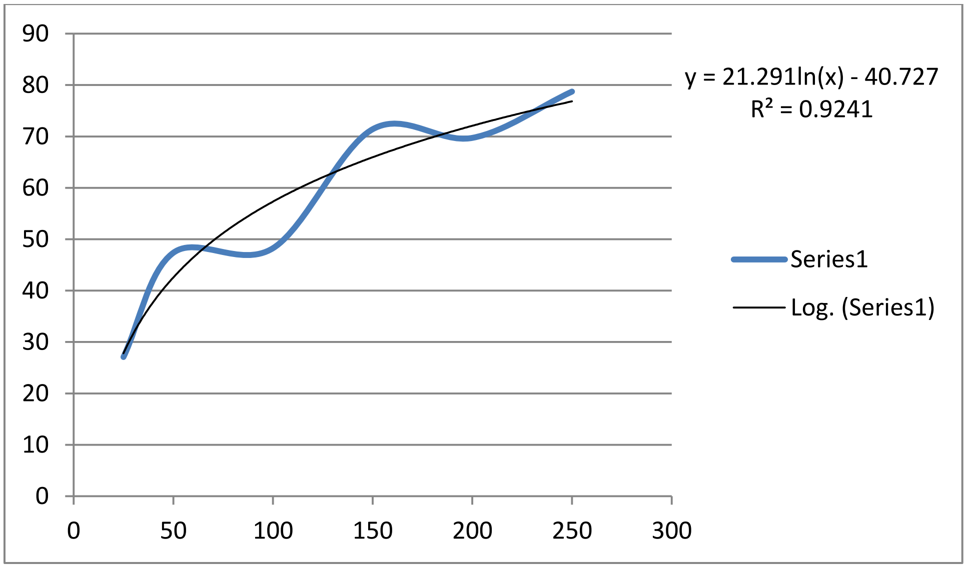

Third read								
			25	50	100	150	200	250
**Time**	**Control**	**tacrine**	**a**	**b**	**c**	**d**	**e**	**f**
0	0.072	0.052	0.056	0.054	0.080	0.053	0.054	0.053
1	0.084	0.053	0.063	0.059	0.087	0.056	0.056	0.055
2	0.093	0.054	0.069	0.065	0.093	0.058	0.058	0.057
3	0.105	0.056	0.075	0.069	0.099	0.061	0.060	0.059
4	0.113	0.057	0.082	0.074	0.104	0.063	0.063	0.061
5	0.127	0.058	0.088	0.080	0.110	0.065	0.065	0.062
6	0.132	0.059	0.096	0.084	0.115	0.068	0.066	0.065
7	0.142	0.060	0.100	0.091	0.120	0.072	0.068	0.067
8	0.149	0.060	0.107	0.096	0.124	0.074	0.072	0.069
9	0.157	0.061	0.112	0.101	0.129	0.075	0.072	0.072
10	0.164	0.062	0.118	0.107	0.134	0.079	0.077	0.072
**Gradient**	**0.0092**	**0.0010**	**0.0062**	**0.0053**	**0.0047**	**0.0026**	**0.0022**	**0.0020**
**% inhibition**		**89.12**	**32.32**	**42.61**	**49.14**	**72.20**	**76.25**	**78.33**

**conc**	**% inhibition**
25	32.32
50	42.61
100	49.14
150	72.20
200	76.25
250	78.33

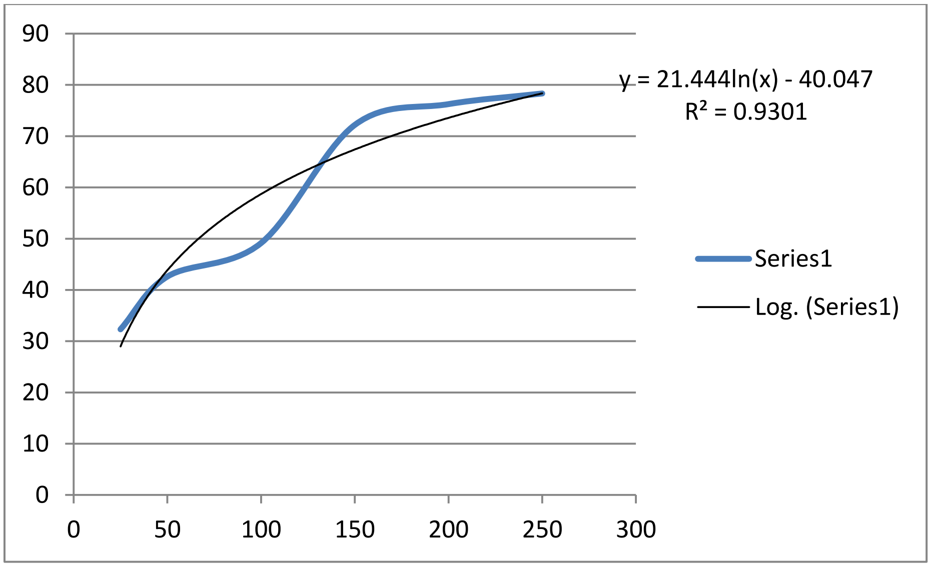

3.1.3 Raw data for IC_{50} calculation of compound **2f**.

First read									
			**30**	**40**	**50**	**60**	**70**	**80**
**Time**	**Control**	**tacrine**	**a**	**b**	**c**	**d**	**e**	**f**
0	0.084	0.051	0.080	0.085	0.098	0.095	0.088	0.079
1	0.100	0.053	0.094	0.100	0.113	0.106	0.095	0.081
2	0.116	0.055	0.108	0.113	0.126	0.115	0.101	0.083
3	0.132	0.057	0.123	0.124	0.138	0.123	0.107	0.085
4	0.146	0.059	0.134	0.135	0.148	0.131	0.112	0.087
5	0.159	0.061	0.146	0.146	0.158	0.138	0.118	0.089
6	0.170	0.064	0.159	0.155	0.166	0.143	0.124	0.093
7	0.181	0.066	0.171	0.165	0.173	0.150	0.128	0.092
8	0.191	0.068	0.184	0.173	0.180	0.155	0.134	0.093
9	0.203	0.070	0.187	0.181	0.187	0.159	0.137	0.095
10	0.210	0.072	0.197	0.188	0.193	0.164	0.142	0.096
**Gradient**	**0.0127**	**0.0021**	**0.0119**	**0.0102**	**0.0093**	**0.0068**	**0.0054**	**0.0017**
**% inhibition**		**83.12**	**6.18**	**19.47**	**26.65**	**46.62**	**57.54**	**86.28**

**conc**	**% inhibition**
30	0.0000
40	19.4684
50	26.6523
60	46.62
70	57.5431
80	86.28

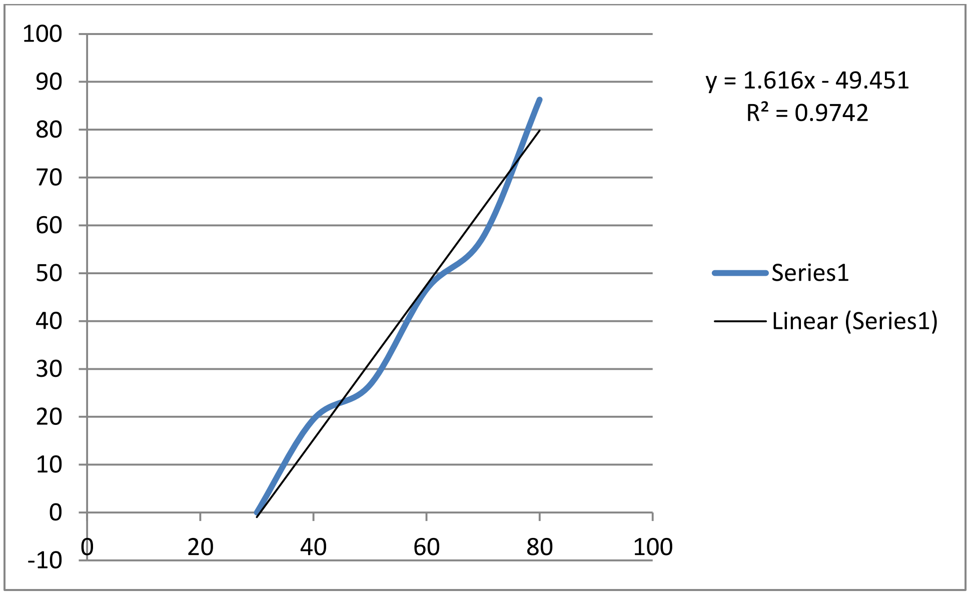

Second read								
			**30**	**40**	**50**	**60**	**70**	**80**
**Time**	**Control**	**tacrine**	**a**	**b**	**c**	**d**	**e**	**f**
0	0.084	0.053	0.095	0.074	0.074	0.076	0.081	0.080
1	0.100	0.056	0.115	0.086	0.080	0.082	0.085	0.083
2	0.116	0.058	0.133	0.095	0.084	0.087	0.089	0.086
3	0.132	0.060	0.149	0.106	0.088	0.091	0.093	0.089
4	0.146	0.063	0.163	0.114	0.093	0.097	0.097	0.090
5	0.159	0.066	0.176	0.122	0.099	0.101	0.100	0.094
6	0.170	0.068	0.186	0.131	0.102	0.105	0.104	0.095
7	0.181	0.071	0.197	0.139	0.106	0.109	0.108	0.098
8	0.191	0.074	0.208	0.147	0.109	0.113	0.111	0.100
9	0.203	0.076	0.219	0.152	0.115	0.116	0.112	0.101
10	0.210	0.078	0.226	0.163	0.118	0.120	0.115	0.101
**Gradient**	**0.0127**	**0.0025**	**0.0129**	**0.0086**	**0.0044**	**0.0043**	**0.0035**	**0.0022**
**% inhibition**		**79.8851**	**-1.6523**	**31.90**	**65.52**	**65.66**	**72.63**	**82.61**

**conc**	**% inhibition**
30	0.0000
40	31.90
50	65.52
60	65.66
70	72.63
80	82.61

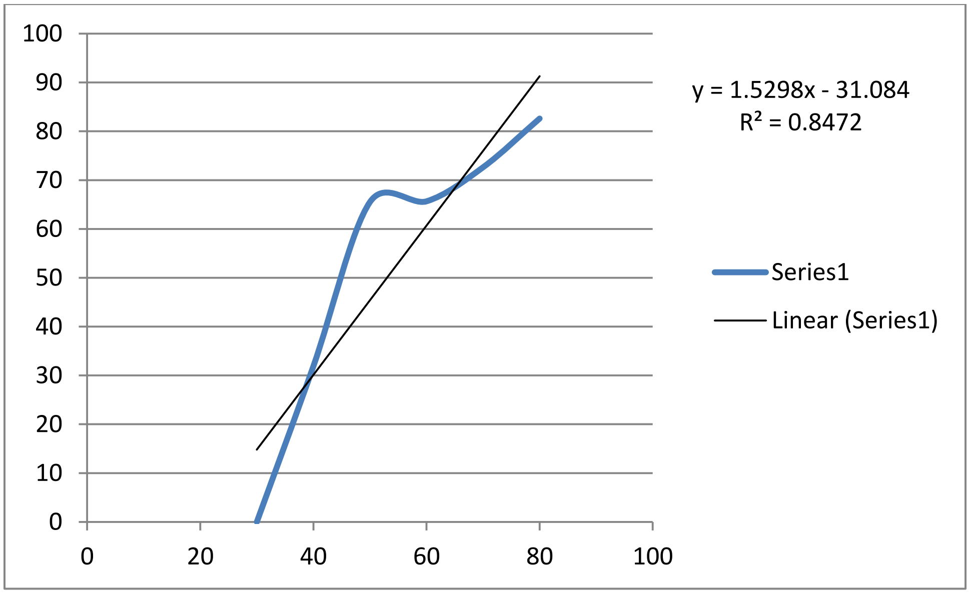

Third read								
			**30**	**40**	**50**	**60**	**70**	**80**
**Time**	**Control**	**tacrine**	**a**	**b**	**c**	**d**	**e**	**f**
0	0.084	0.052	0.087	0.075	0.073	0.077	0.080	0.081
1	0.100	0.054	0.102	0.089	0.078	0.082	0.084	0.084
2	0.116	0.056	0.116	0.101	0.083	0.086	0.087	0.087
3	0.132	0.058	0.130	0.113	0.088	0.090	0.089	0.090
4	0.146	0.060	0.144	0.122	0.092	0.094	0.092	0.093
5	0.159	0.061	0.158	0.132	0.098	0.098	0.095	0.095
6	0.170	0.063	0.168	0.142	0.101	0.101	0.097	0.097
7	0.181	0.066	0.180	0.150	0.105	0.104	0.098	0.100
8	0.191	0.067	0.192	0.159	0.109	0.109	0.102	0.103
9	0.203	0.070	0.206	0.166	0.112	0.110	0.103	0.105
10	0.210	0.072	0.212	0.173	0.118	0.111	0.105	0.105
**Gradient**	**0.0127**	**0.0020**	**0.0127**	**0.0097**	**0.0060**	**0.0035**	**0.0024**	**0.0025**
**% inhibition**		**84.48**	**-0.07**	**23.42**	**52.51**	**72.27**	**80.68**	**80.17**

**conc**	**% inhibition**
30	0.00
40	23.42
50	52.51
60	72.27
70	80.68
80	80.17

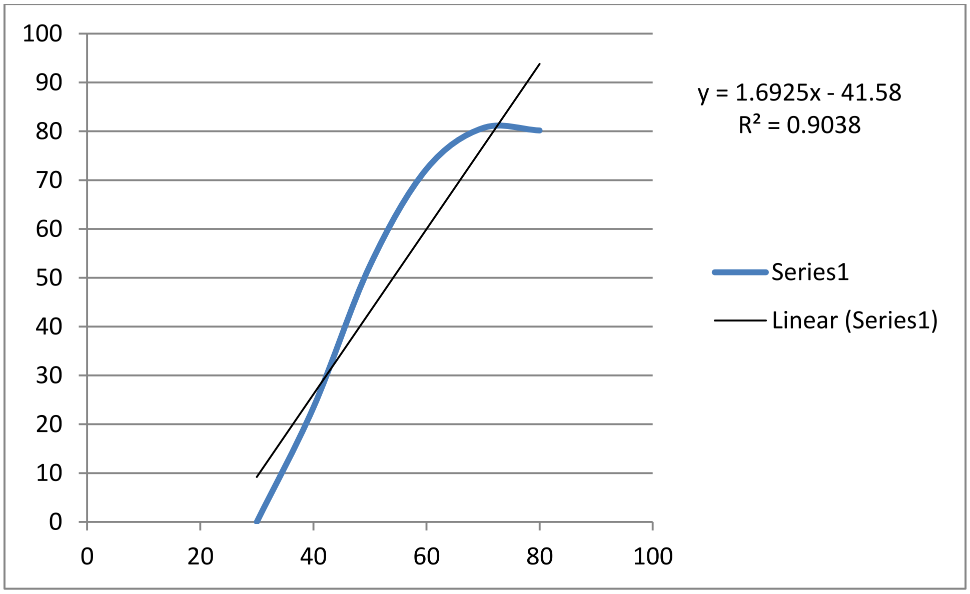

3.1.4 Raw data for IC_{50} calculation of compound **2g**.

First read								
			**50**	**100**	**150**	**200**	**250**	**300**
**Time**	**Control**	**tacrine**	**a**	**b**	**c**	**d**	**e**	**f**
0	0.062	0.049	0.089	0.117	0.142	0.190	0.192	0.220
1	0.066	0.050	0.094	0.118	0.138	0.191	0.195	0.215
2	0.071	0.051	0.100	0.117	0.140	0.194	0.191	0.210
3	0.076	0.051	0.104	0.121	0.143	0.192	0.189	0.216
4	0.082	0.052	0.108	0.125	0.145	0.192	0.189	0.213
5	0.086	0.053	0.114	0.126	0.146	0.193	0.193	0.209
6	0.091	0.054	0.117	0.128	0.148	0.193	0.185	0.211
7	0.096	0.054	0.123	0.126	0.145	0.198	0.186	0.207
8	0.100	0.055	0.126	0.128	0.148	0.194	0.188	0.202
9	0.106	0.056	0.131	0.131	0.148	0.196	0.191	0.203
10	0.107	0.056	0.135	0.132	0.147	0.201	0.192	0.196
**Gradient**	**0.0047**	**0.0007**	**0.0046**	**0.0016**	**0.0009**	**0.0008**	**-0.0003**	**-0.0019**
**% inhibition**		**84.78**	**3.08**	**66.67**	**81.50**	**83.04**	**106.74**	**140.85**

**conc**	**% inhibition**
50	3.0829
100	66.6667
150	81.5029
200	83.04
250	100.0000
300	100.00

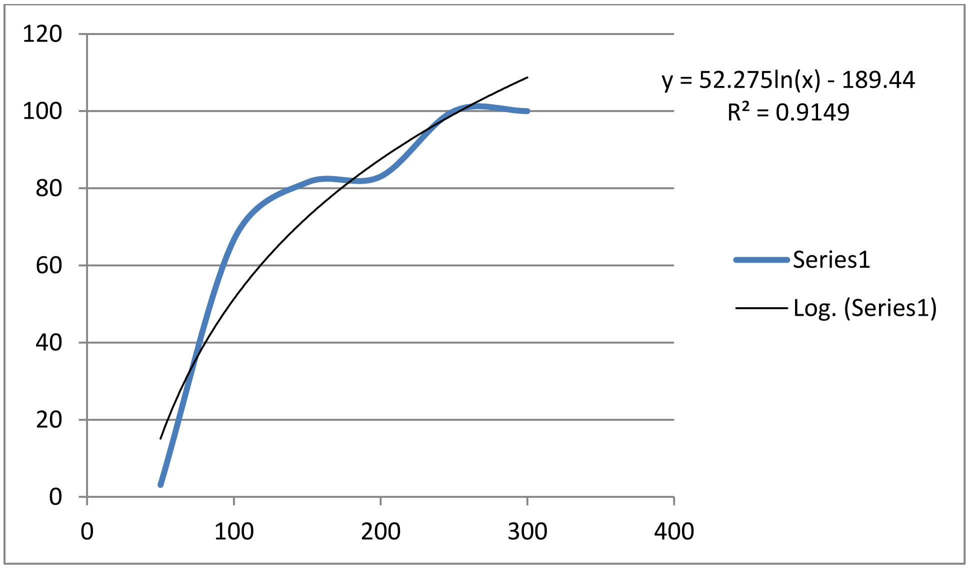

Second read								
			**50**	**100**	**150**	**200**	**250**	**300**
**Time**	**Control**	**tacrine**	**a**	**b**	**c**	**d**	**e**	**f**
0	0.062	0.051	0.089	0.124	0.156	0.176	0.210	0.225
1	0.066	0.051	0.093	0.125	0.157	0.168	0.214	0.219
2	0.071	0.052	0.095	0.130	0.160	0.171	0.212	0.215
3	0.076	0.053	0.099	0.131	0.152	0.172	0.215	0.210
4	0.082	0.053	0.101	0.131	0.158	0.173	0.215	0.213
5	0.086	0.054	0.106	0.137	0.157	0.172	0.213	0.214
6	0.091	0.055	0.110	0.136	0.158	0.174	0.215	0.208
7	0.096	0.055	0.111	0.139	0.159	0.171	0.215	0.206
8	0.100	0.056	0.114	0.136	0.162	0.173	0.213	0.203
9	0.106	0.057	0.121	0.140	0.158	0.176	0.216	0.214
10	0.107	0.057	0.122	0.143	0.164	0.178	0.216	0.207
**Gradient**	**0.0047**	**0.0007**	**0.0033**	**0.0018**	**0.0006**	**0.0004**	**0.0004**	**-0.0014**
**% inhibition**		**86.1272**	**29.2871**	**62.62**	**87.67**	**90.94**	**92.10**	**130.64**

**conc**	**% inhibition**
50	29.2871
100	62.62
150	87.67
200	90.94
250	92.10
300	100.00

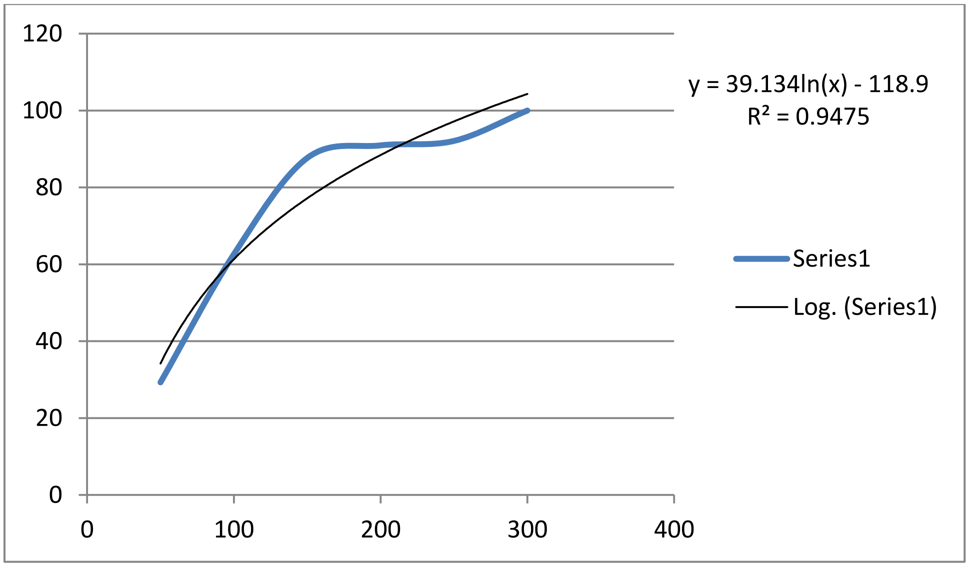

Third read									
			**50**	**100**	**150**	**200**	**250**	**300**
**Time**	**Control**	**tacrine**	**a**	**b**	**c**	**d**	**e**	**f**
0	0.062	0.050	0.088	0.123	0.154	0.170	0.206	0.230
1	0.066	0.050	0.091	0.119	0.148	0.165	0.195	0.220
2	0.071	0.051	0.095	0.123	0.152	0.166	0.196	0.223
3	0.076	0.051	0.098	0.127	0.153	0.166	0.199	0.220
4	0.082	0.052	0.103	0.129	0.150	0.167	0.204	0.213
5	0.086	0.052	0.107	0.129	0.156	0.163	0.203	0.213
6	0.091	0.053	0.108	0.128	0.157	0.164	0.203	0.218
7	0.096	0.053	0.112	0.136	0.150	0.169	0.196	0.216
8	0.100	0.053	0.114	0.134	0.156	0.168	0.199	0.218
9	0.106	0.054	0.119	0.132	0.155	0.166	0.200	0.215
10	0.107	0.054	0.122	0.138	0.158	0.167	0.194	0.208
**Gradient**	**0.0047**	**0.0004**	**0.0034**	**0.0016**	**0.0007**	**0.0000**	**-0.0003**	**-0.0013**
**% inhibition**		**90.94**	**28.32**	**65.90**	**85.81**	**100.39**	**107.32**	**128.52**

**conc**	**% inhibition**
50	28.32
100	65.90
150	85.81
200	100.00
250	100.00
300	100.00

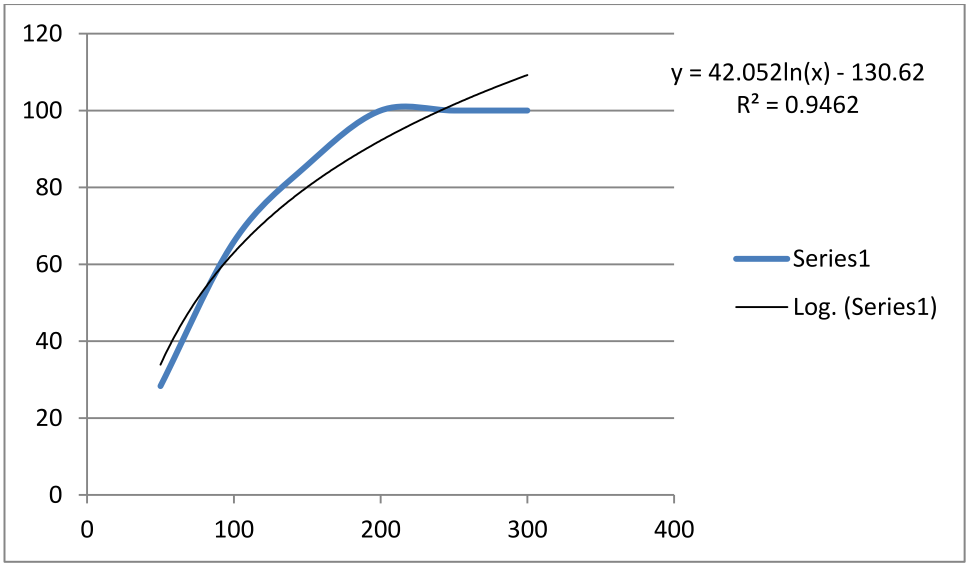

3.1.5 Raw data for IC_{50} calculation of compound **2h**.

First read								
			**6.25**	**12.5**	**25**	**50**	**75**	**100**
**Time**	**Control**	**tacrine**	**a**	**b**	**c**	**d**	**e**	**f**
0	0.073	0.051	0.071	0.071	0.066	0.063	0.064	0.073
1	0.083	0.053	0.082	0.081	0.070	0.064	0.065	0.073
2	0.091	0.054	0.093	0.090	0.075	0.065	0.066	0.073
3	0.101	0.055	0.102	0.098	0.079	0.065	0.067	0.074
4	0.108	0.057	0.111	0.105	0.083	0.066	0.068	0.072
5	0.116	0.058	0.118	0.112	0.086	0.067	0.068	0.073
6	0.126	0.060	0.127	0.121	0.090	0.068	0.068	0.073
7	0.130	0.062	0.134	0.126	0.093	0.068	0.069	0.073
8	0.139	0.063	0.145	0.131	0.093	0.068	0.070	0.074
9	0.147	0.065	0.149	0.136	0.096	0.069	0.070	0.073
10	0.151	0.067	0.161	0.140	0.100	0.069	0.070	0.073
**Gradient**	**0.0079**	**0.0016**	**0.0087**	**0.0069**	**0.0033**	**0.0006**	**0.0006**	**0.0000**
**% inhibition**		**80.19**	**-9.87**	**12.48**	**58.20**	**92.28**	**92.40**	**99.77**

**conc**	**% inhibition**
6.25	0.0000
12.5	12.4760
25	58.1958
50	92.28
75	92.3992
100	99.77

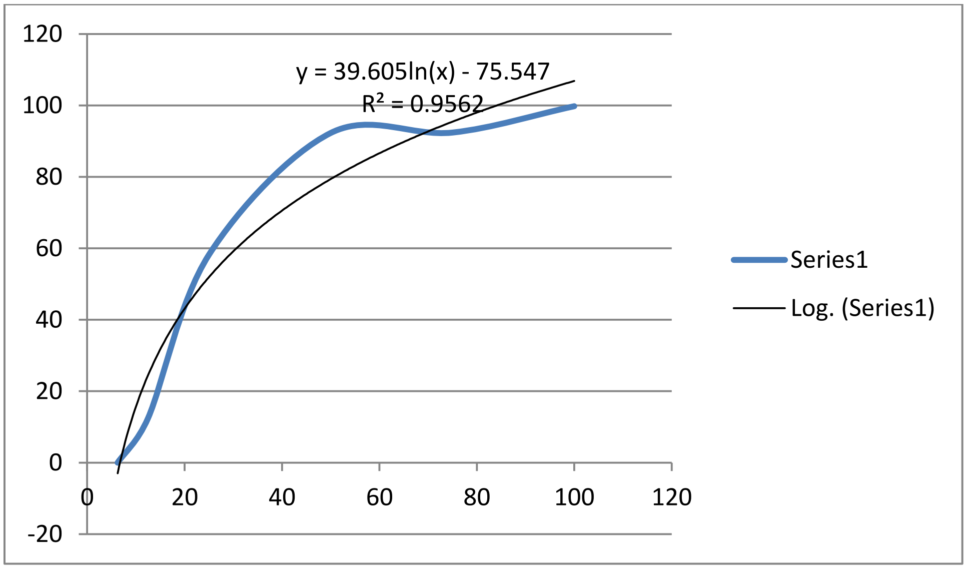

Second read									
			**6.25**	**12.5**	**25**	**50**	**75**	**100**
**Time**	**Control**	**tacrine**	**a**	**b**	**c**	**d**	**e**	**f**
0	0.073	0.053	0.070	0.071	0.060	0.064	0.067	0.063
1	0.083	0.055	0.080	0.080	0.063	0.066	0.068	0.065
2	0.091	0.057	0.090	0.088	0.065	0.068	0.069	0.066
3	0.101	0.058	0.098	0.098	0.067	0.070	0.070	0.068
4	0.108	0.060	0.108	0.105	0.069	0.071	0.071	0.068
5	0.116	0.062	0.117	0.112	0.071	0.073	0.072	0.070
6	0.126	0.063	0.126	0.119	0.072	0.074	0.073	0.071
7	0.130	0.065	0.126	0.127	0.074	0.076	0.073	0.072
8	0.139	0.067	0.139	0.130	0.076	0.077	0.076	0.073
9	0.147	0.069	0.141	0.139	0.078	0.079	0.075	0.075
10	0.151	0.070	0.144	0.144	0.079	0.080	0.075	0.076
**Gradient**	**0.0079**	**0.0017**	**0.0076**	**0.0073**	**0.0019**	**0.0016**	**0.0009**	**0.0012**
**% inhibition**		**78.3493**	**3.8388**	**7.98**	**76.39**	**79.96**	**88.83**	**84.22**

**conc**	**% inhibition**
6.25	3.8388
12.5	7.98
25	76.39
50	79.96
75	88.83
100	84.22

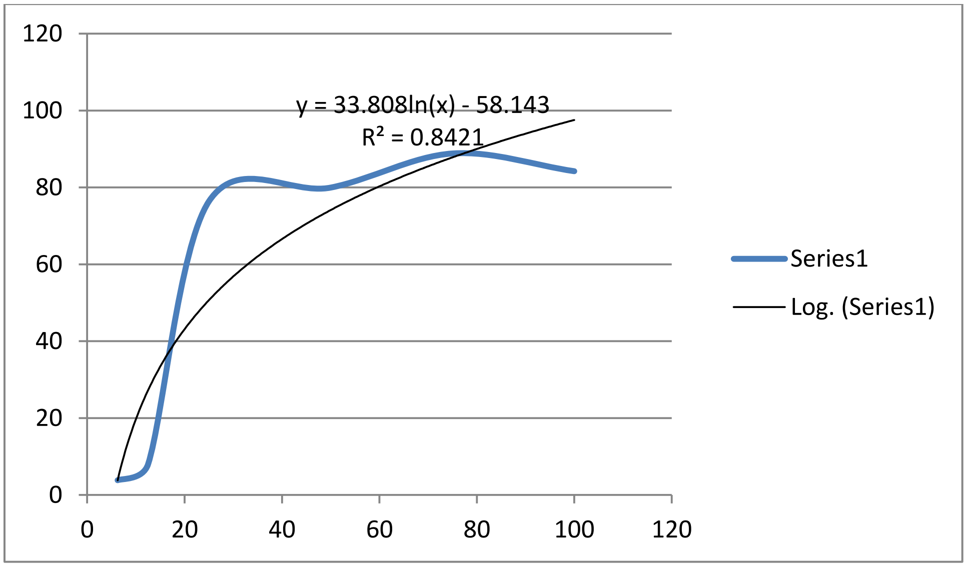

Third read								
			**6.25**	**12.5**	**25**	**50**	**75**	**100**
**Time**	**Control**	**tacrine**	**a**	**b**	**c**	**d**	**e**	**f**
0	0.073	0.053	0.069	0.068	0.062	0.059	0.061	0.067
1	0.083	0.055	0.078	0.075	0.065	0.060	0.062	0.066
2	0.091	0.056	0.086	0.083	0.069	0.062	0.062	0.065
3	0.101	0.057	0.095	0.089	0.071	0.062	0.062	0.065
4	0.108	0.058	0.104	0.098	0.073	0.062	0.063	0.065
5	0.116	0.059	0.110	0.101	0.075	0.062	0.063	0.063
6	0.126	0.061	0.116	0.105	0.079	0.063	0.062	0.062
7	0.130	0.062	0.123	0.112	0.079	0.063	0.063	0.063
8	0.139	0.064	0.134	0.113	0.080	0.064	0.064	0.062
9	0.147	0.064	0.136	0.118	0.085	0.065	0.064	0.062
10	0.151	0.066	0.145	0.122	0.084	0.065	0.064	0.062
**Gradient**	**0.0079**	**0.0013**	**0.0075**	**0.0053**	**0.0025**	**0.0005**	**0.0003**	**-0.0005**
**% inhibition**		**84.11**	**5.11**	**32.63**	**68.79**	**93.21**	**96.55**	**106.56**

**conc**	**% inhibition**
6.25	5.11
12.5	32.63
25	68.79
50	93.21
75	96.55
100	100.00

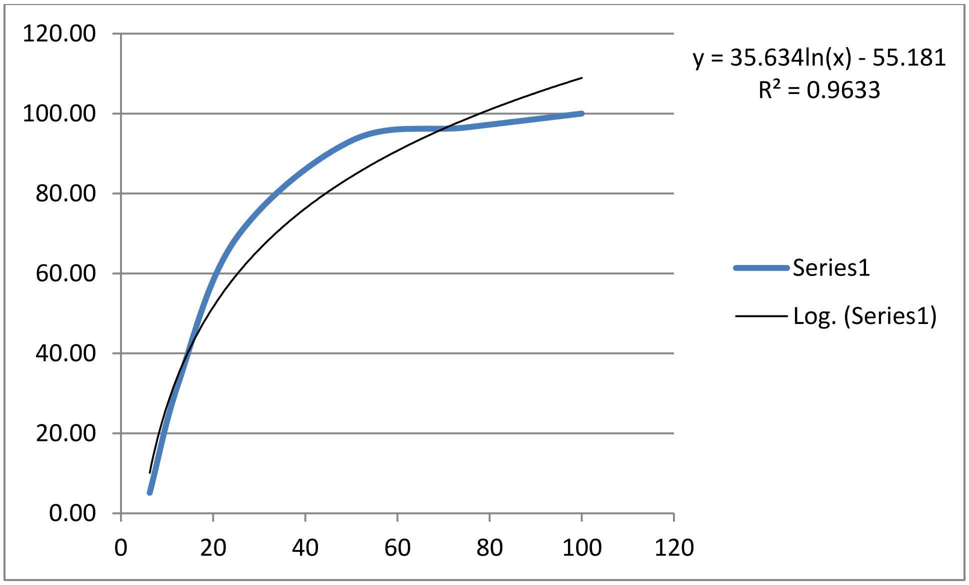

3.1.6 Raw data for IC_{50} calculation of compound **2i**.

First read									
			**25**	**50**	**75**	**100**	**125**	**150**	**200**
**Time**	**Control**	**tacrine**	**b**	**c**	**d**	**e**	**f**	**e**	**f**
0	0.068	0.049	0.074	0.095	0.117	0.134	0.149	0.179	0.201
1	0.071	0.050	0.078	0.098	0.118	0.137	0.152	0.183	0.205
2	0.075	0.050	0.083	0.103	0.122	0.140	0.151	0.182	0.208
3	0.078	0.051	0.086	0.106	0.124	0.142	0.152	0.181	0.209
4	0.081	0.051	0.090	0.109	0.126	0.145	0.155	0.183	0.208
5	0.084	0.052	0.093	0.111	0.128	0.145	0.154	0.183	0.209
6	0.088	0.052	0.095	0.112	0.129	0.145	0.153	0.184	0.207
7	0.089	0.053	0.099	0.116	0.129	0.146	0.155	0.183	0.208
8	0.091	0.053	0.102	0.117	0.131	0.146	0.154	0.183	0.207
9	0.093	0.055	0.102	0.120	0.134	0.149	0.154	0.183	0.206
10	0.096	0.054	0.104	0.119	0.134	0.150	0.156	0.182	0.203
**Gradient**	**0.0028**	**0.0005**	**0.0030**	**0.0025**	**0.0017**	**0.0014**	**0.0005**	**0.0002**	**0.0001**
**% inhibition**		**80.61**	**-9.75**	**10.30**	**37.90**	**49.40**	**81.60**	**92.44**	**97.37**

**conc**	**% inhibition**
25	0.0000
50	10.2957
75	37.90
100	49.3976
125	81.60
150	92.44
200	97.37

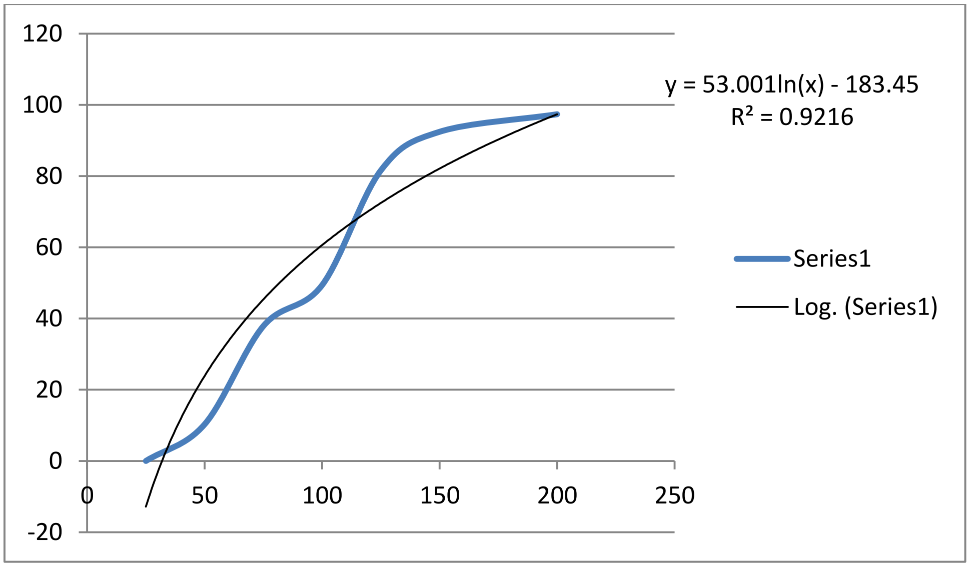

Second read									
			**25**	**50**	**75**	**100**	**125**	**150**	**200**
**Time**	**Control**	**tacrine**	**b**	**c**	**d**	**e**	**f**	**e**	**f**
0	0.068	0.049	0.075	0.096	0.124	0.145	0.162	0.185	0.208
1	0.071	0.049	0.078	0.099	0.125	0.141	0.163	0.187	0.212
2	0.075	0.049	0.081	0.104	0.126	0.145	0.163	0.187	0.211
3	0.078	0.050	0.084	0.106	0.128	0.146	0.162	0.188	0.21
4	0.081	0.050	0.087	0.109	0.129	0.147	0.162	0.189	0.211
5	0.084	0.051	0.089	0.111	0.129	0.147	0.162	0.188	0.209
6	0.088	0.051	0.093	0.112	0.132	0.146	0.161	0.188	0.21
7	0.089	0.052	0.094	0.116	0.134	0.150	0.164	0.188	0.209
8	0.091	0.052	0.098	0.117	0.134	0.148	0.163	0.189	0.211
9	0.093	0.052	0.100	0.120	0.133	0.150	0.165	0.189	0.21
10	0.096	0.053	0.101	0.119	0.135	0.149	0.163	0.189	0.208
**Gradient**	**0.0028**	**0.0004**	**0.0027**	**0.0024**	**0.0011**	**0.0007**	**0.0001**	**0.0003**	**-0.0001**
**% inhibition**		**84.8850**	**3.07**	**14.24**	**58.60**	**76.34**	**94.74**	**89.16**	**103.61**

**conc**	**% inhibition**
25	3.07
50	14.24
75	58.60
100	76.34
125	94.74
150	89.16
200	100.00

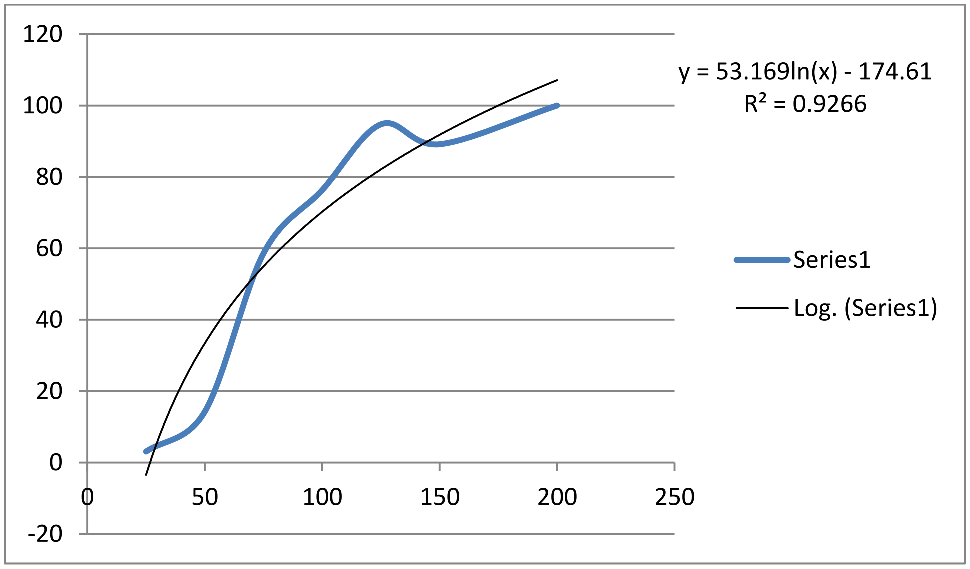

Third read									
			**25**	**50**	**75**	**100**	**125**	**150**	**200**
**Time**	**Control**	**tacrine**	**b**	**c**	**d**	**e**	**f**	**e**	**f**
0	0.068	0.048	0.074	0.094	0.117	0.138	0.155	0.173	0.211
1	0.071	0.049	0.078	0.098	0.121	0.141	0.153	0.174	0.209
2	0.075	0.050	0.081	0.101	0.125	0.141	0.156	0.176	0.211
3	0.078	0.050	0.084	0.104	0.128	0.144	0.157	0.176	0.212
4	0.081	0.051	0.087	0.106	0.131	0.145	0.158	0.177	0.211
5	0.084	0.051	0.087	0.109	0.132	0.144	0.157	0.177	0.212
6	0.088	0.052	0.091	0.110	0.133	0.144	0.159	0.178	0.213
7	0.089	0.052	0.095	0.115	0.136	0.146	0.159	0.179	0.21
8	0.091	0.053	0.097	0.116	0.134	0.148	0.161	0.18	0.21
9	0.093	0.053	0.099	0.119	0.135	0.148	0.161	0.176	0.21
10	0.096	0.054	0.100	0.120	0.136	0.148	0.160	0.177	0.21
**Gradient**	**0.0028**	**0.0005**	**0.0026**	**0.0026**	**0.0018**	**0.0009**	**0.0007**	**0.0004**	**-0.0001**
**% inhibition**		**80.28**	**5.37**	**6.35**	**35.60**	**66.48**	**74.70**	**84.56**	**101.97**

**conc**	**% inhibition**
25	5.37
50	6.35
75	35.60
100	66.48
125	74.70
150	84.55640745
200	100

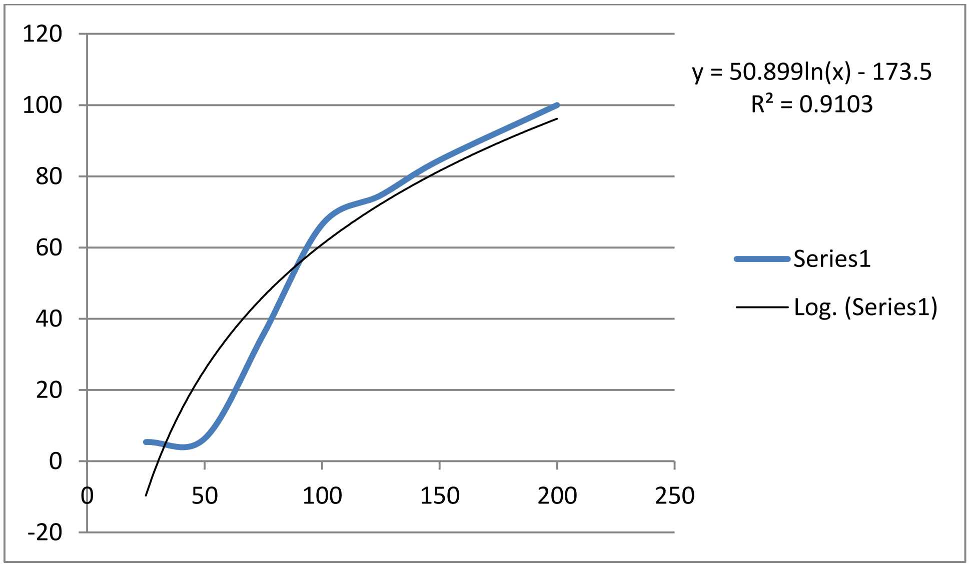

3.1.7 Summary of AChE IC_{50}.

IC_{50} (µg/mL)						
Compound	**2b**	**2c**	**2f**	**2g**	**2h**	**2i**
First read	191.8275	77.84214	61.54146	97.55264	23.80697	81.82918
Second read	171.9302	70.90097	53.00301	74.88399	24.50164	68.33719
Third read	160.4439	66.631	54.10931	73.34383	19.13853	80.72505
Average	174.7339	71.79137	56.21792	81.92682	22.48238	76.96381
S.D.	15.8785	5.658357	4.643383	13.55425	2.916616	7.491239

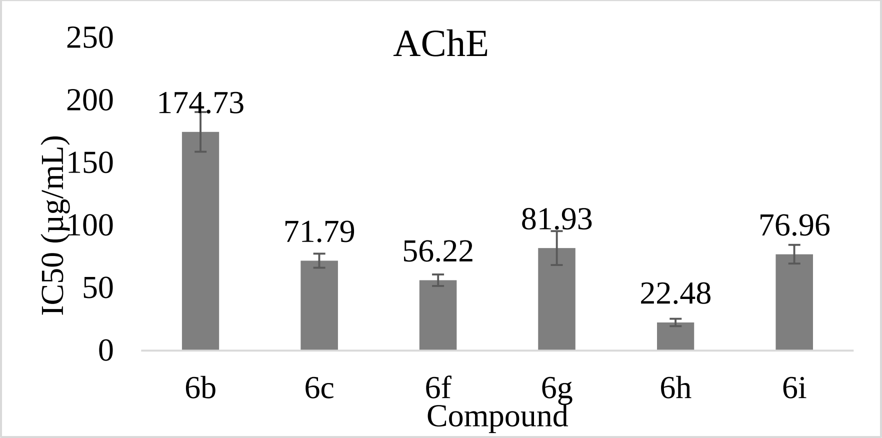

IC_{50} (µM)						
Compound	**2b**	**2c**	**2f**	**2g**	**2h**	**2i**
First read	721.1559	292.6396	227.9313	343.4952	83.82737	269.1749
Second read	646.354	266.545	196.3074	263.676	86.27337	224.7934
Third read	603.1727	250.4925	200.4048	258.2529	67.38918	265.5429
Average	656.8942	269.8924	208.2145	288.4747	79.16331	253.1704
S.D.	59.69362	21.27202	17.19771	47.72624	10.26978	24.64224

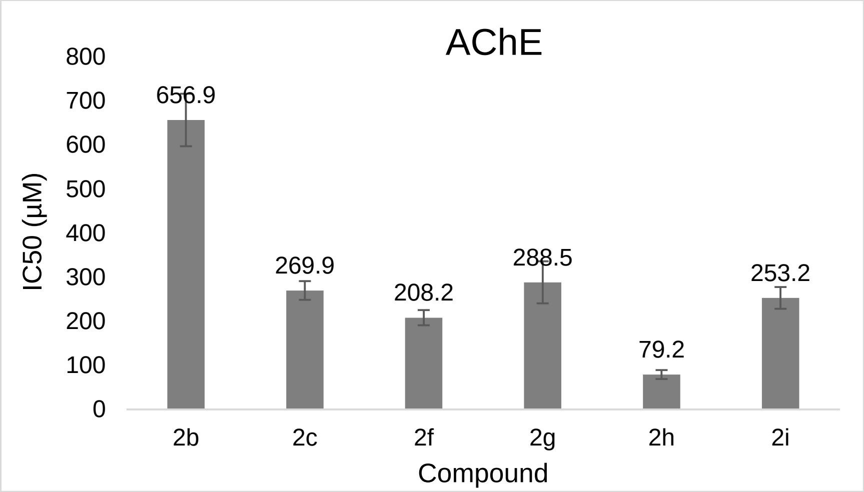

3.1.8 Statistical analysis for the IC_{50} of the test compound (AChE).

ANOVA

IC50

	Sum of Squares	df	Mean Square	F	Sig.
Between Groups	38777.565	5	7755.513	83.987	.000
Within Groups	1108.096	12	92.341		
Total	39885.661	17			

Homogeneous Subsets

IC50

Tukey HSD

Compound	N	Subset for alpha = 0.05		
		1	2	3
2h	3	22.4824		
2f	3		56.2179	
2c	3		71.7914	
2j	3		76.9638	
2g	3		81.9268	
2b	3			1.7473E2
Sig.		1.000	.057	1.000

Means for groups in homogeneous subsets are displayed.

3.2 BChE inhibition

3.2.1 Raw data for IC_{50} calculation of compound **2b**.

First read										
			**50**	**100**	**200**	**300**	**400**	**500**	**600**	**700**
**Time**	**Contro l**	**tacrin e**	**a**	**b**	**c**	**d**	**e**	**f**	**e**	**f**
0	0.057	0.047	0.053	0.053	0.053	0.051	0.051	0.049	0.061	0.064
1	0.059	0.047	0.055	0.055	0.054	0.051	0.052	0.050	0.062	0.064
2	0.062	0.047	0.058	0.057	0.056	0.052	0.052	0.050	0.063	0.065
3	0.064	0.047	0.060	0.059	0.057	0.053	0.053	0.051	0.063	0.065
4	0.066	0.047	0.062	0.061	0.058	0.054	0.054	0.051	0.063	0.066
5	0.070	0.047	0.064	0.062	0.059	0.055	0.054	0.052	0.064	0.066
6	0.072	0.047	0.066	0.064	0.060	0.056	0.055	0.052	0.064	0.066
7	0.074	0.048	0.067	0.066	0.061	0.057	0.055	0.053	0.065	0.067
8	0.076	0.048	0.069	0.067	0.063	0.057	0.056	0.053	0.065	0.067
9	0.078	0.048	0.070	0.069	0.064	0.058	0.057	0.053	0.065	0.068
10	0.081	0.048	0.072	0.070	0.065	0.059	0.058	0.054	0.066	0.068
**Gradient**	**0.0024**	**0.0001**	**0.001 9**	**0.001 7**	**0.001 2**	**0.000 8**	**0.000 7**	**0.000 5**	**0.000 4**	**0.000 4**
**% inhibition**		**94.64**	**21.17**	**28.06**	**49.87**	**64.41**	**72.45**	**80.48**	**81.63**	**82.40**

**conc**	**% inhibition**
50	21.1735
100	28.0612
200	49.8724
300	64.41
400	72.4490
500	80.48
600	81.63
700	82.40

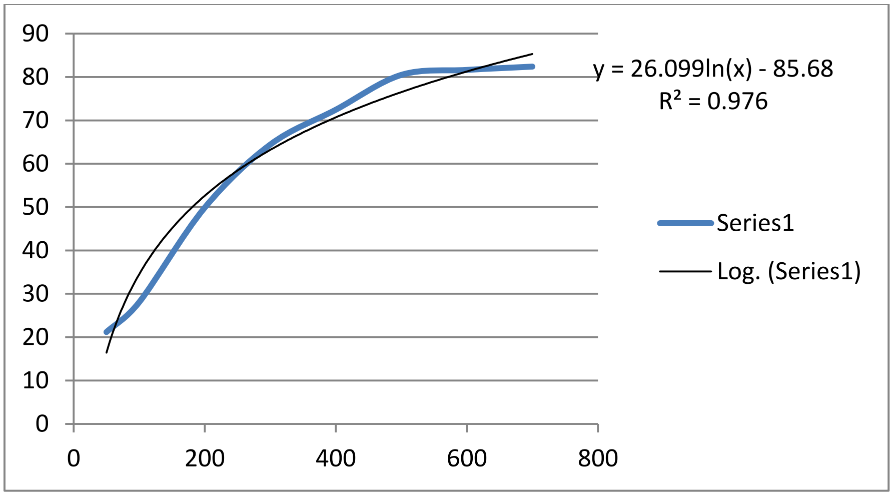

Second read										
			**50**	**100**	**200**	**300**	**400**	**500**	**600**	**700**
**Time**	**Control**	**tacrine**	**a**	**b**	**c**	**d**	**e**	**f**	**e**	**f**
0	0.057	0.051	0.053	0.053	0.050	0.049	0.052	0.050	0.069	0.07
1	0.059	0.051	0.056	0.055	0.052	0.050	0.052	0.050	0.07	0.071
2	0.062	0.051	0.058	0.057	0.053	0.051	0.053	0.051	0.07	0.071
3	0.064	0.051	0.060	0.059	0.055	0.052	0.054	0.051	0.071	0.071
4	0.066	0.051	0.062	0.061	0.056	0.053	0.054	0.052	0.072	0.072
5	0.070	0.051	0.064	0.063	0.058	0.054	0.055	0.052	0.072	0.073
6	0.072	0.052	0.066	0.065	0.059	0.055	0.056	0.053	0.073	0.073
7	0.074	0.052	0.068	0.066	0.060	0.056	0.056	0.054	0.073	0.073
8	0.076	0.052	0.070	0.067	0.061	0.057	0.057	0.054	0.074	0.073
9	0.078	0.052	0.071	0.069	0.062	0.057	0.058	0.055	0.074	0.073
10	0.081	0.052	0.073	0.070	0.063	0.058	0.058	0.056	0.074	0.074
**Gradient**	**0.0024**	**0.0001**	**0.0020**	**0.0017**	**0.0013**	**0.0009**	**0.0007**	**0.0006**	**0.0005**	**0.0004**
**% inhibition**		**94.2602**	**17.3469**	**27.68**	**45.66**	**61.35**	**72.45**	**74.74**	**77.81**	**85.08**

conc	% inhibition
50	17.3469
100	27.68
200	45.66
300	61.35
400	72.45
500	74.74
600	77.81
700	85.08

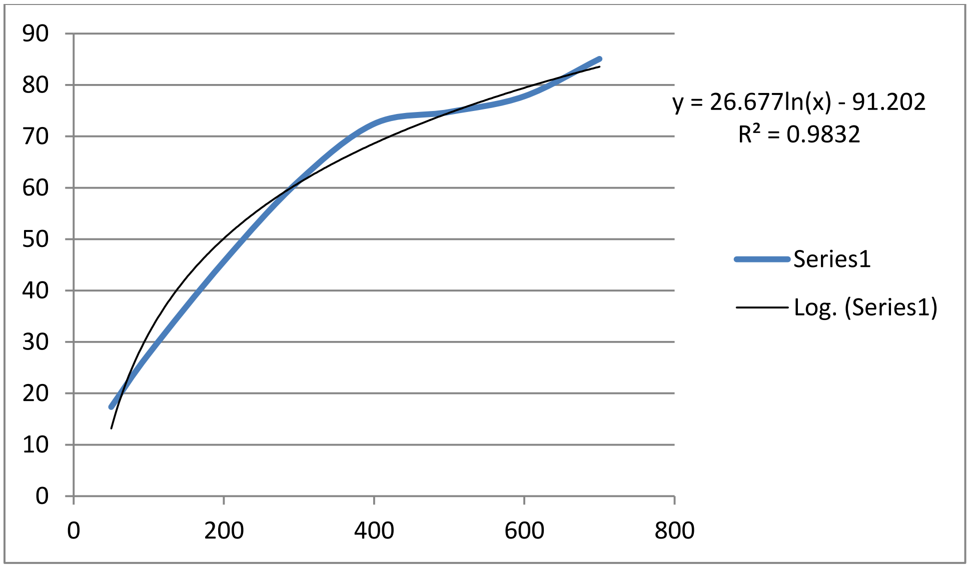

Third read											
			**50**	**100**	**200**	**300**	**400**	**500**	**600**	**700**
**Time**	**Control**	**tacrine**	**a**	**b**	**c**	**d**	**e**	**f**	**e**	**f**
0	0.057	0.049	0.052	0.051	0.051	0.050	0.051	0.053	0.075	0.085
1	0.059	0.049	0.055	0.053	0.053	0.051	0.052	0.053	0.075	0.083
2	0.062	0.049	0.057	0.055	0.054	0.052	0.052	0.053	0.074	0.086
3	0.064	0.049	0.059	0.057	0.056	0.053	0.053	0.054	0.073	0.084
4	0.066	0.049	0.061	0.058	0.057	0.053	0.053	0.055	0.076	0.085
5	0.070	0.049	0.064	0.060	0.059	0.054	0.054	0.055	0.077	0.086
6	0.072	0.049	0.065	0.061	0.060	0.055	0.055	0.056	0.078	0.084
7	0.074	0.049	0.067	0.063	0.061	0.056	0.055	0.057	0.073	0.085
8	0.076	0.049	0.068	0.065	0.062	0.056	0.056	0.057	0.076	0.086
9	0.078	0.049	0.070	0.066	0.063	0.057	0.056	0.058	0.079	0.088
10	0.081	0.049	0.072	0.068	0.065	0.058	0.057	0.059	0.078	0.087
**Gradient**	**0.0024**	**0.0000**	**0.0019**	**0.0017**	**0.0013**	**0.0008**	**0.0006**	**0.0006**	**0.0004**	**0.0003**
**% inhibition**		**100.00**	**18.49**	**30.36**	**43.75**	**67.86**	**75.51**	**73.60**	85.08	88.14

**conc**	**% inhibition**
50	18.49
100	30.36
200	43.75
300	67.86
400	75.51
500	73.60
600	85.07653061
700	88.1377551

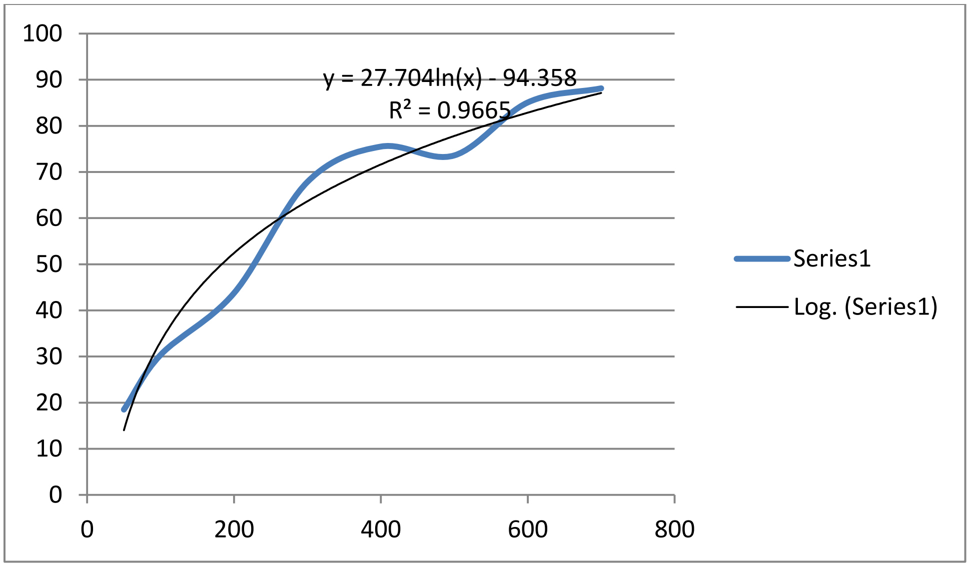

3.2.2 Raw data for IC_{50} calculation of compound **2g**.

First read										
			**50**	**60**	**70**	**80**	**90**	**100**	**110**	**120**
**Time**	**Control**	**tacrine**	**a**	**b**	**c**	**d**	**e**	**f**	**e**	**f**
0	0.055	0.051	0.086	0.095	0.104	0.109	0.116	0.131	0.139	0.135
1	0.057	0.051	0.089	0.097	0.106	0.110	0.114	0.129	0.137	0.138
2	0.059	0.051	0.090	0.099	0.108	0.111	0.115	0.132	0.138	0.137
3	0.061	0.051	0.092	0.099	0.107	0.113	0.120	0.132	0.133	0.14
4	0.063	0.051	0.092	0.103	0.111	0.116	0.117	0.132	0.14	0.14
5	0.065	0.051	0.095	0.102	0.110	0.117	0.118	0.133	0.136	0.136
6	0.066	0.051	0.095	0.105	0.112	0.116	0.118	0.134	0.137	0.134
7	0.067	0.051	0.096	0.107	0.112	0.117	0.119	0.136	0.139	0.133
8	0.068	0.051	0.099	0.108	0.116	0.118	0.124	0.133	0.14	0.139
9	0.070	0.051	0.099	0.108	0.116	0.118	0.121	0.135	0.14	0.136
10	0.071	0.051	0.100	0.109	0.119	0.119	0.123	0.137	0.139	0.137
**Gradient**	**0.0016**	**0.0000**	**0.0013**	**0.0014**	**0.0014**	**0.0010**	**0.0008**	**0.0006**	**0.0002**	**-0.0001**
**% inhibition**		**100.00**	**14.12**	**7.74**	**12.96**	**35.59**	**48.36**	**61.12**	**84.33**	**106.96**

**conc**	**% inhibition**
50	14.1199
60	7.7369
70	12.9594
80	35.59
90	48.3559
100	61.12
110	84.33
120	100.00

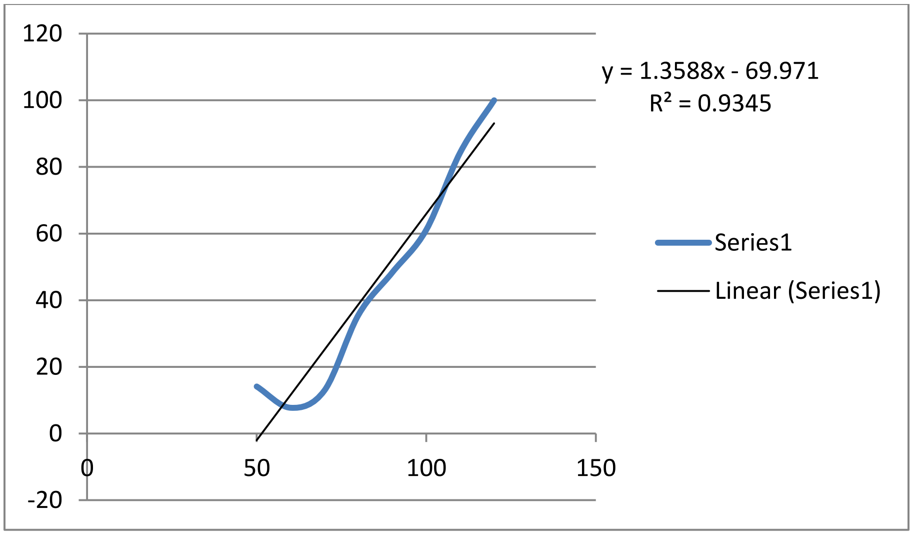

Second read										
			**50**	**60**	**70**	**80**	**90**	**100**	**110**	**120**
**Time**	**Control**	**tacrine**	**a**	**b**	**c**	**d**	**e**	**f**	**e**	**f**
0	0.055	0.050	0.089	0.097	0.101	0.109	0.115	0.127	0.139	0.142
1	0.057	0.050	0.089	0.099	0.098	0.110	0.114	0.124	0.14	0.141
2	0.059	0.050	0.093	0.102	0.102	0.111	0.115	0.123	0.142	0.142
3	0.061	0.050	0.095	0.101	0.103	0.111	0.115	0.125	0.141	0.14
4	0.063	0.050	0.096	0.104	0.105	0.112	0.117	0.125	0.143	0.147
5	0.065	0.050	0.097	0.104	0.109	0.111	0.117	0.127	0.146	0.141
6	0.066	0.050	0.100	0.108	0.109	0.114	0.121	0.127	0.144	0.142
7	0.067	0.051	0.101	0.109	0.108	0.115	0.121	0.125	0.14	0.145
8	0.068	0.051	0.105	0.110	0.112	0.115	0.120	0.127	0.146	0.143
9	0.070	0.050	0.104	0.111	0.111	0.118	0.123	0.128	0.142	0.144
10	0.071	0.051	0.106	0.113	0.112	0.117	0.124	0.130	0.147	0.146
**Gradient**	**0.0016**	**0.0001**	**0.0018**	**0.0016**	**0.0014**	**0.0009**	**0.0010**	**0.0004**	**0.0005**	**0.0004**
**% inhibition**		**94.1973**	**-14.3133**	**0.19**	**12.38**	**45.45**	**35.01**	**73.89**	**65.76**	**76.79**

**conc**	**% inhibition**
50	0
60	0.19
70	12.38
80	45.45
90	35.01
100	73.89
110	65.76
120	100.00

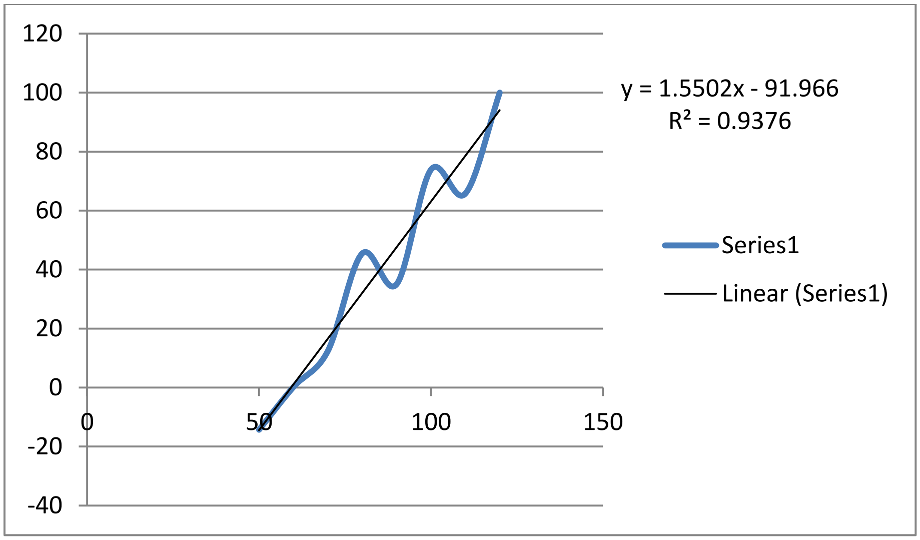

Third read										
			**50**	**60**	**70**	**80**	**90**	**100**	**110**	**120**
**Time**	**Control**	**tacrine**	**a**	**b**	**c**	**d**	**e**	**f**	**e**	**f**
0	0.055	0.051	0.087	0.093	0.103	0.109	0.116	0.122	0.129	0.143
1	0.057	0.051	0.087	0.096	0.103	0.110	0.114	0.122	0.131	0.142
2	0.059	0.051	0.091	0.095	0.103	0.111	0.115	0.121	0.129	0.138
3	0.061	0.051	0.090	0.096	0.104	0.113	0.120	0.122	0.129	0.14
4	0.063	0.051	0.094	0.099	0.106	0.119	0.119	0.122	0.13	0.139
5	0.065	0.051	0.096	0.099	0.109	0.117	0.118	0.124	0.133	0.139
6	0.066	0.051	0.098	0.101	0.109	0.116	0.118	0.123	0.132	0.14
7	0.067	0.051	0.098	0.102	0.109	0.117	0.117	0.126	0.132	0.139
8	0.068	0.051	0.099	0.106	0.112	0.118	0.124	0.125	0.133	0.138
9	0.070	0.051	0.101	0.106	0.112	0.118	0.121	0.126	0.134	0.138
10	0.071	0.051	0.101	0.108	0.111	0.119	0.123	0.126	0.134	0.139
**Gradient**	**0.0016**	**0.0000**	**0.0015**	**0.0015**	**0.0011**	**0.0010**	**0.0008**	**0.0005**	0.0005	-0.0003
**% inhibition**		**100.00**	**1.35**	**6.00**	**32.69**	**37.33**	**51.84**	**66.92**	66.92	121.47

**conc**	**% inhibition**
50	1.35
60	6.00
70	32.69
80	37.33
90	51.84
100	66.92
110	66.9245648
120	100

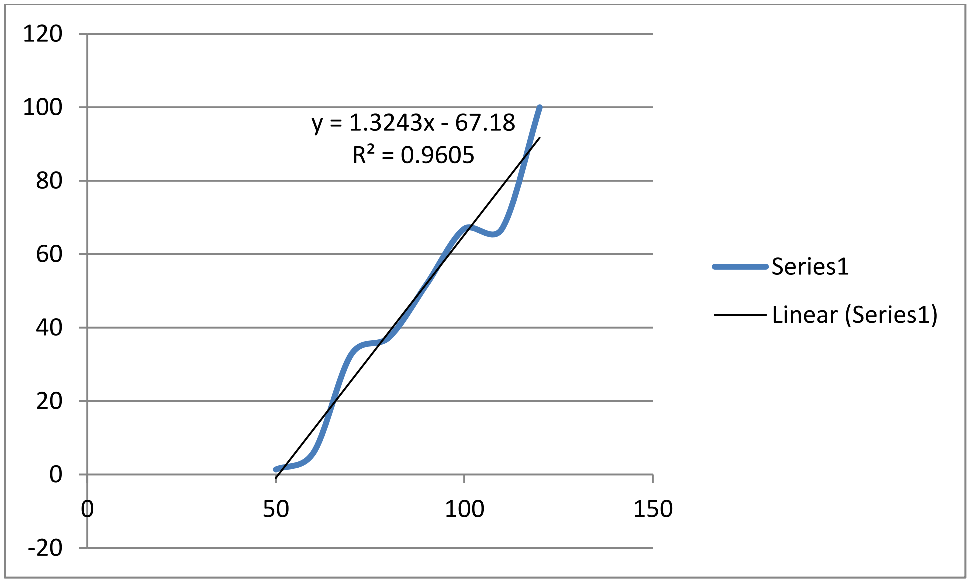

3.2.3 Raw data for IC_{50} calculation of compound **2h**.

First read									
			**25**	**50**	**100**	**150**	**200**	**250**
**Time**	**Control**	**tacrine**	**a**	**b**	**c**	**d**	**e**	**f**
0	0.053	0.048	0.060	0.057	0.059	0.064	0.065	0.070
1	0.055	0.048	0.062	0.059	0.060	0.065	0.066	0.069
2	0.057	0.048	0.064	0.060	0.061	0.066	0.067	0.069
3	0.059	0.048	0.067	0.061	0.063	0.067	0.067	0.069
4	0.061	0.049	0.069	0.063	0.065	0.068	0.067	0.069
5	0.063	0.049	0.071	0.065	0.066	0.069	0.067	0.069
6	0.065	0.049	0.072	0.066	0.067	0.070	0.068	0.069
7	0.067	0.049	0.075	0.067	0.069	0.071	0.068	0.069
8	0.069	0.049	0.076	0.068	0.071	0.073	0.068	0.069
9	0.069	0.049	0.078	0.069	0.072	0.072	0.068	0.069
10	0.070	0.049	0.080	0.070	0.073	0.073	0.069	0.069
**Gradient**	**0.0018**	**0.0001**	**0.0020**	**0.0013**	**0.0015**	**0.0009**	**0.0003**	**0.0000**
**% inhibition**		**93.01**	**-9.32**	**28.12**	**19.13**	**48.09**	**83.03**	**102.50**

**conc**	**% inhibition**
25	0.0000
50	28.1198
100	19.1348
150	48.09
200	83.0283
250	100.00

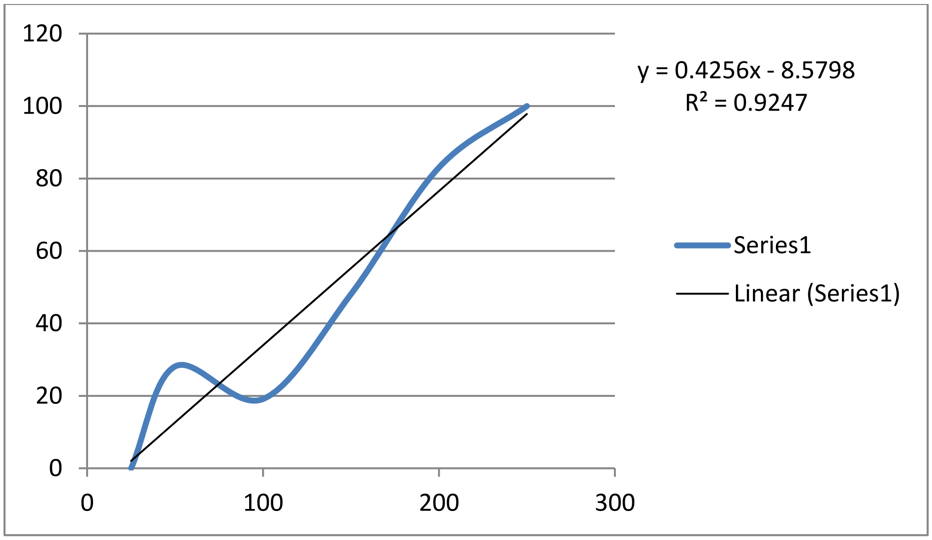

Second read									
			**25**	**50**	**100**	**150**	**200**	**250**
**Time**	**Control**	**tacrine**	**a**	**b**	**c**	**d**	**e**	**f**
0	0.053	0.049	0.060	0.056	0.058	0.064	0.066	0.070
1	0.055	0.049	0.062	0.058	0.060	0.065	0.067	0.070
2	0.057	0.049	0.064	0.059	0.061	0.066	0.067	0.070
3	0.059	0.049	0.066	0.061	0.063	0.067	0.067	0.069
4	0.061	0.049	0.067	0.063	0.064	0.068	0.068	0.069
5	0.063	0.049	0.068	0.064	0.065	0.069	0.068	0.069
6	0.065	0.049	0.070	0.066	0.067	0.070	0.068	0.068
7	0.067	0.049	0.071	0.067	0.068	0.071	0.069	0.068
8	0.069	0.049	0.073	0.068	0.069	0.072	0.069	0.068
9	0.069	0.049	0.075	0.070	0.071	0.073	0.069	0.068
10	0.070	0.049	0.077	0.070	0.071	0.074	0.070	0.067
**Gradient**	**0.0018**	**0.0000**	**0.0016**	**0.0015**	**0.0013**	**0.0010**	**0.0003**	**-0.0003**
**% inhibition**		**100.0000**	**11.6473**	**20.13**	**27.12**	**45.09**	**81.03**	**115.97**

**conc**	**% inhibition**
25	11.6473
50	20.13
100	27.12
150	45.09
200	81.03
250	100.00

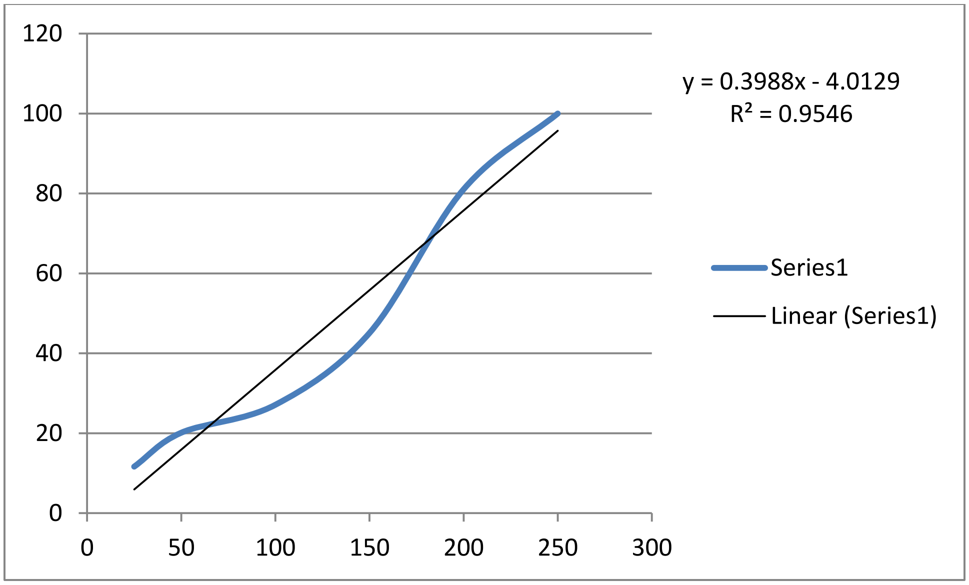

Third read									
			**25**	**50**	**100**	**150**	**200**	**250**
**Time**	**Control**	**tacrine**	**a**	**b**	**c**	**d**	**e**	**f**
0	0.053	0.049	0.059	0.056	0.057	0.063	0.062	0.066
1	0.055	0.049	0.061	0.058	0.058	0.065	0.062	0.065
2	0.057	0.049	0.063	0.059	0.060	0.065	0.063	0.065
3	0.059	0.049	0.065	0.061	0.062	0.067	0.063	0.065
4	0.061	0.049	0.067	0.062	0.063	0.067	0.064	0.066
5	0.063	0.049	0.069	0.064	0.065	0.069	0.064	0.067
6	0.065	0.049	0.071	0.066	0.066	0.069	0.064	0.066
7	0.067	0.049	0.072	0.066	0.068	0.070	0.064	0.067
8	0.069	0.049	0.073	0.068	0.069	0.072	0.065	0.067
9	0.069	0.049	0.074	0.070	0.070	0.072	0.066	0.067
10	0.070	0.049	0.077	0.070	0.072	0.072	0.067	0.068
**Gradient**	**0.0018**	**0.0000**	**0.0017**	**0.0014**	**0.0015**	**0.0009**	**0.0004**	**0.0003**
**% inhibition**		**100.00**	**5.16**	**20.63**	**17.64**	**49.08**	**75.54**	**86.02**

**conc**	**% inhibition**
25	5.16
50	20.63
100	17.64
150	49.08
200	75.54
250	86.02

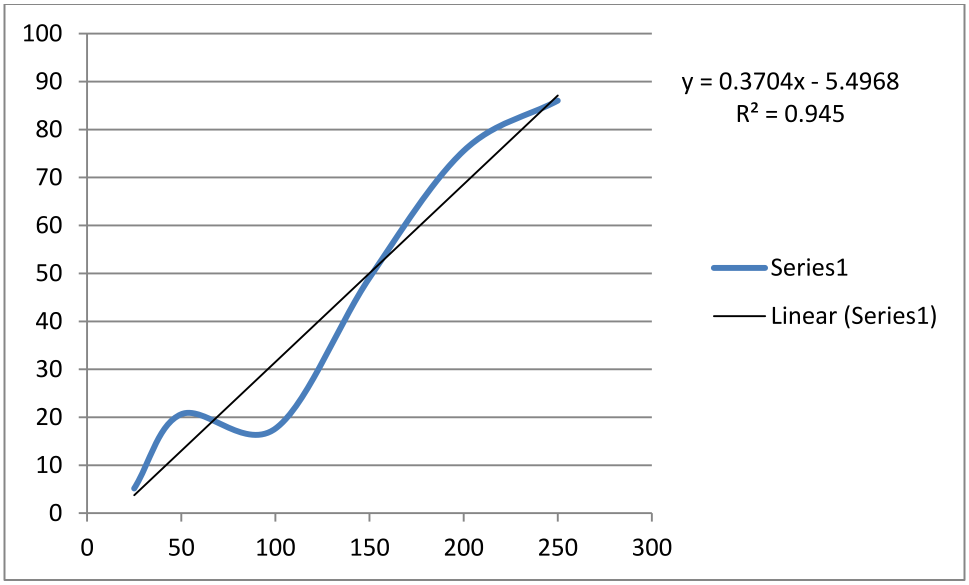

3.2.4 Raw data for IC_{50} calculation of compound **2j**.

First read									
			**3.125**	**6.25**	**12.5**	**25**	**50**	**100**
**Time**	**Control**	**tacrine**	**a**	**b**	**c**	**d**	**e**	**f**
0	0.060	0.052	0.056	0.055	0.059	0.058	0.053	0.051
1	0.064	0.052	0.060	0.058	0.062	0.061	0.054	0.052
2	0.067	0.052	0.063	0.061	0.065	0.063	0.056	0.052
3	0.070	0.052	0.068	0.063	0.067	0.064	0.057	0.052
4	0.074	0.052	0.070	0.065	0.070	0.066	0.058	0.053
5	0.077	0.052	0.073	0.068	0.072	0.067	0.059	0.053
6	0.080	0.052	0.077	0.070	0.074	0.069	0.062	0.053
7	0.083	0.052	0.080	0.073	0.077	0.070	0.061	0.053
8	0.086	0.052	0.082	0.074	0.078	0.072	0.063	0.054
9	0.088	0.052	0.086	0.077	0.080	0.073	0.064	0.054
10	0.091	0.052	0.087	0.078	0.082	0.074	0.064	0.054
**Gradient**	**0.0031**	**0.0000**	**0.0032**	**0.0023**	**0.0023**	**0.0015**	**0.0012**	**0.0003**
**% inhibition**		**100.00**	**-0.29**	**26.30**	**27.75**	**50.87**	**63.01**	**91.04**

**conc**	**% inhibition**
3.125	0.0000
6.25	26.3006
12.5	27.7457
25	50.87
50	63.0058
100	91.04

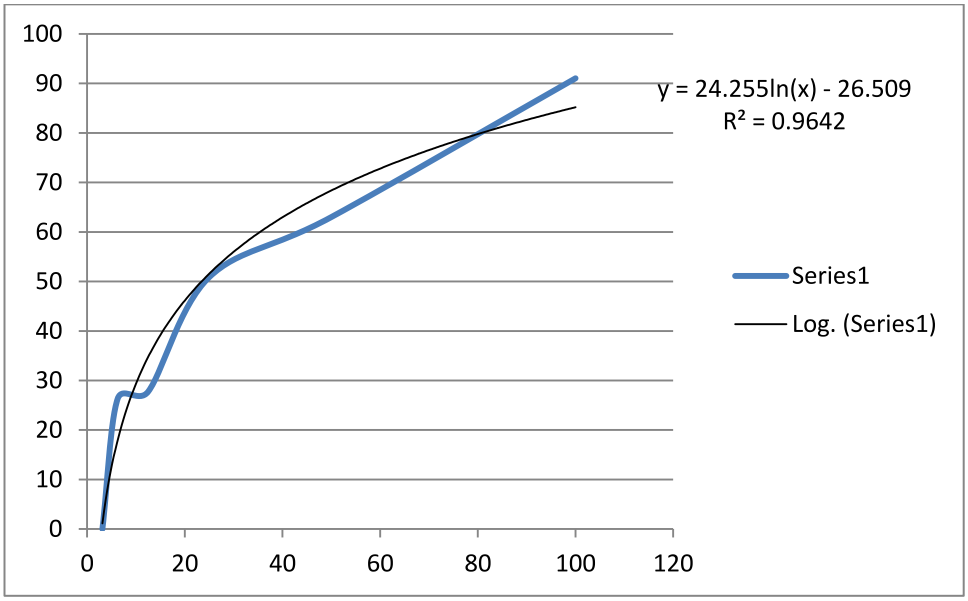

Second read									
			**3.125**	**6.25**	**12.5**	**25**	**50**	**100**
**Time**	**Control**	**tacrine**	**a**	**b**	**c**	**d**	**e**	**f**
0	0.060	0.053	0.055	0.055	0.053	0.055	0.054	0.053
1	0.064	0.053	0.058	0.058	0.055	0.056	0.055	0.054
2	0.067	0.053	0.061	0.060	0.056	0.057	0.056	0.054
3	0.070	0.053	0.064	0.063	0.058	0.059	0.057	0.054
4	0.074	0.053	0.066	0.065	0.059	0.060	0.058	0.055
5	0.077	0.053	0.069	0.067	0.061	0.061	0.059	0.055
6	0.080	0.053	0.072	0.069	0.063	0.063	0.061	0.055
7	0.083	0.053	0.074	0.070	0.064	0.064	0.061	0.056
8	0.086	0.053	0.077	0.073	0.066	0.066	0.062	0.056
9	0.088	0.053	0.078	0.074	0.068	0.067	0.064	0.057
10	0.091	0.053	0.080	0.076	0.069	0.069	0.064	0.057
**Gradient**	**0.0031**	**0.0000**	**0.0025**	**0.0021**	**0.0016**	**0.0014**	**0.0010**	**0.0004**
**% inhibition**		**100.0000**	**19.3642**	**34.68**	**48.55**	**55.49**	**66.76**	**87.86**

**conc**	**% inhibition**
3.125	19.3642
6.25	34.68
12.5	48.55
25	55.49
50	66.76
100	87.86

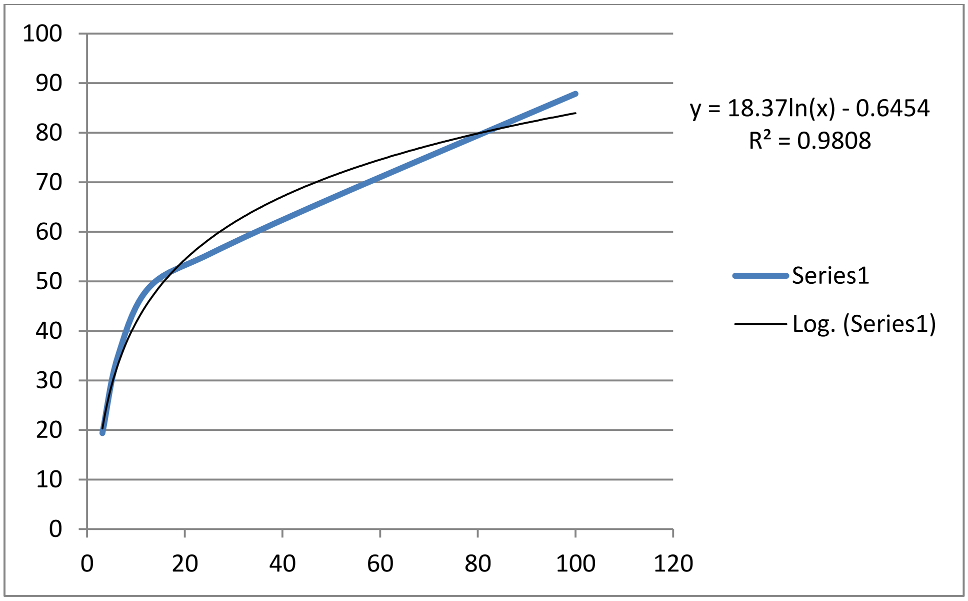

Third read								
			**3.125**	**6.25**	**12.5**	**25**	**50**	**100**
**Time**	**Control**	**tacrine**	**a**	**b**	**c**	**d**	**e**	**f**
0	0.060	0.054	0.055	0.053	0.055	0.052	0.053	0.056
1	0.064	0.055	0.059	0.056	0.058	0.053	0.054	0.056
2	0.067	0.056	0.063	0.058	0.061	0.055	0.055	0.056
3	0.070	0.055	0.067	0.060	0.063	0.056	0.056	0.056
4	0.074	0.055	0.070	0.063	0.066	0.057	0.057	0.057
5	0.077	0.054	0.073	0.065	0.068	0.058	0.058	0.057
6	0.080	0.053	0.075	0.069	0.071	0.059	0.059	0.058
7	0.083	0.053	0.079	0.072	0.072	0.060	0.060	0.058
8	0.086	0.053	0.081	0.074	0.074	0.062	0.061	0.058
9	0.088	0.053	0.083	0.076	0.076	0.063	0.062	0.058
10	0.091	0.053	0.086	0.079	0.078	0.064	0.062	0.059
**Gradient**	**0.0031**	**-0.0003**	**0.0030**	**0.0026**	**0.0021**	**0.0012**	**0.0010**	**0.0003**
**% inhibition**		**108.09**	**3.47**	**16.76**	**34.78**	**62.14**	**69.65**	**90.17**

**conc**	**% inhibition**
3.125	3.47
6.25	16.76
12.5	34.78
25	62.14
50	69.65
100	90.17

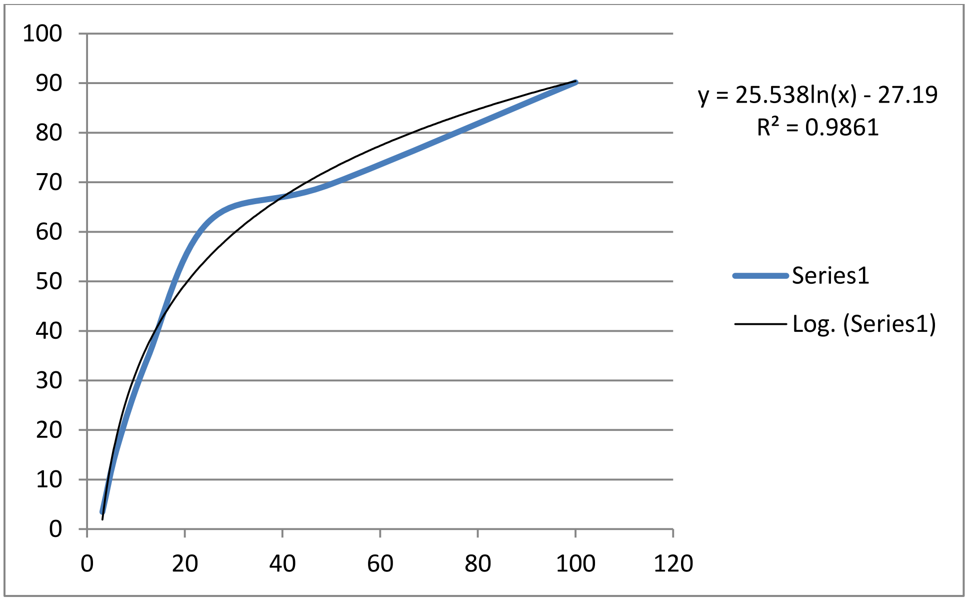

3.2.5 Raw data for IC_{50} calculation of compound **2k**.

First read								
			**12.5**	**25**	**50**	**100**	**150**	**200**
**Time**	**Control**	**tacrine**	**a**	**b**	**c**	**d**	**e**	**f**
0	0.052	0.049	0.050	0.051	0.051	0.069	0.099	0.105
1	0.054	0.049	0.052	0.052	0.052	0.069	0.095	0.106
2	0.056	0.049	0.054	0.054	0.053	0.068	0.094	0.109
3	0.058	0.049	0.056	0.056	0.054	0.069	0.096	0.106
4	0.060	0.049	0.058	0.057	0.055	0.069	0.094	0.107
5	0.062	0.049	0.059	0.059	0.056	0.070	0.097	0.106
6	0.063	0.049	0.061	0.061	0.057	0.071	0.095	0.107
7	0.065	0.049	0.063	0.062	0.058	0.071	0.102	0.113
8	0.067	0.049	0.064	0.064	0.058	0.073	0.096	0.111
9	0.068	0.049	0.066	0.066	0.059	0.074	0.098	0.111
10	0.069	0.049	0.067	0.067	0.061	0.073	0.100	0.112
**Gradient**	**0.0017**	**0.0000**	**0.0017**	**0.0017**	**0.0009**	**0.0006**	**0.0003**	**0.0007**
**% inhibition**		**100.00**	**-1.99**	**1.27**	**44.12**	**66.91**	**80.47**	**159.31**

**conc**	**% inhibition**
12.5	0.0000
25	1.2658
50	44.1230
100	66.91
150	80.4702
200	100.00

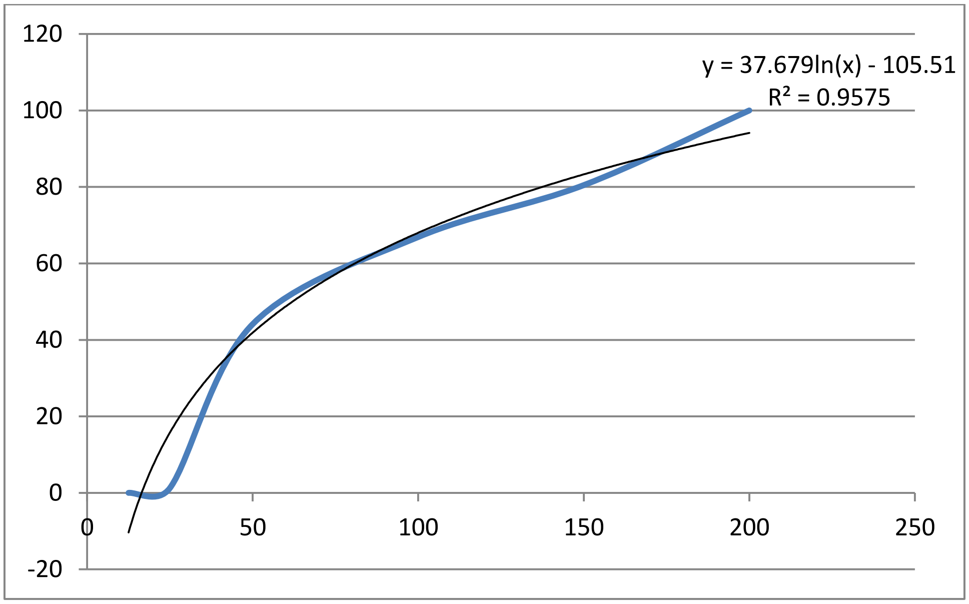

Second read								
			**12.5**	**25**	**50**	**100**	**150**	**200**
**Time**	**Control**	**tacrine**	**a**	**b**	**c**	**d**	**e**	**f**
0	0.052	0.049	0.050	0.050	0.051	0.074	0.090	0.126
1	0.054	0.049	0.051	0.052	0.052	0.073	0.087	0.124
2	0.056	0.049	0.053	0.053	0.053	0.071	0.085	0.129
3	0.058	0.049	0.054	0.055	0.054	0.073	0.087	0.125
4	0.060	0.049	0.056	0.056	0.055	0.072	0.086	0.122
5	0.062	0.048	0.058	0.058	0.056	0.073	0.087	0.120
6	0.063	0.048	0.060	0.058	0.057	0.073	0.094	0.122
7	0.065	0.049	0.060	0.059	0.058	0.076	0.089	0.119
8	0.067	0.049	0.062	0.060	0.059	0.077	0.093	0.123
9	0.068	0.048	0.063	0.061	0.061	0.076	0.090	0.120
10	0.069	0.049	0.062	0.061	0.061	0.076	0.092	0.118
**Gradient**	**0.0017**	**0.0000**	**0.0014**	**0.0011**	**0.0010**	**0.0004**	**0.0005**	**-0.0008**
**% inhibition**		**102.7125**	**18.0832**	**33.82**	**38.16**	**74.50**	**68.54**	**146.65**

**conc**	**% inhibition**
12.5	18.0832
25	33.82
50	38.16
100	74.50
150	68.54
200	100.00

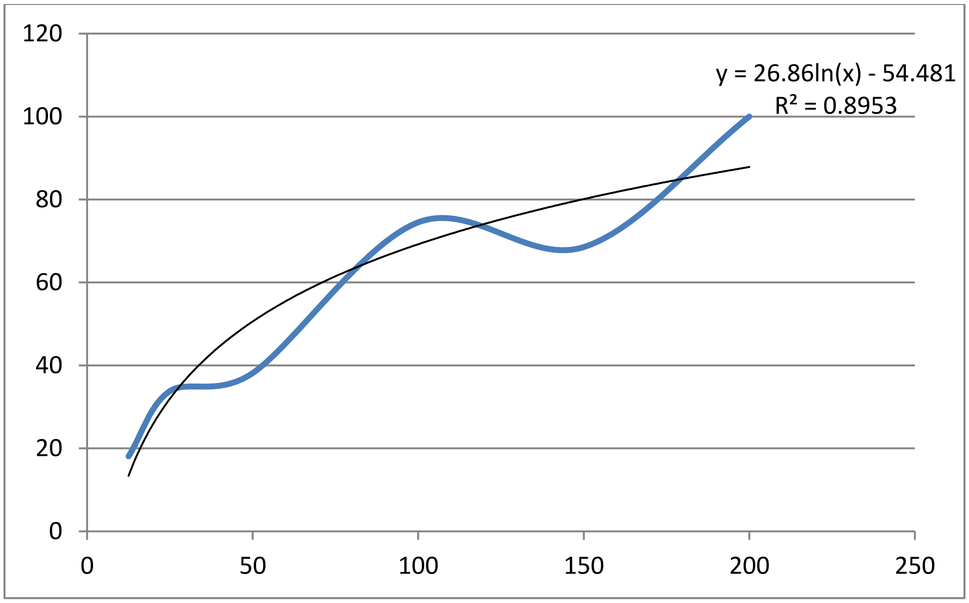

Third read									
			12.5	25	50	100	150	200
**Time**	**Control**	**tacrine**	**a**	**b**	**c**	**d**	**e**	**f**
0	0.052	0.049	0.049	0.051	0.051	0.070	0.101	0.124
1	0.054	0.049	0.051	0.052	0.052	0.070	0.103	0.125
2	0.056	0.049	0.052	0.053	0.053	0.069	0.106	0.121
3	0.058	0.049	0.054	0.054	0.053	0.070	0.107	0.124
4	0.060	0.049	0.055	0.056	0.054	0.070	0.106	0.122
5	0.062	0.049	0.056	0.057	0.055	0.071	0.104	0.127
6	0.063	0.049	0.058	0.058	0.056	0.072	0.107	0.122
7	0.065	0.049	0.059	0.059	0.056	0.072	0.108	0.119
8	0.067	0.049	0.060	0.060	0.058	0.074	0.108	0.128
9	0.068	0.049	0.061	0.061	0.057	0.075	0.107	0.119
10	0.069	0.049	0.062	0.063	0.059	0.074	0.107	0.121
**Gradient**	**0.0017**	**0.0000**	**0.0013**	**0.0012**	**0.0009**	**0.0006**	**0.0005**	**-0.0003**
**% inhibition**		**100.00**	**22.97**	**30.02**	**45.75**	**66.91**	**70.16**	**115.19**

conc	% inhibition
12.5	22.97
25	30.02
50	45.75
100	66.91
150	70.16
200	100.00

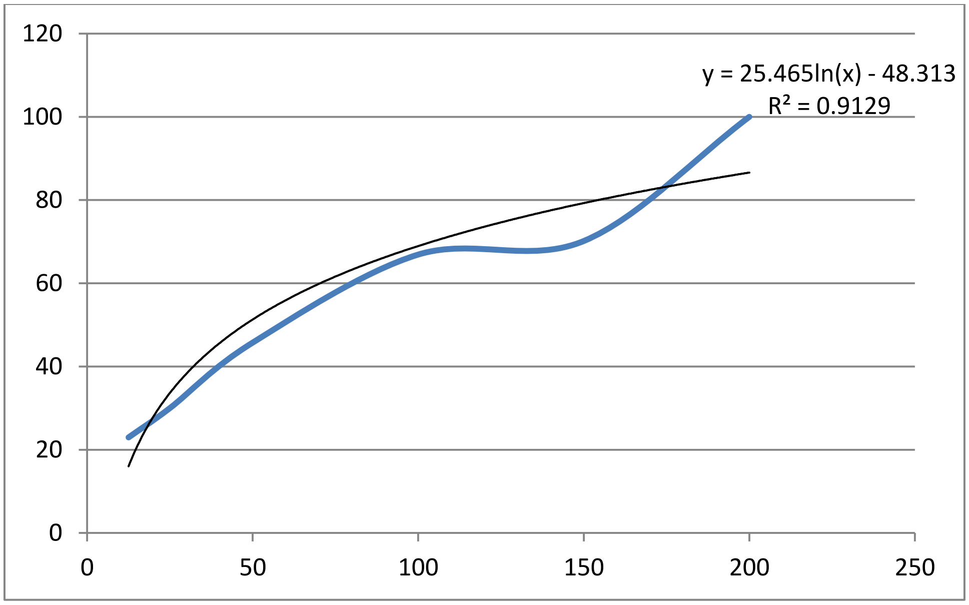

3.2.6 Raw data for IC_{50} calculation of compound **2n**.

First read								
			**25**	**50**	**100**	**150**	**200**	**250**
**Time**	**Control**	**tacrine**	**a**	**b**	**c**	**d**	**e**	**f**
0	0.055	0.049	0.052	0.052	0.050	0.052	0.051	0.051
1	0.057	0.050	0.054	0.054	0.051	0.053	0.052	0.052
2	0.059	0.049	0.056	0.056	0.053	0.054	0.053	0.053
3	0.062	0.050	0.058	0.058	0.054	0.055	0.054	0.054
4	0.064	0.049	0.060	0.060	0.055	0.056	0.054	0.054
5	0.066	0.050	0.062	0.063	0.056	0.057	0.056	0.055
6	0.068	0.050	0.064	0.063	0.058	0.058	0.056	0.056
7	0.070	0.050	0.066	0.066	0.058	0.060	0.057	0.056
8	0.072	0.050	0.067	0.067	0.060	0.061	0.059	0.057
9	0.074	0.050	0.069	0.069	0.061	0.062	0.059	0.058
10	0.075	0.050	0.070	0.070	0.062	0.063	0.060	0.059
**Gradient**	**0.0021**	**0.0001**	**0.0018**	**0.0018**	**0.0012**	**0.0011**	**0.0009**	**0.0007**
**% inhibition**		**96.05**	**10.83**	**11.27**	**42.02**	**45.53**	**56.52**	**63.98**

**conc**	**% inhibition**
25	10.8346
50	11.2738
100	42.0205
150	45.53
200	56.5154
250	63.98

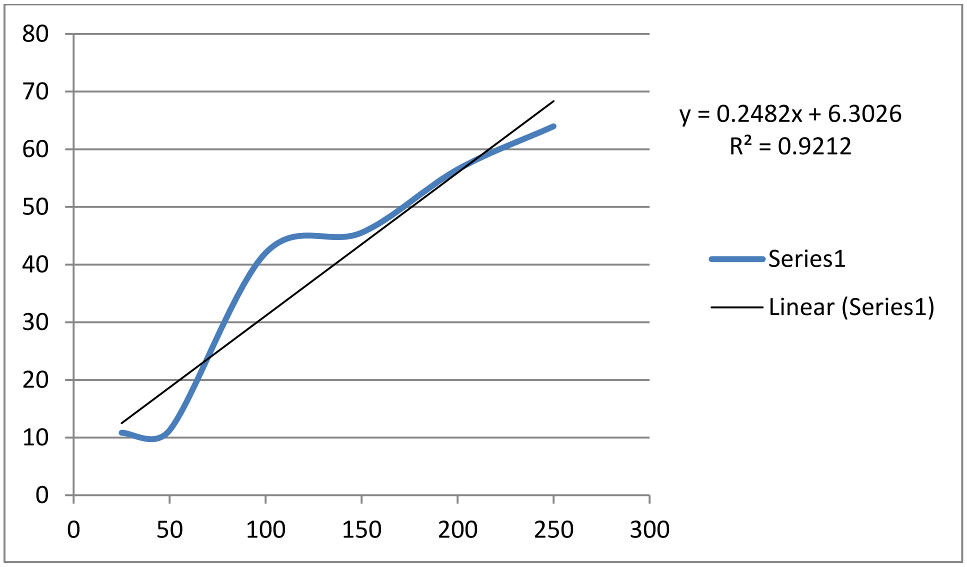

Second read								
			25	50	100	150	200	250
**Time**	**Control**	**tacrine**	**a**	**b**	**c**	**d**	**e**	**f**
0	0.055	0.049	0.049	0.049	0.050	0.051	0.054	0.052
1	0.057	0.049	0.051	0.051	0.052	0.052	0.055	0.052
2	0.059	0.049	0.053	0.053	0.053	0.053	0.055	0.053
3	0.062	0.049	0.054	0.055	0.055	0.054	0.056	0.053
4	0.064	0.049	0.056	0.057	0.057	0.055	0.057	0.054
5	0.066	0.049	0.059	0.058	0.058	0.056	0.058	0.055
6	0.068	0.050	0.060	0.059	0.060	0.058	0.059	0.056
7	0.070	0.050	0.062	0.061	0.061	0.058	0.061	0.056
8	0.072	0.050	0.063	0.063	0.062	0.060	0.061	0.058
9	0.074	0.050	0.064	0.064	0.064	0.061	0.062	0.058
10	0.075	0.050	0.065	0.066	0.065	0.062	0.063	0.060
**Gradient**	**0.0021**	**0.0001**	**0.0017**	**0.0016**	**0.0015**	**0.0011**	**0.0009**	**0.0008**
**% inhibition**		**93.4114**	**20.0586**	**20.50**	**27.53**	**45.97**	**54.76**	**61.79**

**conc**	**% inhibition**
25	20.0586
50	20.50
100	27.53
150	45.97
200	54.76
250	61.79

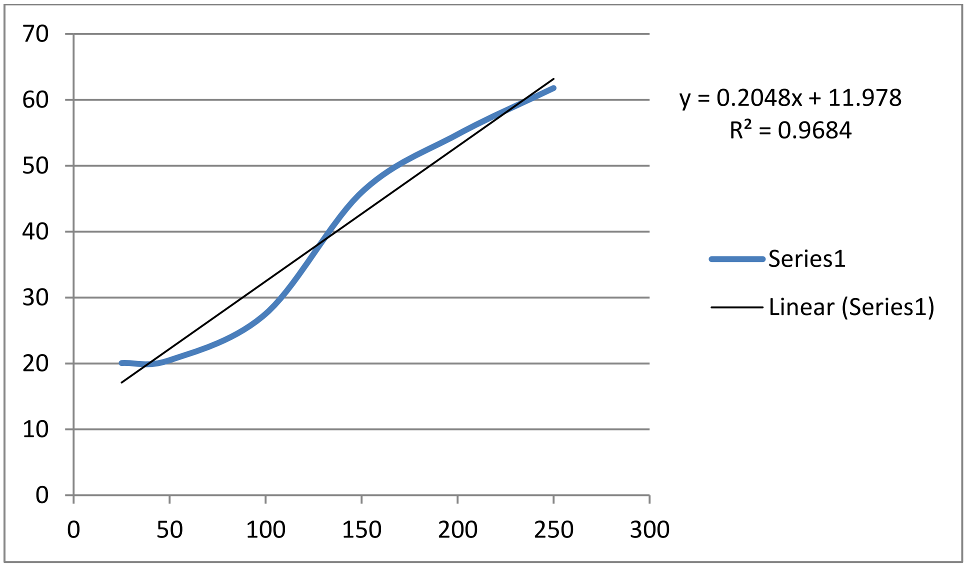

Third read								
			25	50	100	150	200	250
**Time**	**Control**	**tacrine**	**a**	**b**	**c**	**d**	**e**	**f**
0	0.055	0.049	0.049	0.049	0.053	0.051	0.051	0.052
1	0.057	0.049	0.051	0.051	0.054	0.052	0.052	0.054
2	0.059	0.050	0.053	0.053	0.056	0.053	0.052	0.053
3	0.062	0.050	0.055	0.055	0.057	0.054	0.053	0.054
4	0.064	0.050	0.056	0.056	0.058	0.055	0.054	0.055
5	0.066	0.050	0.058	0.058	0.059	0.056	0.055	0.056
6	0.068	0.050	0.060	0.059	0.060	0.057	0.056	0.056
7	0.070	0.050	0.062	0.061	0.061	0.059	0.057	0.057
8	0.072	0.050	0.064	0.062	0.062	0.059	0.057	0.058
9	0.074	0.050	0.065	0.063	0.064	0.060	0.059	0.059
10	0.075	0.050	0.067	0.065	0.065	0.061	0.059	0.060
**Gradient**	**0.0021**	**0.0001**	**0.0018**	**0.0015**	**0.0012**	**0.0010**	**0.0008**	**0.0007**
**% inhibition**		**96.05**	**13.47**	**25.33**	**43.78**	**50.81**	**59.15**	**63.98**

**conc**	**% inhibition**
25	13.47
50	25.33
100	43.78
150	50.81
200	59.15
250	63.98

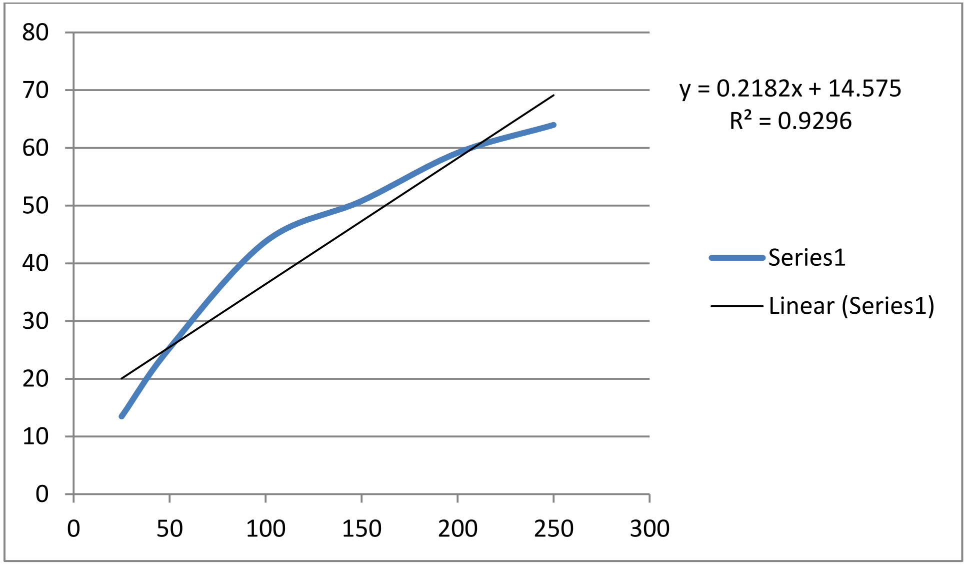

3.2.7 Summary of BChE IC_{50}.

IC_{50} (µg/mL)						
Compound	**2b**	**2g**	**2h**	**2j**	**2k**	**2n**
First read	181.0307	88.29188	137.6405	176.0572	62.00609	23.43803
Second read	198.9441	91.57915	135.4386	185.6543	48.90287	15.75192
Third read	183.2273	88.48448	149.8294	162.3511	47.4991	20.54371
Average	187.7341	89.45184	140.9695	174.6875	52.80269	19.91122
S.D.	8.093133	1.583152	7.259294	11.65833	2.747945	2.603234

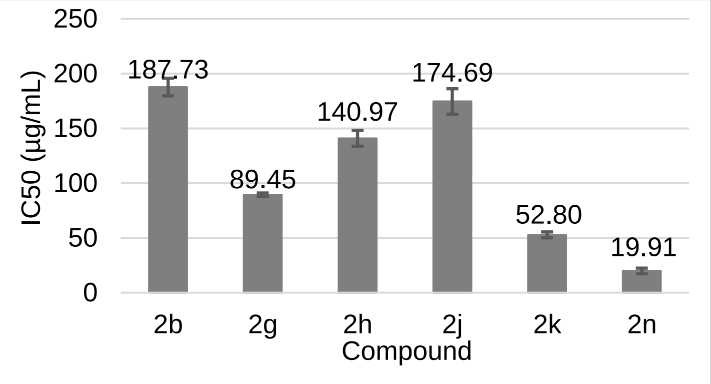

IC_{50} (µM)

Compound	**2b**	**2g**	**2h**	**2j**	**2k**	**2n**
First read	680.5665	310.8869	484.6497	579.1356	224.6598	96.85136
Second read	747.9102	322.4618	476.8964	610.7049	177.1843	65.09059
Third read	688.8246	311.5651	527.5682	534.0495	172.0982	84.89135
Average	705.7671	314.9713	496.3714	574.63	191.3141	82.27777
S.D.	36.72981	6.495859	27.29393	38.52581	28.98995	16.04088

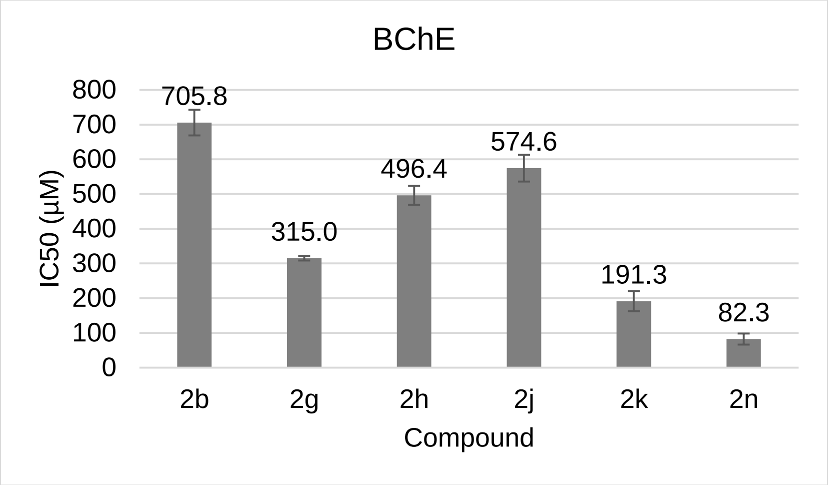

3.2.8 Statistical analysis for the IC_{50} of the test compound (BChE).

ANOVA

IC50

	Sum of Squares	df	Mean Square	F	Sig.
Between Groups	69848.265	5	13969.653	445.215	.000
Within Groups	376.528	12	31.377		
Total	70224.792	17			

Homogeneous Subsets

IC50

Tukey HSD

Compound	N	Subset for alpha = 0.05				
		1	2	3	4	5
2j	3	19.8888				
2k	3		54.4701			
2g	3			89.6876		
2h	3				1.3750E2	
2n	3					1.7891E2
2b	3					1.8858E2
Sig.		1.000	1.000	1.000	1.000	.341

Means for groups in homogeneous subsets are displayed.

References

1. Bernstein, J.; Davis, R.E.; Shimoni, L.; Chang, N.-L. Patterns in hydrogen bonding: Functionality and graph set analysis in crystals. *Angewandte Chemie International Edition in English* **1995**, *34*, 1555-1573.
